# Asymmetric biotic interchange across the Bering land bridge between Eurasia and North America

**DOI:** 10.1093/nsr/nwz035

**Published:** 2019-03-15

**Authors:** Dechun Jiang, Sebastian Klaus, Ya-Ping Zhang, David M Hillis, Jia-Tang Li

**Affiliations:** 1 CAS Key Laboratory of Mountain Ecological Restoration and Bioresource Utilization & Ecological Restoration and Biodiversity Conservation Key Laboratory of Sichuan Province, Chengdu Institute of Biology, Chinese Academy of Sciences, Chengdu 610041, China; 2 Department of Ecology and Evolution, J. W. Goethe University, Frankfurt am Main 60438, Germany; 3 State Key Laboratory of Genetic Resources and Evolution, Kunming Institute of Zoology, Chinese Academy of Sciences, Kunming 650223, China; 4 CAS Center for Excellence in Animal Evolution and Genetics, Chinese Academy of Sciences, Kunming 650223, China; 5 Department of Integrative Biology and Biodiversity Center, University of Texas at Austin, Austin 78712, USA

**Keywords:** multiple taxa, biogeography, Bering land bridge, Cenozoic

## Abstract

The exchange of biotas between Eurasia and North America across the Bering land bridge had a major impact on ecosystems of both continents throughout the Cenozoic. This exchange has received particular attention regarding placental mammals dispersing into the Americas, including humans after the last glacial period, and also as an explanation for the disjunct distribution of related seed plants in eastern Asia and eastern North America. Here, we investigate bi-directional dispersal across the Bering land bridge from estimates of dispersal events based on time-calibrated phylogenies of a broad range of plant, fungus and animal taxa. We reveal a long-lasting phase of asymmetrical biotic interchange, with a peak of dispersal from Asia into North America during the late Oligocene warming (26–24 Ma), when dispersal in the opposite direction was greatly decreased. Influx from North America into Asia was lower than in the opposite direction throughout the Cenozoic, but with peak rates of dispersal at the end of the Eocene (40–34 Ma) and again in the early to middle Miocene (16–14 Ma). The strong association between dispersal patterns and environmental changes suggests that plants, fungi and animals have likely dispersed from stable to perturbed environments of North America and Eurasia throughout the Cenozoic.

## INTRODUCTION

Much of the biodiversity we see today is the result of floral and faunal interchange between previously independently evolving biotas, followed by subsequent diversification [1]. For interchange between Eurasian and North American biotas, the Bering land bridge acted as a bottleneck, and it has been considered one of the most significant regions on Earth for paleogeographic and biogeographic studies [2]. Geological records suggest that the Bering land bridge connected northeastern Asia and northwestern North America for much of the time since the Cretaceous [2], which is also supported by the occurrence of closely related fossil plant taxa on both sides of the Pacific [3]. In contrast, there has been little or no land connection, and hence much less biotic exchange, between North America and Eurasia across the north Atlantic since the earliest Eocene [4–6].

Dispersal across the Bering land bridge has been proposed as an explanation for the appearance of many mammalian taxa in the North American fossil record [7], as well as for the disjunct distribution of related extant floras in eastern Asia and western North America. Time-calibrated phylogenies have been used to show that this American–Asian disjunction in seed plants had several origins: thermophilic, subtropical species dispersed during the early Eocene, followed by dispersal of more temperate species since the Miocene, with an estimated peak between 25 and 3 Ma, when mainly boreal groups associated with coniferous forests were exchanged [5,6,8,9]. The Bering land bridge remained a potential dispersal corridor until the Pliocene [10], with a last episode of dispersal of arctic groups associated with tundra vegetation during the last glacial period [11,12].

We used time-calibrated phylogenies of a broad range of plant, fungal, and animal groups to clarify the magnitude of dispersal between Asia and North America during the Paleogene and Neogene periods, with higher resolution and broader coverage of taxa than has been possible in previous studies [5,6,8,9]. We corrected our results for phylogenetic diversification, thus controlling for a potential bias in sampling that may strongly affect biogeographic inference, and investigated the robustness of our results. Specifically, we asked (i) if dispersal between the continents was a uniform process over time, or if there were periods of high and low dispersal; and (ii) if overall biotic interchange has been asymmetrical over time, as previously suggested for seed plants [6]. To answer these questions, we analyzed combined patterns of floral and faunal interchange across the Bering land bridge based on 54 phylogenies that document 92 biogeographic exchanges between Eurasia and North America from the late Cretaceous to the present. Based on estimates of phylogenetic divergence times for these biogeographic events, we calculated the ‘maximal number of potential dispersal events’ (MDEs) per million years [13] to reconstruct the magnitude and temporal pattern of dispersal between East Asia and North America. Finally, we discuss these dispersal patterns in the context of known paleoenvironmental changes.

## RESULTS

**Figure 1.**
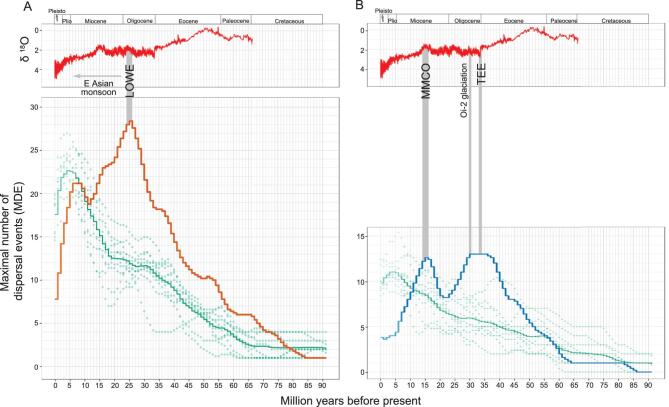
Rates of biotic exchange (A) from Asia to North America and (B) from North America to Asia, based on the maximum number of dispersal events (MDEs) and correlation of MDEs with climatic events as visualized by deep sea benthic foraminifera δ ^18^O data [22]. Random MDEs (green dots; green lines: mean) reflect the expectations based on the diversification pattern of the underlying phylogenies. LOWE: late Oligocene warming event; MMCO: mid-Miocene climatic optimum; Plio: Pliocene; Pleisto: Pleistocene; TEE: terminal Eocene event/Oi-1 glaciation.

The overall rate of biotic exchange was higher for dispersal from Eurasia to North America (compared to dispersal in the reverse direction) throughout the Cenozoic, although this asymmetry stems in part from the greater phylogenetic diversity of the respective taxa in Eurasia (Fig. 1). Once the underlying phylogenetic diversity (which reflects opportunity for dispersal) is taken into account, three periods of significant deviations in dispersal rates between Eurasia and North America became apparent (Fig. 1). Significantly elevated rates of dispersal (over null expectations based on phylogenetic diversity) from North America to Eurasia existed at the end of the Eocene (∼40–34 Ma; bootstrap support (BS) = 47), and again in the early to middle Miocene (∼16–14 Ma, BS = 99). Between these two peaks (throughout the Oligocene and early Miocene, ∼34–16 Ma), dispersal in the opposite direction (from Eurasia to North America) was significantly elevated, with a peak in Eurasian to North American dispersal around 26–24 Ma (BS = 85).

As expected, the resulting mean random MDEs for both dispersal directions showed a steady increase over time with a peak at 3–7 Ma and a subsequent slowing in lineage accumulation toward the present, most likely due to sampling bias, as younger lineages are more likely to escape sampling [14]. This pattern differed from the original, biogeographically inferred MDEs (Fig. 1). In contrast, if the number of dispersal events at a given age would strictly follow the underlying overall diversification pattern of the phylogenies, the peaks of original and mean random MDE should coincide. The decrease in mean random MDE after 7 Ma is most likely a result of incomplete sampling, as previously inferred based on avian phylogenies with near-complete species sampling [13], and so was not considered further. Our approach ensures that peaks of dispersal between Asia and North America are most likely controlled by biogeographical processes, and not by species and phylogeny sampling bias (i.e. overall phylogenetic diversification).

## DISCUSSION

Our analysis clearly supports the view that overall biotic interchange between Asia and North America was asymmetrical, with periods of higher and lower species exchange in each direction, as previously suggested for specific taxa like mammals [7] and seed plants [6]. We next considered how environmental conditions may have affected the rates and direction of intercontinental biotic interchange of different taxa between Eurasia and North America. We are aware that our selection of taxa had to follow operational criteria depending on the availability of sequence data and a reliable molecular clock calibration rather than conceptual considerations. Also, the potential environmental causes of variation in biotic interchange represent interpretations of the present results, although we tried to back them with evidence from the fossil record where available.

**Figure 2.**
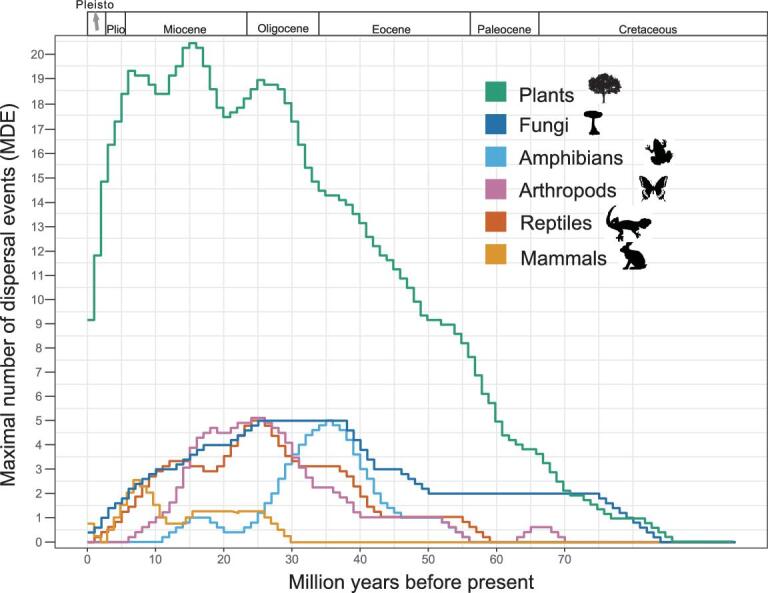
Biotic interchange between North America and Asia for specific taxon groups. The number of phylogenies (N_phy_) and the number of dispersal events (N_de_) for each taxon group were as follows: for plants: N_phy_ = 33, N_de_ = 57; for fungi: N_phy_ = 1, N_de_ = 5; for arthropods: N_phy_ = 5, N_de_ = 9; for amphibians: N_phy_ = 3, N_de_ = 6; for reptiles: N_phy_ = 7, N_de_ = 10; and for mammals: N_phy_ = 5, N_de_ = 5. Plio: Pliocene; Pleisto: Pleistocene.

As seen in Fig. 2, dispersal rates between Eurasia and North America of different taxonomic groups peaked at different times. In general, most intercontinental exchanges of arthropods, reptiles, amphibians, and fungi occurred much earlier (∼40–25 Ma) than the corresponding exchanges of plants and mammals (∼25–8 Ma).

The Bering land bridge served as a major dispersal corridor throughout the Paleocene and early Eocene when a continuous belt of boreotropical forests—a mixture of deciduous hardwoods and evergreen subtropical elements—extended over the entire Northern Hemisphere [15–17]. This finding is supported by middle Eocene dispersal events in both directions that are dominated by warm-adapted taxa including reptiles and arthropods, and, specifically for dispersal from North America to Asia, by amphibians (Fig. 2).

The first peak of North American to Eurasian dispersal in the late Eocene coincides with a pulse in climate cooling, as is evident from the pollen and plant megafossil record of North America [18–21]. The global climate deteriorated strongly at the Eocene–Oligocene boundary [22,23] and seasonally dry woodland savannahs spread in western North America [24]. Additionally, the northern boreotropical forests along the potential dispersal route across the Bering land bridge were replaced after the Eocene–Oligocene transition by mixed mesophytic forests, mixed deciduous hardwoods and coniferous forests [21,25]. This environmental change may have allowed better-adapted species to introgress into Asia. The Oi-2 Oligocene cooling event at ca. 30 Ma [26]—although considered minor on a global scale—coincides with the beginning of a decrease in biotic influx into Eurasia from North America. The climate-induced turnover of the North American source biota at this time offers an explanation for this drop in dispersal rates. However, it has to be noted that the early dispersal peak from North America to Eurasia is not as robustly supported by the present data as the other two dispersal peaks. Also, as the terminal Eocene event affected both North America and Asia, it remains difficult to explain the unidirectionality of dispersal by climatic changes alone.

The decrease in biotic exchange from North America to Eurasia beginning in the early Eocene contrasts with a simultaneous increase in exchange in the opposite direction. A likely explanation is that diverse environments expanded in East Asia during this time—contrary to the situation in North America—and that the Eurasian biota was able to invade the more disturbed North American habitats. The retraction of the Chinese arid belt northwestward during the Oligocene and the establishment of deciduous, humid forests [27] that continued far northward as broad-leaved forests and cypress swamps [28], may have formed a coastal dispersal corridor for the species-rich Asian biota. The peak of biotic influx coincides exactly with the late Oligocene warming event [23] and is primarily a result of increased dispersal of reptile taxa at this time (Fig. 2).

The decline in dispersal from Asia to North America during the Miocene coincides with the establishment of monsoonal climates in East Asia at ∼22 Ma [29]. Whereas warm and humid environments prevailed throughout the Miocene of northeastern China [27], the Russian Far East experienced continued cooling [30,31]. This is consistent with most temperate seed plants showing disjunct biogeographic patterns between eastern Asia and eastern North America since the Miocene [6], following cooling of the Northern Hemisphere [32].

Dispersal from North America into Asia increased again during the mid-Miocene climatic optimum (MMCO). This increase was once again associated with exchanges of warm-adapted taxa like amphibians and reptiles, which showed elevated MDEs during this period (Fig. 2). Favorable environmental conditions in Northern America during the MMCO are also reflected by elevated mammal diversity in the fossil record [33,34].

Shifting environmental conditions in North America and Eurasia, combined with associated phylogenetic diversification of taxa in these areas, thus potentially account for the most of the shifting asymmetries of biotic interchange between North America and Eurasia. Although rates of exchange from Eurasia to North America have generally been higher than the reverse throughout the Cenozoic, that asymmetry was most evident during the late Oligocene warming event, and was least evident during cooler periods at the end of the Eocene and in the Miocene.

While climatic conditions oscillated frequently throughout the Cenozoic, we could show that the major climatic events at the latest Eocene, late Oligocene and middle Miocene have most likely left their traces in species’ phylogenies. We hope that the present results, and our interpretation of the latter, can stimulate discussion on the importance of environmental variables for biotic interchange. An exhaustive comparative look at the fossil record of East Asia and North America, also for non-mammalian taxa, may yield and independent line of evidence, either supporting or weakening the present biogeographic hypotheses, and the accuracy of the phylogeographic approach that we have developed.

## MATERIALS AND METHODS

### Phylogenetic inference and divergence time estimation

We re-calculated divergence times of 54 phylogenetic data sets using sequences obtained from GenBank (see Supplementary Information for details). We selected phylogenies that met the following criteria: (i) the taxonomic rank covered by the study should be above genus level to control divergence times, and (ii) the taxon distribution should cover East Asia and North America. The resulting data set was biased towards plant taxa (61% of phylogenies, in contrast to 28% vertebrates, 9% invertebrates and 2% fungi). However, as vegetation is a proxy for environmental conditions [35], this does not represent a systematic drawback in the context of the questions that we asked. Sequences were aligned with MUSCLE [36] (gap opening penalty = −800; gap extension penalty = −500) as implemented in MEGA v.6.0 [37]. Divergence time estimations were conducted in BEAST v.1.7.5 [38], applying a Yule tree prior and an uncorrelated relaxed molecular clock model. The details of each calibration scheme are given in the Supporting Information. Data sets were partitioned and the best-fitting model of sequence evolution applied as suggested by PartitionFinder v.1.1.1 [39], based on the Bayesian information criterion. The log-files of the Bayesian analyses were checked in Tracer v.1.5 [40] for autocorrelation and stationarity of the sampled parameters. We ran the analyses for 200 M iterations and sampled parameters and trees every 5,000th iteration; the first 10% of samples were discarded as ‘burn-in’. We investigated the influence of prior assumptions on the results by sampling from the prior only.

### Ancestral area estimation

To estimate the temporal patterns of dispersal maxima and minima, we compiled 92 credibility intervals of divergence times (Supplementary Table S1) at nodes for which we inferred dispersal/range shifts between Asia and North America. Biogeographical inference was conducted in R v.3.1.1 [41] using the package BioGeoBEARS v.0.2.1 [42]. We applied a dispersal-extinction-cladogenesis model of range evolution [43], with tips pruned to species-level and—in case these were only distantly related to the ingroup—outgroups removed for the biogeographical analyses. The area coding generally follows the original studies with few exceptions (see Supplementary Information for details).

### Biogeographical meta-analyses

The biogeographical meta-analyses were based on the consideration that—under the assumption that dispersal events have the same abiotic causes—dispersal events are more likely to have happened when the corresponding divergence time intervals overlap, in line with the concept of geodispersal [44]. This is reflected by the MDEs per million years that were calculated by summing up potential dispersal events over all data sets through time―based on the credibility intervals (95% highest posterior densities, rounded to the full million) of corresponding divergence times―using time slices of one million years [13]. All dispersal events were treated as independent, in addition to when they originated from the same phylogeny, and hypothetical transoceanic long-distance dispersal was not taken into account (but we pre-selected against studies that inferred transoceanic dispersal rather than terrestrial range expansion). We discriminated between dispersal direction, that is, from Asia to North America (61 events) and vice versa (31 events). Additionally, we calculated MDEs for fungi (5 events), plants (57 events), arthropods (9 events), amphibians (6 events), reptiles (excluding birds) (10 events) and mammals (5 events). To avoid over-interpretation of slight (possibly stochastic) changes in MDE, we smoothed the data by calculating mean values in a sliding window approach with a time frame of 5 Myr.

### Comparison with phylogenetic diversification

The accuracy of phylogeny-based meta-analytical approaches in biogeography [13,45,46] has questioned due to a potential bias in phylogeny sampling, because the majority of published phylogenetic studies deal with taxon levels (genera, subfamilies and families) that in most cases only diversified since the Miocene [47,48]. If true, this inflation of Neogene diversification would consequently lead to an excess of inferred biogeographic events during this period and, in combination with a predicted steady increase of lineages through time [49], result in the previously described dispersal patterns. To rule out this possibility and to investigate the patterns resulting from the underlying phylogenetic information alone (in contrast to sampling node age credibility intervals based on biogeographic estimates), we calculated MDEs from a random sample of node age credibility intervals for the present data set. Specifically, we drew at random the same number of divergence time credibility intervals from each of the phylogenies as the originally inferred number of dispersal events with the ‘sample’ function in R 3.3.1. This ensures that each phylogeny is weighted equally as in the original study, as some phylogenies contained multiple dispersal events. These random node credibility intervals were then used to calculate raw ‘random MDEs’ per million years for each dispersal direction. Finally, we calculated the mean from 10 such random data replicates that was, as in the original study, additionally subjected to smoothing by a sliding window approach.

To assess the robustness of the inferred dispersal patterns we followed a bootstrap approach. We generated 100 bootstrap replicates for each dispersal direction by re-sampling dispersal times’ credibility intervals without replacement using the ‘sample’ function in R (Supplementary Fig. S1). We then inferred the frequency of co-occurrence between bootstrap replicate MDE peaks and MDE peaks of the original data (26–24 Ma for MDE from North America to Asia; 40–34 and 16–14 Ma from Asia to North America). This frequency represents the BS value of the respective MDE peaks.

## Supplementary Material

nwz035_Supplemental_File
