# Supplementary material for: Asymmetric biotic interchange across the Bering land bridge between Eurasia and North America
Source: Natl Sci Rev. 2019 Mar 15;6(4):739–45. doi: 10.1093/nsr/nwz035 (PMC8291635; doi:10.1093/nsr/nwz035)

Supplementary Data:

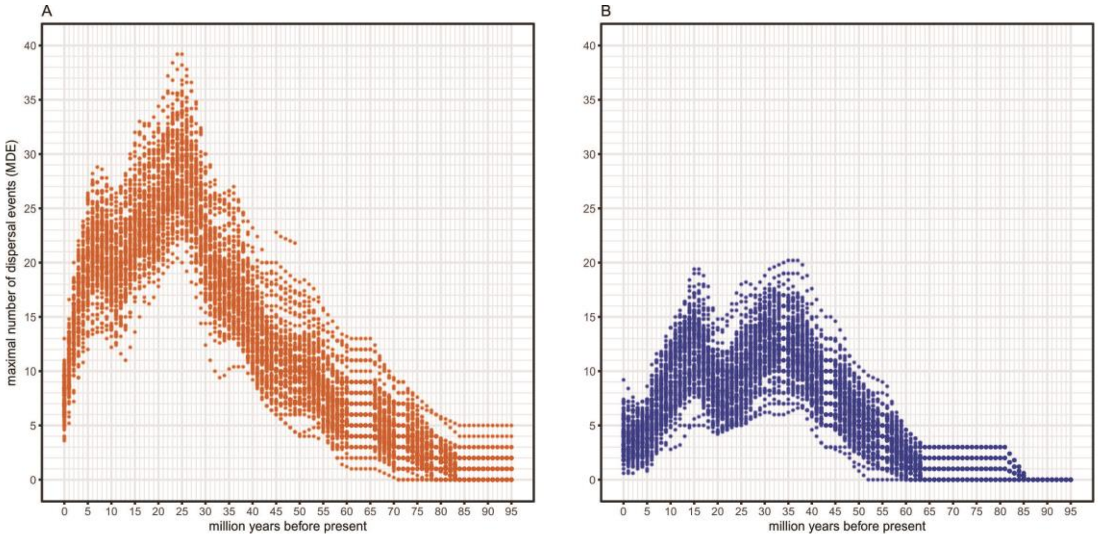

Fig. S1: Results of the bootstrap analyses based on 100 MDE replicates. (A) From Asia to North America; (B) from North America to Asia.

Table S1: Inferred age of dispersal events between eastern Asia (AS) and North America (NA). Given are lower and upper bounds of the 95% highest posterior density in million years before present.

**Taxon**	**95% HPD**		**Dispersal direction**
	**Upper bound**	**Lower bound**	
Woodwardia (Blechnaceae, Polypodiales)	54	31	NA→AS
Woodwardia (Blechnaceae, Polypodiales)	40	15	AS→NA
Picea (Pinaceae, Coniferales)	60	34	NA→AS
Picea (Pinaceae, Coniferales)	63	25	NA→AS
Osmorhiza (Apiaceae, Apiales)	11	6	AS→NA
Prunus (Rosaceae, Rosales)	20	2	AS→NA
Prunus (Rosaceae, Rosales)	26	3	AS→NA
Prunus (Rosaceae, Rosales)	8	25	AS→NA
Prunus (Rosaceae, Rosales)	44	18	AS→NA
Patis and *Ptilagrostis* (Poaceae, Stipeae)	7	2	AS→NA
Aristolochia (Aristolochiaceae, Aristolochiales)	49	12	NA→AS
Astilbe (Saxifragaceae, Rosales)	24	10	AS→NA
Ranunculeae (Ranunculaceae, Ranunculales)	10	2	AS→NA
Ranunculeae (Ranunculaceae, Ranunculales)	10	3	AS→NA
Ranunculeae (Ranunculaceae, Ranunculales)	7	1	NA→AS
Ranunculeae (Ranunculaceae, Ranunculales)	4	0	NA→AS
Scrophularia (Scrophulariaceae, Tubiflorae)	29	28	AS→NA
Thuja (Cupressaceae, Pinales)	60	59	AS→NA
Thuja (Cupressaceae, Pinales)	48	14	NA→AS
Hamamelis (Hamamelidaceae, Saxifragales)	33	6	AS→NA
Circaea (Onagraceae, Myrtales)	31	16	AS→NA
Circaea (Onagraceae, Myrtales)	9	2	AS→NA
Circaea (Onagraceae, Myrtales)	12	4	AS→NA
Zizania (Poaceae, Cyperales)	3	0	NA→AS
Zizania (Poaceae, Cyperales)	4	2	NA→AS
Toxicodendron (Anacardiaceae, Sapindales)	21	9	AS→NA
Toxicodendron (Anacardiaceae, Sapindales)	42	23	NA→AS
Phryma (Phrymaceae, Tubiflorae)	4	2	NA→AS
Mitchelleae (Rubiaceae, Rubiales)	20	6	AS→NA
Calycanthus (Calycanthaceae, Ranales)	69	5	AS→NA
Lathyrus (Leguminosae, Rosales)	2	0	AS→NA
Taxus (Taxaceae, Taxales)	16	7	NA→AS
Thuja (Cupressaceae, Pinales)	18	3	AS→NA
Rhus(Anacardiaceae, Sapindales)	30	17	NA→AS
Pseudotsuga (Pinaceae, Pinales)	33	29	NA→AS
Orontioideae (Alismatales, Dicotyledons)	29	2	AS→NA
Kelloggia (Rubiaceae, Rubiales)	18	1	AS→NA
Aesculus (Sapindaceae, Sapindales)	57	54	AS→NA

Aesculus (Sapindaceae, Sapindales)	57	25	AS→NA
Aesculus (Sapindaceae, Sapindales)	70	16	AS→NA
Sect. *Quinquefoliae* (*Pinus*, Pinaceae, Pinales)	16	3	AS→NA
Sect. *Quinquefoliae* (*Pinus*, Pinaceae, Pinales)	59	30	NA→AS
Sect. *Quinquefoliae* (*Pinus*, Pinaceae, Pinales)	51	25	NA→AS
Sect. *Quinquefoliae* (*Pinus*, Pinaceae, Pinales)	31	11	NA→AS
Sect. *Quinquefoliae* (*Pinus*, Pinaceae, Pinales)	21	6	NA→AS
Juniperus (Cupressaceae, Pinales)	99	25	AS→NA
Juniperus (Cupressaceae, Pinales)	38	15	AS→NA
Juniperus (Cupressaceae, Pinales)	17	5	AS→NA
Abies (Pinaceae, Pinales)	34	20	AS→NA
Abies (Pinaceae, Pinales)	35	8	AS→NA
Peracarpeae (Campanulaceae, Campanulales)	19	12	AS→NA
Peracarpeae (Campanulaceae, Campanulales)	12	5	AS→NA
Peracarpeae (Campanulaceae, Campanulales)	6	2	AS→NA
Calocedrus (Cupressaceae, Pinales)	32	21	AS→NA
Altingiaceae (Saxifragales, Dicotyledons)	76	36	AS→NA
Rhodiola (Crassulaceae, Saxifragales)	10	5	AS→NA
Dryopteridaceae (Polypodiales, Equisetopsida)	85	39	NA→AS
Rana (Ranidae, Anura)	45	25	AS→NA
Rana (Ranidae, Anura)	55	35	AS→NA
Hyla (Hylidae, Anura)	42	30	NA→AS
Hyla (Hylidae, Anura)	21	15	NA→AS
Bufonidae (Anura, Amphibian)	41	32	NA→AS
Bufonidae (Anura, Amphibian)	39	29	NA→AS
Natricine (Colubridae, Serpentiformes)	28	23	AS→NA
Rat snakes (Colubridae, Serpentiformes)	58	32	AS→NA
Rat snakes (Colubridae, Serpentiformes)	42	26	AS→NA
Geoemydidae (Tesudines, Reptilia)	16	5	AS→NA
Geoemydidae (Tesudines, Reptilia)	25	9	NA→AS
Emys (Emydidae, Testudines)	17	7	NA→AS
Elapoidea (Serpentiformes, Reptilia)	31	21	AS→NA
Plestiodon (Scincidae, Lacertiformes)	40	25	AS→NA
Viperidae (Squamata, Reptilia)	30	20	AS→NA
Viperidae (Squamata, Reptilia)	18	11	NA→AS
Dipodoidea (Rodentia, Mammalia)	30	16	AS→NA
Mustela (Mustelidae, Carnivora)	1	0	AS→NA
Microtus (Circetidae, Rodentia)	1	0	AS→NA
Myotis (Vespertilionidae, Chiroptera)	13	7	AS→NA
Leporidae (Lagomorpha, Mammalia)	11	7	NA→AS
Melaphidina (Aphidoidea, Insecta)	55	29	AS→NA
Limnogonus (Gerridae, Hemiptera)	39	38	AS→NA
Aporini (Pompilidae, Hymenoptera)	22	15	NA→AS
Papilionidae (Lepidoptera, Insecta)	29	18	AS→NA

Papilionidae (Lepidoptera, Insecta)	32	17	AS→NA
Papilionidae (Lepidoptera, Insecta)	38	24	NA→AS
Papilionidae (Lepidoptera, Insecta)	19	10	NA→AS
Apaturinae (Nymphalidae, Lepidoptera, Insecta)	33	22	AS→NA
Apaturinae (Nymphalidae, Lepidoptera, Insecta)	28	16	AS→NA
Sect. *Phalloideae* (*Amanita*, Amanitaceae, Agaricales)	78	25	AS→NA
Sect. *Phalloideae* (*Amanita*, Amanitaceae, Agaricales)	49	10	AS→NA
Sect. *Phalloideae* (*Amanita*, Amanitaceae, Agaricales)	41	3	AS→NA
Sect. *Phalloideae* (*Amanita*, Amanitaceae, Agaricales)	83	17	AS→NA
Sect. *Phalloideae* (*Amanita*, Amanitaceae, Agaricales)	41	6	NA→AS

Supplementary Information: Details on phylogenetic and biogeographic analyses

We collected 54 phylogenetic data using sequences obtained from GenBank, re-calculated their divergence times, and estimated ancestral area of them. Dispersal events from these results were used to conduct biogeographical meta-analyses in this study. On account of differences in fossil calibration points, outgroups, and gene fragments used in our study, results of calibrated phylogenies may be different from published ones. In case our calibration departs dramatically from the published divergence times, we also compared them in this section. Phylogenetic and biogeographic analyses details were listed below:

***Woodwardia* (Blechnaceae, Polypodiales)**

The phylogeny of *Woodwardia* published by Li et al. [50] was re-analysed using *rbcL* and *rps4* sequences. The alignment length of data set was 1323 bp for *rbcL* and 1007 bp for *rps4*, respectively. To calibrate the phylogeny, we used the earliest divergence of the woodwardioid ferns (55.8 Ma; log-normal calibration density; offset = 54 Ma, s.d. = 0.5 Ma, 5–95% interquantile range = 54.8–58.15 Ma) as the calibration point because fossils assignable to *Woodwardia* are known from throughout the Cenozoic [51]. We applied an uncorrelated lognormal relaxed molecular clock using a HKY substitution model for *rbcL* and *rps4* data set. The resulting mean substitution rate was 0.05% per Ma (95% CI = 0.04–0.07% per Ma). We defined the biogeographic areas as East Palearctic, West Palearctic, and North America. Root ages of this taxus were 56.78 Ma (95% HPD: 54.46–58.08Ma) for our calibration and 56.51±2.89 Ma for the published divergence times.

***Picea* (Pinaceae, Coniferales)**

The phylogeny of genus *Picea* published by Ran et al. [52] was re-analysed using two chloroplast (*trnC-trnD* and *trnT-trnF*) and one mitochondrial (*rps3*) as well as three single-copy nuclear genes (*FTL1*, *LFY* and *s.d.D1*). The data set included 3511 bp for *FTL1*, 3063 bp for *LFY*, 2332 bp for *s.d.D1*, 6614 bp for *rps3*, and 4001 bp for combined sequences of two chloroplast genes. Following the study of Lockwood et al. [53], we used two fossil calibration points. For the root constraint (the most recent

common ancestor of the three genera *Cathaya*, *Picea* and *Pinus*), we used a standard deviation of 0.5, a prior mean of 2.38, and an offset at 131.1 Ma based on the earliest fossil record of *Picea burtonii* from British Columbia [54]. In addition, the earliest fossil of the *Pinus* subgenus *Strobus* [55] was used to calibrate the divergence time between two subgenera of *Pinus*, represented by the species *P. thunbergii* and *P. strobus* (mean = 2.38, s.d. = 0.25, offset at 73 Ma). We applied a uncorrelated lognormal relaxed molecular clock using a HKY+G substitution model for *FTL1* data set, a GTR+I+G substitution model for *LFY* data set, a GTR+G substitution model for *s.d.D1* data set, a GTR+I+G substitution model for rps3 data set, and a GTR+G substitution model for combined data set of two chloroplast genes. The resulting mean substitution rate was 0.04% per Ma (95% CI = 0.03–0.05% per Ma). We defined the biogeographic areas as East Palearctic, West Palearctic, and North America. Root ages of this taxus were 83.22 Ma (95% HPD: 78.95–88.78 Ma) for our calibration and 83.36 Ma (95% HPD: 78.35–89.16 Ma) for the published divergence times.

***Osmorhiza* (Apiaceae, Apiales)**

The phylogeny of genus *Osmorhiza* published by Yi et al. [56] was re-analysed using nine chloroplast markers and two nuclear loci. The chloroplast data set included *atpB-rbcL*, *ndhF*, *psbA-trnH*, *psbE-petL*, *rpl16*, *rps16*, *trnL-F*, *rpl32-trnL*, and *trnQ-rps16* (9352 bp total). The nuclear data set included *ITS* and *ETS* (891 bp total). There is no fossil record for *Osmorhiza* and its close relatives, and fossils are rare in the family Apiaceae [57,58]. Results of Banasiak et al. [57], which calculated the divergence times of Apiaceae subfamily Apioideae, showed that the divergence between the [*Chaerophyllum* - *Sphallerocarpus*] clade and the [*Anthriscus* - *Geocaryum* - *Kozlovia* - *Myrrhis* - *Neoconopodium* - *Krasnovia-Osmorhiza*] clade was estimated to be 16.43 Ma (95% HPD: 12.65–18.64 Ma), and between *Osmorhiza* and the [*Anthriscus* - *Geocaryum* - *Kozlovia* - *Myrrhis* - *Neoconopodium* - *Krasnovia*] clade to be 11.08 Ma (95% HPD: 7.85–12.65 Ma). In this study, we used a log-normal prior for dating *Osmorhiza* clades, with the divergence of the *Chaerophyllum* clade from the [*Anthriscus* - *Myrrhis* - *Osmorhiza*] clade set at a stdev of 0.4, a prior mean

of 0.01, and an offset of 15.4 Ma, and the divergence of the [*Anthriscus* - *Myrrhis*] clade and the *Osmorhiza* clade set at a s.d. of 0.4, a prior mean of 0.08, and an offset of 10 Ma. We applied an uncorrelated lognormal relaxed molecular clock using a GTR+I+G substitution model for the chloroplast data set, and a GTR+I substitution model for the nuclear data set. The resulting mean substitution rate was 0.12% per Ma (95% CI = 0.09–0.16% per Ma). We defined the biogeographic areas as East Asia, Northern America, and Southern America. Root ages of this taxus were 11.34 Ma (95% HPD: 10.44–12.89 Ma) for our calibration and 5.51 Ma for the published divergence times.

Prunus (Rosaceae, Rosales)

The phylogeny of *Prunus* published by Chin et al. [59] was re-analyzed using plastid genes. The data set included 806 bp for *matK*, 574 bp for *rbcL*, 522 bp for *trnL-F*, and 1570 bp for *trnS-G*. We applied a calibration ages to the divergence node of *Prunus* (log-normal calibration density; mean = 1.0 Ma, s.d. = 0.9 Ma, offset = 56 Ma) from Wang et al. [60] as the calibration point. We applied an uncorrelated lognormal relaxed molecular clock using a HKY+G substitution model for the *matK* data set, a GTR+I substitution model for the *rbcL* data set, a GTR substitution model for the *trnL-F* data set, and a HKY+G substitution model for the *trnS-G* data set. The resulting mean substitution rate was 0.03% per Ma (95% CI = 0.027–0.047% per Ma). We defined the biogeographic areas as East Asia, Europe, western Asia, North America, Southeast Asia, South America and Africa. Root ages of this taxus were 58.43 Ma (95% HPD: 56.12–65.49 Ma) for our calibration and 60.7 Ma (95% HPD: 56.4–64.7 Ma) for the published divergence times.

***Patis* and *Ptilagrostis* (Poaceae, Stipeae)**

The origin and dispersal of two genera, *Patis and Ptilagrostis* which published by Romaschenko et al. [61] was re-analysed using four plastid DNA sequences and two nuclear DNA sequences. The plastid data set included 783 bp for *ndhF*, 826 bp for *rpl32-trnL*, 771 bp for *rps16-trnK*, and 786 bp for *rps16 intron*.The nuclear data set

includes 610 bp for *ITS* and 304 bp for *At103*. To calibrate the phylogeny, we used the earliest fossil of [*Stipeae - Stipidium*] which morphologically resembles contemporary *Hesperostipa* and *Piptochaetium* [62–64]. The calibration was done using a 21 Ma offset and standard deviation of 0.8, which resulted in 95% prior distribution of 21.27–24.73 Ma. We applied an uncorrelated lognormal relaxed molecular clock using a GTR+I+G substitution model for the *ITS* data set, a HKY+G substitution model for the *At103* data set, a GTR+G substitution model for the *ndhF* data set, a HKY+G substitution model for the *rpl32-trnL* data set, a HKY+G substitution model for the *rps16-trnK* data set, and a HKY substitution model for the *rps16 intron* data set. The resulting mean substitution rate was 0.07% per Ma (95% CI = 0.06–0.087% per Ma). We defined the biogeographic areas as East Palearctic, West Palearctic, North America and South America. Root age of these taxa was 67.39 Ma (95% HPD: 45.21–96.27 Ma) for our calibration and there was no exact data of the dispersal event for the published divergence times.

***Aristolochia* (Aristolochiaceae, Aristolochiales)**

The phylogeny of *Aristolochia* published by González et al. [65] was re-analysed using *matK* gene, *trnK* intron and *trnK-psbA* spacer sequences (3152 bp total). Although dated fossils exist for *Aristolochia*, none of them can be placed unambiguously within a particular clade because they correspond only to leaves. Thus, we took calibration points from closely related Piperales lineages. In accordance with Symmank et al. [66], we used the Saururaceae fossil *Saururus tuckerae* from the mid-Eocene (48.5 Ma; log-normal calibration density, mean = 0.4 Ma, s.d. = 0.01 Ma, offset = 47 Ma) [67] as well as the pollen fossil *Lactori pollenites* (92 Ma; log-normal calibration density, mean = 0.0 Ma, s.d. = 0.01 Ma, offset = 91 Ma) [68,69]. These two fossils are the only two well-dated and unambiguously assigned records in Piperales. We applied an uncorrelated lognormal relaxed molecular clock using a GTR+G substitution model for the whole data set. The resulting mean substitution rate was 0.078% per Ma (95% CI = 0.055–0.1% per Ma). We defined the biogeographic areas as East Palearctic, West Palearctic and North America. Root ages

of this taxus were 46.81 Ma (95% HPD: 26.5–73.87 Ma) for our calibration and 30.24 Ma (95% HPD: 19–43 Ma) for the published divergence times.

***Astilbe* (Saxifragaceae, Rosales)**

Phylogenetic analyses were conducted for *Astilbe* (Saxifragaceae) by Zhu et al. [70] using sequences of nuclear ribosomal internal transcribed spacer (*ITS*; 726 bp) and plastid sequences (2210 bp total) including *matK*, *trnL-trnF* and *psbA-trnH* regions. Based on the oldest fossil of Saxifragales from the Late Cretaceous of New Jersey, USA, with fossilized flowers and fruits (89.3–93.5 Ma, [71]), we calibrated the stem age of the [Iteaceae+Pterostemonaceae] clade using a mean age of the fossil as 91.4 Ma with the standard error of 1.0 to roughly match the fossil age of 89.3–93.5 Ma. Based on leaf fossils of *Ribes* L. of Grossulariaceae (50 Ma) and leaf records of *Ribes* (45 Ma), we calibrated the minimum stem age of Grossulariaceae to be 50 Ma with the standard error of 0.6 according to Wehr & Hopkins [72] and Hermsen [73]. We applied an uncorrelated lognormal relaxed molecular clock using a GTR+I+G substitution model for the *ITS* data set, and a GTR+G substitution model for the plastic sequences data set. The resulting mean substitution rate was 0.14% per Ma (95% CI = 0.12–0.16% per Ma). We defined the biogeographic areas as eastern Eurasia and North America. Root ages of this taxus were 28.28 Ma (95% HPD: 18.67–36.96 Ma) for our calibration and 20.69 Ma (95% HPD: 12.14–30.22 Ma) for the published divergence times.

Ranunculeae (Ranunculaceae, Ranunculales)

The phylogeny of Ranunculeae published by Emadzade &Hörandl [74] was re-analysed using *ITS*, *matK-trnK* and *psbJ-petA* sequences. The data set included 730 bp for *ITS*, 1958bp for *matK-trnK*, and 649 bp for *psbJ-petA*. The age of the split of *Ranunculus* (Ranunculeae) and *Clematis* (Anemoneae) estimated as 46.6 Ma, based on a fossil calibrated study of Ranunculales [75]. We used normal prior distributions for this point at a mean of 46 Ma and a stdev of 2 Ma. We used a minimum age for *Myosurus* of 23 Ma with an exponential prior distribution with an offset of 23 Ma and

a mean of 1 Ma. Within *Ranunculus*, we further used the divergence time between *Ranunculus carpaticola* and *Ranunculus notabilis* (0.914 Ma) according to Nei [76]. A normal distribution was used for the age of the split with the mean of 0.914 Ma. We applied an uncorrelated lognormal relaxed molecular clock using a GTR substitution model for the *ITS* data set, a GTR+I+G substitution model for the *matK-trnK* data set, and a HKY+G substitution model for the *psbJ-petA* data set. The resulting mean substitution rate was 0.33% per Ma (95% CI = 0.22–0.44% per Ma). We defined the biogeographic areas as Asia, Europe, North America, South America, Africa and Australia. Root ages of this taxus were 20.45 Ma (95% HPD: 13.62–29.01 Ma) for our calibration and 18.11 Ma for the published divergence times.

***Scrophularia* (Scrophulariaceae, Tubiflorae)**

The phylogeny of genus *Scrophularia* L. published by Scheunert and Heubl [77] was re-analysed using nuclear ribosomal *ITS*. The data set included 590 bp total. To calibrate the phylogeny, we decided to follow Bremer et al. [78], which were probably more reliable than other studies. Bremer et al. [78] dated the separation of Scrophulariaceae from the Plantaginaceae at 76 Mya (normal calibration density; mean = 76 Ma, s.d. = 2Ma). We applied an uncorrelated lognormal relaxed molecular clock using a GTR+G substitution model for the *ITS* data set. The resulting mean substitution rates was 0.13% per Ma (95% CI = 0.09–0.16% per Ma). We defined the biogeographic areas as East Asia, Europe and North America. Root ages of this taxus were 28.56 Ma (95% HPD: 28.36–28.75 Ma) for our calibration and 28.56 Ma (95% HPD: 13.97–46.5 Ma) for the published divergence times.

***Thuja* (Cupressaceae, Pinales)**

Nucleotide sequences of five cpDNA regions (5170 bp total), three nuclear genes (6040 bp total) were employed to reconstruct the phylogeny of *Thuja* by Peng et al. [79]. The cpDNA data set included *rpl16*, *AtpI-rpoC1*, *trnS-trnM*, *trnS-trnG* and *trnT-trnF*. The nuclear genes data set included *ITS*, *LEAFY* and *4CL*. The age of the most recent common ancestor of *Thuja* was fixed at 60 Mya based on the warliest

reliable fossil record of the genus from the middle Paleocene of Ellesmere Island in the Canadian Arctic [80–82]. To calibrate the phylogeny we used a normal prior distribution with a mean of 60 Ma and a stdev of 0.5 Ma. We applied an uncorrelated lognormal relaxed molecular clock using a GTR+G substitution model for the cpDNA data set and a HKY+I substitution model for the nuclear gene data set. The resulting mean substitution rates was 0.018% per Ma (95% CI = 0.015–0.025% per Ma). We defined the biogeographic areas as East Asia and North America. Root ages of this taxus were 59.99 Ma (95% HPD: 59.02–60.99 Ma) for our calibration and 60 Ma for the published divergence times.

***Hamamelis* (Hamamelidaceae, Saxifragales)**

The evolution of genus *Hamamelis* was re-analysed through phylogenetic analyses [83]. Phylogenetic relationships of all *Hamamelis* species were reconstructed using sequence data from six plastid (4961 bp total) and two nuclear DNA regions (1053 bp total). The plastid data set included *trnL-F*, *psaA-ycf3*, *rps16*, *matK*, *atpB-rbcL*, and *psbA-trnH*. The nuclear region data set included *ITS* and *ETS*. The stem age of Altingiaceae was assigned at 90 Ma, based on fossil inflorescences, fruits and pollen of *Microaltingia* [84]. The divergence of the clade (*Liquidambar acalycina*, *L. formosana*) was constrained at 15.6 Ma based on the fossil *L. changii* [85,86]. Within Hamamelidaceae, the stem *Corylopsis* was assigned at 50 Ma based on fossil leaves of *Corylopsis reedae* in the lower Eocene [87]. We also used the earliest known fossil *Fothergilla malloryi* in the lower Eocene [87] as a calibration point (the age is 50 Ma). These constraining ages were set to be normally distributed with a standard deviation (s.d. = 2). We applied an uncorrelated lognormal relaxed molecular clock using a GTR+G substitution model for the plastid data set, and a GTR+I+G substitution model for the nuclear region data set. The mean substitution rate was 0.049% per Ma (95% CI = 0.042–0.056% per Ma). We defined the biogeographic areas as East Asia and North America. Root ages of this taxus were 23.07 Ma (95% HPD: 8.71–42.88 Ma) for our calibration and 10.6 Ma (95% HPD: 4.2–19.6 Ma) for the published divergence times.

***Circaea* (Onagraceae, Myrtales)**

The phylogeny of *Circaea* published by Xie et al. [88] was re-analysed using three plastid markers (3545 bp total) and nr*ITS* sequences (648 bp total). The plastid data set included *petB-petD*, *rpl16*, and *trnL-F*. To calibrate the phylogeny, we followed Berry et al. [89] and constrained the *Circaea-Fuchsia* node with the age of 41.5 Ma (the average age from different fossil constrainrts). The root such as the divergence time of *Hauya* and the [*Circaea* – *Fuchsia*] clade was constrained to be 52.6 Ma estimated by Berry et al. [89]. The time estimate of the nodes was regarded as minimum ages. We applied an uncorrelated lognormal relaxed molecular clock using a GTR+I+G substitution model for the plastid data set, and a HKY substitution model for the nr*ITS* data set. The resulting mean substitution rate was 0.039% per Ma (95% CI = 0.031–0.048% per Ma). We defined the biogeographic areas as Eastern Palearctic, Western Palearctic, eastern North America, western North America, South America, Central America and the South Pacific area. Root ages of this taxus were 23.77 Ma (95% HPD: 16.42–31.13 Ma) for our calibration and 16.17 Ma (95% HPD: 7.69–24.53 Ma) for the published divergence times.

***Zizania* (Poaceae, Cyperales)**

The phylogeny of *Zizania* published by Xu et al. [90] was re-constructed using sequences of seven DNA fragments (5597 total) from chloroplast, mitochondrial and nuclear genomes. The data set included *atpB-rbcL*, *matK*, *rps16*, *trnL-F*, *trnH-psbA*, *nad1*, and *Adh1a*. Two calibration points were used to estimate the divergence time within *Zizania*: The minimum age for the stem node of Oryzeae was 34.5 Ma (normal calibration density; s.d. = 6.8 Ma) obtained from Vicenti et al. [91]. And the minimum age for the stem node of *Leersia* was 7 Ma (normal calibration density; s.d. = 2 Ma), which was the spikelets found in a Miocene excavation in Germany [92]. We applied an uncorrelated lognormal relaxed molecular clock using a HKY substitution model for the whole data set. The resulting mean substitution rate was 0.26% per Ma (95% CI = 0.15–0.38% per Ma). We defined the biogeographic areas as Asia, North

America and South America. Root ages of this taxus were 3.85 Ma (95% HPD: 2.19–6.2 Ma) for our calibration and 3.74 Ma (95% HPD: 1.04–7.23 Ma) for the published divergence times.

***Toxicodendron* (Anacardiaceae, Sapindales)**

The phylogeny of *Toxicodendron* published by Nie et al. [93] was re-constructed using sequences of two chloroplast DNA fragments and three nuclear DNA fragments. The data set included 3024 bp for cpDNA and 1808 bp for nrDNA. The cpDNA data set contained fragments of *ndhF* and *trnL-F*. The nrDNA data set contained fragments of *ITS*, *ETS*, and *NIA-i3*. Wood and pollen fossils of Anacardiaceae have been dated to the Paleocene [94,95]. Thus, a normally distributed calibration prior with the mean 60 Ma and standard deviation 3 Ma was constrained for the root age of the family. Reliable macro-fossils of *Rhus* were found from western North America in the middle Eocene [96]. The minimum stem age of *Rhus* was thus calibrated to be 44 Ma with standard deviation of 1 Ma. We applied an uncorrelated lognormal relaxed molecular clock using a GTR+G substitution model from both of the chloroplast and nuclear data set. The mean substitution rate was 0.11% per Ma (95% CI = 0.092–0.13% per Ma). We defined the biogeographic areas as Asia, North America, South America and Africa. Root age of this taxus was 32.30 Ma (95% HPD: 23.39–42.39 Ma) for our calibration and there was no exact data of the root age for the published divergence times.

***Phryma* (Phrymaceae, Tubiflorae)**

The phylogeny of *Phryma* published by Nie et al. [97] was re-analysed using sequences of nuclear ribosomal *ITS* and chloroplast *rps16* and *trnL-F*. The data set included 614 bp for *ITS*, 844 bp for *rps16*, and 889 bp for *trnL-F*. To allow multiple fossil calibrations in a broad phylogenetic framework, sequences of some additional taxa were obtained in this study. To calibrate the phylogeny, we used three calibration points between outgroups in this study: The divergence time of *Verbena*, *Stachytarpheta* and *Jacaranda*, *Chilopsis*, *Macrocatalpa*, *Catalpa*, *Campsis*, *Tecoma*

was constrained to be 49.4 Ma (normal calibration density; s.d. = 2 Ma). The divergence time of *Chilopsis* and *Macrocatalpa*, *Catalpa* was constrained to be 35 Ma (normal calibration density; s.d. = 1 Ma). The divergence time of *Fraxinus* and *Osmanthus* and *Olea* was constrained to be 37 Ma (normal calibration density; s.d. = 1 Ma). We applied an uncorrelated lognormal relaxed molecular clock using HKY substitution model for *ITS*, *rps16* and *trnL-F*, respectively. The mean substitution rate was 0.35% per Ma (95% CI = 0.18–0.54% per Ma). We defined the biogeographic areas as Asia and North America. Root ages of this taxus were 3.733 Ma (95% HPD: 2.72–4.73 Ma) for our calibration and 3.68±2.46 Ma for the published divergence times.

Mitchelleae (Rubiaceae, Rubiales)

The phylogeny of *Mitchella* and its close relative *Damnacanthus* published by Huang et al. [98] was re-analysed using sequences of the nuclear internal transcribed spacer (*ITS*; 898 bp) and three plastid markers (*atpB*, 1091 bp; *rbcL*, 1409 bp; *trnL-F*, 1284 bp). To allow multiple fossil calibrations in a broad phylogenetic framework of Rubiaceae, sequences of 63 additional taxa were obtained in this study. We followed Antonelli et al. [99] in using the oldest fossil of this genus to place a minimum age constraint of 33.9 Ma (lognormal calibration density), which was fixed by using the ending point of the geological epoch to which the fossil belongs as the stem age of *Cephalanthus*. The pollen fossil of *Faramea* have been reported from the Late Eocene [100]; we thus used 37 Ma (lognormal calibration density) to set a minimum age of the *Faramea* stem node. Saenger [101] reported pollen fossil age of *Scyphiphora*, accordingly we used 23 Ma (lognormal calibration density) as a minimum age prior for the *Scyphiphora* stem node. According to the phylogenetic analysis of *Morinda* [102] and the well preserved fossil *Morinda chinensis* dated to the late Early to the early Late Eocene [103], we calibrated the stem age of *Morinda* with the prior of 44.5 Ma (normal calibration density; s.d. = 3 Ma). To root the tree, 78 Ma was enforced as the divergence time between Rubiaceae and other Gentianales, based on Bremer et al. [78], who used a broad sampling of asterids and multiple fossils. We applied an

uncorrelated lognormal relaxed molecular clock using a GTR+I+G substitution model for the *ITS* data set, a HKY+G substitution model for the *atpB* data set, a GTR+I+G substitution model for the *rbcL* data set and a GTR+G substitution model for the *trnL-F* data set. The resulting mean substitution rate was 0.12% per Ma (95% CI = 0.11–0.16% per Ma). We defined the biogeographic areas as East Asia and North America. Root age of this taxus was 60.24 Ma (95% HPD: 50.21–70.54 Ma) for our calibration and there was no exact data of the root age for the published divergence times.

***Calycanthus* (Calycanthaceae, Ranales)**

The phylogeny of Calycanthaceae published by Zhou et al. [104] was re-analysed using *rbcL* sequences. The data set included 1321 bp for *rbcL* gene. Calibrations for the divergence time estimation were as follows: (1) the root of Laurales was constrained to maximally 140 Ma, based on the onset of angiosperm radiation [105] [106]. We thus used 140 Ma (normal calibration density; s.d. = 0.5 Ma) to set the age of the root node. (2) The split between Atherospermataceae and Gomortegaceae was constrained to 88–86 Ma based on the earliest pollen of Atherospermataceae [107]. We thus used 87 Ma (normal calibration density; s.d. = 1 Ma) to set the age of this node. (3) The split between neotropical *Siparuna* and African *Glossocalyx* was constrained to 90–88 Ma, based on molecular-clock estimates for Laurales using a larger sample of family representatives [108]. We thus used 89 Ma (normal calibration density; s.d. = 1 Ma) to set the age of this node. (4) The divergence of *Laureliopsis* was constrained to minimally 83 Ma, based on the oldest *Laureliopsis* wood [109]. We thus used 83 Ma (normal calibration density; s.d. = 0.6 Ma) to set the age of this node. We applied an uncorrelated lognormal relaxed molecular clock using a GTR+I+G substitution model for the *rbcL* data set. The resulting mean substitution rate was 0.02% per Ma (95% CI = 0.015–0.026% per Ma). We defined the biogeographic areas as East Asia and North America. Root ages of this taxus were 13.04 Ma (95% HPD: 1.71 – 36.02 Ma) for our calibration and 16.0 Ma for the published divergence times.

***Lathyrus* (Leguminosae, Rosales)**

The phylogeny of *Lathyrus* published by Kenicer et al. [110] was re-analysed using *trnL-F*, *trnS-G* and *ITS* sequences. The data set included 660 bp for *trnL-F*, 839 bp for *trnS-G*, and 595 bp for *ITS* data set. To calibrate the phylogeny, we used the origin time of the Fabeae (17.5 Ma; normal calibration density; mean = 17.5 Ma, s.d. = 1.5 Ma) as root age. We applied an uncorrelated lognormal relaxed molecular clock using a GTR+G substitution model for the *trnL-F* data set, a GTR+G substitution model for the *trnS-G* data set, and a GTR+I+G substitution model for the *ITS* data set. Mean substitution were allowed to vary. The resulting mean substitution rates was 0.72% per Ma (95% CI = 0.6–0.85% per Ma). We defined the biogeographic areas as Asia, Europe, North America, South America and Africa. Root ages of this taxus were 6.12 Ma (95% HPD: 5.55–6.7 Ma) for our calibration and 5.4–6.3 Ma for the published divergence times.

***Taxus* (Taxaceae, Taxales)**

The phylogeny of *Taxus* published by Li et al. [111] was re-analysed using the nuclear ribosomal DNA *ITS* region (1169 bp total). The fossil record of *Taxus* and *Pseudotaxus* allowed them to calibrate a base substitution rate. Fossils of *Taxus* date back to the middle Jurassic, and *Pseudotaxus* has been recorded from the late Cretaceous [112]. We presume that the two lineages diverged at least by the middle Jurrassic (165 Ma). We thus used 165 Ma (normal calibration density; s.d. = 10 Ma) to set the age of the root node of phylogeny. We applied an uncorrelated lognormal relaxed molecular clock using a HKY substitution nodel for the *ITS* data aet. The mean substitution rates was 0.43% per Ma (95% CI = 0.21–0.66% per Ma). We defined the biogeographic areas as Asia, Europe and North America. Root ages of this taxus were 11.12 Ma for our calibration and 9.2–16.15 Ma for the published divergence times.

***Thuja* (Cupressaceae, Pinales)**

The phylogeny of *Thuja* L. published by Li et al. [113] was re-analysed using

sequences of nuclear ribosomal DNA internal transcribed spacer (*ITS*) region. The data set included 1143 bp. We used a Plio/Pleistocene fossil age (1.8 Ma; normal calibration density; s.d. = 1 Ma) to calibrate the divergence of *T. occidentalis* [81]. We applied an uncorrelated lognormal relaxed molecular clock using a HKY+G substitution model for the *ITS* data set. The resulting mean substitution rate was 0.25% per Ma (95% CI = 0.11-0.46% per Ma). We defined the biogeographic areas as East Asia, eastern North America and western North America. Root ages of this taxus were 21 Ma (95% HPD: 6.67–39.85 Ma) for our calibration and 25.6±13.1 Ma for the published divergence times.

***Rhus* (Anacardiaceae, Sapindales)**

The phylogeny of *Rhus* published by Yi et al. [114] was re-analyzed using nuclear *ITS* sequence and two chloroplast regions (*ndhF* and *trnL-F*). The data set included 734 bp for *ITS*, 2126 bp for *ndhF*, and 950 bp for *trnL-F*. Based on the fossil of *Rhus* and its closed relatives, we can constrain the ages of three nodes in the phylogeny of the *Rhus* complex. First, we used the age of the earliest divergence of the Rhoeae (60 Ma; lognormal calibration density; offset = 60 Ma, mean = 0.3, s.d. = 0.7) because fossils of tribe Rhoeae date back to Paleocene [115]. Second, we constrained the crown clade of *Rhus* subgenus *Lobadium* as 28 Ma (normal calibration density; s.d. = 2 Ma) because the oldest fossil for *Rhus* subgenus *Lobaduim* was reported during the Late Oligocene [116]. And finally, we constrained the minimum age of the crown group of *Pistacia* to be 30 Ma (lognormal calibration density; offset = 60 Ma, mean = 0.20, s.d. = 0.6) because the oldest fossil of *Pistacia* was from southern France during Oligocene [117]. We applied an uncorrelated lognormal relaxed molecular clock using a GTR+I+G substitution model for the *ITS* data set, a GTR+I+G substitution model for the ndhF data set, and a GTR+I+G substitution model for the *trnL-F* data set. The mean substitution rates was 0.037% per Ma (95% CI = 0.032–0.043% per Ma). We defined the biogeographic areas as Asia, North America and Africa. Root ages of this taxus were 34.44 Ma (95% HPD: 27.52–41.29 Ma) for our calibration and 38.1±3.0 Ma for the published divergence times.

Pseudotsuga **(Pinaceae, Pinales)**

The phylogeny of genus *Pseudotsuga* published by Wei et al. [118] was re-analyzed using seven plastid regions and one nuclear gene (*LEAFY*; 2152 bp total). The plastid data set (9435 bp total) included *atpB-rbcL*, *trnT-trnF*, *trnC-trnD*, *petG-psaJ*, *trnfM-trnS*, *cox1* and *nad5* intron. To calibrate the phylogeny, we used earliest known fossil records for *Pseudotsuga* which were found in the early Oligocene lowland Willamette and Rujada paleofloras of central Oregon [119]. We fixed the age of root for *Pseudotsuga* to 32 Ma (normal calibration density; s.d. = 1 Ma). We applied an uncorrelated lognormal relaxed molecular clock using a HKY substitution model for the *LEAFY* data set, and a GTR+I substitution model for the whole plastid regions data set. The resulting substitution rate was 0.14% per Ma (95% CI = 0.054–0.22% per Ma). We defined the biogeographic areas as East Asia and North America. Root ages of this taxus were 31.9 Ma (95% HPD: 29.87–33.79 Ma) for our calibration and 32 Ma for the published divergence times.

Orontioideae (Alismatales, Dicotyledons)

The phylogeny of the subfamily Orontioideae (*Symplocarpus*, *Lysichiton*, and *Orontium*) of Araceae published by Nie et al. [120] was re-analyzed using *trnL-F* and *ndhF* sequences. The data set included 3150 bp total. We focused on the proto Araceae, especially on Orontioideae, which had a reliable fossil infructescence showing similarities to, but distincted from *Symplocarpus* from the late Cretaceous of Canada [121]. To calibrate the phylogeny, we used a minimal age of 72 Ma (normal calibration density; s.d. = 5) based on fossils described as *Albertarum pueri* and assigned to Orontioideae. The root of Araceae was constrained to 120 Ma (normal calibration point; s.d. = 5) based on the fossil species *Mayoa portugallica*, a highly characteristic aperturate, striate fossil pollen described from the Early Cretaceous (110–120 Ma) of Torres Vedras in the Western Portuguese Basin [122]. We applied an uncorrelated lognormal relaxed molecular clock using a HKY+G substitution model for the *trnL-F* and *ndhF* data set. The resulting substitution rate was 0.09% per Ma

(95% CI = 0.076–0.1% per Ma). We defined the biogeographic areas as East Asia and North America. Root ages of this taxus were 39.6 Ma (95% HPD: 25.2–54.54 Ma) for our calibration and 75.31 ± 2.95 Ma for the published divergence times.

***Kelloggia* (Rubiaceae, Rubiales)**

The phylogeny of *Kelloggia* and its relatives published by Nie et al. was re-analyzed using sequences of three chloroplast DNA regions (1333 bp total; *rbcl* gene, *atpB-rbcL* spacer, and *rps16* intron). The fossil record of Rubiaceae is rather pooly known, with only a few unequivocal records in the Northern Hemisphere [96,123]. *Emmenopterys* Oliver is the earliest record of Rubiaceae fruits from the middle Eocene [96,124]. The fossil seed *Pinckneya* is known from the Oligocene [125]. Pollen fossils of *Faramea* are known from Late Eocene to recent [126]. Based on the available fossil data, nodes of the *Faramea–Coussarea* clade and the *Emmenopterys–Pinckneya* clade were constrained with ages of 40 (normal calibration density; s.d. = 1) and 45 Ma (normal calibration density; s.d. = 1), respectively. Because of the first record of Rubiaceae fossils from the Early Eocene [127], the root of Rubiaceae was set to be 54 Ma (normal calibration density; s.d. = 1). We applied an uncorrelated lognormal relaxed molecular clock using a GTR+I+G substitution model for the chloroplast data set. The resulting substitution rate was 0.059% per Ma (95% CI = 0.049–0.07% per Ma). We defined the biogeographic areas as East Asia and North America. Root ages of this taxus were 7.03 Ma (95% HPD: 1.17–18.38 Ma) for our calibration and 5.42 ± 2.32 Ma for the published divergence times.

***Aesculus* (Sapindaceae, Sapindales)**

The phylogeny of *Aesculus* published by Harris et al. [128] was re-analyzed using chloroplast DNA regions, *ITS* and part of the *LEAFY* gene. The data set included 823 bp for *ITS* and 811 bp for *LEAFY* gene. The chloroplast data set included *rps16*, *trnH-K* and *matK* (4506 bp total). To calibrate the phylogeny, we used the fossil *Aesculus hickeyi* [129] from the Paleocene-Eocene boundaryto set the age of the root (lognormal calibration density; offset = 58 Ma, mean = 0.5, s.d. = 3.0). We applied an

uncorrelated lognormal relaxed molecular clock using a HKY substitution model for the *ITS* data set, a HKY+G substitution model for the *LEAFY* data set, and a HKY substitution model for *rps16*, *trnH-K* and *matK* data set. The resulting substitution rate was 0.092% per Ma (95% CI = 0.072–0.11% per Ma). We defined the biogeographic areas as Europe, East Asia, western North America, eastern North America and South America. Root ages of this taxus were 61 Ma (95% HPD: 58.14–67.48 Ma) for our calibration and 62.36 Ma (95% HPD: 58.12–72.81 Ma) for the published divergence times.

Sect. *Quinquefoliae* (*Pinus*, Pinaceae, Pinales)

The phylogeny of sect. *Quinquefoliae* (*Pinus*) published by Hao et al. [130] was re-analyzed using four cpDNA fragments (*matK*, *trnG*, *ycf1* and *trnC-D*) and a single-copy nuclear gene (*LEAFY*). The data set included 13463 bpfor chloroplast fragments and 3425 bp for nuclear gene. Two fossil calibrations were used to constrain (1) the crown age of *Pinus*: The earliest fossil can be reliably attributed to *Pinus yorkshirensis* [131] dates from the Early Cretaceous (131–129 Ma). The age of this fossil is very close to that of *P. belgica* (145–125 Ma) [132]. Thus the crown age of *Pinus* was calibrated to 130 Ma (gamma prior distribution; shape = 4.0, scale = 1.0). (2) The divergence time of two subgenera of *Pinus*: Subgenus *Strobus* can be dated to the Late Cretaceous (85.8–83.5 Ma; [55]) based on the anatomy of silicified fossil wood, which provides a minimum age for the divergence between the two subgenera of *Pinus*. Thus the divergence time of the two subgenera was set to 85 Ma (gamma prior distribution; shape = 4.0, scale = 1.0). We applied an uncorrelated lognormal relaxed molecular clock using a GTR+G substitution model for the nuclear data set, and a GTR+I+G substitution model for the chloroplast data set. The resulting substitution rate was 0.067% per Ma (95% CI = 0.054–0.08% per Ma). We defined the biogeographic areas as East Asia, North Asia, western North America, eastern North America and Europe. Root ages of this taxus were 58.75 Ma (95% HPD: 44.47–72.55 Ma) for our calibration and 48.25 Ma (95% HPD: 33.22–64.04 Ma) for the published divergence times.

***Juniperus* (Cupressaceae, Pinales)**

The phylogeny of *Juniperus* published by Mao et al. [133] was re-analyzed using nine chloroplast DNA regions (*matK*, *rbcL*, *trnL-F*, *rps4*, *trnS-G*, *trnD-T*, *trnV*, *petB-D* and *psbB1-B2*). The data had a 9980 bp. The earliest calibration point used was the stem lineage of *Chamaecyparis*, which was set at 99.6 Ma (normal calibration density; s.d. = 2), given that seed cone or foliage remnants comparable to *Chamaecyparis* occur frequently from this point onwards [82,134]. The second calibration point used was the crown lineage of *Chamaecyparis,* which was set at 83.5 Ma (normal calibration density; s.d. = 2.0) based on the fossil record of *Chamaecyparis corpulenta* [135] which indicate diversification within *Chamaecyparis* from this point onwards. Within *Juniperus*, we constrained the divergence of sect. *Sabina* (stem lineage) from the other two sections to a minimum age of 33.9 Ma (lognormal calibration density; mean = 1.0, s.d. = 0.5) based on the fossil of *J. pauli* [136]. We constrained the crown lineage of the serrate-leaved series to a minimum age of 23 Ma (lognormal calibration density; mean = 1.0, s.d. = 0.5) based on the fossil of *J. creedensis* [137]. We constrained the divergence of the most recent common ancestor of the species pair *J. occidentalis* and *J. osteosperma* from its sister lineage to a minimum age of 16 Ma (lognormal calibration density; mean = 1.0, s.d. = 0.5) based on the fossil of *J. desatoyana* [138]. Three further minimum age calibration points outside of *Juniperus* were included: First, the split of *Calocedrus* from its sister lineage (i.e., the stem lineage of *Calocedrus*) was constrained to 28.4 Ma (lognormal calibration density; mean = 1, s.d. = 0.6) based on the fossil *Calocedrus suleticensis* [139]. Second, the split of *Tetraclinis* from its sister lineage (i.e., the stem lineage of *Tetraclinis*) was constrained to 23.0 Ma (lognormal calibration density; mean = 1.5, s.d. = 0.6) based on the fossil *Tetraclinis salicornioides* [140]. Third, the split of *Fokienia* from the MRCA of *Chamaecyparis obtusa* and *C. lawsonia* (i.e., the stem lineage of *Fokienia*) was constrained to 61.7 Ma (lognormal calibration density; mean = 0.5, s.d. = 0.5) based on the fossil *Fokienia ravenscragensis* [141,142]. We applied an uncorrelated lognormal relaxed

molecular clock using a GTR+I+G substitution model for the chloroplast data set. The resulting substitution rate was 0.031% per Ma (95% CI = 0.027–0.036% per Ma). We defined the biogeographic areas as Europe, East Asia, North America and Africa. Root ages of this taxus were 42.16 Ma (95% HPD: 33.94–74.86 Ma) for our calibration and 43.66 Ma (95% HPD: 34.09–52.87 Ma) for the published divergence times.

***Abies* (Pinaceae, Pinales)**

The phylogeny of *Abies* published by Xiang et al. [143]was re-analyzed using DNA data from three genes, i.e., internal transcribed spacer *ITS* (1700 bp total), three chloroplast DNA intergenic spacers (2888 bp) and two mitochondrial intergenic spacers (830 bp). The chloroplast data set included *rpl16*, *rps12-rpl20* and *trn T-F*. The mitochondrial data set included *ssu* rRNA and *nad5* intron 1. The macrofossil record of *Abies miller*i, known from the early Middle Eocene (47 Ma; [144]), was used to constrained the minimum age of the root of *Abies* using a lognormal prior distribution (offset = 46 Ma, mean = 1.4, s.d. = 1.4). The fossil of *A. concoloroides* [145] was used to constrain the minimum age of the crown node of the clade including sects. *Grandes* and *Oiamel* with a uniform prior distribution of 47–16 Ma. The fossil of *A. aburaensis* [145] was assigned to the crown node of sect. *Momi* using a lognormal prior distribution (offset = 16 Ma, mean = 0.5, s.d. = 1.2). We applied an uncorrelated lognormal relaxed molecular clock using a HKY+G substitution model for the *ITS* data set, a GTR+G substitution model for the chloroplast data set, and a GTR substitution model for the mitochondrial data set. The resulting substitution rate was 0.032% per Ma (95% CI = 0.025-0.038% per Ma). We defined the biogeographic areas as East Asia, Europe and western North America. Root ages of this taxus were 43.26 Ma (95% HPD: 32.84–54.13 Ma) for our calibration and 48.6 Ma (95% HPD: 33.7–73.4 Ma) for the published divergence times.

***Peracarpeae* (Campanulaceae, Campanulales)**

The phylogeny of *Peracarpeae* published by Zhou et al. [146] was re-analyzed using four plastid markers (5445 bp total; *matK*, *atpB*, *rbcL* and *trnL-F*). The sole fossil

record of Campanulaceae are *Campanula* seeds from the Miocene from southern Poland [147,148]. Considering the certainty of this fossil taxon in the *Rapunculus* clade but the uncertainty concerning the specific affinity with extant species, we placed it as a minimum age constraint for the crown age of the *Rapunculus* clade (lognormal calibration density; offset = 16.1 Ma, mean = 1.0, s.d. = 1.0). The divergence time between *Lobelia* and the Campanulaceae *sensu stricto* [149] was estimated at 56–49 Ma based on the broad dating analyses of angiosperm families by Wikström et al. [150] (normal calibration density; mean = 52.2 Ma, s.d. = 4.5) as our prior for the root node. We applied an uncorrelated lognormal relaxed molecular clock using a GTR+I+G substitution model for the plastid data set. The resulting substitution rate was 0.17% per Ma (95% CI = 0.14–0.2% per Ma). We defined the biogeographic areas as East Asia, western Eurasia and North America. Root ages of this taxus were 21.55 Ma (95% HPD: 18.17–26.32 Ma) for our calibration and 21.54 Ma (95% HPD: 18.19–28.02 Ma) for the published divergence times.

***Calocedrus* (Cupressaceae, Pinales)**

The phylogeny of *Calocedrus* published by Chen et al. [151] was re-analyzed using nuclear *ITS* sequences (1123 bp total). The root of *Calocedrus* was constrained between 33.7 and 23.8 Ma (normal calibration density; mean = 28.75 Ma, s.d. = 4.95) because the genus appeared at the beginning of the Oligocene and became abundant during this epoch. We applied an uncorrelated lognormal relaxed molecular clock using a HKY+G substitution model for the *ITS* data set. The resulting substitution rate was 0.044% per Ma (95% CI = 0.024–0.068% per Ma). We defined the biogeographic areas as East Asia and North America. Root ages of this taxus were 27.06 Ma (95% HPD: 21–32.95 Ma) for our calibration and 25.2 Ma for the published divergence times.

Altingiaceae (Saxifragales, Dicotyledons)

The phylogeny of Altingiaceae published by Ickert-Bond and Wen [86] was re-analyzed using five non-coding chloroplast regions (5632 bp total). The data set

included *trnL-F*, *psaA-ycf3*, *rps16*, *trnS-G*, *trnG* and *rbcL*. The stem lineage of Altingiaceae was constrained to be 90 Ma (normal calibration density; s.d. = 0.5) based on fossil inflorescences, fruits and pollen of *Microaltingia* [84]. The split between Hamamelideae and Corylopsideae was constrained to be 85 Ma (normal calibration density; s.d. = 0.5) based on the fossil flowers of *Androdeciduaendressii* [152], while the age of *Corylopsis* was constrained to 50 Ma (normal calibration density; s.d. = 0.3) based on fossil leaves of *Corylopsis reedae* [87]. Within Altingiaceae we constrained the divergence of the clade [*L. acalycina* [*L. formosana* - *A. obvata*]] from the remaining eastern Asian species to 15.6 Ma (normal calibration density; s.d. = 0.5) based on the western North American Middle Miocene *L. changii* [85]. We applied an uncorrelated lognormal relaxed molecular clock using a HKY+G substitution model for the chloroplast data set. The resulting substitution rate was 0.027% per Ma (95% CI = 0.022–0.032% per Ma). We defined the biogeographic areas as East Asia, Western Asia and North America. Beacause of more sequences we used in our study, root ages of this taxus were 57.16 Ma (95% HPD: 36.15–79.8 Ma) for our calibration and 39.12±7.37 Ma for the published divergence times.

***Rhodiola* (Crassulaceae, Saxifragales)**

The phylogeny and divergence time of *Rhodiola* published by Zhang et al. [153] was re-analyzed using *matK* and *rbcL* sequences. The data set included 734 bp for *matK* and 1221 bp for *rbcL*. The oldest *Myriophyllum*-like fossil pollen dates from the Late Eocene of the southeast United States [154]. We thus set the stem age of *Myriophyllum* to 37.7–34.75 Ma (exponential calibration density; offset = 34.7, mean = 1.0). The divergence time of Crassulaceae from Haloragaceae and its allies was estimated to be 84–69 Ma [155]. We used this time estimate to set the root age (normal calibration density; mean = 76.5, s.d. = 4.8) as the prior for the root node. We applied an uncorrelated lognormal relaxed molecular clock using a GTR+G substitution model for the *matK* data set and a HKY+I substitution model for the *rbcL* data set. The resulting substitution rate was 0.054% per Ma (95% CI = 0.041–0.067% per Ma). We defined the biogeographic areas as East Asia, Europe and North America.

Root ages of this taxus were 11.39 Ma (95% HPD: 8.89–14.52 Ma) for our calibration and 12.12 Ma (95% HPD: 6.32–20.23 Ma) for the published divergence times.

Dryopteridaceae (Polypodiales, Equisetopsida)

The phylogeny of the polystichoid ferns (Dryopteridaceae) published by Le Péchon et al. [156] was re-analyzed using five plastid loci (3275 bp total). The data set included the intergenic spaces *psbA-trnH*, *trnS-rps4*, *trnL-F*, the *trnL* intron and the protein-coding gene *rbcL*. The fossil *Elaphoglossum miocenicum* was considered as an extinct crown group representative of the genus *Elaphoglossum* [157], which was a bolbitoid fern described from the Early Miocene (20–15 Ma). We thus used this time estimate to constrain the crown group of *Elaphoglossum* (normal calibration density; mean = 17.5, s.d. = 1.5). The second fossil, *Protodrynaria takhtajanii* has been reported from the Eocene–Oligocene boundary (33.9 Ma) [158], which is considered to be closely related to the extant genus *Drynaria* within the family Polypodiaceae. We thus used this age to constrained the divergence between Polypodiaceae and Davalliaceae (normal calibration density; mean = 33.9, s.d. = 1.0). The third fossil taxon can undoubtedly be assigned to the extant genus *Woodwardia* with a first appearance in the Palaeocene, having been widespread throughout the Paleogene [51]. We thus used this age to constrained the divergence between *Blechnum* and *Woodwardia* (normal calibration density; mean = 55.8, s.d. = 1.0). We furthermore constrained the root of the phylogeny using a secondary calibration point derived from a phylogeny of leptosporangiate ferns [159], which estimated the crown group age of eupolypods between 127.7 and 117.1 Ma. We thus applied this time interval (uniform calibration density) as a conservative estimate. We applied an uncorrelated lognormal relaxed molecular clock using a GTR+I+G substitution model for the plastid data set. The resulting substitution rate was 0.097% per Ma (95% CI = 0.08–0.12% per Ma). We defined the biogeographic areas as Eurasia, southwestern Indian Ocean Islands, Hawaii, South America, North America, Australia and Africa. Because of more widely outgroup selections, root age of this taxus was 127.67 Ma (95% HPD: 125.76–129.69 Ma) for our calibration and there was not corresponding root age for

the published divergence times.

***Rana* (Ranidae, Anura)**

The phylogeny of the genus *Rana* published by Yuan et al. [160] was re-analyzed using sequences of six nuclear and three mitochondrial loci. The nuclear data set included 3860 bp from *RAG1*, *RAG2*, *BDNF*, *SLC8A3*, *TYR* and *POMC*. The mitochondrial data set included 3835 bp from *12S rRNA-16S rRNA*, *cytb* and *ND2*. The root stem age of genus *Rana* except for *Rana weiningensis* was calibrated using a previous age estimate from Bossuyt et al. [161] (normal calibration density; mean = 31.2, s.d. = 8.1). The fossil ilia of several "*Rana* cf. *Rana pipiens*" individuals from the Early Miocene of Florida (18 Ma; [162,163]) were not distinguishable from extant species within the *R. pipiens* group, so we inferred the clade's minimum age as 18 Ma (lognormal calibration density; offset = 18 Ma, mean = 2, s.d. = 1). The Hottell Ranch site fossils (15 Ma), identified as *Rana* aff. *R. clamitans* [164] is a species within the *R. catesbeiana* clade. The MRCA of the *R. catesbeiana* group was calibrated at a minimum age of 15 Ma (lognormal calibration density; offset = 15 Ma, mean = 2, s.d. = 1). We used the single articulated fossil (*R. temporaria* group) dated to the Early Miocene at 20–19 Ma, [165] to calibrate the node immediately ancestral to the MRCA of the divergence of *R. asiatica* from the *R. temporaria* group (lognormal calibration density; offset = 19 Ma, mean = 2, s.d. = 1). We applied an uncorrelated lognormal relaxed molecular clock using a GTR+I+G substitution model for the mitochondrial data set, and a HKY substitution model for the nuclear data set. The resulting substitution rate was 0.67% per Ma (95% CI = 0.52–0.81% per Ma). We defined the biogeographic areas as East Asia, east North America, Europe, Central Asia, western North America, the Mexican Plateau and the Neotropics. Root ages of this taxus were 44.59 Ma (95% HPD: 35.44–55.49 Ma) for our calibration and 47.9 Ma (95% HPD: 40.3–54.6 Ma) for the published divergence times.

***Hyla* (Hylidae, Anura)**

The phylogeny of the genus *Hyla* published by Li et al. [166] was re-analyzed using

four mitochondrial genes (*12S* and *16S rRNA* genes, *tRNA*^{Leu} and *ND1*) and one nuclear gene (POMC). The mitochondrial data set included 2140 bp and the nuclear data set included 492 bp. A Middle Miocene (16 Ma) fossil of *Acris barbouri* unearthed in North America is probably the sister group of extant *Acris* species [167]. As the genus *Acris* is in all previous phylogenies retrieved as the sister group of the genus *Pseudacris* [168–171], we set the age for the divergence of *Acris* and *Pseudacris* to 16 Ma (gamma calibration density; offset = 16.0 Ma, shape = 1.0, scale = 1.0). Fossil *H. miofloridana* was also described from Miocene Hemingfordian sediments in North America (20–16 Ma), supposedly a close relative of extant *H. gratiosa* [167].We assume based on this fossil that the age of the MRCA of the monophyletic group of *H. gratiosa*, *H. cinerea* and *H. gratiosa* is 16 Ma (gamma calibration density; offset = 16.0 Ma, shape = 1.0, scale = 1.0). Fossil *H. miocenia* from North America is dated to the Middle Miocene (16–14 Ma) and is morphologically close to the extant species *H. chrysoscelis* and *H. versicolor* [167]. As *H. avivoca* is most likely the sister taxon to *H. chrysoscelis*, we set the minimum age of the MRCA of *H. versicolor* and *H. chrysoscelis* to 14 Ma (gamma calibration density; offset = 14.0 Ma, shape = 1.0, scale = 1.0). We applied an uncorrelated lognormal relaxed molecular clock using a GTR+I+G substitution model for the mitochondrial data set. The resulting substitution rate was 0.28% per Ma (95% CI = 0.19–0.38% per Ma). We defined the biogeographic areas as North America, eastern Eurasia and western Eurasia. Root ages of this taxus were 48.07 Ma (95% HPD: 24.01–58 Ma) for our calibration and 42–30 Ma for the published divergence times.

Bufonidae (Anura, Amphibian)

The phylogenetic hypothesis of the relationships among true toads published by Pramuk et al. [172] was derived from DNA fragments from three mitochondrial (*12S rRNA*, *tRNA*^{val}, *16S rRNA*) and two nuclear (*RAG1*, *CXCR4*) genes. The mitochondrial data set included 2587 bp total; the nuclear data sets included 729 bp for *CXCR4* and 790 bp for *RAG1*. In this study, they used fourteen fossil calibrations and three divergence estimated from the literature. We chose three of the calibration nodes to

re-estimate the phylogeny: the node of oldest leptodactylid [173] (normal calibration density; mean = 87.4, s.d. = 5.5), the node of oldest bufonid [174] (lognormal calibration density; offset = 54, mean = 0.1, s.d. = 1.0), and the node of oldest pelobatid [175] (normal calibration density; mean = 45.5, s.d. = 4.1). We applied an uncorrelated lognormal relaxed molecular clock using a GTR+I+G substitution model for the *12S-16S* rRNA data set, a GTR+I+G substitution model for the *CXCR4* data set, and a HKY substitution model for the *RAG1* data set. The resulting substitution rates was 0.46% per Ma (95% CI = 0.4–0.52% per Ma). We defined the biogeographic areas as North America, Eurasia, Africa and South America. Root age of this taxus was 39.98 Ma (95% HPD: 35.02–45.61 Ma) for our calibration and there was not corresponding root age for the published divergence times.

***Natricine* (Colubridae, Serpentiformes)**

The phylogeny of *Natricine* published by Guo et al. [176] was re-analyzed using six mitochondrial gene fragments (*12S rRNA*, *cytb*, *ND1*, *ND2*, *ND4* and *CO1*) and one nuclear gene (*c-mos*). The data set included 982 bp for *12S RNA*, 1057 bp for *cytb*, 964 bp for *ND1*, 1032 bp for *ND2*, 668 bp for *ND4*, 309 bp for *CO1*, and 413 bp for *c-mos*. The oldest fossil of a stem natricine, *Natrix mlynarskii* dates from 32 to 30 Ma [177]. We thus placed this fossil at the MRCA of all Natricinae (normal calibration density; mean = 30, s.d. = 4). The oldest member of the extant genus *Natrix* is *N. saniensis* or *N. merkurensis*, both dated at 22 Ma [178]. This fossil was placed at the MRCA of *Natrix* on the tree (normal calibration density; mean = 22, s.d. = 3.6). Finally, we used the oldest member of the New World genus, *Thamnophis* [179] (normal calibration density; mean = 16, s.d. = 2.4) to calibrate the age of *Thamnophis*. We applied an uncorrelated lognormal relaxed molecular clock using a GTR+I+G substitution model for the *12S rRNA* data set, a HKY+G substitution model for the *c-mos* data set, a HKY+I+G substitution model for the *CO1* data set, a GTR+I+G substitution model for the *cytb* data set, a GTR+I+G substitution model for the *ND1* data set, a GTR+I+G substitution model for the *ND2* data set, and a HKY+I+G substitution model for the *ND4* data set. The resulting substitution rate was 0.02% per

Ma (95% CI = 0.01–0.03% per Ma). We defined the biogeographic areas as western Eurasia, eastern Eurasia, Africa and North America. Root ages of this taxus were 31.69 Ma (95% HPD: 30.68–31.46 Ma) for our calibration and 33 Ma (95% HPD: 28–40 Ma) for the published divergence times.

Rat snakes (Colubridae, Serpentiformes)

The phylogeny of rat snakes published by Burbrink and Lawson [180] was re-analyzed using four mitochondrial DNA (*cytb*, *ND1*, *ND2*, *ND4*) and one nuclear DNA (*c-mos*). The data set included 1118 bp for *cytb*, 971 bp for *ND1*, 1032 bp for *ND2*, 883 bp for *ND4*, and 570 bp for *c-mos*. The calibration dates for *Lampropeltis* ranged from 19 to 15 Ma (uniform calibration density) based on the fossil record of genus *Lampropeltis* known as early as 15 Ma [181]. The calibration dates for *Pantherophis* ranged from 20 to 16 Ma (uniform calibration density) based on the New World fossil record [181]. We set the MRCA of *Zamenis situla* and *Z. lineatus* within the range of 20–6 Ma (uniform calibration density) based on the fossils considered directly ancestral to *Z. situla* and *Z. lineatus* dated at 6 Ma and the first rat snake appearance in Europe at 20 Ma [182]. We applied an uncorrelated lognormal relaxed molecular clock using a HKY+I+G substitution model for the *cytb* data set, a GTR+I+G substitution model for the *ND1* data set, a GTR+I+G substitution model for the *ND2* data set, a GTR+I+G substitution model for the *ND4* data set, and a HKY+G substitution model for the *c-mos* data set. The resulting substitution rate was 0.53% per Ma (95% CI = 0.4–0.66% per Ma). We defined the biogeographic areas as western Eurasia, eastern Eurasia, Africa and North America. Root age of this taxus was 71.1 Ma (95% HPD: 22.78–105 Ma) for our calibration and there was not corresponding root age for the published divergence times.

Geoemydidae (Tesudines, Reptilia)

The phylogeny of the turtle family Geoemydidae published by Spinks et al. [183] was re-analyzed using the mitochondrial data set (1556 bp total). We applied an uncorrelated lognormal relaxed molecular clock using a HKY+I+G substitution model

for the mitochondrial data set. There is no fossil record for this taxon and its closer relatives; therefore, we re-analyzed the data set using a mean rate of 1.2 % per Ma (s.d. = 0.7) for the mitochondrial genes [184,185]. We defined the biogeographic areas as East Palearctic, West Palearctic, North America and South America. Root age of this taxus was 24.7 Ma (95% HPD: 14.69–40.58 Ma) for our calibration and there was not corresponding root age for the published divergence times.

***Emys* (Emydidae, Testudines)**

The phylogeny of *Emys* published by Spinks and Shaffer [186] was re-analyzed using data from seven nuclear (*HNF*, *R35*, *RAG*, *RELN*, *TB29*, *TB73*, *TGFB*) and one mitochondrial (*cytb*) gene. We found strong disagreement between mitochondrial and nuclear gene trees. The nuclear genes increased the phylogenetic resolution, therefore we used exclusively the nuclear data set in this study. The data set included 767 bp for *HNF*, 976 bp for *R35*, 788 bp for *RAG*, 1104 bp for *RELN*, 590 bp for *TB29*, 667 bp for *TB73*, and 937 bp for *TGFB*. We used the fossil calibrations and estimated divergence times reported by Near et al. [187]. We applied an uncorrelated lognormal relaxed molecular clock using a HKY+G substitution model for each of the nuclear data set. The resulting mean substitution rate was 0.085% per Ma (95% CI = 0.067–0.1% per Ma). We defined the biogeographic areas as western North America, eastern North America and Eurasia. Root ages of this taxus were 12.86 Ma (95% HPD: 6.97–17.13 Ma) for our calibration and 17.2 Ma (95% HPD: 12.5–23.3 Ma) for the published divergence times.

Elapoidea (Serpentiformes, Reptilia)

The phylogeny of the snake superfamily Elapoidea published by Kelly et al. [188] was re-analyzed using four mitochondrial (*cytb*, *ND1*, *ND2*, *ND4*) and one nuclear gene (*c-mos*) data set. The data sets were analyzed singly and combination. The result showed that the combined data set increased the phylogenetic resolution. We thus used the combined data set (4601 bp total) in this study. To calibrate the phylogeny, we used the common ancestor of Asian cobras of the genus *Naja* and their closest

relatives (16 Ma; normal calibration density; mean = 15.7, s.d. = 1.0) as calibration point because fossil evidence suggests an age of 16 Ma for the divergence between Asian *Naja* and their closest relatives [189]. According to the study of Sanders et al. [190], we set the age of the MRCAs of Acrochordidae and Colubroidea (uniform calibration density; upper = 48, lower = 35) and of Scolecophidia and Alethinophidia (uniform calibration density; upper = 120, lower = 92). We applied an uncorrelated lognormal relaxed molecular clock using a GTR+I+G substitution model for the combined data set. The resulting substitution rate was 1.45% per Ma (95% CI = 1.24–1.67% per Ma). We defined the biogeographic areas as Africa, Eurasia, Australia and America. Root ages of this taxus were 42.18 Ma (95% HPD: 35.77–48.76 Ma) for our calibration and 41.3 Ma (95% HPD: 35.7–46.3 Ma) for the published divergence times.

***Plestiodon* (Scincidae, Lacertiformes)**

The phylogeny of *Plestiodon* lizards published by Brandley et al. [191] was re-analyzed using eight independently evolving loci: *BNDF* (645 bp), *MKL1* (903 bp), *ND1* (1208 bp), *PRLR* (570 bp), *PTGER4* (468 bp), *R35* (663 bp), *RAG1* (2727 bp), and *SNCAIP* (483 bp). The age of crown Episquamata (*Anniella*, *Aspidoscelis*, *Basiliscus*, and *Bipes*) was calibrated using the age of the earliest stem "anguimorph" fossils [192](lognormal calibration density; offset = 148 Ma, mean = 0.0, s.d. = 1.769).The age of the divergence between Amphisbaenia (*Bipes biporus*) and Teiidae (*Aspidoscelis*) was calibrated using the ageof the earliest teiioid (Polyglyphanodontidae) fossils (e.g., *Bicuspidon* from the Albian–Cenomanian boundary [192,193] (lognormal calibration density; offset = 96, mean = 0.0, s.d. = 2.016). We applied an uncorrelated lognormal relaxed molecular clock using a GTR+I+G substitution model for the *BNDF* data set, a GTR+I+G substitution model for the *MKL1* data set, a GTR+I+G substitution model for the *ND1* data set, a HKY+G substitution model for the *PRLR* data set, a HKY+I+G substitution model for the *PTGER4* data set, a HKY+G substitution model for the *R35* data set, a GTR+I+G substitution model for the *RAG1* data set, and a HKY+I+G substitution model for the

SNCAIP data set. The resulting substitution rate was 0.08% per Ma (95% CI = 0.064–0.098% per Ma). We defined the biogeographic areas as Asia and North America. Root ages of this taxus were 33.1 Ma (95% HPD: 23.89–45.15 Ma) for our calibration and 23.6 Ma (95% HPD: 18–29.6 Ma) for the published divergence times.

Viperidae (Squamata, Reptilia)

The phylogeny of Viperinae published by Wüster et al. [194] was re-analyzed using sequences of four mitochondrial genes (*12S*, *16S*, *NADH4* and *cytb*). The data set included 381 bp for *12S*, 388 bp for *16S*, 599 bp for *NADH4* and 641 bp for *cytb*. We used the following calibration constraints: (a) *Porthidium* (normal calibration density; mean = 3.5, s.d. = 0.51): the initial divergence of three South American populations of the genus *Porthidium*, which almost certainly invaded South America and diverged there after the uplift of the Isthmus of Panama, approximately at 3.5 Ma [195]. (b) *Eurasian vipers* (lognormal calibration density; offset = 20, mean = 1, s.d. = 1): fossil evidence suggests that the initial divergence of the Eurasian viper clade (excluding *Pseudocerastes* and *Eristicophis*) had begun by 20 Ma [189]. (c) *Naja* (lognormal calibration density; offset = 16, mean = 1, s.d. = 1): the split between the Asian *Naja* clade and its African sister clade dates back to a minimum age of 16 Ma based on the presence of characteristic apomorphies of the Asian clade in the fossil record [189,196]. (d) *Hemorrhois* (normal calibration density; mean = 18, s.d. = 2.04): the likely cladogenesis between eastern and western species occurred after Asia and Africa became joined at approximately 18–16 Ma [197]. (e) *Rattlesnakes* (lognormal calibration density; offset = 9, mean = 1, s.d. = 1): the divergence between *Crotalus* and *Sistrurus* occurred before 9 Ma based on the age of a fossil vertebra of *Sistrurus* [198]. (f) *Colubroidea* (lognormal calibration density; offset = 40, mean = 2, s.d. = 1.2): The youngest unambiguous colubroid fossils date back approximately 40 Ma to the Eocene of Asia [199]. We did not consider the putative colubroid fossils from the Cenomanian (approximately 95 Ma) described by Rage and Werner [200] and Rage et al. [201]. (g) Tree root height (lognormal calibration density; offset = 45, mean = 2.5, s.d. = 1.25): the problem of the root height of the tree ties in with the issue of the age

of the Colubroidea and Caenophidia. We applied an uncorrelated lognormal relaxed molecular clock using a GTR+I+G substitution model for the *12S rRNA* gene data set, a GTR+I+G substitution model for the *16S rRNA* gene data set, a GTR+I+G substitution model for the *NADH4* data set, and a GTR+I+G substitution model for the *cytb* data set. The resulting substitution rates was 0.95% per Ma (95% CI = 0.77-1.14% per Ma). We defined the biogeographic areas as Europe, Southeast Asia, North America, Africa, Asia and South America. Root ages of this taxus were 46.19 Ma (95% HPD: 37.55–56.05 Ma) for our calibration and 47.4 Ma (95% HPD: 38.1–57.4 Ma) for the published divergence times.

Dipodoidea (Rodentia, Mammalia)

The phylogeny of Dipodoidea published by Pisano et al. [202] was re-analyzed using five protein-coding genes (*BRCA1*, *cytb*, *GHR*, *IRBP*, *RAG1*). The data set included 500 bp for *BRCA1*, 1103 bp for *cytb*, 850 bp for *GHR*, 1075 bp for *IRBP*, and 1056 bp for *RAG1*. We assigned the oldest record of Sciuridae at 36 Ma, fossil *Douglassciurus jeffersoni* [203], to the divergence between *Aplodontia rufa* (Aplodontiidae) and the monophyletic group composed of *Sciurus aestuans* and *Marmota marmota* (Sciuridae) (lognormal calibration density; offset = 34.94, mean = 1.48, s.d. = 0.9). We assigned the oldest record of *Progonomys* [204] to the split between the early diverging tribe Phloemyini (*Batomys granti*) and the remaining tribes of Murinae (Apodemini, *Apodemus sylvaticus* and *A. mystacinus*; Rattini, *Rattus tanezumi* and *Maxomys surifer*) (lognormal calibration density; offset = 7.95, mean = 2.36, s.d. = 0.89). Fossils of *Apodemus jeanteti* (7 Ma) and *Apodemus dominans* (7 Ma) are considered to be close to extant *A. mystacinus* and *A. sylvaticus*, respectively [205]. Consequently, we assigned a minimum age of 7 Ma for the split between *A. mystacinus* and *A. sylvaticus* (lognormal calibration density; offset = 4.376, mean =2.4345, s.d. = 0.75). *Sicista primus* is the earliest known fossil attributed to the genus *Sicista* and was recovered from the 17 million-year-old deposits in Nei Mongol, China [206]. Following Zhang et al. [207], we assumed a minimum age of the radiation of modern Sminthidae of 17 Ma. Consequently, we calibrated the MRCA of Sminthidae

(lognormal calibration density; offset = 14.95, mean =2.195, s.d. = 0.75). We applied an uncorrelated lognormal relaxed molecular clock using a HKY+G substitution model for the *BRCA1* data set, a GTR+I+G substitution model for the *cytb* data set, a HKY+G substitution model for the *GHR* data set, a GTR+I+G substitution model for the *IRBP* data set, and a GTR+G substitution model for the *RAG1* data set. The resulting substitution rate was 0.21% per Ma (95% CI = 0.16–0.25% per Ma). We defined the biogeographic areas as Asia, Africa, Europe and North America. Root ages of this taxus were 50.95 Ma (95% HPD: 38.75–65.94 Ma) for our calibration and 40.62 Ma (95% HPD: 35.97–48.27 Ma) for the published divergence times.

***Mustela* (Mustelidae, Carnivora)**

The phylogeny of *Mustela* published by Harding and Smith [208] was re-analyzed using the whole 1140 bp mitochondrial cytochrome b (*cytb*) gene. Fossil calibrations used in the relaxed molecular clock approach incorporated the appearance of *Pseudobassaris* (28.5–28.0 Ma) and *Plesictis* (24.7–24.3 Ma) fossils to independently define the root and crown heights of the Mustelidae relative to Procyonidae [209–211]. We chose the older fossil appearance of *Pseudobassaris* (normal calibration density; mean = 28.5 Ma, s.d. = 1.0) to represent the root height of the tree. Mean crown height for the Mustelidae was represented by the younger appearance of *Plesictis* (normal calibration density; mean = 24.3 Ma, s.d. = 1.0). We applied an uncorrelated lognormal relaxed molecular clock using a HKY+I+G substitution model for the *cytb* data set. The resulting substitution rate was 1.39% per Ma (95% CI = 1.18–1.61% per Ma). We defined the biogeographic areas as North America, South America, East Asia, Eurasia and Europe. Root ages of this taxus were 8.84 Ma (95% HPD: 7.95–9.74 Ma) for our calibration and 7.5–9.5 Ma for the published divergence times.

***Microtus* (Circetidae, Rodentia)**

The phylogeny of the root vole (*Microtus oeconomus*) published by Brunhoff et al. [212] was re-analyzed using the whole 1143 bp mitochondrial cytochrome b (*cytb*) gene. We applied an uncorrelated lognormal relaxed molecular clock using a

HKY+I+G substitution model for the *cytb* data set. To calibration the phylogeny, we used the divergence time between the two European groups and the clade of Asian and Beringian species (lognormal calibration density; offset = 0.15, mean = 0.1, SD = 0.3). The resulting mean substitution rate was 1.78% per Ma (95% CI = 1.14–2.5% per Ma). We defined the biogeographic areas as European, Asia and Beringia. Root ages of this taxus were 0.27 (95% HPD: 0.085–0.20 Ma) Ma for our calibration and 0.19–0.66 Ma for the published divergence times.

Myotis (Vespertilionidae, Chiroptera)

The phylogeny of genus Myotis published by Ruedi and Mayer [213] was re-analyzed using *cytb* (1141 bp total) and *RAG2* (1148 bp total). We used the same model of DNA evolution (with TS/TV ratio set at 8.56 and gamma shape set at 0.24) for both genes analyses. We applied an uncorrelated lognormal relaxed molecular clock using a HKY+I substitution model for the *cytb* data set, and a HKY substitution model for the *RAG2* data set. We applied a calibration age to the root node of all species of *Myotis* (normal calibration density; mean = 12.2, SD = 2.0) and a calibration age to the divergence of *M. schaubi* and *M. nattereri* (normal calibration density; mean = 6.0, SD = 1.6). The resulting mean substitution rates were 2.64% per Ma (95% CI = 1.86–3.55% per Ma) for *cytb*, 0.1% per Ma (95% CI = 0.069–0.14% per Ma) for *RAG2*. We defined the biogeographic areas as Asia, Australia, Africa, North America and South America. Root age of this taxus was 10.67 Ma (95% HPD: 7.56–13.9 Ma) for our calibration and there was not corresponding root age for the published divergence times.

Leporidae (Lagomorpha, Mammalia)

The phylogeny of the rabbits and hares (Leporidae) published by Matthee et al. [214] was re-analyzed using five nuclear (*SPTBN1*, *PRKCI*, *THY*, *TG*, and *MGF*) and two mitochondrial (*cytb* and *12S* rRNA) gene fragments. The data set were analyzed separately and concatenated. The result showed that the combined data set increased the phylogenetic resolution among genera. We thus used the combined data set (5395

bp total) in this study. To calibrate the phylogeny, we incorporated six fossi-based time constrains from the literature (uniform calibration density). The first pair was the 40 Ma lower and 20 Ma upper divergence between the ochotonid and the leporid lineages [215]; the second was the origin of modern leporids with an upper age at 12 Ma [216] and a lower at 20 Ma [217]. The third pair involved the 6 Ma lower and 4 Ma upper divergence of the genus *Lepus* [218]. We applied an uncorrelated lognormal relaxed molecular clock using a GTR+I+G substitution model for the combined data set. The mean substitution rates was 1.24% per Ma (95% CI = 0.88-1.63% per Ma). We defined the biogeographic areas as Asia, Africa, Europe, North America and South America. Root ages of this taxus were 13.9 Ma (95% HPD: 11.71–16.16 Ma) for our calibration and 14 Ma (95% HPD: 12.08–17.48 Ma) for the published divergence times.

Melaphidina (Aphidoidea, Insecta)

The phylogeny of aphids of the tribus Melaphidina published by Ren et al. [219] was re-analyzed based on mitochondrial cytochrome c oxidase subunits I and II (*COI-COII*), leucine tRNA ($tRNA^{LEU}$), cytochrome b (*cytb*), and nuclear elongation factor 1a genes (*EF1a*). The data set included 1070 bp for *EF1a* and 2489 bp for the whole mitochondrial data set. To calibrate the phylogeny, we used two Baltic amber fossil genera assigned to Macrosiphini (Aphididae; Aphidinae) [220], the extinct *Halajaphis* and extant *Pseudamphorophora*. We thus set the common ancestor of the Macrosiphini representatives to the Early/Middle Eocene boundary (normal calibration density; mean = 48, s.d. = 2). We applied an uncorrelated lognormal relaxed molecular clock using a GTR+G substitution model for the *EF1a* data set, and a GTR+I+G substitution model for the mitochondrial data set. The resulting mean substitution rate was 0.18% per Ma (95% CI = 0.13–0.22% per Ma). We defined the biogeographic areas as Asia and North America. Root ages of this taxus were 59.18 Ma (95% HPD: 45.46–72.05 Ma) for our calibration and 46–55 Ma for the published divergence times.

***Limnogonus* (Gerridae, Hemiptera)**

The phylogeny of *Limnogonus* published by Ye et al. [221] was re-analyzed using three mitochondrial (*16S rRNA*, *COI* and *COII*) and one nuclear (*28S rRNA*) gene. The data set included 451 bp for *16S rRNA*, 514 bp for *28S rRNA* and 1210 bp for *COI-COII*. The well-preserved *Succineogerris larssoni* from the Eocene-Oligocene (54–38 Ma) Baltic amber was morphologically similar to the extant genus *Neogerris* and was identified as a close relative of *Neogerris* [222–224]. Therefore, we used a normal prior distribution (mean = 46 Ma, s.d. = 4) to constrain the crown age of the genus *Neogerris*. We applied an uncorrelated lognormal relaxed molecular clock using a GTR+I+G substitution model for the *16S rRNA* data set, a GTR+I+G substitution model for the *28S rRNA* data set, and a GTR+I+G substitution model for the *COI-COII* data set. The resulting mean substitution rate was 0.35% per Ma (95% CI = 0.23–0.5% per Ma). We defined the biogeographic areas as East Asia, Australia, Africa and South America. Root ages of this taxus were 67.02 Ma (95% HPD: 50.84–90.42 Ma) for our calibration and 49 Ma (95% HPD: 38–60 Ma) for the published divergence times.

***Aporini* (Pompilidae, Hymenoptera)**

The phylogeny of the *Aporini* published by Rodriguez et al. [225] was re-analyzed using four nuclear markers (elongation factor-1 a F2 copy (*EF1*; 729 bp total), long-wavelength rhodopsin (*LWRH*; 661 bp total), wingless (*Wg*; 389 bp total) and the D2–D3 regions of the 28S ribosomal RNA (*28S rRNA*; 908 bp total). The common ancestor of *Anoplius* and *Allochares* was given a normal prior of 25 Ma (s.d. = 10), based on a fossil of *Anoplius* sp. from the Dominican amber that belongs to the stem group of *Anoplius*. The common ancestor of *Cryptocheilus* and *Dipogon*, as well as the common ancestor of *Agenioideus* and [*Allochares* + *Anoplius*] were given a normal prior (mean = 33.0 Ma, s.d. = 0.5), based on the fossils of *Cryptocheilus hypogaeus* and *Agenioideus saxigena* found in the Colorado Florissant beds. The crown-group node of all taxa included in the analysis (family Pompilidae) was assigned a normal prior of 43 Ma (s.d. = 10), based on Wilson et al. [226]. We applied

an uncorrelated lognormal relaxed molecular clock using a GTR+I+G substitution model for the *28S rRNA*, a GTR+I+G substitution model for the *EF1*, a GTR+I+G substitution model for the *LWRH*, and a GTR+I+G substitution model for the *Wg* data set. The resulting mean substitution rate was 0.53% per Ma (95% CI = 0.36–0.71% per Ma). We defined the biogeographic areas as the Palaearctic, North America, Middle America and South America. Root ages of this taxus were 28.76 Ma (95% HPD: 20.38–41.20 Ma) for our calibration and 22.6 Ma (95% HPD: 17.4–28.83 Ma) for the published divergence times.

Papilionidae (Lepidoptera, Insecta)

The phylogeny of swallowtail butterflies (Papilionidae) published by Condamine et al. [227] was re-analyzed using DNA data from mitochondrial (*COI* and *COII*; 2189 bp total) and nuclear genes (*EF-1α*; 1164 bp total). The oldest swallowtail fossils are from the early Eocene (48 Ma; Green River formation in Colorado), and consist of two species in the genus *Praepapilio* [228]. Therefore we calibrated the Papilionidae crown group with a minimum age of 48 Ma (logmornal calibration density; mean = 1.0, s.d. = 0.5). The two other unequivocal fossils of Papilionidae are *Thaites ruminiana* from the Early Oligocene (30 Ma), and *Doritites bosniackii* from the Middle Miocene (15 Ma). We have conservatively chosen to use 30 Ma (lognormal calibration density; mean = 1.0, s.d. = 0.5) as the minimum age for the Parnassiinae crown group whereas the crown of Luehdorfiini was confined to a minimum age of 15 Ma (logmornal calibration density; mean = 1.0, s.d. = 0.5). We applied an uncorrelated lognormal relaxed molecular clock using a GTR+I+G substitution model for the mitochondrial data set, and a GTR+I+G substitution model for the *EF-1α* data set. The resulting mean substitution rate was 1.18% per Ma (95% CI = 1.05–1.33% per Ma). We defined the biogeographic areas as West Palearctic, East Palearctic, West Nearctic, East Nearctic, Africa, Madagascan, South America, Central America, India, Southeast Asia and Australasia. Root ages of this taxus were 47.81 Ma (95% HPD: 46.80–48.78 Ma) for our calibration and 49.01 Ma (95% HPD: 42.82–59.88 Ma) for the published divergence times.

Apaturinae (Nymphalidae, Lepidoptera, Insecta)

The phylogeny of Apaturinae published by Ohshma et al. [229] was re-analyzed using mitochondrial (5715 bp total) and nuclear (5575 bp total) DNA sequence data. The mitochondrial data set included the *COI*, *COII*, *ATPase8*, *ATPase6*, *COIII*, *ND3* and *ND5* genes. The nuclear data set included *EF1a*, *Wg*, *ArgK*, *CAD*, *GAPDH*, *IDH*, *MDH*, and *RpS5*. Wahlberg et al. [230] have estimated the age of Nymphalidae using seven fossil data for calibration [230]. The result indicated that Apaturinae and their sister subfamily Biblidinae diverged at 61.73 Ma. Thus, we set 61.73 Ma (normal calibration density; mean = 61.73, s.d. = 1) as the divergence time for the split between Apaturinae and *Ariadne*. We applied an uncorrelated lognormal relaxed molecular clock using a GTR+I+G substitution model for the mitochondrial data set, and a GTR+I+G substitution model for the nuclear data set. The resulting mean substitution rate was 0.4% per Ma (95% CI = 0.35–0.45% per Ma). We defined the biogeographic areas as Asia, Europe, South America, Southeast Asia, North America and Africa. Root ages of this taxus were 45.59 Ma (95% HPD: 39.21–51.57 Ma) for our calibration and 44.5 Ma (95% HPD: 42.1–46.9 Ma) for the published divergence times.

Sect. *Phalloideae* (*Amanita*, Amanitaceae, Agaricales)

The phylogeny of lethal amanitas (*Amanita* section *Phalloideae*) published by Cai et al. [231] was re-analyzed using five gene loci (*nrLSU*, *ITS*, *rpb2*, *EF1-α* and *β-tubulin*). The data set included 2392 bp. Given that fossil records of fungi are limited, it has been difficult to choose a reliable calibration point to estimate the divergence time for any fungal groups. Therefore, an extensive sampling of outgroup species for which fossils were available were selected to estimate the divergence time of *Amanita*. Two primary fossils include in this study: (a) the divergence between Ascomycota and Basidiomycota at 528 Ma, by placing *Paleopyrenomycites devonicus* in the subphylum Pezizomycotina [232]; (b) the divergence between Hymenochaetaceae and Fomitopsidaceae based on the 125 million-year-old fossil

Quatsinoporites cranhamii [233], the estimated divergence time between Ascomycota and Basidiomycota is 582–400 Ma (normal calibration density; mean = 492, s.d. = 56). We applied an uncorrelated lognormal relaxed molecular clock using a GTR+I+G substitution model for the whole data set. The resulting mean substitution rate was 0.11% per Ma (95% CI = 0.096–0.13% per Ma). We defined the biogeographic areas as North America, East Asia, Australia and Europe. Root age of this taxus was 213.04 Ma (95% HPD: 157.25–271.15 Ma) for our calibration and there was not corresponding root age for the published divergence times.

Supplementary References:

50. Li, C-X, Lu, S-G, Ma, J-Y *et al*. Phylogeographic history of the woodwardioid ferns, including species from the Himalayas. *Palaeoworld* 2016; **25**: 318–324.

51. Collinson, ME Cainozoic ferns and their distribution. *Brittonia* 2001; **53**:173–235.

52. Ran, J-H, Shen, T-T, Liu, W-J *et al*. Mitochondrial introgression and complex biogeographic history of the genus *Picea*. *Mol Phylogenet Evol* 2015; **93**: 63–76.

53. Lockwood, JD, Aleksić, JM, Zou, J *et al.* A new phylogeny for the genus *Picea* from plastid, mitochondrial, and nuclear sequences. *Mol Phylogenet Evol* 2013; **69**: 717–727.

54. Klymiuk, AA, Stockey, RA. A lower Cretaceous (Valanginian) seed cone provides the earliest fossil record for *Picea* (Pinaceae). *Am J Bot* 2012; **99**: 1069–1082.

55. Meijer, JJF. Fossil woods from the Late Cretaceous Aachen Formation. *Rev Palaeobot Palyno* 2000; **112**: 297–336.

56. Yi, T-S, Jin, G-H, Wen, J. Chloroplast capture and intra- and inter-continental biogeographic diversification in the Asian - New World disjunct plant genus *Osmorhiza* (Apiaceae). *Mol Phylogenet Evol* 2015; **85**: 10–21.

57. Banasiak, Ł, Piwczyński, M, Uliński, T *et al.* Dispersal patterns in space and time. A case study of Apiaceae subfamily Apioideae. *J Biogeogr* 2013; **40**: 1324–1335.

58. Spalik, K, Piwczyński, M, Danderson, CA *et al.* Amphitropic amphiantarctic disjunctions in Apiaceae subfamily Apioideae. *J Biogeogr* 2010; **37(10)**: 0–0.

59. Chin, S-W, Shaw, J, Haberle, R *et al*. Diversification of almonds, peaches, plums and cherries - molecular systematics and biogeographic history of *Prunus* (Rosaceae). *Mol Phylogenet Evol* 2014; **76**: 34–48.

60. Wang, H, Moore, MJ, Soltis, PS *et al.* Rosid radiation and the rapid rise of angiosperm-dominated forests. *Proc Natl Acad Sci USA* 2009; **106**: 3853–3858.

61. Romaschenko, K, Garcia-Jacas, N, Peterson, PM *et al.* Miocene-Pliocene speciation, introgression, and migration of *Patis* and *Ptilagrostis* (Poaceae. Stipeae). *Mol Phylogenet Evol* 2014;**70**: 244–259.

62. Thomasson, JR. Epidermal patterns of the lemma in some fossil and living grasses and their phylogenetic significance. *Science* 1978; **199**: 975–977.

63. Thomasson, JR. *Paleoeriocoma* (Gramineae, Stipeae) from the Miocene of Nebraska. Taxonomic and Phylogenetic Significance. *Syst Bot* 1980; **5**: 233.

64. Thomasson, JR. Miocene Fossil Grasses. Possible adaptation in reproductive bracts (lemma and palea). *Ann Missouri Bot Gard* 1985; **72**: 843.

65. González, F, Wanke, S. Present trans-Pacific disjunct distribution of *Aristolochia* subgenus *Isotrema* (Aristolochiaceae) was shaped by dispersal, vicariance and extinction. *J Biogeogr* 2014; **41**: 380–391.

66. Symmank, L, Marie-Stéphanie, S, Smith, JF *et al.* The extraordinary journey of *Peperomia* subgenus *Tildenia* (Piperaceae): Insights into diversification and colonization patterns from its cradle in Peru to the Trans-Mexican Volcanic Belt. *J Biogeogr* 2011; **38**: 2337–2349.

67. Smith, SY, Stockey, RA. Establishing a fossil record for the perianthless Piperales: *Saururus tuckerae* sp. nov. (Saururaceae) from the Middle Eocene Princeton Chert. *Am J Bot* 2007; **94**: 1642–1657.

68. Zavada, MS, Benson, JM. First fossil evidence for the primitive angiosperm family Lactoridaceae. *Am J Bot* 1987; **74**: 1590.

69. Macphail, MK, Partridge, AD, Truswell, EM. Fossil pollen records of the problematical primitive angiosperm family Lactoridaceae in Australia. *Plant Syst Evol* 1999; **214**: 199–210.

70. Zhu, W-D, Nie, Z-L, Wen, J *et al*. Molecular phylogeny and biogeography of *Astilbe* (Saxifragaceae) in Asia and eastern North America. *Bot J Linn Soc* 2013; **171**: 377–394.

71. Hermsen, EJ, Gandolfo, MA, Nixon, KC *et al*. *Divisestylus* gen. nov. (aff. Iteaceae), a fossil saxifrage from the Late Cretaceous of New Jersey, USA. *Am J Bot* 2003; **90**: 1373–1388.

72. Wehr, WC, Hopkins, DQ. The Eocene orchards and gardens of Republic, Washington. *Washington Geology* 1994; **22**: 27–34.

73. Hermsen, EJ. *The fossil record of Iteaceae and Grossulariaceae in the Cretaceous and Tertiary of the United States and Canada* (Botanical Society of America). http://ecommons.cornell.edu/bitstream/1813/1509/1/Hermsen_dissertation_final_06_23_05.pdf. (2005)

74. Emadzade, K, Hörandl, E. Northern Hemisphere origin, transoceanic dispersal, and diversification of Ranunculeae DC. (Ranunculaceae) in the Cenozoic. *J Biogeogr* 2011; **38**: 517–530.

75. Anderson, CL, Bremer, K, Friis, EM. Dating phylogenetically basal eudicots using *rbcL* sequences and multiple fossil reference points. *Am J Bot* 2005; **92**: 1737–1748.

76. Nei, M. *Molecular population genetics and evolution*. Amsterdam: North-Holland Publishing, 1975.

77. Scheunert, A, Heubl, G. Phylogenetic relationships among New World *Scrophularia* L. (Scrophulariaceae). New insights inferred from DNA sequence data. *Plant Sys Evol* 2011; **291**: 69–89.

78. Bremer, K, Friis, E, Bremer, B *et al*. Molecular phylogenetic dating of asterid flowering plants shows Early Cretaceous diversification. *Syst Biol* 2004; **53**: 496–505.

79. Peng, D, Wang X-Q. Reticulate evolution in *Thuja* inferred from multiple gene sequences: Implications for the study of biogeographical disjunction between eastern Asia and North America. *Mol Phylogenet Evol* 2008; **47**: 1190–1202.

80. McIver, EE, Basinger, JF. The morphology and relationships of *Thuja polaris* sp.nov. (Cupressaceae) from the early Tertiary, Ellesmere Island, Arctic Canada. *Can J Bot* 1989; **67**: 1903–1915.

81. Bennike, O. The Kap København Formation: stratigraphy and palaeobotany of a

Plio-Pleistocene sequence in Peary Land, North Greenland. *Meddel Grønland* 1990; 23.

82. Farjon, A. *A monograph of Cupressaceae and Sciadopitys*. Kew: Royal Botanic Gardens, 2005.
83. Xie, L, Yi, T-S, Li, R *et al*. Evolution and biogeographic diversification of the witch-hazel genus (*Hamamelis* L., Hamamelidaceae) in the Northern Hemisphere. *Mol Phylogenet Evol* 2010; **56**: 675–689.
84. Zhou, ZK, Crepet, WL, Nixon KC. The earliest fossil evidence of the Hamamelidaceae: Late Cretaceous (Turonian) inflorescences and fruits of Altingioideae. *Am J Bot* 2001; **88**: 753–766.
85. Pigg, KB, Ickert-Bond, SM, Wen, J. Anatomically preserved *Liquidambar* (Altingiaceae) from the middle Miocene of Yakima Canyon, Washington state, USA, and its biogeographic implications. *Am J Bot* 2004; **91**: 499–509.
86. Ickert-Bond, SM, Wen J. Phylogeny and biogeography of Altingiaceae: Evidence from combined analysis of five non-coding chloroplast regions. *Mol Phylogenet Evol* 2006; **39**: 512–528.
87. Radtke, MG, Pigg, KB, Wehr, WC. Fossil *Corylopsis* and *Fothergilla* leaves (Hamamelidaceae) from the Lower Eocene flora of Republic, Washington, U.S.A., and their evolutionary and biogeographic significance. *Int J Plant Sci* 2005; **166**: 347–356.
88. Xie, L, Wagner, WL, Ree, RH *et al*. Molecular phylogeny, divergence time estimates, and historical biogeography of *Circaea* (Onagraceae) in the Northern Hemisphere. *Mol Phylogenet Evol* 2009; **53**: 995–1009.
89. Berry, PE, Hahn, WJ, Sytsma, *et al*. Phylogenetic relationships and biogeography of *Fuchsia* (Onagraceae) based on noncoding nuclear and chloroplast DNA data. *Am J Bot* 2004; **91**: 601–614.
90. Xu, X, Walters, C, Antolin, MF *et al.* Phylogeny and biogeography of the eastern Asian-North American disjunct wild-rice genus (*Zizania* L., Poaceae). *Mol Phylogenet Evol* 2010; **55**: 1008–1017.
91. Vicenti, A, Barber, JC, Aliscioni, SS *et al*. The age of the grasses and clusters of origins of C 4 photosynthesis. *Global Change Bio* 2008;**14**: 2963–2977.
92. Heer, O. *Flora tertiaria helvetiae. Die tertiäre Flora der Schweiz.* Winterthur: J. Wurster & Comp, 1855.
93. Nie, Z-L, Sun, H, Meng Y *et al*. Phylogenetic analysis of *Toxicodendron* (Anacardiaceae) and its biogeographic implications on the evolution of north temperate and tropical intercontinental disjunctions. *J Syst Evol* 2009; **47**: 416–430.
94. Hsu, J. Late Cretaceous and Cenozoic vegetation in China, emphasizing their connections with North America. *Ann Missouri Bot Gard* 1983; **70**: 490.
95. Muller, J. Significance of fossil pollen for angiosperm history. *Ann Missouri Bot Gard* 1984; **71**: 419.
96. Manchester, SR. *Fruits and Seeds of the Middle Eocene Nut Beds Flora, Clarno Formation,*

Oregon New York: Paleontological Research Institution, 1994.

97. Nie, Z-L, Sun, H, Beardsley, PM *et al*. Evolution of biogeographic disjunction between eastern Asia and eastern North America in *Phryma* (Phrymaceae). *Am J Bot* 2006; **93**: 1343–1356.

98. Huang, W-P, Sun, H, Deng, T *et al.* Molecular phylogenetics and biogeography of the eastern Asian-eastern North American disjunct *Mitchella* and its close relative *Damnacanthus* (Rubiaceae, Mitchelleae). *Bot J Linn Soc* 2013; **171**: 395–412.

99. Antonelli, A, Nylander, JAA, Persson, C *et al*. Tracing the impact of the Andean uplift on Neotropical plant evolution. *Proc Natl Acad Sci USA* 2009; **106**: 9749–9754.

100. Graham, A. Fossil record of the Rubiaceae. *Ann Missouri Bot Gard* 2009; **96**: 90–108.

101. Saenger, P. Mangrove vegetation: an evolutionary perspective. *Mar Freshwater Res* 1998; **49**: 277.

102. Razafimandimbison, SG, McDowell, TD, Halford, DA *et al*. Molecular phylogenetics and generic assessment in the tribe Morindeae (Rubiaceae-Rubioideae). How to circumscribe *Morinda* L. to be monophyletic? *Mol Phylogenet Evol* 2009; **52**: 879–886.

103. Shi, X, Jin, J, Ye, C *et al*. First fruit fossil record of *Morinda* (Rubiaceae) from China. *Rev Palaeobot Palynol* 2012; **179**: 13–16.

104. Zhou, S, Renner, SS, Wen, J. Molecular phylogeny and intra- and intercontinental biogeography of Calycanthaceae. *Mol Phylogenet Evol* 2006; **39**: 1–15.

105. Brenner, GJ. *Flowering Plant Origin, Evolution & Phylogeny*, eds Taylor, DW, Hickey, LJ. (Springer US, Boston, MA), pp 91–115, 1996.

106. Hughes, NF. *The enigma of angiosperm origins*. Cambridge: Cambridge University Press, 1994.

107. Mohr, B. *Org. Deutscher Palaeontologen (ODP) Kolloquium, Freiburg im Breisgau, Germany. Abstracts*, ed Org. Deutscher Palaeontologen. (Freiburg i. Br.), p 40. 1998.

108. Renner, SS, Friis, I, Balslev, H. *Plant diversity and complexity patterns: local, regional and global dimensions: proceedings of an international symposium held at the Royal Danish Academy of Sciences and Letters in Copenhagen, Denmark, 25-28 May, 2003*, eds Ib F, Balslev H, pp 441–458. 2005.

109. Poole, I, Francis, JE. The first record of fossil atherospermataceous wood from the upper Cretaceous of Antarctica. *Rev Palaeobot Palynol* 1999; **107**: 97–107.

110. Kenicer, GJ, Kajita, T, Pennington, RT *et al*. Systematics and biogeography of *Lathyrus* (Leguminosae) based on internal transcribed spacer and cpDNA sequence data. *Am J Bot* 2005; **92**: 1199–1209.

111. Li, J, Davis, CC, Del Tredici, P *et al*. Phylogeny and biogeography of Taxus (Taxaceae) inferred from sequences of the internal transcribed spacer region of nuclear ribosomal DNA. *Harv pap in bot* 2001; **6**: 267–274.

112. Florin, R. *The distribution of conifer and taxad genera in time and space*. Uppsala: Almqvist

& Wiksells boktr, 1963.

113. Li, J-H Xiang, Q-P. Phylogeny and biogeography of *Thuja* L. (Cupressaceae), an eastern Asian and North American disjunct genus. *J Integr Plant Bio* 2005; **47**: 651–659.

114. Yi, T, Miller, AJ, Wen, J. Phylogenetic and biogeographic diversification of *Rhus* (Anacardiaceae) in the Northern Hemisphere. *Mol Phylogenet Evol* 2004; **33**: 861–879.

115. Anderson, WR. An integrated system of classification of flowering plants. *Brittonia* 1982; **34**: 268–270.

116. Becker, HF. The Metzel Ranch Flora of the Upper Ruby River Basin, southwestern Montana. *Palaeontogr Abteil B* 1972; 1–61.

117. Zohary, M. A monographical study of the genus *Pistacia*. *Palest J Bot (Jerusalem Series)* 1952; **5**:187–228.

118. Wei, X-X, Yang, Z-Y, Li Y *et al*. Molecular phylogeny and biogeography of *Pseudotsuga* (Pinaceae): Insights into the floristic relationship between Taiwan and its adjacent areas. *Mol Phylogenet Evol* 2010; **55**: 776–785.

119. Schorn, HE. A preliminary discussion of fossil larches (*Larix*, Pinaceae) from the arctic. *Quatern Int* 1994; **22**:173–183.

120. Nie, Z-L, Sun, H, Li H *et al*. Intercontinental biogeography of subfamily Orontioideae (*Symplocarpus*, *Lysichiton*, and *Orontium*) of Araceae in Eastern Asia and North America. *Mol Phylogenet Evol* 2006; **40**: 155–165.

121. Bogner, J, Hoffman, GL, Aulenback, KR. A fossilized aroid infructescence, *Albertarum pueri* gen.nov. et sp.nov., of Late Cretaceous (Late Campanian) age from the Horseshoe Canyon Formation of southern Alberta, Canada. *Can J Bot* 2005; **83**: 591–598.

122. Friis, EM, Pedersen, KR, Crane, PR. Araceae from the Early Cretaceous of Portugal. Evidence on the emergence of monocotyledons. *Proc Natl Acad Sci USA* 2004; **101**: 16565–16570.

123. Robbrecht, E. Generic distribution patterns in subsaharan African Rubiaceae (*Angiospermae*). *J Biogeogr* 1996; **23**: 311–328.

124. Manchester, SR. Biogeographical relationships of North American tertiary floras. *Ann Missouri Bot Gard* 1999; **86**: 472.

125. Meyer, HW, Manchester, SR. *The oligocene Bridge Creek flora of the John Day formation, Oregon*. Berkeley California: University of California Press, 1997.

126. Graham, A. Studies in Neotropical Paleobotany. IV. The Eocene Communities of Panama. *Ann Missouri Bot Gard* 1985;**72**: 504.

127. Roth, JL, Dilcher, DL. Investigations of angiosperms from the Eocene of North America: Stipulate leaves of the Rubiaceae including a probable polyploid population. *Am J Bot* 1979; **66**: 1194.

128. Harris, AJ, Xiang ,Q-Y, Thomas, DT. Phylogeny, origin, and biogeographic history of *Aesculus* L. (Sapindales) - an update from combined analysis of DNA sequences, morphology,

and fossils. *Taxon* 2009; **58**: 108–126.

129. Manchester, SR. Leaves and Fruits of *Aesculus* (Sapindales) from the Paleocene of North America. *Int J Plant Sci* 2001; **162**: 985–998.

130. Hao, Z-Z, Liu, Y-Y, Nazaire, M *et al*. Molecular phylogenetics and evolutionary history of sect. Quinquefoliae (*Pinus*): Implications for Northern Hemisphere biogeography. *Mol Phylogenet Evol* 2015; **87**: 65–79.

131. Ryberg, PE, Rothwell, GW, Stockey, RA *et al.* Reconsidering Relationships among Stem and Crown Group Pinaceae. Oldest Record of the Genus *Pinus* from the Early Cretaceous of Yorkshire, United Kingdom. *Int J Plant Sci* 2012; **173**: 917–932.

132. Alvin, KL. Further Conifers of the Pinaceae from the Wealden Formation of Belgium. *Inst R Sci Nat Belg Mem* 1960; **146**: 1–39.

133. Mao, KS, Hao, G, Liu JQ *et al*. Diversification and biogeography of *Juniperus* (Cupressaceae): variable diversification rates and multiple intercontinental dispersals. *New Phytol* 2010; **188**: 254–272.

134. Stockey, RA, Kvaček, J, Hill, RS *et al*. *A monograph of Cupressaceae and Sciadopitys*, ed Farjon A. (Royal Botanic Gardens Kew, Kew), pp 54–68, 2005.

135. McIver, EE. An early *Chamaecyparis* (Cupressaceae) from the Late Cretaceous of Vancouver Island, British Columbia, Canada. *Can J Bot* 1994; **72**: 1787–1796.

136. Kvaček, Z. A new juniper from the Palaeogene of Central Europe. *Fedd Repert* 2002; **113**: 492–502.

137. Axelrod, DI. *The late Oligocene Creede flora, Colorado*. California Berkeley: University of California Press, 1987.

138. Axelrod, DI. *The early Miocene Buffalo Canyon flora of western Nevada*. California Berkeley: University of California Press, 1991.

139. Kvaček, Z. An ancient *Calocedrus* (Cupressaceae) from the European Teriary. *Flora* 1999; **194**: 237–248.

140. Kvaček, Z, Manchester, SR, Schorn, HE. Cones, Seeds, and Foliage of Tetraclinis Salicornioides (Cupressaceae) from the Oligocene and Miocene of Western North America. A Geographic Extension of the European Tertiary Species. *Int J Plant Sci* 2000;**161**: 331–344.

141. McIver, EE, Basinger, JF. Fossil seed cones of *Fokienia* (Cupressaceae) from the Paleocene Ravenscrag Formation of Saskatchewan, Canada. *Can J Bot* 1990; **68**: 1609–1618.

142. McIver, EE. Fossil *Fokienia* (Cupressaceae) from the Paleocene of Alberta, Canada. *Can J Bot* 1992; **70**: 742–749.

143. Xiang, Q-P, Wei, R, Shao, Y-Z *et al.* Phylogenetic relationships, possible ancient hybridization, and biogeographic history of *Abies* (Pinaceae) based on data from nuclear, plastid, and mitochondrial genomes. *Mol Phylogenet Evol* 2015; **82**: 1–14.

144. Schorn, HE, Wehr, WC. *Abies milleri,* sp. nov., from the Middle Eocene Klondike mountain formation, Republic, Ferry county, Washington. *Burke Museum contributions in anthropology*

and natural history 1986; **1**: 1–7.

145. Liu, T. *A monograph of the genus Abies* (Taipei), 1971.

146. Zhou, Z, Wen, J, Li, G *et al*. Phylogenetic assessment and biogeographic analyses of tribe *Peracarpeae* (Campanulaceae). *Plant Sys Evol* 2012; **298**: 323–336.

147. Srodoniowa, LM. Macroscopic plant remains from the freshwater Miocene of the Nowy Sacz Basin (west Carpathians, Poland). *Acta Palaeobot* 1979; **20**: 3–117.

148. Cellinese, N, Smith, SA, Edwards, EJ *et al*. Historical biogeography of the endemic Campanulaceae of Crete. *J Biogeogr* 2009; **36**: 1253–1269.

149. Cosner, ME, Raubeson, LA, Jansen, RK. Chloroplast DNA rearrangements in Campanulaceae. Phylogenetic utility of highly rearranged genomes. *BMC evol biol* 2004; **4**: 27.

150. Wikström, N, Savolainen, V, Chase, MW. Evolution of the angiosperms. Calibrating the family tree. *Proc Biol sci* 2001; **268**: 2211–2220.

151. Chen, C-H, JenPan, H, Tsai, CC *et al*. Phylogeny of *Calocedrus* (Cupressaceae), an eastern Asian and western North American disjunct gymnosperm genus, inferred from nuclear ribosomal nrITS sequences. *Bot Stud* 2009; **50**: 425–433.

152. Magallón, S, Herendeen, PS, Crane, PR. *Androdecidua endressii* gen. et sp. nov., from the Late Cretaceous of Georgia (United States): Further floral diversity in Hamamelidoideae (Hamamelidaceae). *Int J Plant Sci* 2001; **162**: 963–983.

153. Zhang, J-Q, Meng, S-Y, Allen, GA *et al*. Rapid radiation and dispersal out of the Qinghai-Tibetan Plateau of an alpine plant lineage *Rhodiola* (Crassulaceae). *Mol Phylogenet Evol* 2014; 77: 147–158.

154. Frederiksen, NO. Sporomorphs from the Jackson Group (upper Eocene) and adjacent strata of Mississippi and western Alabama. *Professional Paper* 1980.

155. Bell, CD, Soltis, DE, Solti, PS. The age and diversification of the angiosperms re-revisited. *Am J Bot* 2010; **97**: 1296–1303.

156. Le Péchon, T, Zhang, L, He, H *et al*. A well-sampled phylogenetic analysis of the polystichoid ferns (Dryopteridaceae) suggests a complex biogeographical history involving both boreotropical migrations and recent transoceanic dispersals. *Mol Phylogenet Evol* 2016; **98**: 324–336.

157. Lóriga, J, Schmidt, AR, Moran, RC *et al*. The first fossil of a bolbitidoid fern belongs to the early-divergent lineages of Elaphoglossum (Dryopteridaceae). *Am J Bot* 2014; **101**: 1466–1475.

158. Vikulin, SV, Bobrov, AE. New fossil genus *Protodrynaria* (Polypodiaceae) from the Paleogene flora of Tim (the south of the Middle Russian upland). *Botanicheskii zhurnal*. 1987.

159. Schuettpelz, E, Pryer, KM. Evidence for a Cenozoic radiation of ferns in an angiosperm-dominated canopy. *Proc Natl Acad Sci USA* 2009; **106**: 11200–11205.

160. Yuan, Z-Y, Zhou, W-W, Chen, X *et al*. Spatiotemporal diversification of the True Frogs

(genus *Rana*). A Historical framework for a widely studied group of model organisms. *Syst Biol* 2016; **65**: 824–842.

161. Bossuyt, F, Brown, RM, Hillis, DM *et al.* Phylogeny and biogeography of a cosmopolitan frog radiation: Late Cretaceous diversification resulted in continent-scale endemism in the family Ranidae. *Syst Biol* 2006; **55**: 579–594.

162. Holman, AJ. Early Miocene anurans from Florida. *Quarterly Journal of the Florida Academy of Sciences* 1965; **28**: 68–82.

163. Holman, AJ. Additional Miocene anurans from Florida. *Quarterly Journal of the Florida Academy of Sciences* 1968; **30**: 121–140.

164. Voorhies, MR, Holman, AJ, Xiang-Xu X. The Hottell Ranch rhino quarries (basal Ogallala; medial Barstovian), Banner County, Nebraska; Part I; Geologic setting, faunal lists, lower vertebrates. *Rocky Mount Geol* 1987; **25**: 55–69.

165. Böhme, M The oldest representative of a brown frog (Ranidae) from the Early Miocene of Germany. *Acta Palaeontol Polon* 2001; **46**: 119–124.

166. Li, J-T, Wang, J-S, Nian, H-H *et al.* Amphibians crossing the Bering Land Bridge. Evidence from holarctic treefrogs (*Hyla*, Hylidae, Anura). *Mol Phylogenet Evol* 2015; **87**: 80–90.

167. Holman, AJ. *Fossil frogs and toads of North America* Bloomington: Indiana University Press, 2003.

168. Wiens, JJ, Graham, CH, Moen, DS *et al*. Evolutionary and ecological causes of the latitudinal diversity gradient in hylid frogs. Treefrog trees unearth the roots of high tropical diversity. *Am nat* 2006; **168**: 579–596.

169. Smith, SA, Oca, ANM, de Reeder TW *et al*. A phylogenetic perspective on elevational species richness patterns in Middle American treefrogs. Why so few species in lowland tropical rainforests? *Evol int J org evol* 2007; **61**: 1188–1207.

170. Smith, SA, Stephens, PR, Wiens, JJ. Replicate patterns of species richness, historical biogeography, and phylogeny in Holarctic treefrogs. *Evol int J org evol* 2005; **59**: 2433.

171. Wiens, JJ, Kuczynski, CA, Hua, X *et al*. An expanded phylogeny of treefrogs (Hylidae) based on nuclear and mitochondrial sequence data. *Mol Phylogenet Evol* 2010; **55**: 871–882.

172. Pramuk, JB, Robertson, T, Sites, JW *et al*. Around the world in 10 million years: Biogeography of the nearly cosmopolitan true toads (Anura: Bufonidae). *Glob Ecol Bioger* 2010; **17**:72-83.

173. Báez, AM, Perí, S. *Baurubatrachus pricei*, nov. gen. et sp. un anuro del Cretácico Superior de Minas Gerais, Brazil. *Anais Da Academia Brasileira De Ciências* 1989; **61**: 447–458.

174. Báez, AM, Gasparini, ZB. *The South American herpetofauna : its origin, evolution, and dispersal /,* eds Duellman WE, League H, Society for the Study of Amphibians, Reptiles., Symposium on the South American Herpetofauna. (Museum of Natural History, University of Kansas, Lawrence, Kan.), pp 29–55. 1979.

175. Antunes, MT, Russell, DE. Le gisement de Silveirinha (bas Mondego, Porugal): La plus ancienne fauna de vértebres éocenes connue en Europe. *Comptes Rendus de l'Académie des*

Sciences de Paris, Série II 1981; **293**: 1099–1102.

176. Guo, P, Liu, Q, Xu, Y *et al.* Out of Asia: Natricine snakes support the Cenozoic Beringian Dispersal Hypothesis. *Mol Phylogenet Evol* 2012; **63**: 825–833.

177. Rage. JC. The oldest known colubrid snakes. State of the art. *Acta Zool Cracov* 1988; **31**: 457–474.

178. Szyndlar, Z. Snake fauna (Reptilia, Serpentes) from the Early/Middle Miocene of Sandelzhausen and Rothenstein 13 (Germany). *Paläontologische Zeitschrift* 2009; **83**: 55–66.

179. Dowling, HG. Fossil snakes of North America: origin, evolution, distribution, paleoecology. *Copeia* 2002; **2002**: 527–529.

180. Burbrink, FT, Lawson, R. How and when did Old World ratsnakes disperse into the New World? *Mol Phylogenet Evol* 2007; **43**: 173–189.

181. Holman, JA. *Fossil snakes of North America: Origin, evolution, distribution, paleoecology* Bloomington: Indiana University Press, 2000.

182. Ivanov, M. The oldest known Miocene snake fauna from Central Europe: Merkur-North locality, Czech Republic. *Acta Palaeontol Polon* 2002; **47**: 513–534.

183. Spinks, PQ, Shaffer, HB, Iverson, JB *et al*. Phylogenetic hypotheses for the turtle family Geoemydidae. *Mol Phylogenet Evol* 2004; **32**: 164–182.

184. Sanguila, MB, Siler, CD, Diesmos, AC *et al*. Phylogeography, geographic structure, genetic variation, and potential species boundaries in Philippine slender toads. *Mol Phylogenet Evol* 2011; **61**: 333–350.

185.Martin, AP, Palumbi, SR. Body size, metabolic rate, generation time, and the molecular clock. *Proc Natl Acad Sci USA* 1993; **90**: 4087–4091.

186. Spinks, PQ, Shaffer, HB. Conflicting mitochondrial and nuclear phylogenies for the widely disjunct *Emys* (Testudines, Emydidae) species complex, and what they tell us about biogeography and hybridization. *Syst Biol* 2009; **58**: 1–20.

187. Near, TJ, Meylan, PA, Shaffer, HB. Assessing concordance of fossil calibration points in molecular clock studies: An example using turtles. *Am nat* 2005; **165**: 137–146.

188. Kelly, CMR, Barker, NP, Villet, MH *et al*. Phylogeny, biogeography and classification of the snake superfamily Elapoidea: A rapid radiation in the late Eocene. *Cladistics* 2009; **25**: 38–63.

189. Szyndlar, Z, Rage, JC. Oldest fossil vipers (Serpentes: Viperidae) from the Old World. *Kaupia* 1999; **8**: 9–20.

190. Sanders, KL, Lee, MSY, Leys, R *et al*. Molecular phylogeny and divergence dates for Australasian elapids and sea snakes (Hydrophiinae): Evidence from seven genes for rapid evolutionary radiations. *J evol bio* 2008; **21**: 682–695.

191. Brandley, MC, Wang, Y, Guo, X *et al.* Accommodating heterogenous rates of evolution in molecular divergence dating methods: An example using intercontinental dispersal of *Plestiodon* (*Eumeces*) lizards. *Syst Biol* 2011; **60**: 3–15.

192. Conrad, JL. Phylogeny and systematics of Squamata (Reptilia) based on morphology. *B Am*

Mus Nat Hist 2008; **310**: 1–182.

193. Nydam, RL, Cifelli, RL. A new teiid lizard from the Cedar Mountain Formation (Albian–Cenomanian boundary) of Utah. *J Vert Paleont* 2002; **22**: 276–285.

194. Wüster, W, Peppin, L, Pook, CE *et al*. A nesting of vipers: Phylogeny and historical biogeography of the Viperidae (Squamata: Serpentes). *Mol Phylogenet Evol* 2008; **49**: 445–459.

195. Wüster, W, Höggren, M, Douglas, ME *et al. Biology of the vipers*. Eagle Mountain: Eagle Mountain Publication, 2002, 111–128.

196. Wüster, V, Crookes, S, Ineich, I *et al.* The phylogeny of cobras inferred from mitochondrial DNA sequences: Evolution of venom spitting and the phylogeography of the African spitting cobras (Serpentes: Elapidae: *Naja nigricollis* complex). *Mol Phylogenet Evol* 2007; **45**: 437–453.

197. Nagy, ZT, Joger, U, Wink, M *et al*. Multiple colonization of Madagascar and Socotra by colubrid snakes. Evidence from nuclear and mitochondrial gene phylogenies. *Proc Biol sci* 2003; **270**: 2613–2621.

198. Parmley, D, Holman, JA. Earliest Fossil Record of a Pigmy Rattlesnake (Viperidae, *Sistrurus* Garman). *J Herpetol* 2007; **41**: 141–144.

199. Head, JJ, Holroyd, PA, Hutchison, JH *et al*. First report of snakes (Serpentes) from the Late Middle Eocene Pondaung Formation, Myanmar. *J Vertebr Paleontol* 2005; **25**: 246–250.

200. Rage, JC, Werner, C Mid-Cretaceous (Cenomanian) snakes from Wadi Abu Hashim, Sudan: The earliest snake assemblage. *Bernard Price Inst Palaeontol Res* 1999; **35**: 85–110.

201. Rage, JC, Tiwari, BN, Thewissen, JGM *et al*. Early Eocene snakes from Kutch, Western India, with a review of the Palaeophiidae. *Geodiversitas* 2003; **25**: 695–716.

202. Pisano, J, Condamine, FL, Lebedev, V *et al.* Out of Himalaya: The impact of past Asian environmental changes on the evolutionary and biogeographical history of Dipodoidea (Rodentia). *J Biogeogr* 2015; **42**: 856–870.

203. McKenna, MC, Bell, SK. *Classification of mammals above the species level* New York: Columbia University Press, 1997.

204. Jacobs, LL, Flynn, LJ. in *Interpreting the past: Essays on human, primate, and mammal evolution in honor of David Pilbeam*, eds Lieberman, D, Smith, RJ, Kelley, J. (Brill Academic Pub, Boston, Mass.), pp 63–80. 2005.

205. Michaux, J, Aguilar, JP, Montuire, S *et al*. Les Murinae (Rodentia, Mammalia) néogènes du Sud de la France. Évolution et paléoenvironnements. *Geobios* 1997; **30**: 379–385.

206. Kimura, Y The earliest record of birch mice from the Early Miocene Nei Mongol, China. *Naturwissenschaften* 2011; **98**: 87–95.

207. Zhang, Q, Xia, L, Kimura, Y *et al.* Tracing the origin and diversification of Dipodoidea (order Rodentia): Evidence from fossil record and molecular phylogeny. *Evol Biol* 2013; **40**: 32–44.

208. Harding, LE, Smith, FA. Mustela or Vison? Evidence for the taxonomic status of the American mink and a distinct biogeographic radiation of American weasels. *Mol Phylogenet Evol* 2009; **52**: 632–642.

209. Wolsan, M. Phylogeny and classification of early European Mustelida (Mammalia: Carnivora). *Acta Theriologica* 1993; **38**: 345–384.

210. Wolsan, M. Oldest mephitine cranium and its implications for the origin of skunks. *Acta Palaeontologica Polonica* 1999; **44**: 223–230.

211. Sato, JJ, Hosoda, T, Wolsan, M *et al.* Phylogenetic relationships and divergence times among mustelids (Mammalia: Carnivora) based on nucleotide sequences of the nuclear interphotoreceptor retinoid binding protein and mitochondrial cytochrome b genes. *Zool Sci* 2003; **20**: 243.

212. Brunhoff, C, Galbreath, KE, Fedorov, VB *et al*. Holarctic phylogeography of the root vole (*Microtus oeconomus*): Implications for late Quaternary biogeography of high latitudes. *Mol Ecol* 2003; **12**: 957–968.

213. Ruedi, M, Mayer, F. Molecular systematics of bats of the genus *Myotis* (Vespertilionidae) suggests deterministic ecomorphological convergences. *Mol Phylogenet Evol* 2001; **21**: 436–448.

214. Matthee, CA, van Vuuren, BJ, Bell, D *et al*. A molecular supermatrix of the rabbits and hares (Leporidae) allows for the identification of five intercontinental exchanges during the Miocene. *Syst Biol* 2004; **53**: 433–447.

215. Springer, MS, Murphy, WJ, Eizirik, E *et al*. Placental mammal diversification and the Cretaceous-Tertiary boundary. *Proc Natl Acad Sci USA* 2003; **100**: 1056–1061.

216. Voorhies, MR, Timperley, CL. A new *Pronotolagus* (Lagomorpha, Leporidae) and other leporids from the Valentine Railway quarries (Barstovian, Nebraska), and the archaeolagine-leporine transition. *J Vertebr Paleontol* 2010;**17**: 725–737.

217. Su, C, Nei, M. Fifty-million-year-old polymorphism at an immunoglobulin variable region gene locus in the rabbit evolutionary lineage. *Proc Natl Acad Sci USA* 1999; **96**: 9710–9715.

218. Yamada, F, Takaki, M, Suzuki, H. Molecular phylogeny of Japanese Leporidae, the Amami rabbit *Pentalagus furnessi*, the Japanese hare *Lepus brachyurus*, and the mountain hare *Lepus timidus*, inferred from mitochondrial DNA sequences. *Genes Genet Syst* 2002; **77**: 107–116.

219. Ren, Z, Zhong, Y, Kurosu, U *et al.* Historical biogeography of Eastern Asian-Eastern North American disjunct *Melaphidina* aphids (Hemiptera: Aphididae: Eriosomatinae) on *Rhus* hosts (Anacardiaceae). *Mol Phylogenet Evol* 2013; **69**: 1146–1158.

220. Heie, OE, Wegierek, P. A list of fossil aphids (Homoptera: Aphidinea). *Ann Upper Silesian Mus Entomol* 1998; **8**: 159–192.

221. Ye, Z, Zhen, Y, Zhou, Y *et al*. Out of Africa: Biogeography and diversification of the pantropical pond skater genus *Limnogonus* Stål, 1868 (Hemiptera: Gerridae). *Ecol Evol* 2017; **7**: 793–802.

222. Andersen, NM. Fossil water striders in the Eocene Baltic amber (Hemiptera, Gerromorpha).

Insect Sys Evol 2000; **31**: 257–284.

223. Damgaard, J, Moreira, FFF, Zettel, H *et al*. Molecular phylogeny of the pond skaters (Gerrinae), discussion of the fossil record and a checklist of species assigned to the subfamily (Hemiptera:Heteroptera: Gerridae). *Insect Sys Evol* 2014; **45**: 251–281.

224. Zettel, H, Heiss, E. New species of water striders (Hemiptera: Heteroptera: Gerromorpha: Hydrometridae, Gerridae) from Eocene Baltic amber. *Annalen des Naturhistorischen Museums in Wien. Serie A für Mineralogie und Petrographie, Geologie und Paläontologie, Anthropologie und Prähistorie* 2011; **113**: 543–553.

225. Rodriguez, J, Pitts, JP, Dohle, CD *et al*. Historical biogeography of the widespread spider wasp tribe Aporini (Hymenoptera:Pompilidae). *J Biogeogr* 2015; **42**: 495–506.

226. Wilson, JS, Dohlen, CD, von Forister ML *et al*. Family-level divergences in the stinging wasps (Hymenoptera: Aculeata), with correlations to angiosperm diversification. *Evol Biol* 2013; **40**: 101–107.

227. Condamine, FL, Sperling, FAH, Kergoat, GJ *et al*. Global biogeographical pattern of swallowtail diversification demonstrates alternative colonization routes in the Northern and Southern hemispheres. *J Biogeogr* 2013; **40**: 9–23.

228. Simonsen, TJ, Zakharov, EV, Djernaes, M *et al.* Phylogenetics and divergence times of Papilioninae (Lepidoptera) with special reference to the enigmatic genera *Teinopalpus* and *Meandrusa*. *Cladistics* 2011; **27**: 113–137.

229. Ohshima, I, Tanikawadodo, Y, Saigusa, T *et al.* Phylogeny, biogeography, and host-plant association in the subfamily Apaturinae (Insecta:Lepidoptera: Nymphalidae) inferred from eight nuclear and seven mitochondrial genes. *Mol Phylogenet Evol* 2010; **57**: 1026–1036.

230. Wahlberg, N, Leneveu, J, Kodandaramaiah, U *et al.* Nymphalid butterflies diversify following near demise at the Cretaceous/Tertiary boundary. *Proc Biol sci* 2009; **276**: 4295–4302.

231. Cai, Q, Tulloss, RE, Tang, L-P *et al.* Multi-locus phylogeny of lethal amanitas: Implications for species diversity and historical biogeography. *BMC evol biol*2014; **14**: 143.

232. Berbee, ML, Taylor, JW. Dating the molecular clock in fungi – how close are we? *Fungal Biol Rev* 2010; **24**: 1–16.

233. Smith, SY, Currah, RS, Stockey, RA. Cretaceous and Eocene poroid hymenophores from Vancouver Island, British Columbia. *Mycologia* 2004; **96**: 180–186.

Supplementary Data: Phylograms with estimated divergence times (maximum clade credibility trees) and ancestral areas (DEC-model of range evolution); the latter annotated for each phylogeny once with best range estimate and once with relative likelihood of ranges as pie chart.

Woodwardia BioGeoBEARS DEC

ancstates: global optim, 3 areas max. d=0.0013; e=0; j=0; LnL=−17.40

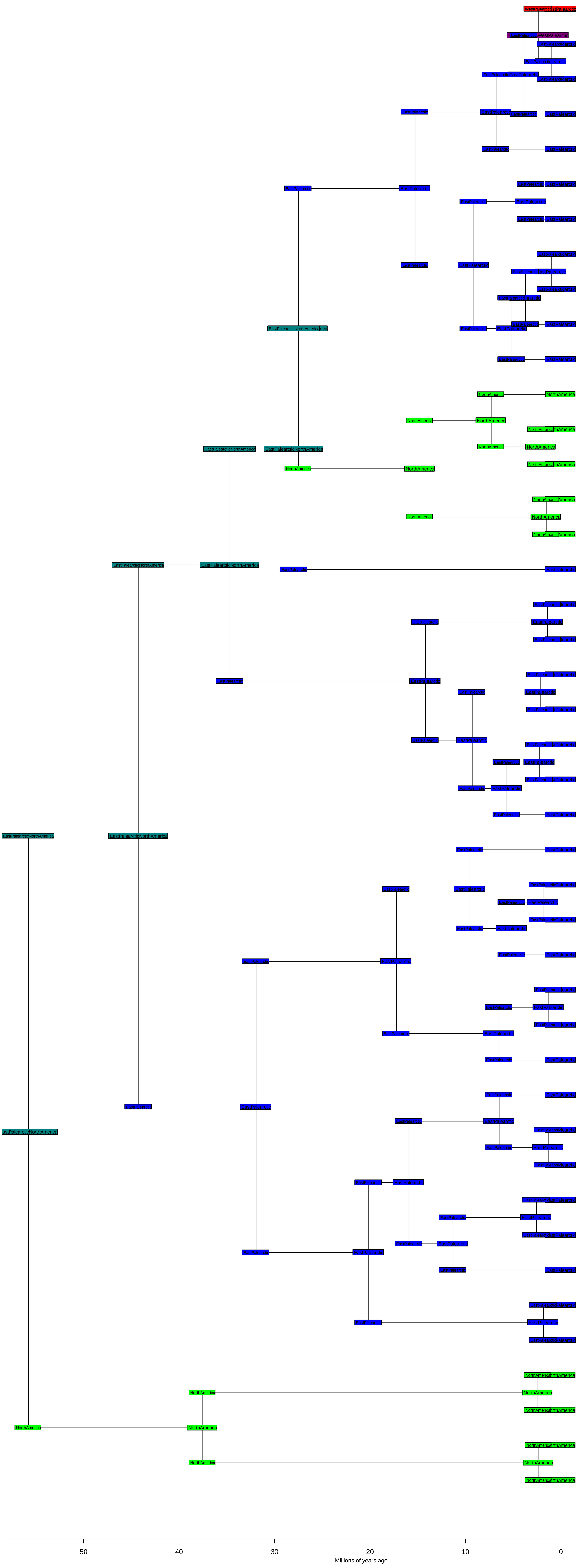

Woodwardia BioGeoBEARS DEC

ancstates: global optim, 3 areas max. d=0.0013; e=0; j=0; LnL=−17.40

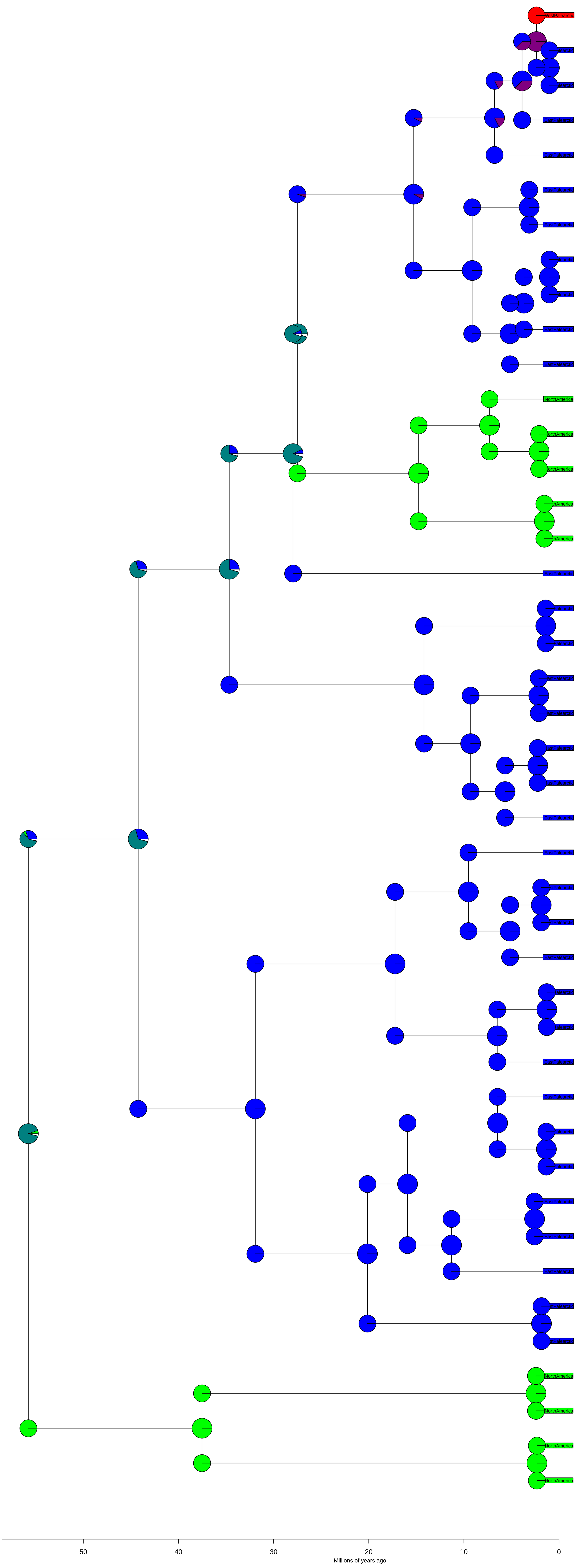

Picea BioGeoBEARS DEC

ancstates: global optim, 3 areas max. d=0.0028; e=0; j=0; LnL=−51.16

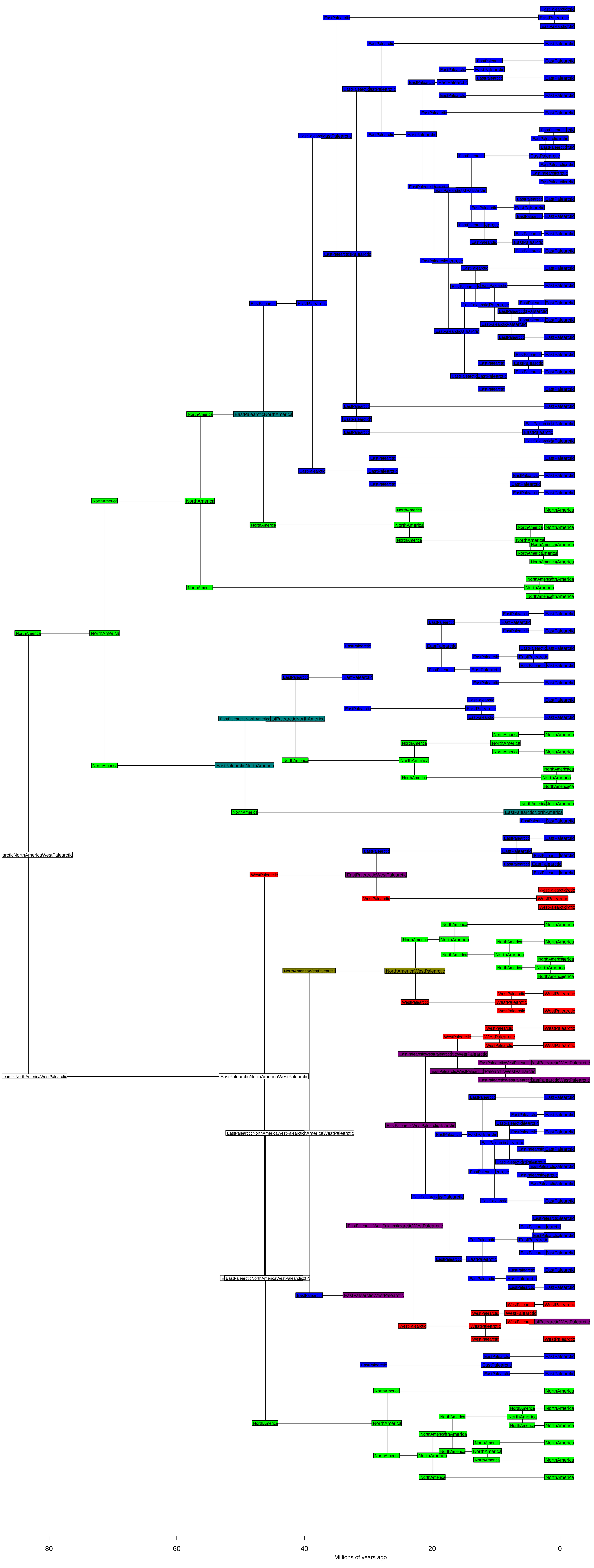

Picea BioGeoBEARS DEC

ancstates: global optim, 3 areas max. d=0.0028; e=0; j=0; LnL=−51.16

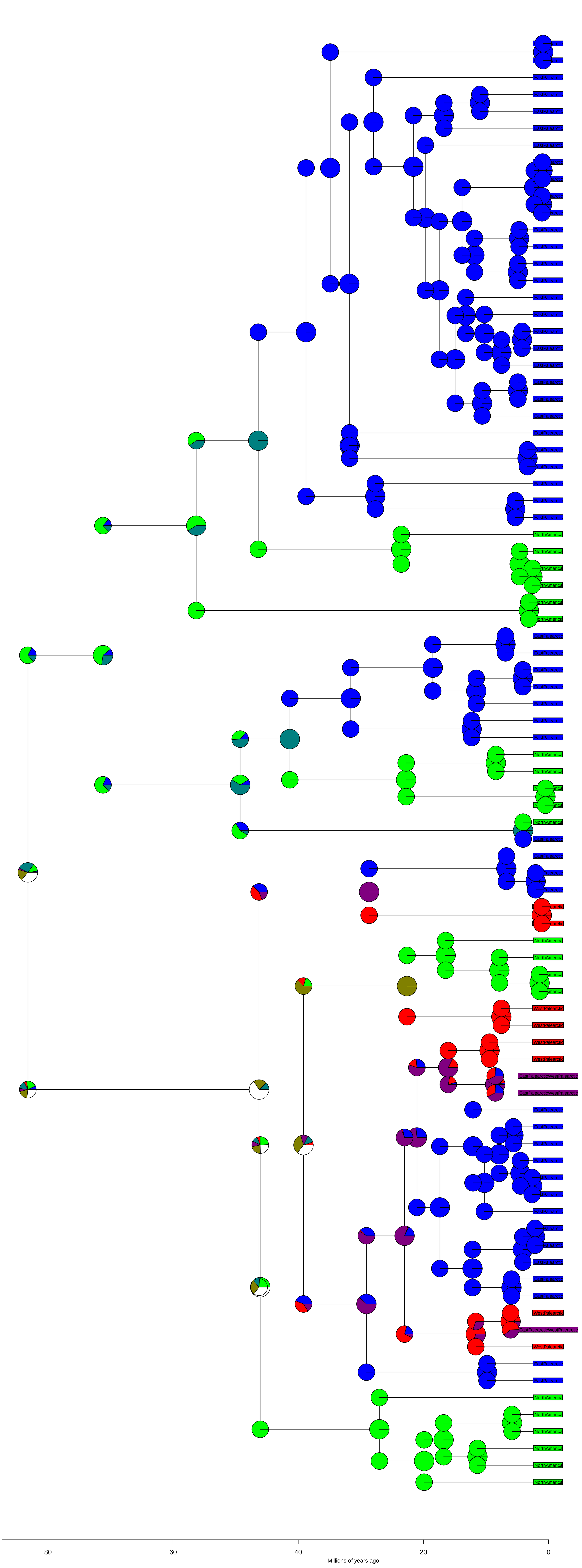

Osmorhiza BioGeoBEARS DEC

ancstates: global optim, 3 areas max. d=0.0201; e=0; j=0; LnL=−34.13

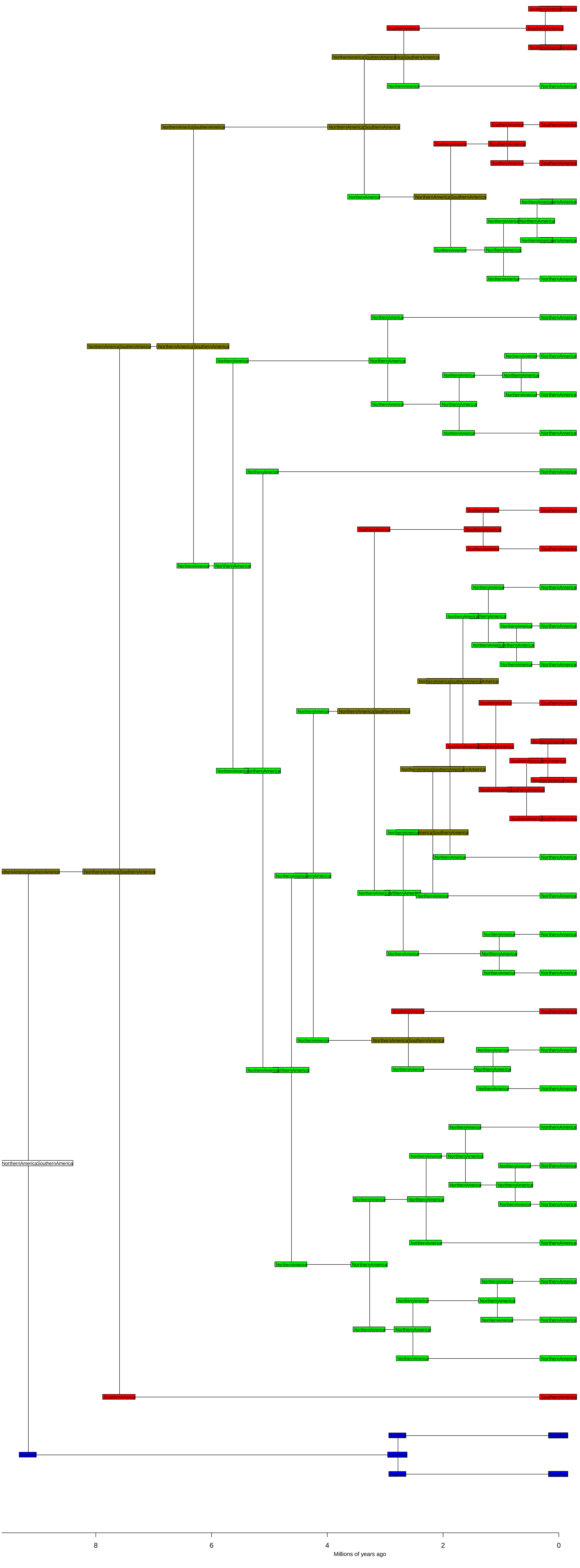

Osmorhiza BioGeoBEARS DEC

ancstates: global optim, 3 areas max. d=0.0201; e=0; j=0; LnL=−34.13

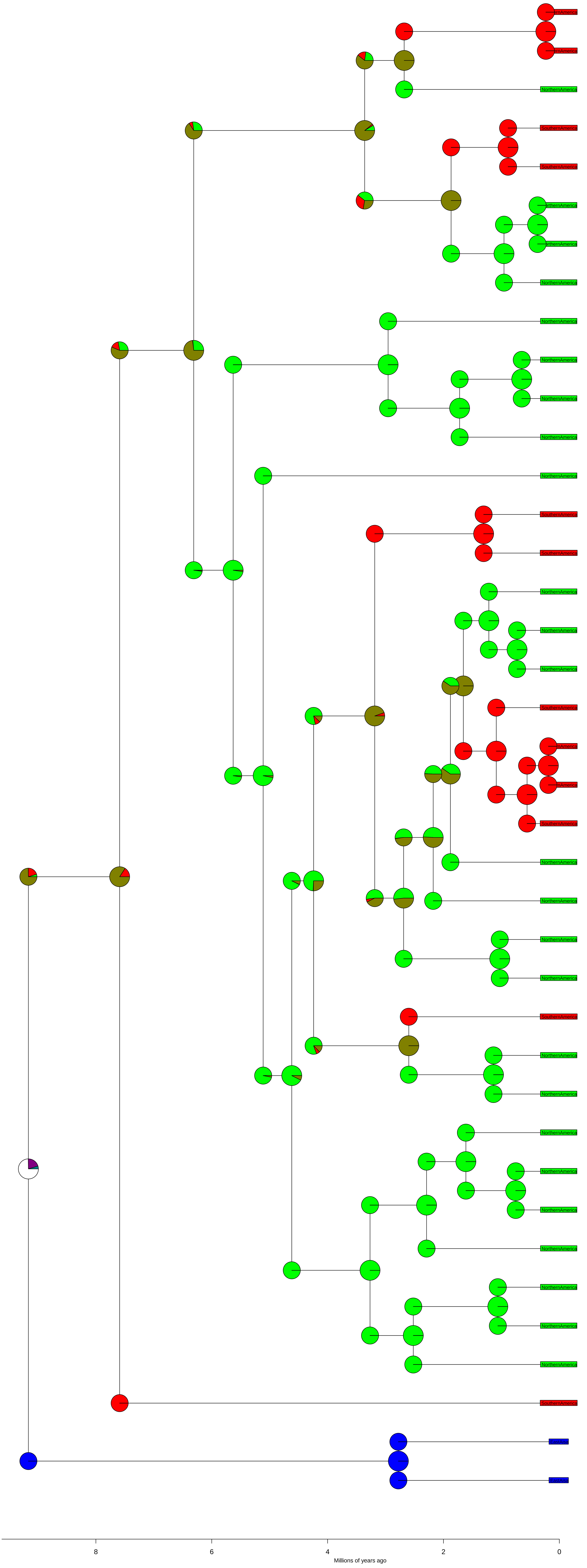

Prunus BioGeoBEARS DEC

ancstates: global optim, 3 areas max. d=0.007; e=0.0019; j=0; LnL=−222.91

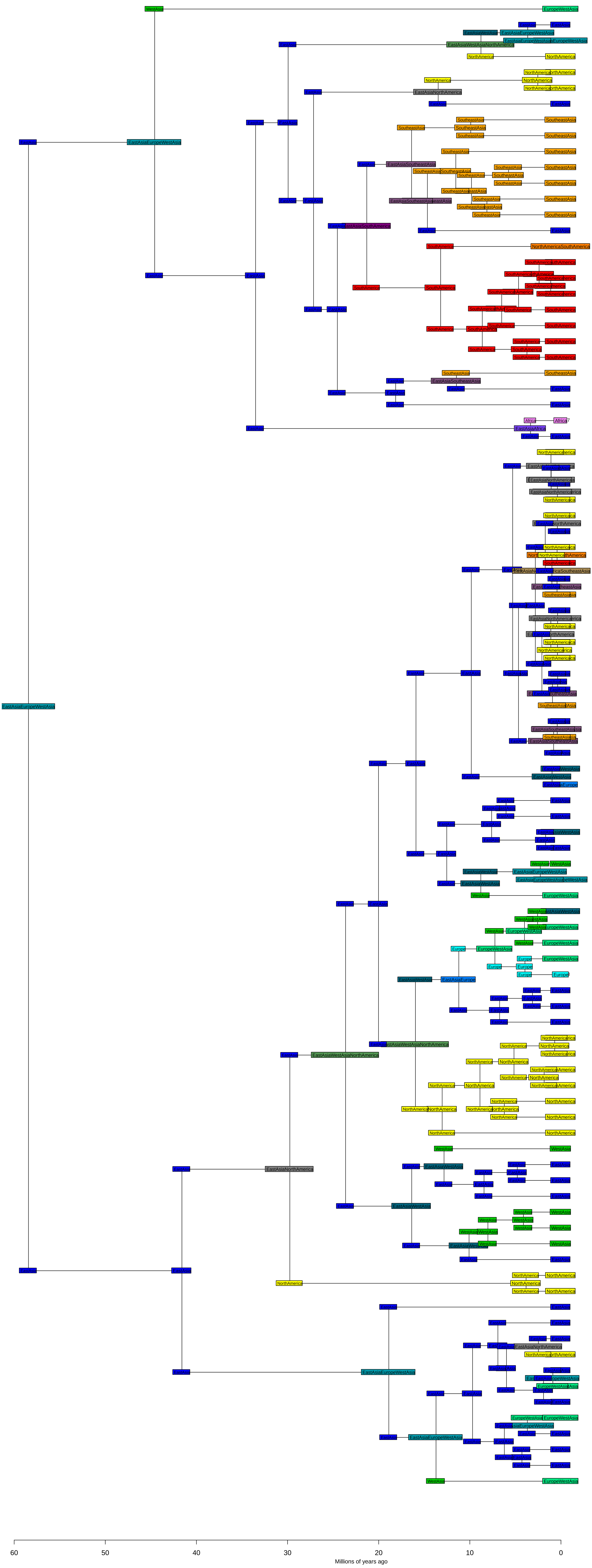

Prunus BioGeoBEARS DEC

ancstates: global optim, 3 areas max. d=0.007; e=0.0019; j=0; LnL=−222.91

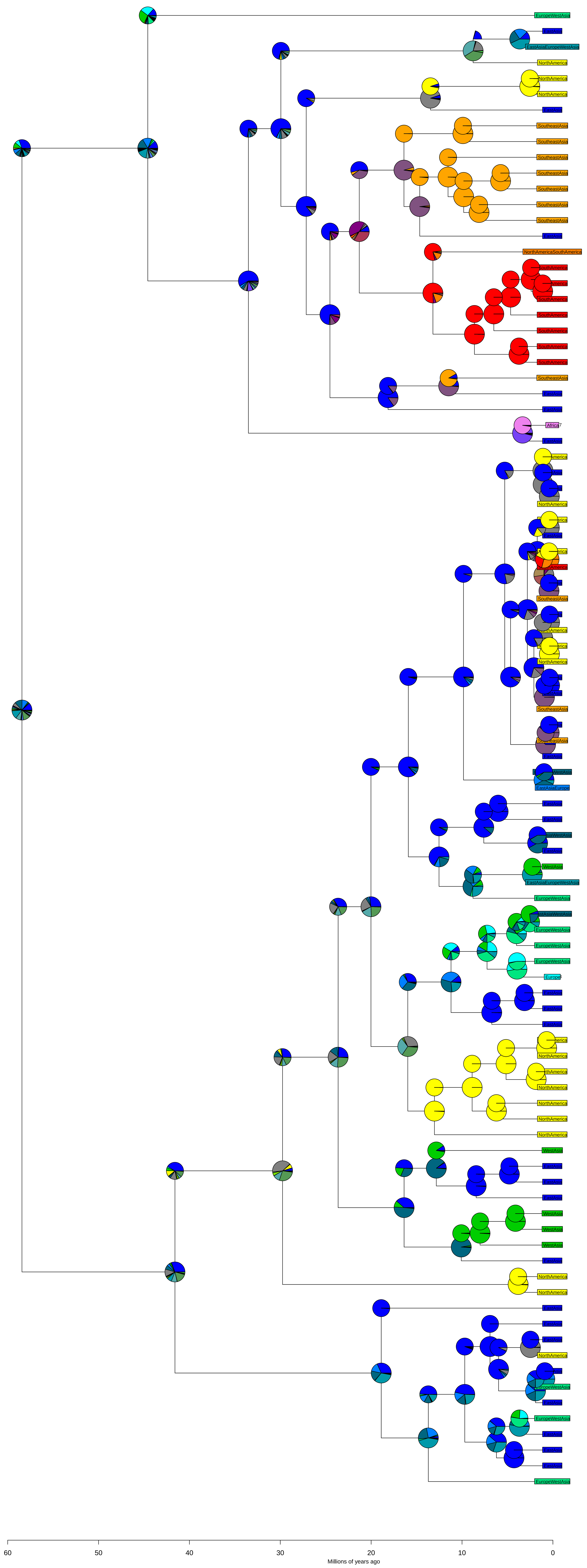

Patis and *Ptilagrostis* BioGeoBEARS DEC

ancstates: global optim, 3 areas max. d=0.004; e=0.0041; j=0; LnL=−121.29

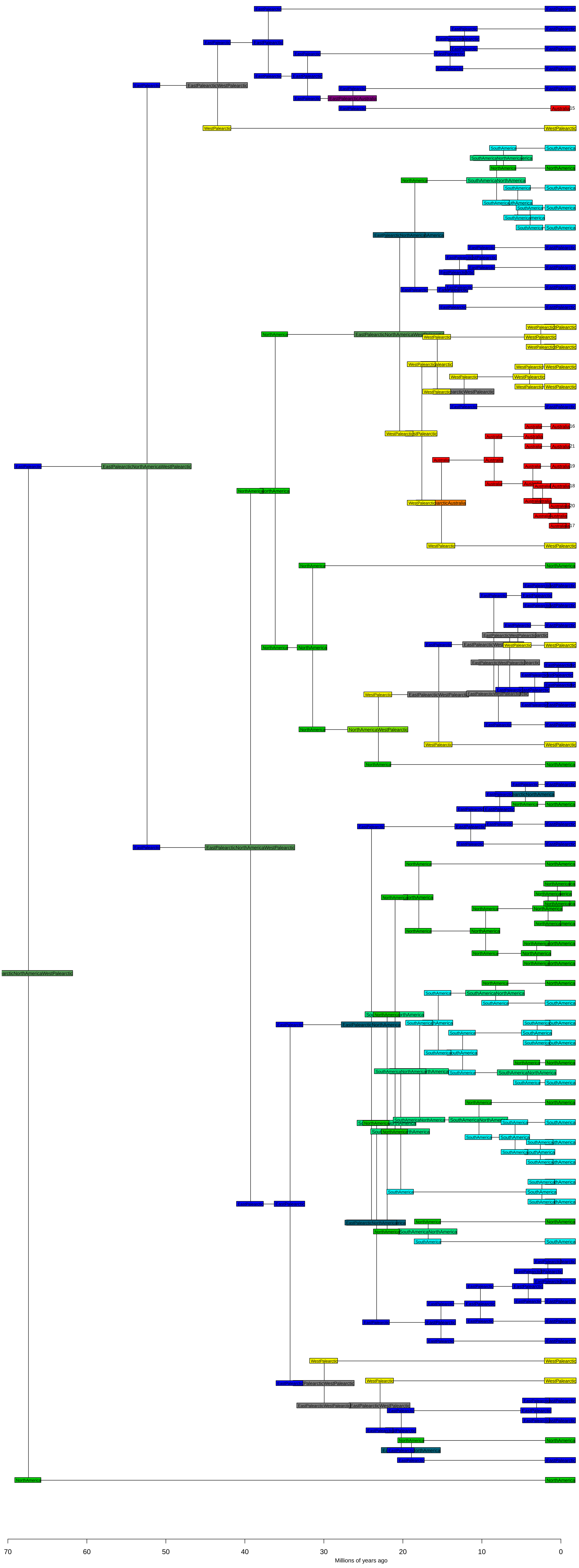

Patis and *Ptilagrostis* BioGeoBEARS DEC

ancstates: global optim, 3 areas max. d=0.004; e=0.0041; j=0; LnL=−121.29

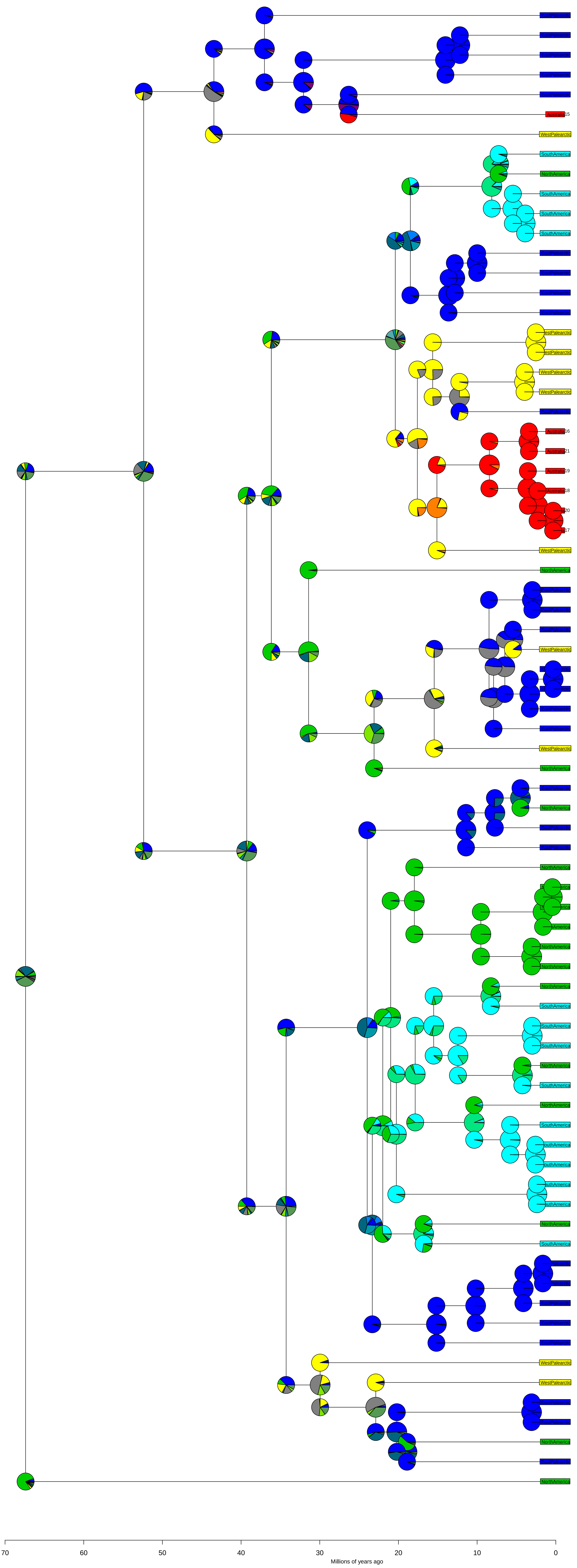

Aristolochia BioGeoBEARS DEC

ancstates: global optim, 3 areas max. d=0.0023; e=0; j=0; LnL=−16.01

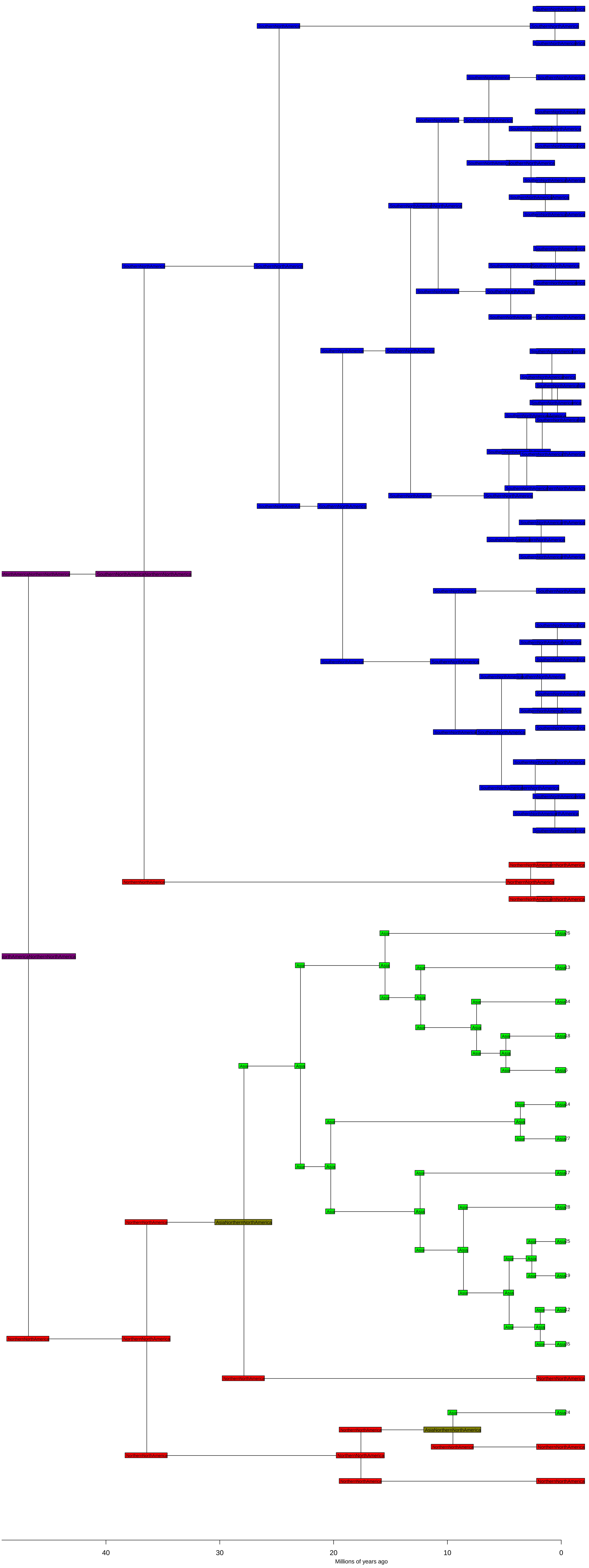

Aristolochia BioGeoBEARS DEC

ancstates: global optim, 3 areas max. d=0.0023; e=0; j=0; LnL=−16.01

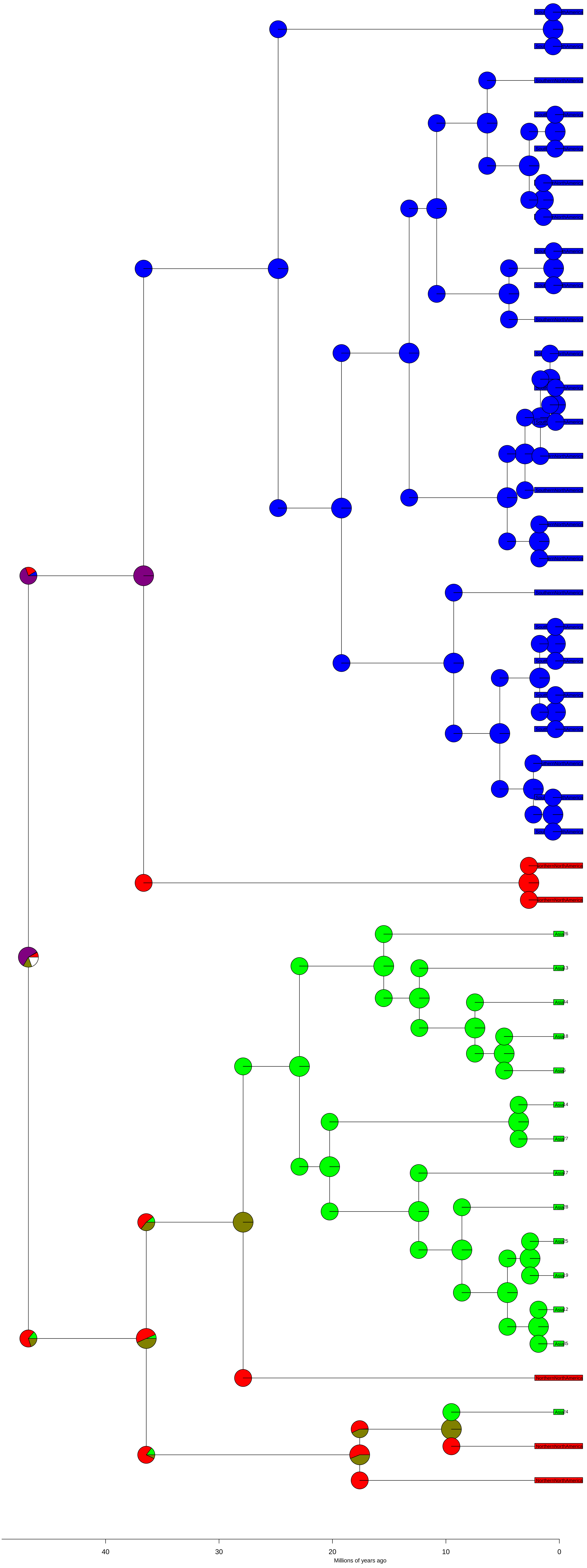

Astilbe BioGeoBEARS DEC

ancstates: global optim, 3 areas max. d=0; e=0; j=0; LnL=−5.38

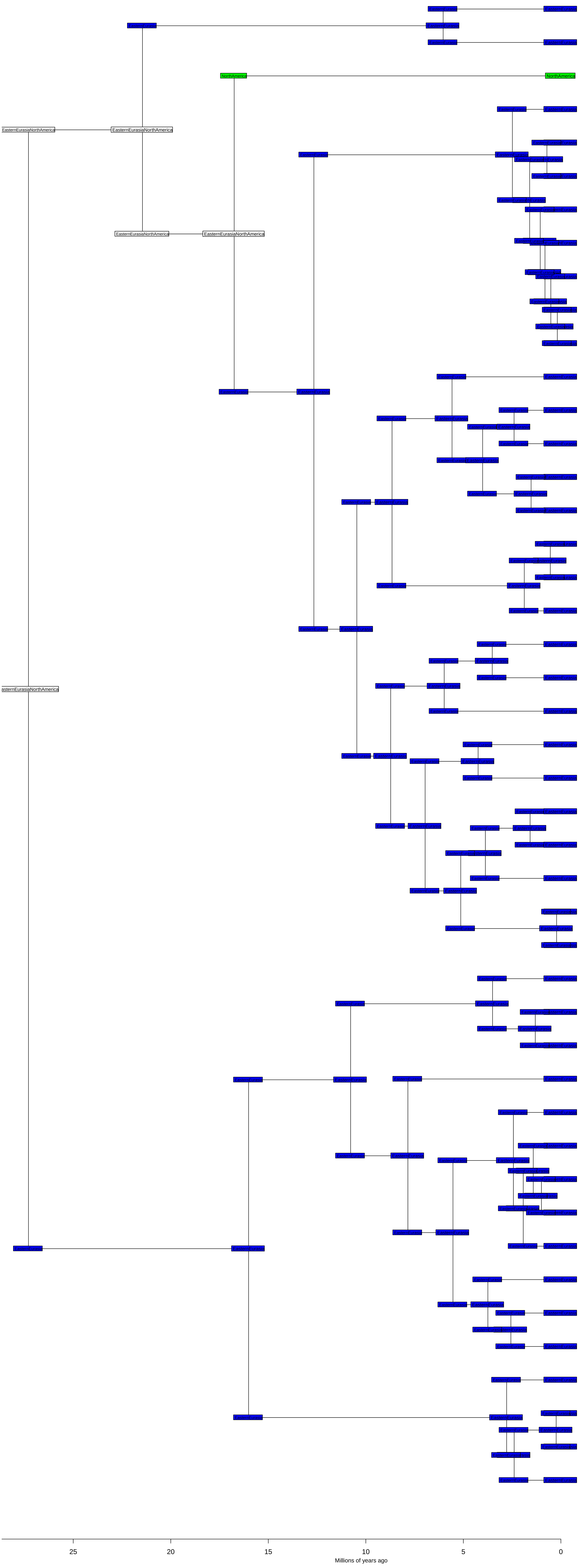

Astilbe BioGeoBEARS DEC

ancstates: global optim, 3 areas max. d=0; e=0; j=0; LnL=−5.38

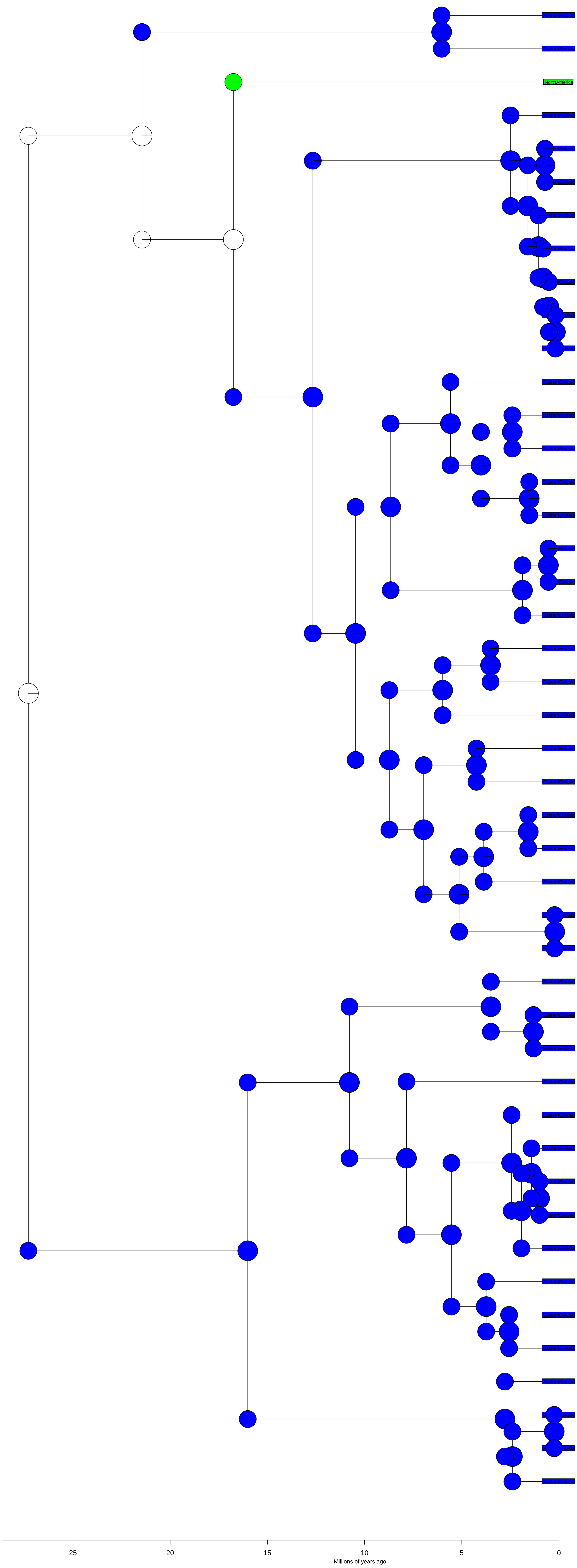

Ranunculeae BioGeoBEARS DEC

ancstates: global optim, 3 areas max. d=0.0134; e=0.0084; j=0; LnL=-148.91

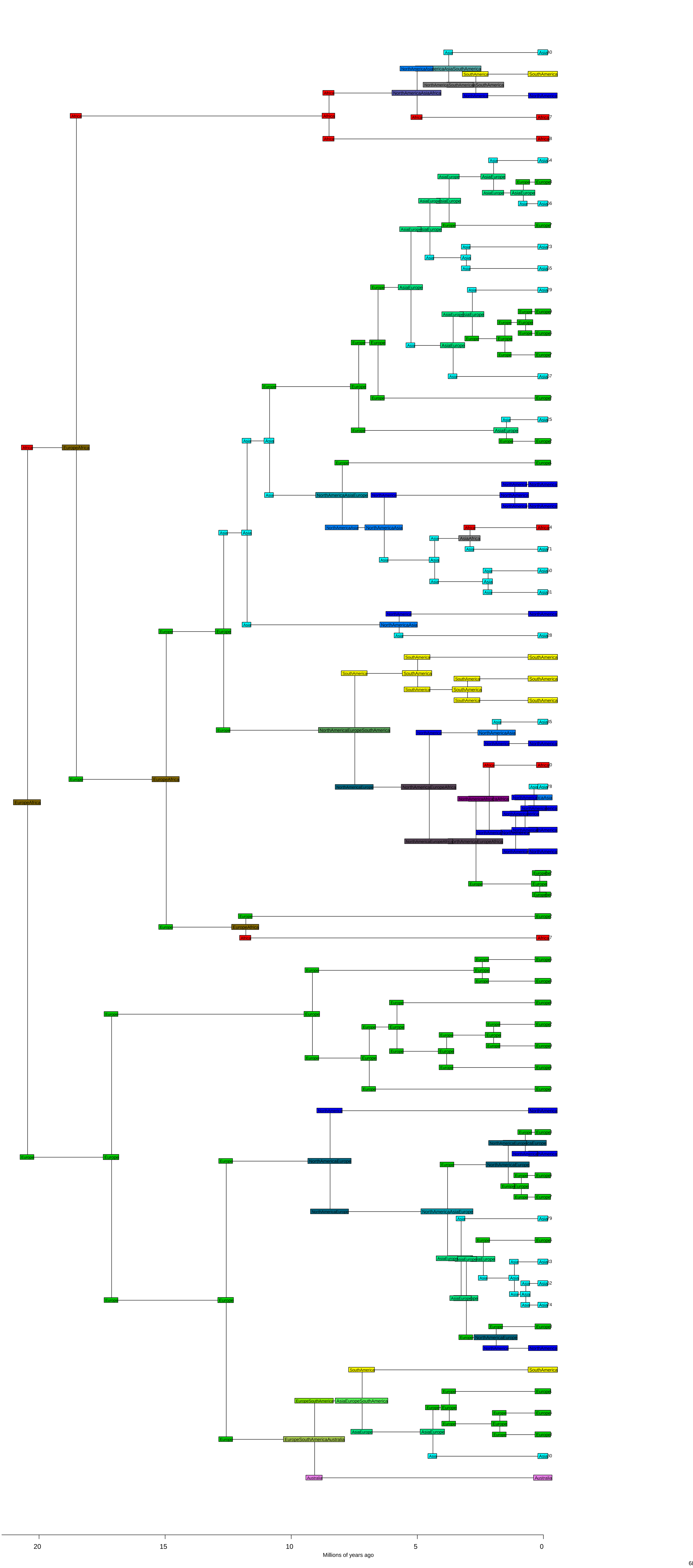

Ranunculeae BioGeoBEARS DEC

ancstates: global optim, 3 areas max. d=0.0134; e=0.0084; j=0; LnL=−148.91

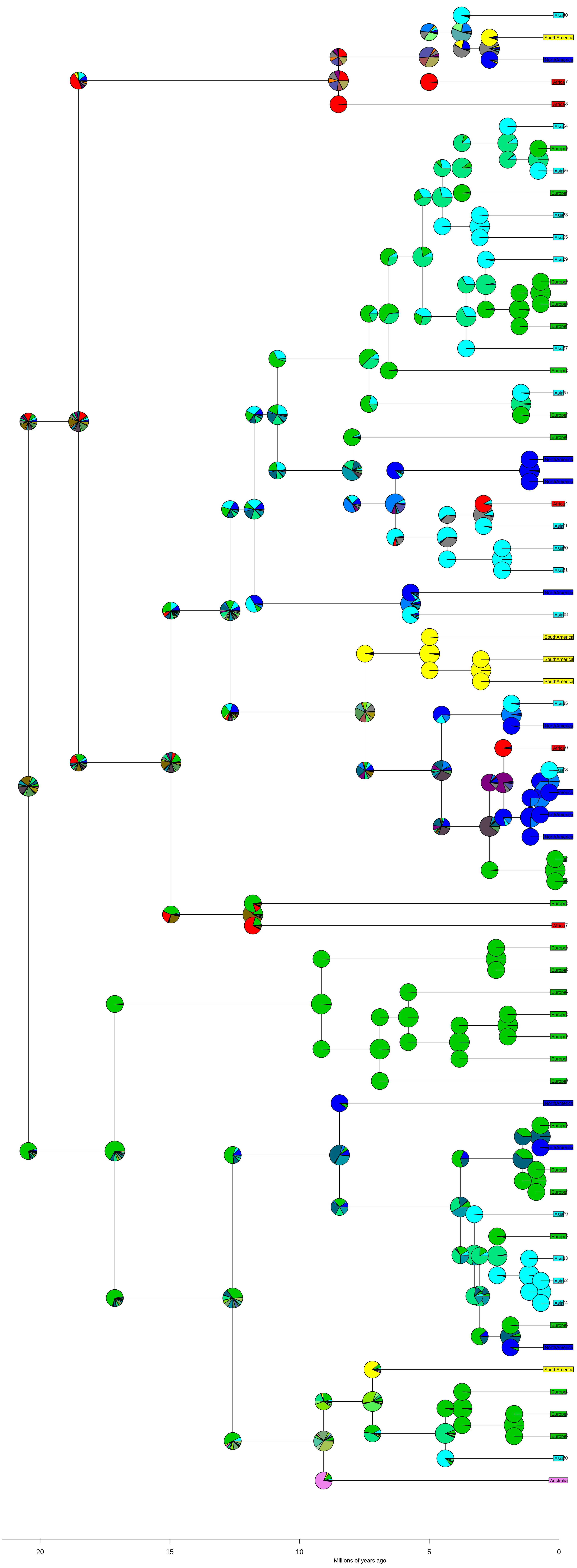

Scrophularia BioGeoBEARS DEC

ancstates: global optim, 3 areas max. d=0.0048; e=0; j=0; LnL=−22.58

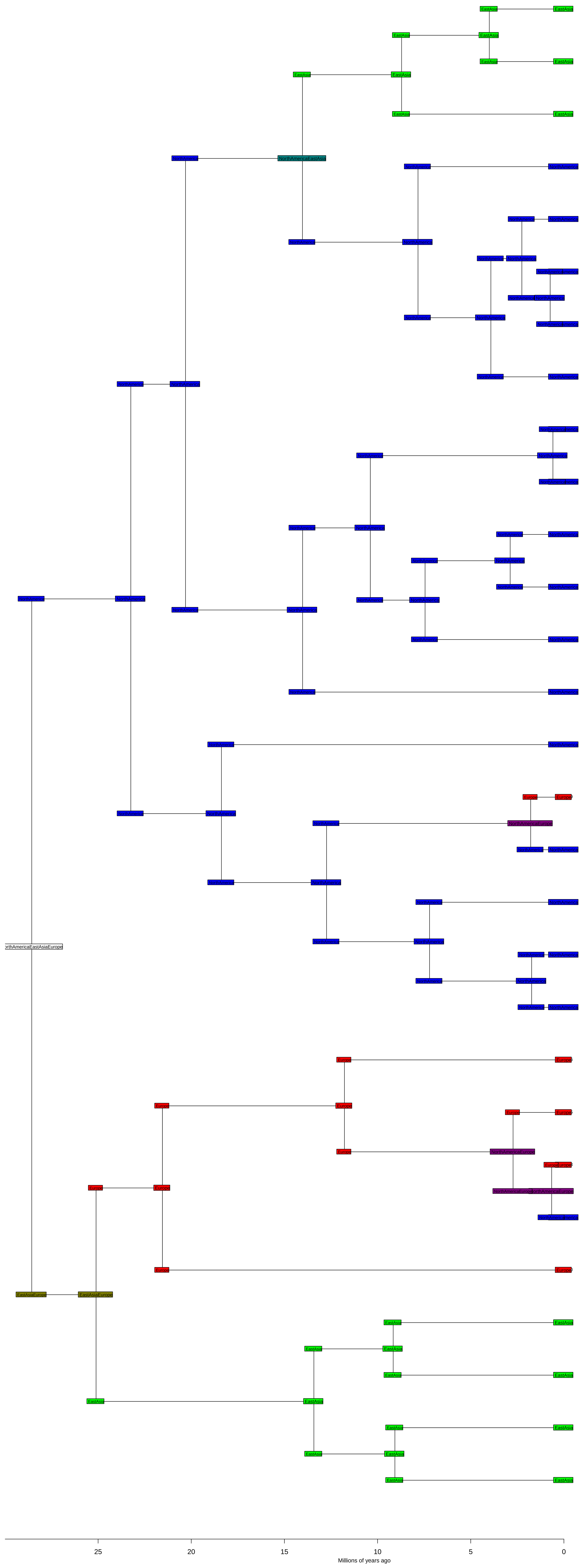

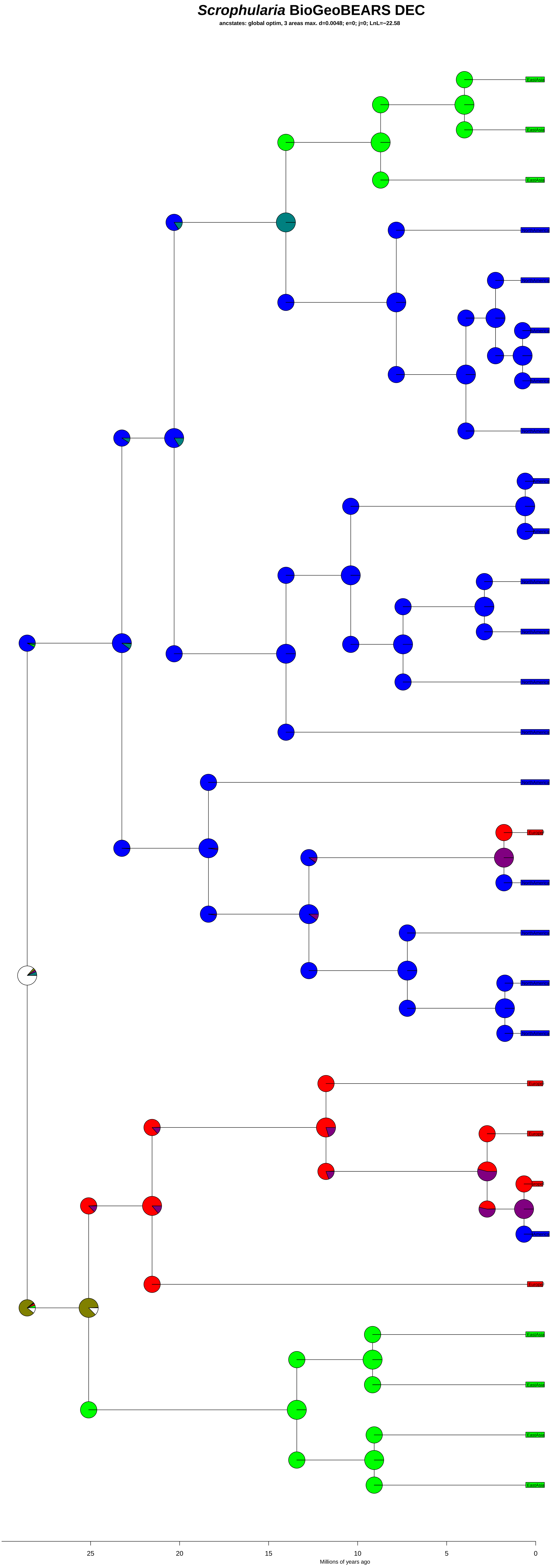
Scrophularia BioGeoBEARS DEC
ancstates: global optim, 3 areas max. d=0.0048; e=0; j=0; LnL=−22.58
EastAsia
Europe
25
20
15
10
5
0
Millions of years ago

Thuja BioGeoBEARS DEC

ancstates: global optim, 3 areas max. d=0; e=0; j=0; LnL=−5.38

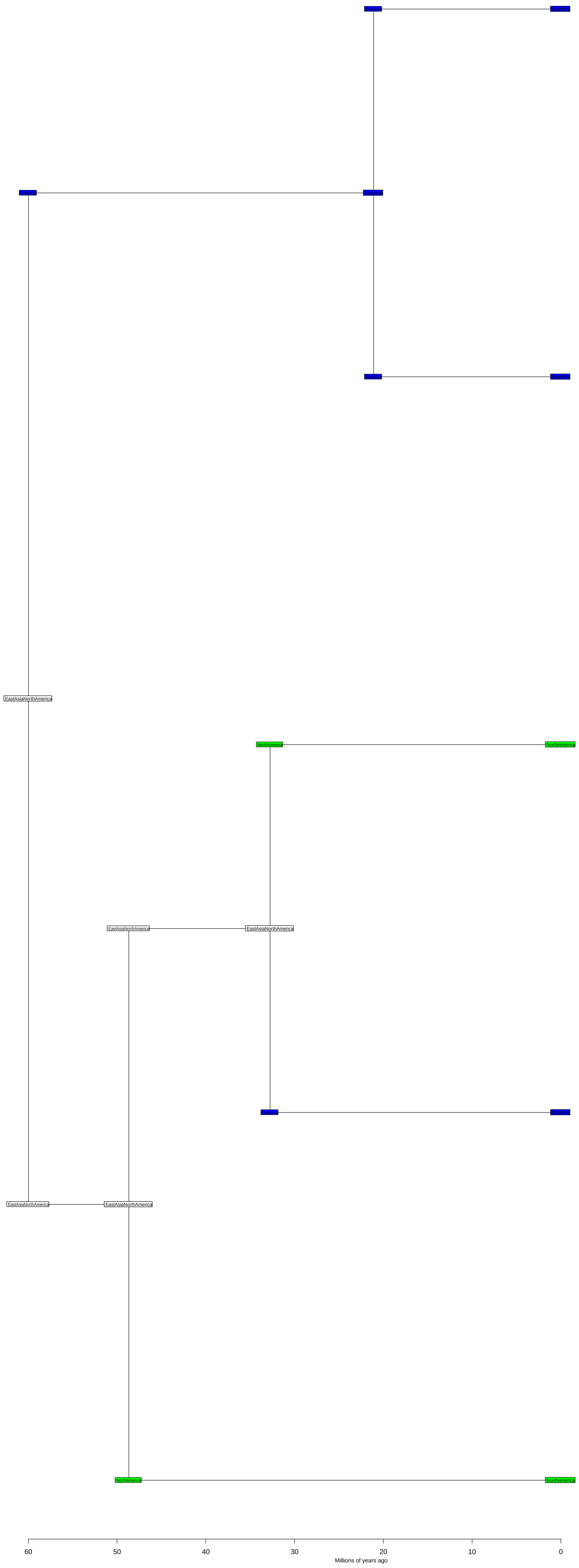

Thuja BioGeoBEARS DEC

ancstates: global optim, 3 areas max. d=0; e=0; j=0; LnL=−5.38

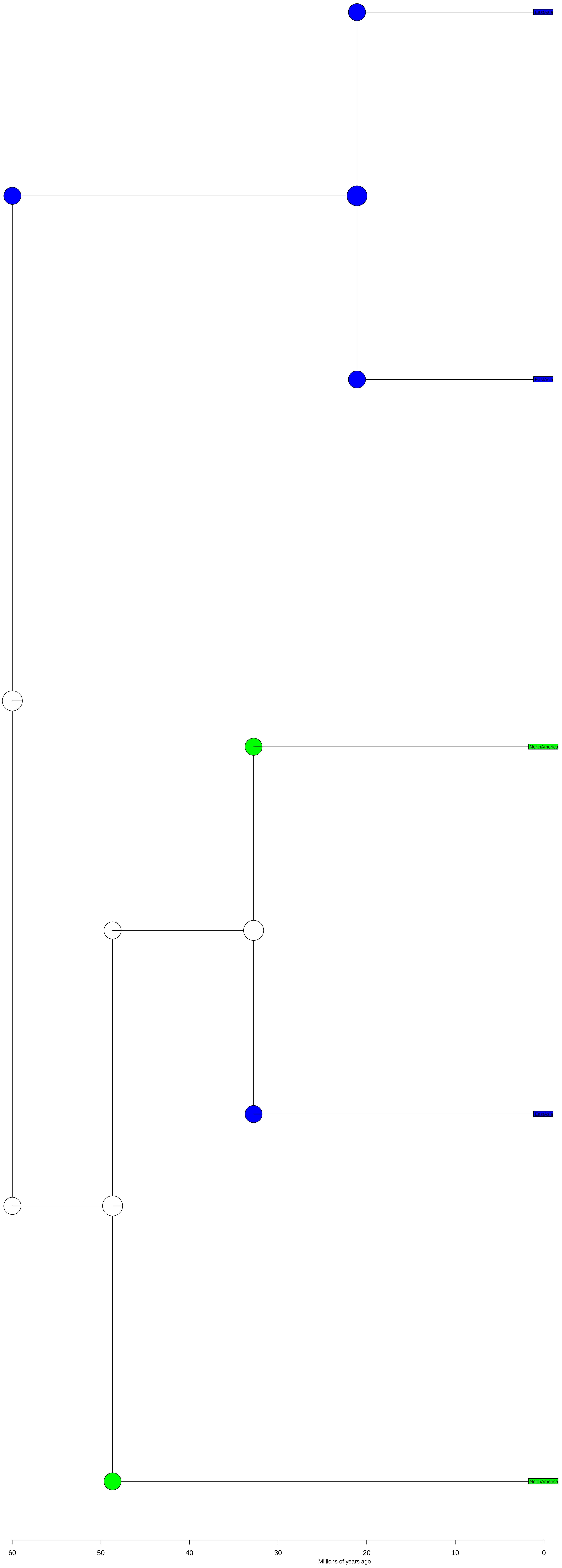

Hamamelis BioGeoBEARS DEC

ancstates: global optim, 3 areas max. d=0; e=0; j=0; LnL=−3.58

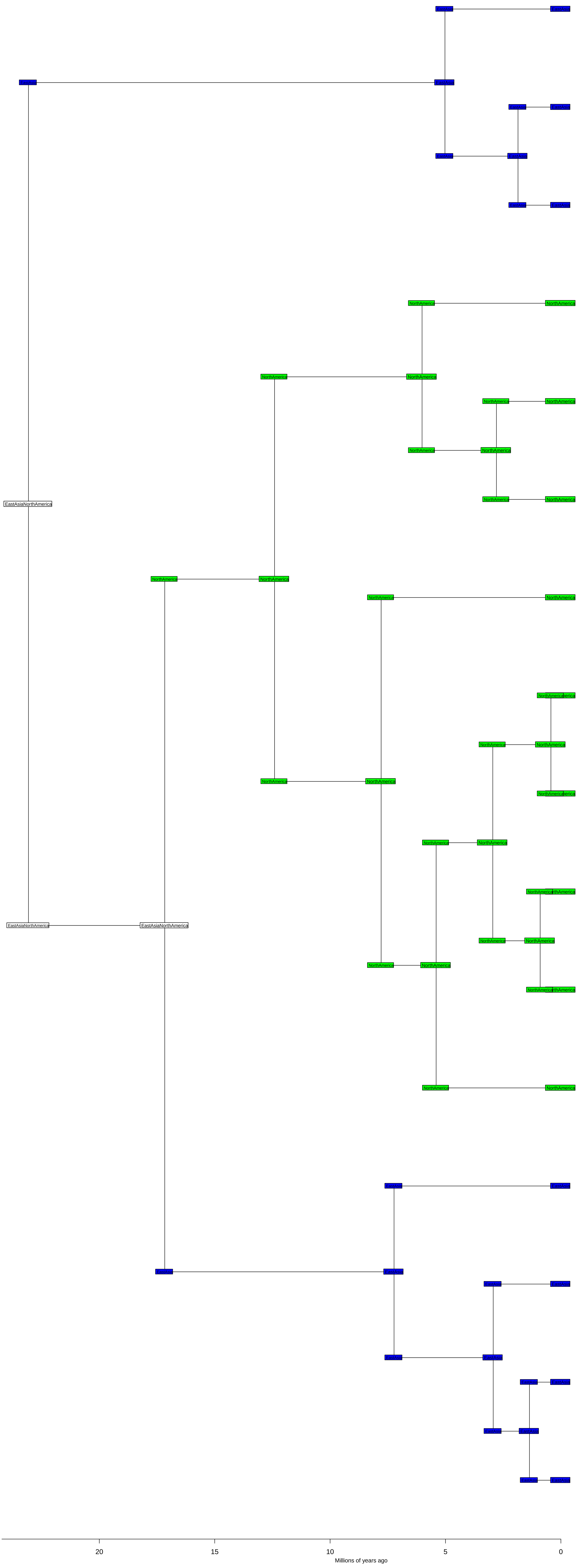

Hamamelis BioGeoBEARS DEC

ancstates: global optim, 3 areas max. d=0; e=0; j=0; LnL=−3.58

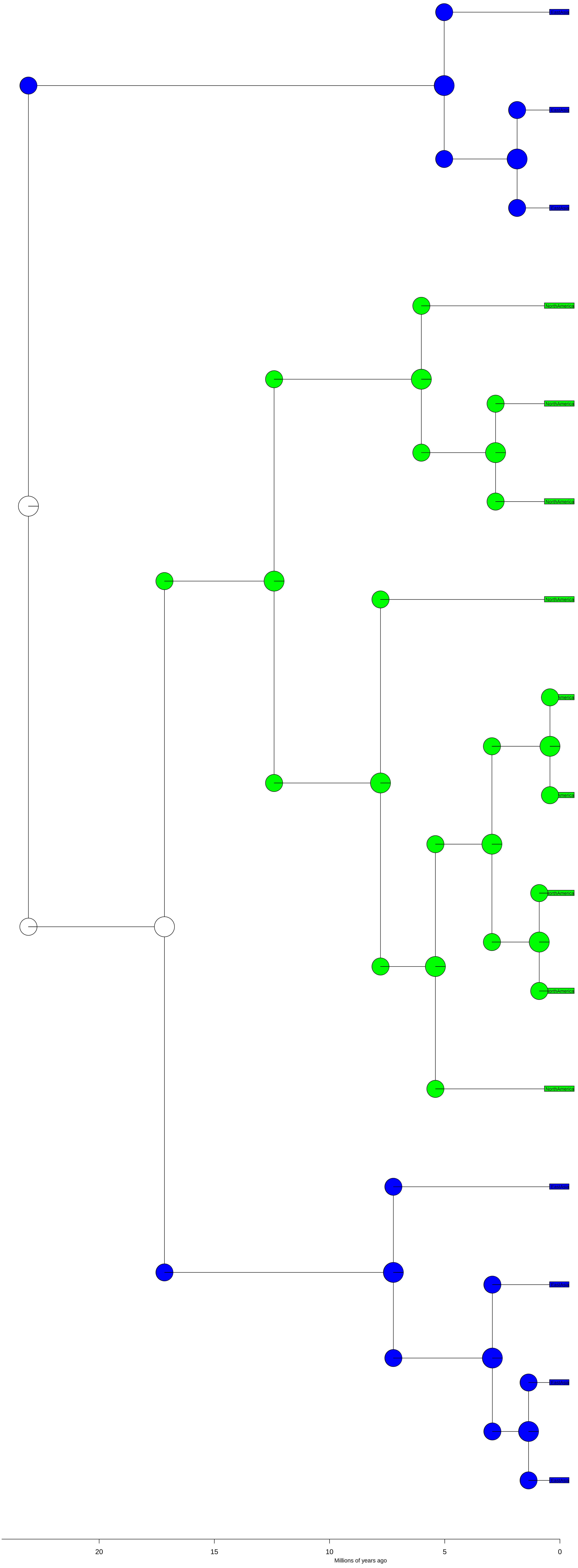

Circaea BioGeoBEARS DEC

ancstates: global optim, 3 areas max. d=0.0021; e=0; j=0; LnL=−62.04

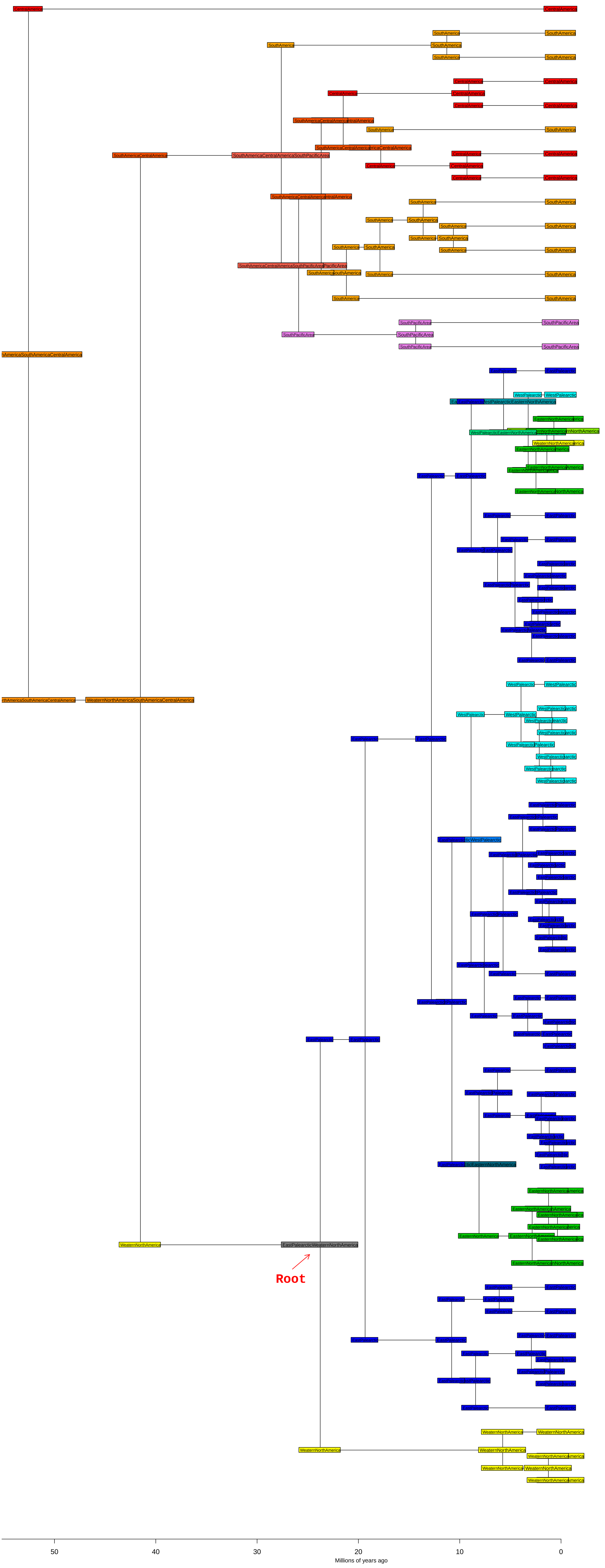

Circaea BioGeoBEARS DEC

ancstates: global optim, 3 areas max. d=0.0021; e=0; j=0; LnL=−62.04

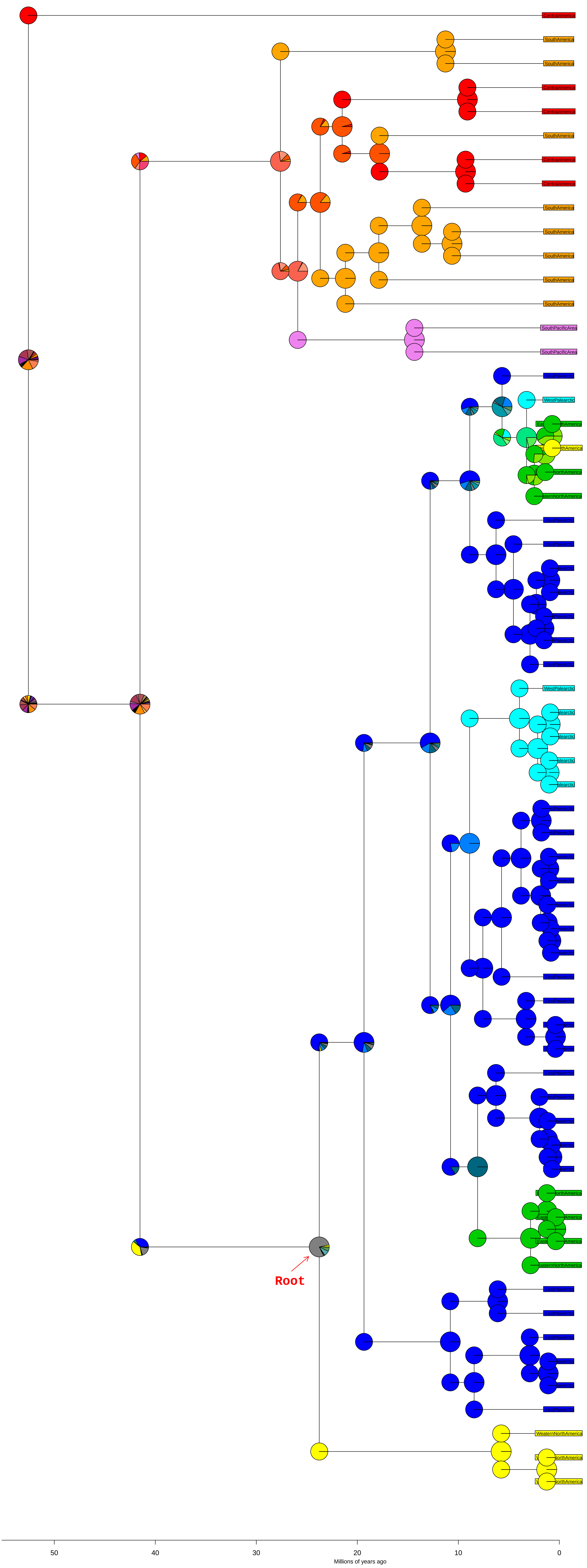

Zizania BioGeoBEARS DEC

ancstates: global optim, 3 areas max. d=0.0662; e=0; j=0; LnL=−15.39

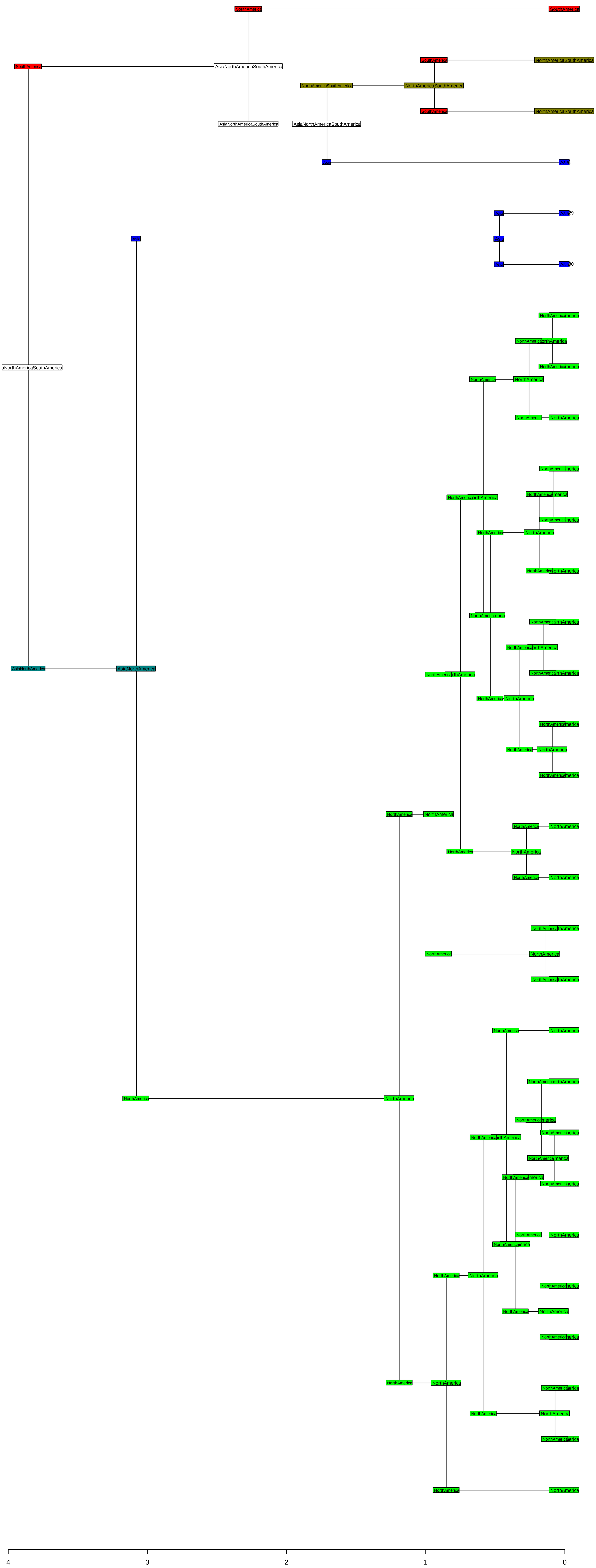

Zizania BioGeoBEARS DEC

ancstates: global optim, 3 areas max. d=0.0662; e=0; j=0; LnL=−15.39

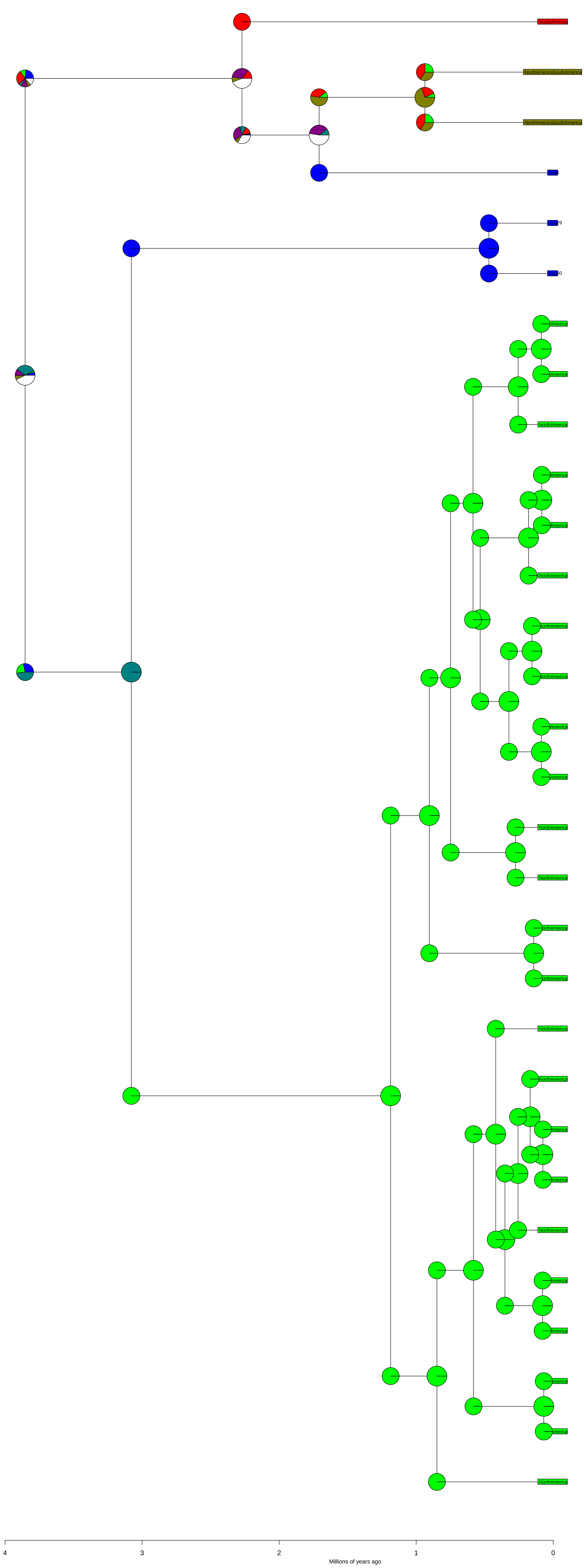

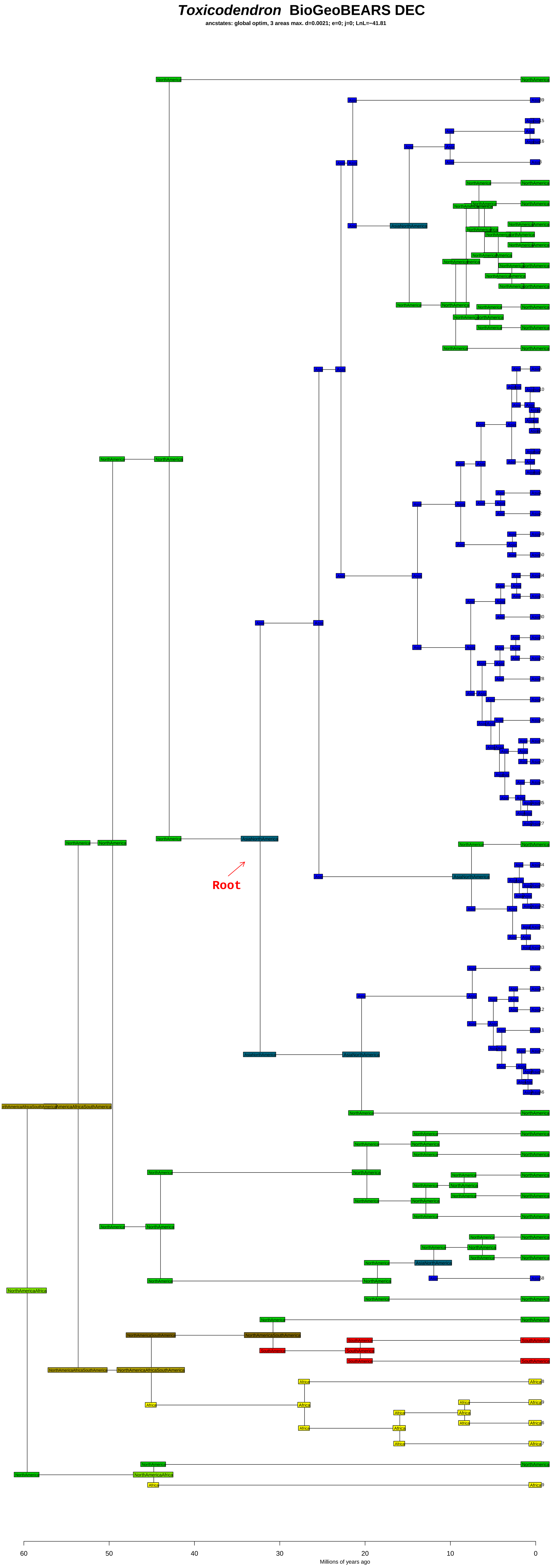
Toxicodendron BioGeoBEARS DEC
ancstates: global optim, 3 areas max. d=0.0021; e=0; j=0; LnL=-41.81
Root
60
50
40
30
20
10
0
Millions of years ago

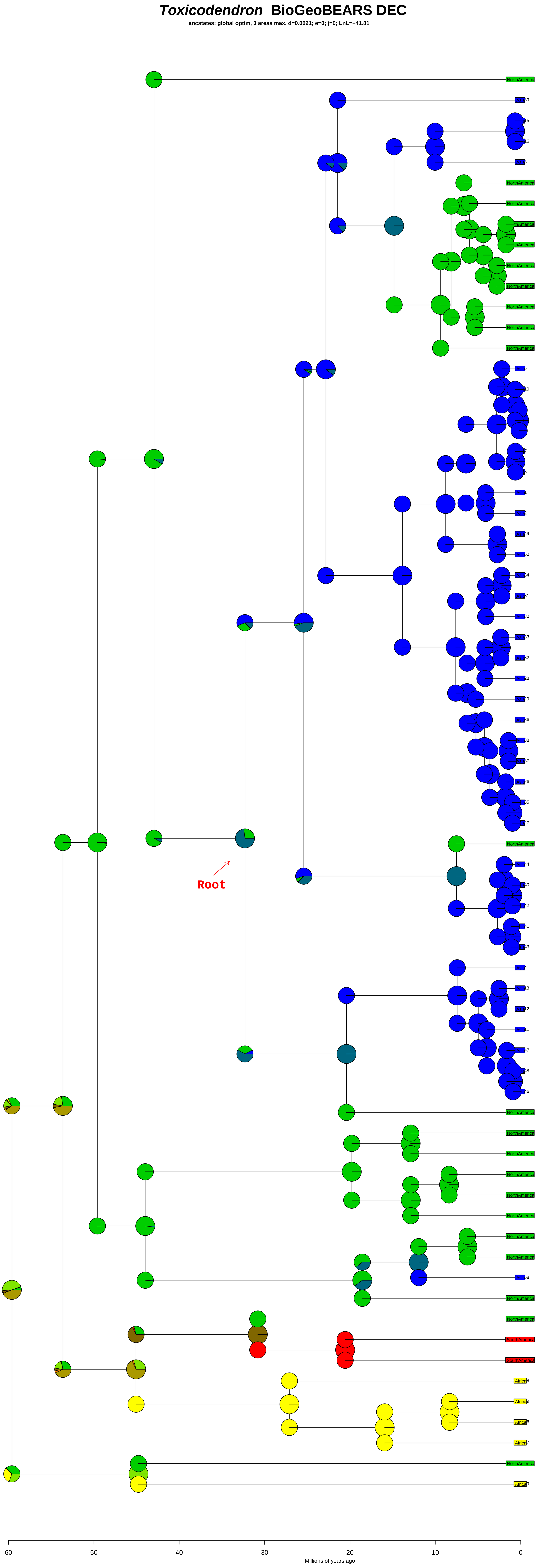
Toxicodendron BioGeoBEARS DEC
ancstates: global optim, 3 areas max. d=0.0021; e=0; j=0; LnL=−41.81
Root
NorthAmerica
SouthAmerica
Africa
60
50
40
30
20
10
0
Millions of years ago

Phryma BioGeoBEARS DEC

ancstates: global optim, 3 areas max. d=0; e=0; j=0; LnL=−1.79

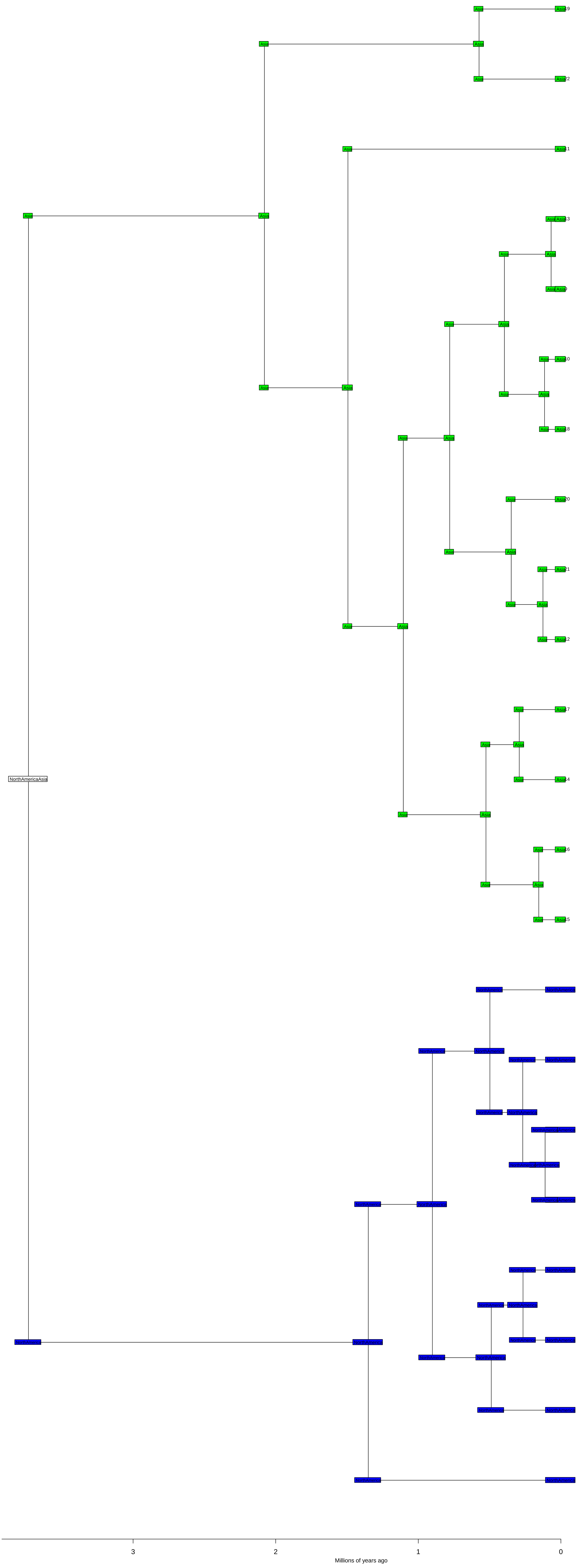

Phryma BioGeoBEARS DEC

ancstates: global optim, 3 areas max. d=0; e=0; j=0; LnL=−1.79

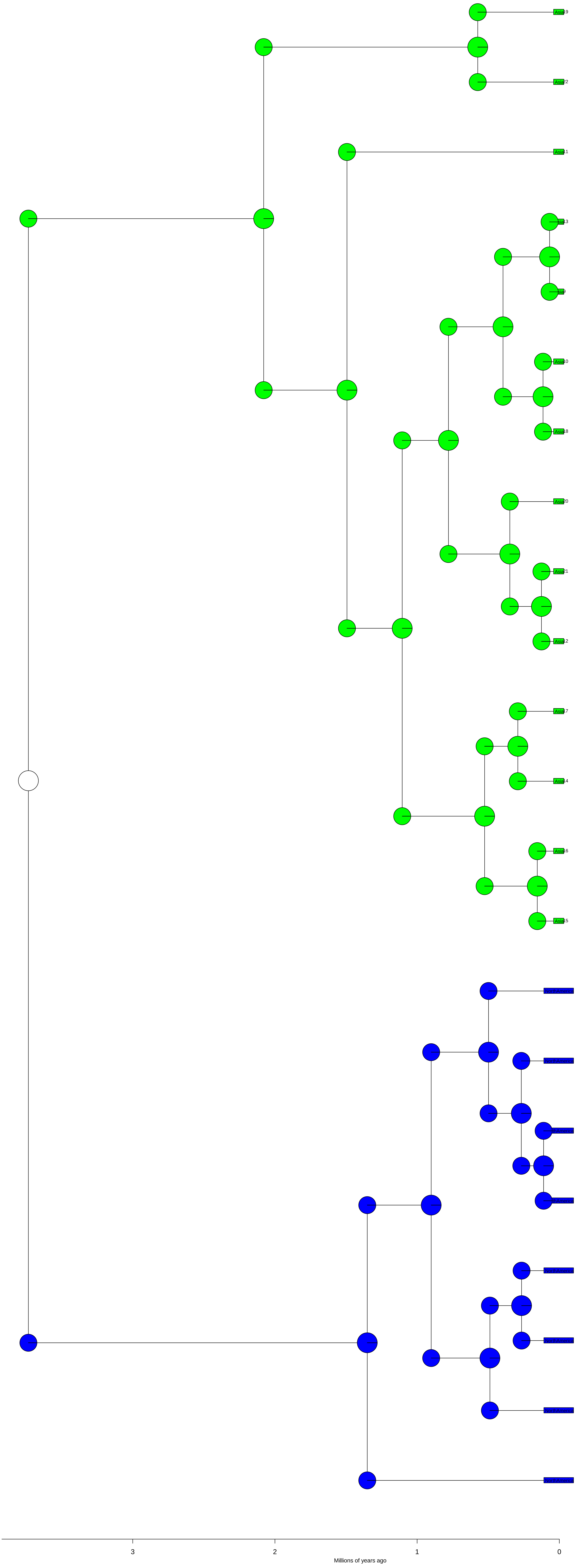

Mitchelleae BioGeoBEARS DEC

ancstates: global optim, 3 areas max. d=0.0084; e=0; j=0; LnL=−12.45

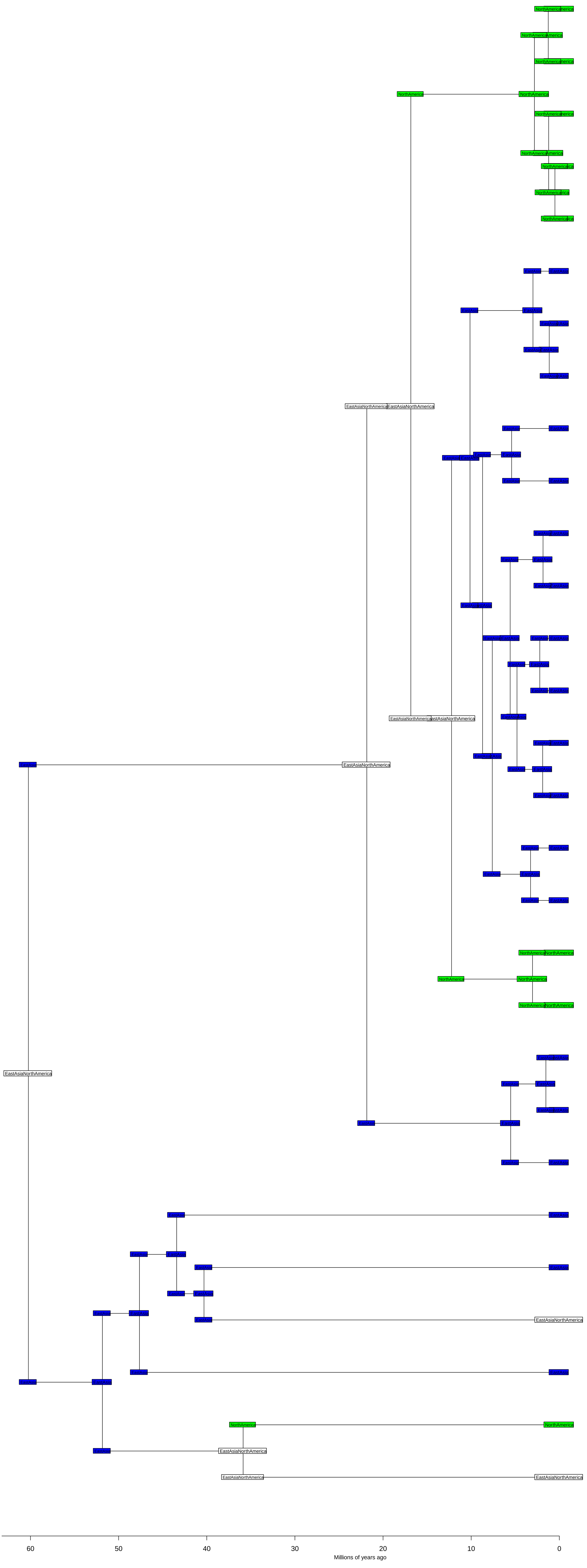

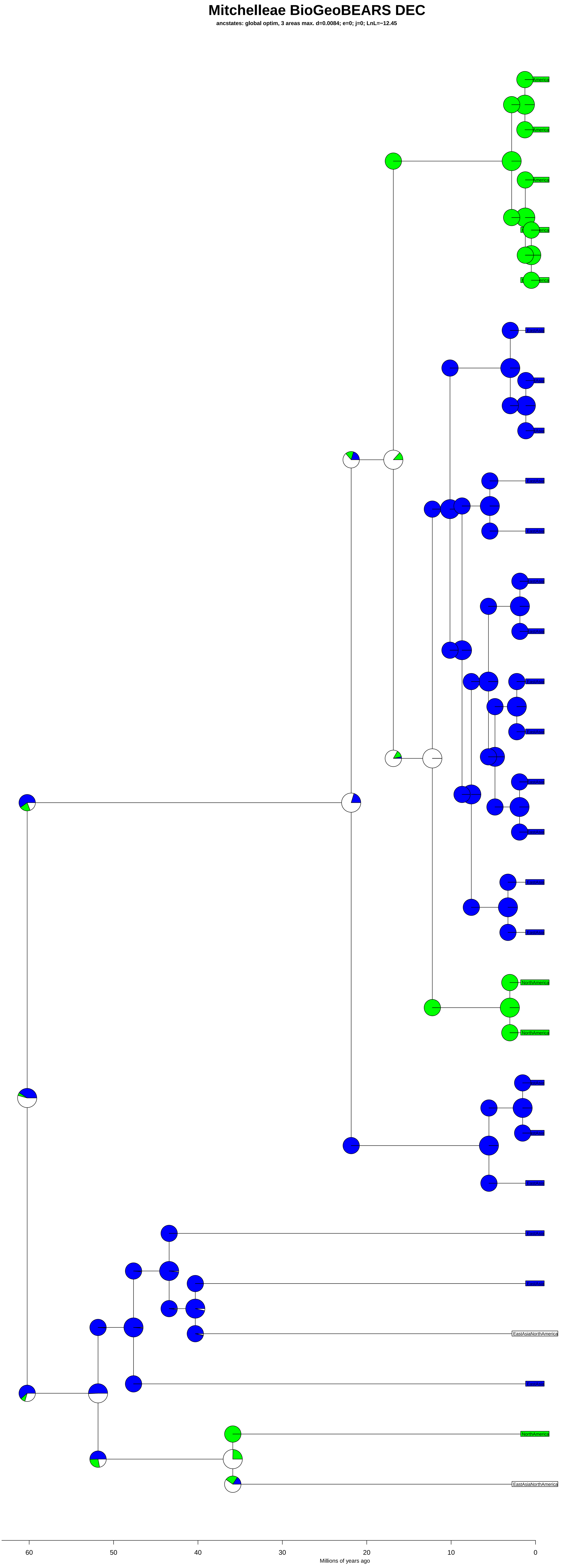
Mitchelleae BioGeoBEARS DEC
ancstates: global optim, 3 areas max. d=0.0084; e=0; j=0; LnL=−12.45
NorthAmerica
NorthAmerica
EastAsiaNorthAmerica
NorthAmerica
EastAsiaNorthAmerica
60
50
40
30
20
10
0
Millions of years ago

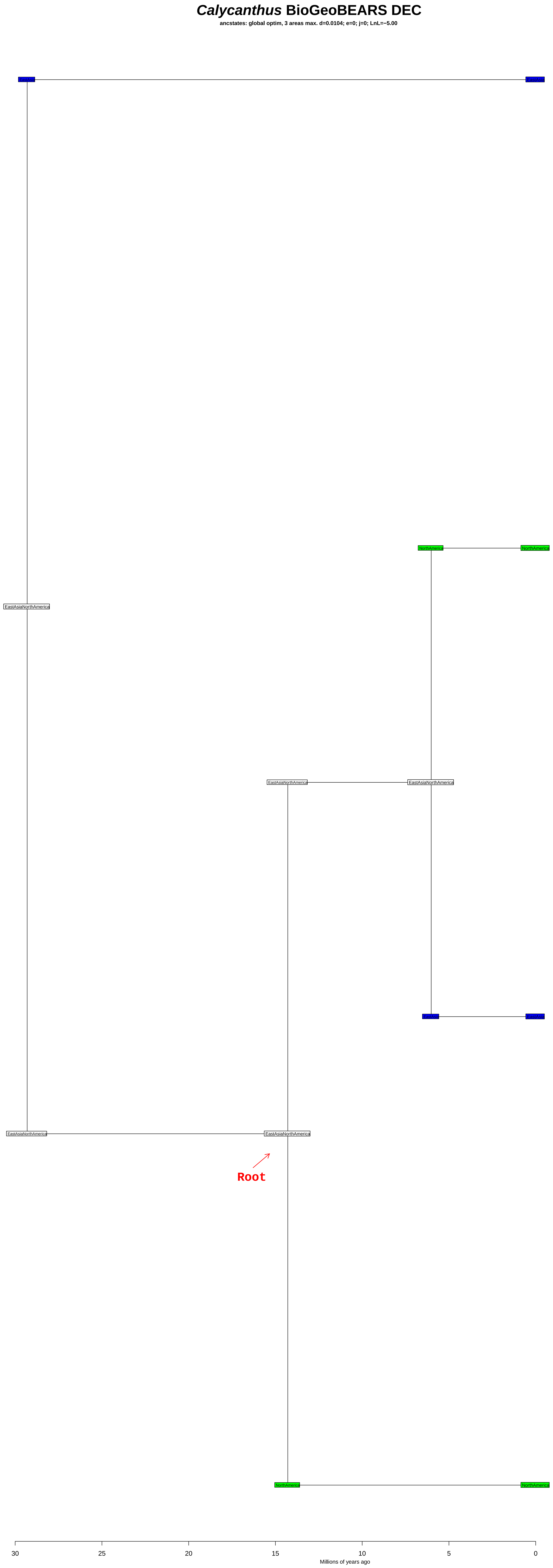

Calycanthus BioGeoBEARS DEC
ancstates: global optim, 3 areas max. d=0.0104; e=0; j=0; LnL=−5.00
EastAsia
EastAsia
NorthAmerica
NorthAmerica
EastAsiaNorthAmerica
EastAsiaNorthAmerica
EastAsiaNorthAmerica
EastAsia
EastAsia
EastAsiaNorthAmerica
EastAsiaNorthAmerica
Root
NorthAmerica
NorthAmerica
30
25
20
15
10
5
0
Millions of years ago

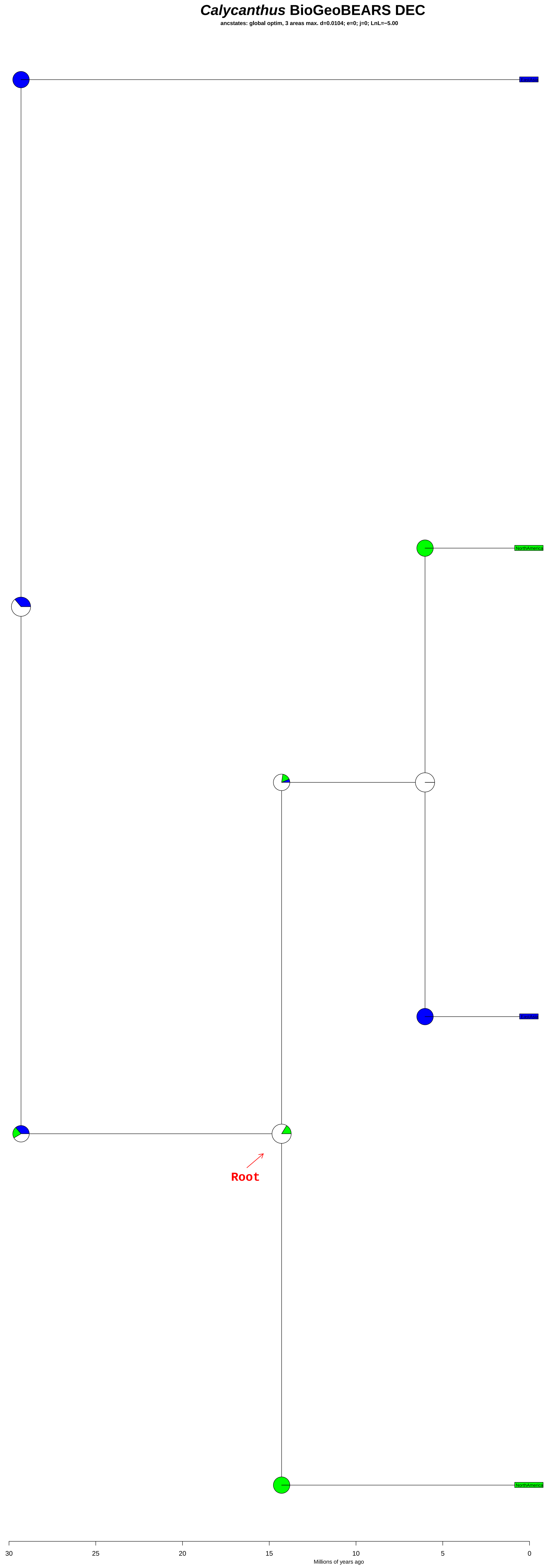

Calycanthus BioGeoBEARS DEC
ancstates: global optim, 3 areas max. d=0.0104; e=0; j=0; LnL=−5.00
EastAsia
NorthAmerica
EastAsia
Root
NorthAmerica
30
25
20
15
10
5
0
Millions of years ago

Lathyrus BioGeoBEARS DEC

ancstates: global optim, 3 areas max. d=0.0148; e=0.0036; j=0; LnL=−60.26

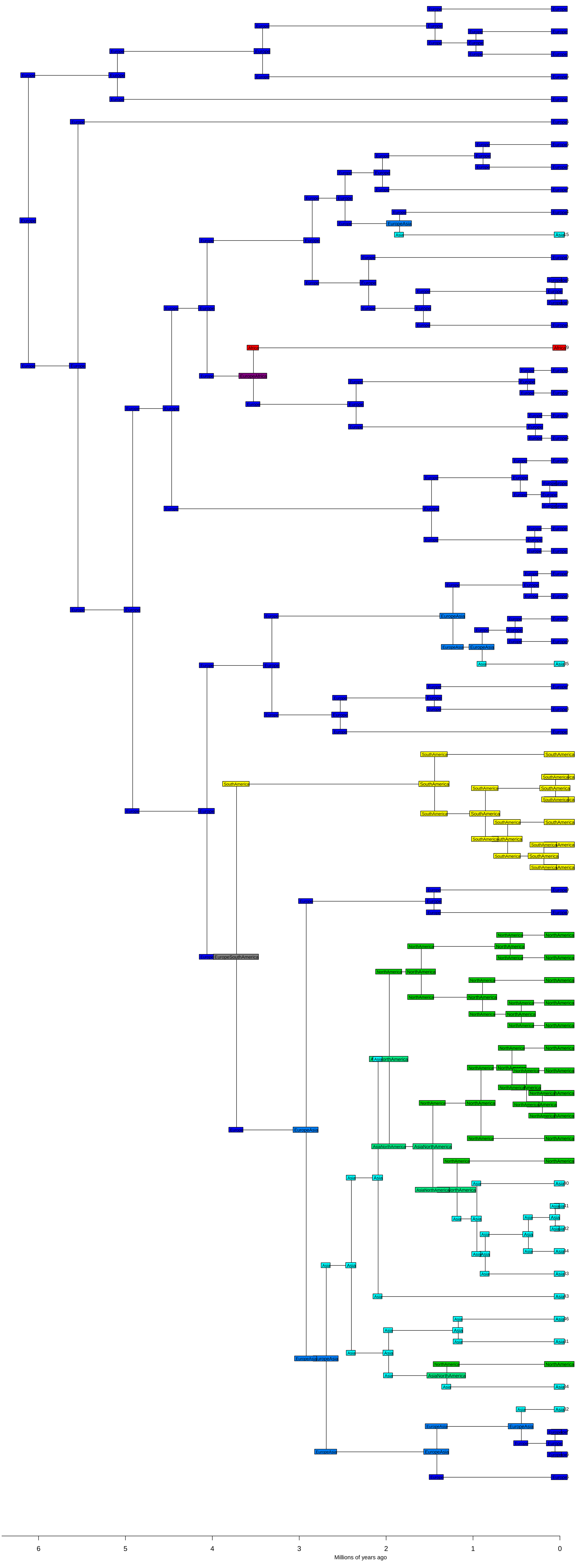

***Lathyrus* BioGeoBEARS DEC**

ancstates: global optim, 3 areas max. d=0.0148; e=0.0036; j=0; LnL=−60.26

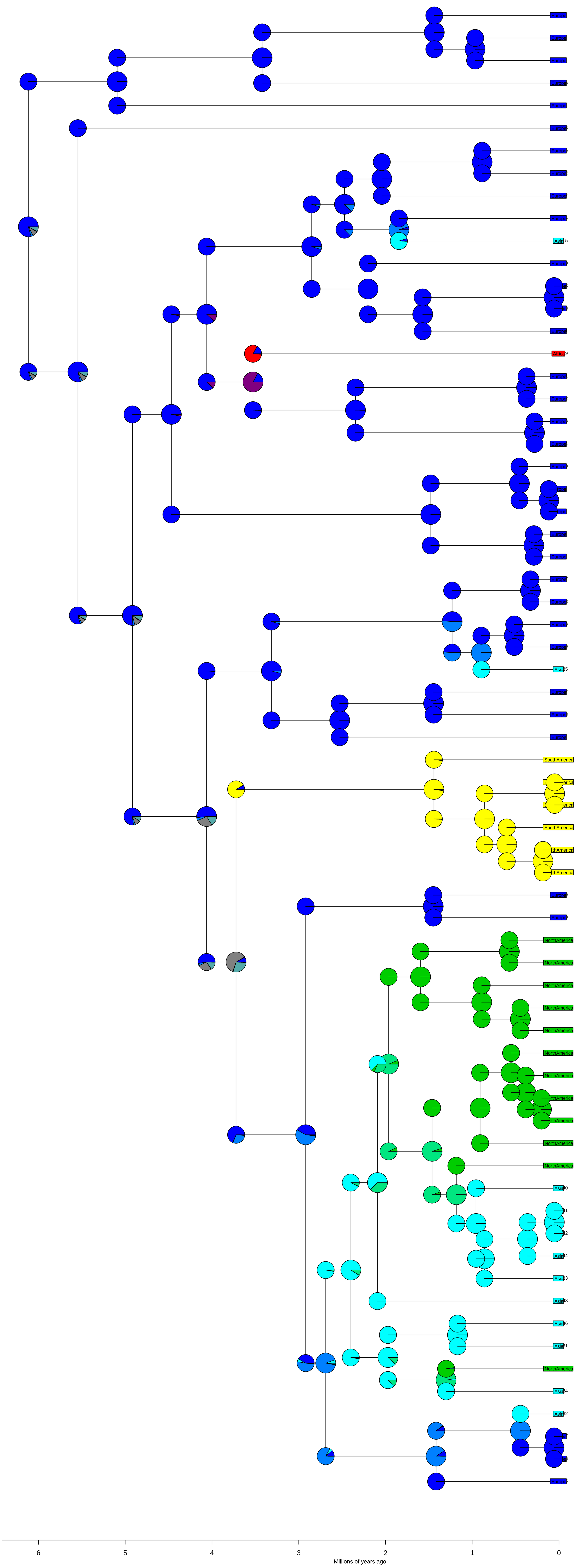

Taxus BioGeoBEARS DEC

ancstates: global optim, 3 areas max. d=0.0353; e=0; j=0; LnL=−17.05

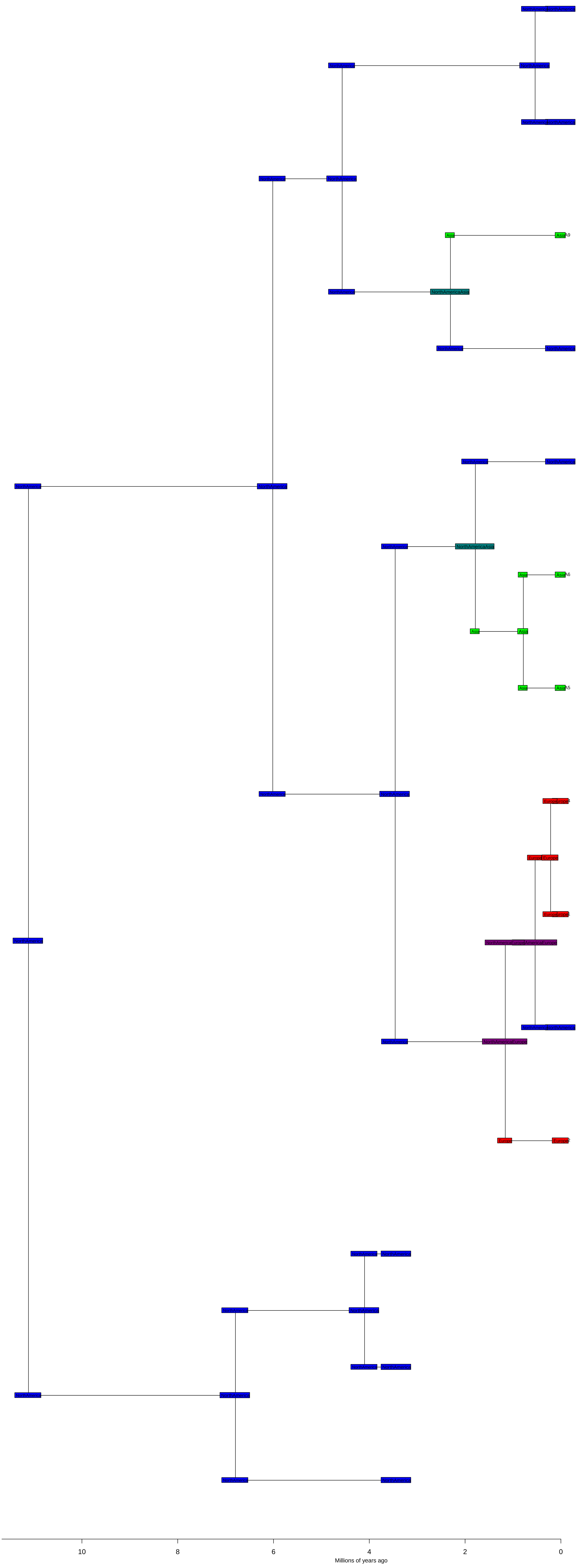

Taxus BioGeoBEARS DEC

ancstates: global optim, 3 areas max. d=0.0353; e=0; j=0; LnL=−17.05

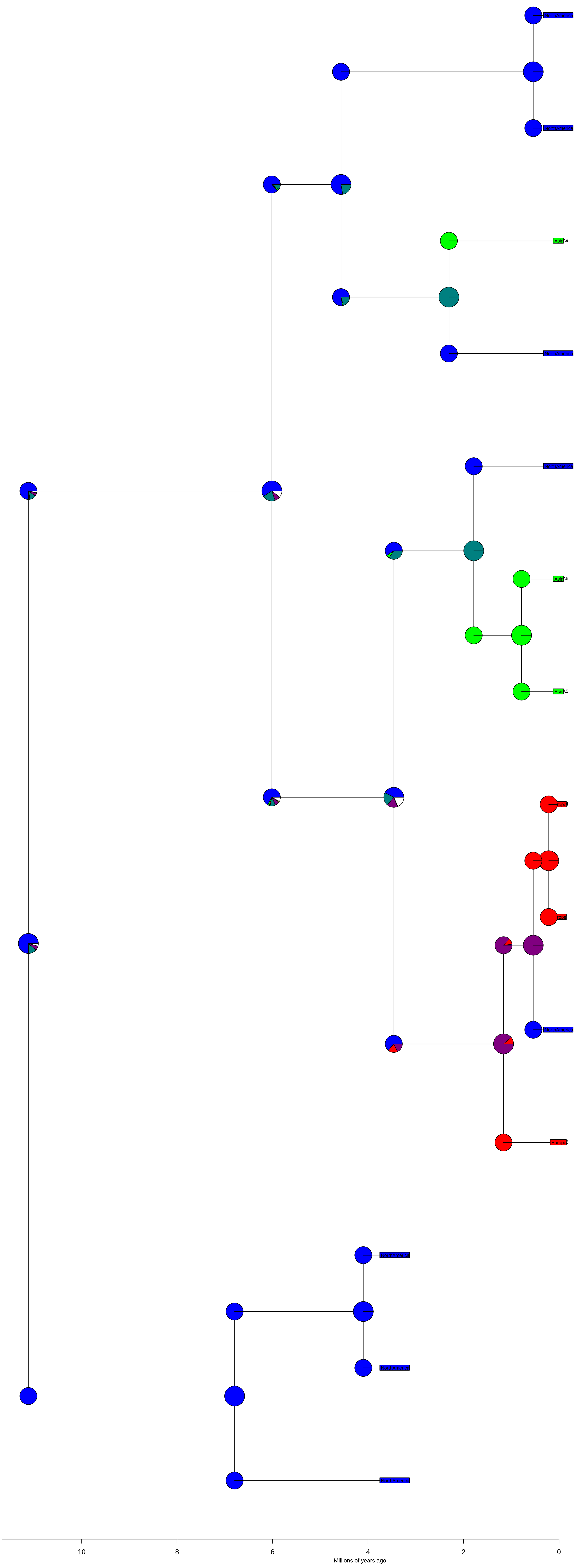

Thuja BioGeoBEARS DEC

ancstates: global optim, 2 areas max. d=0.0134; e=0.0051; j=0; LnL=−10.34

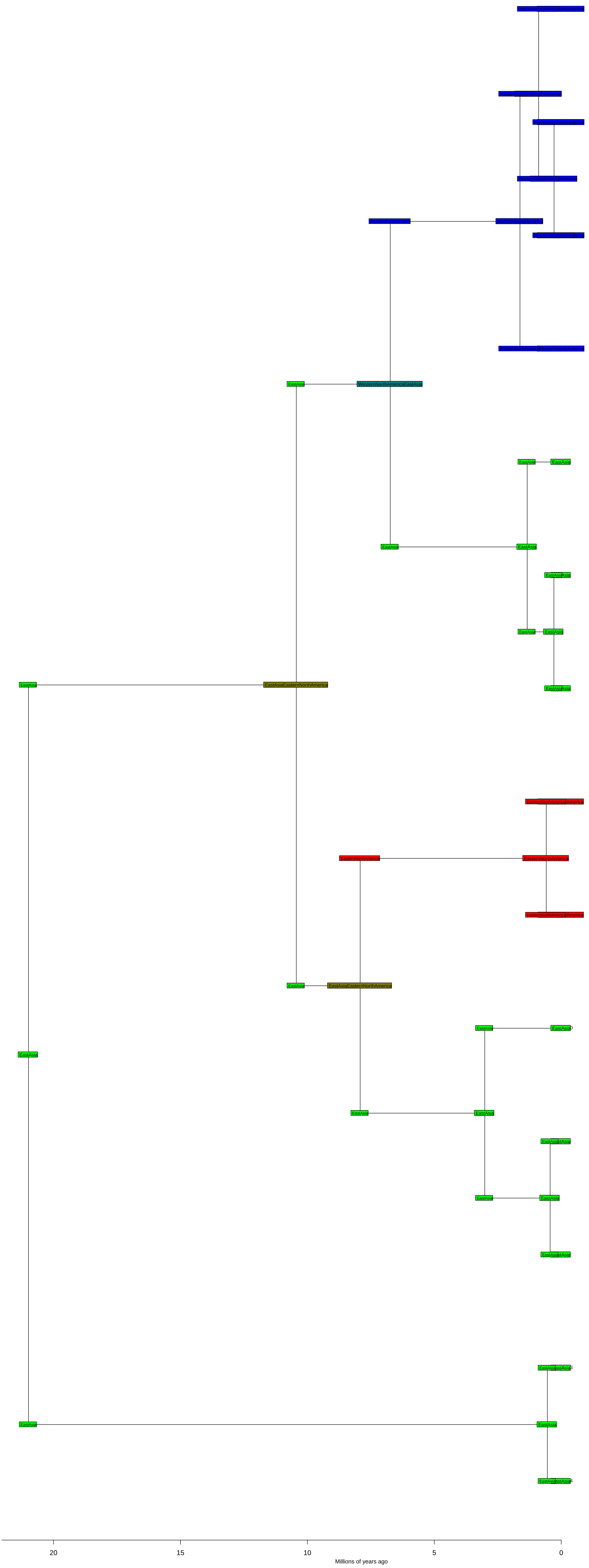

Thuja BioGeoBEARS DEC

ancstates: global optim, 2 areas max. d=0.0134; e=0.0051; j=0; LnL=−10.34

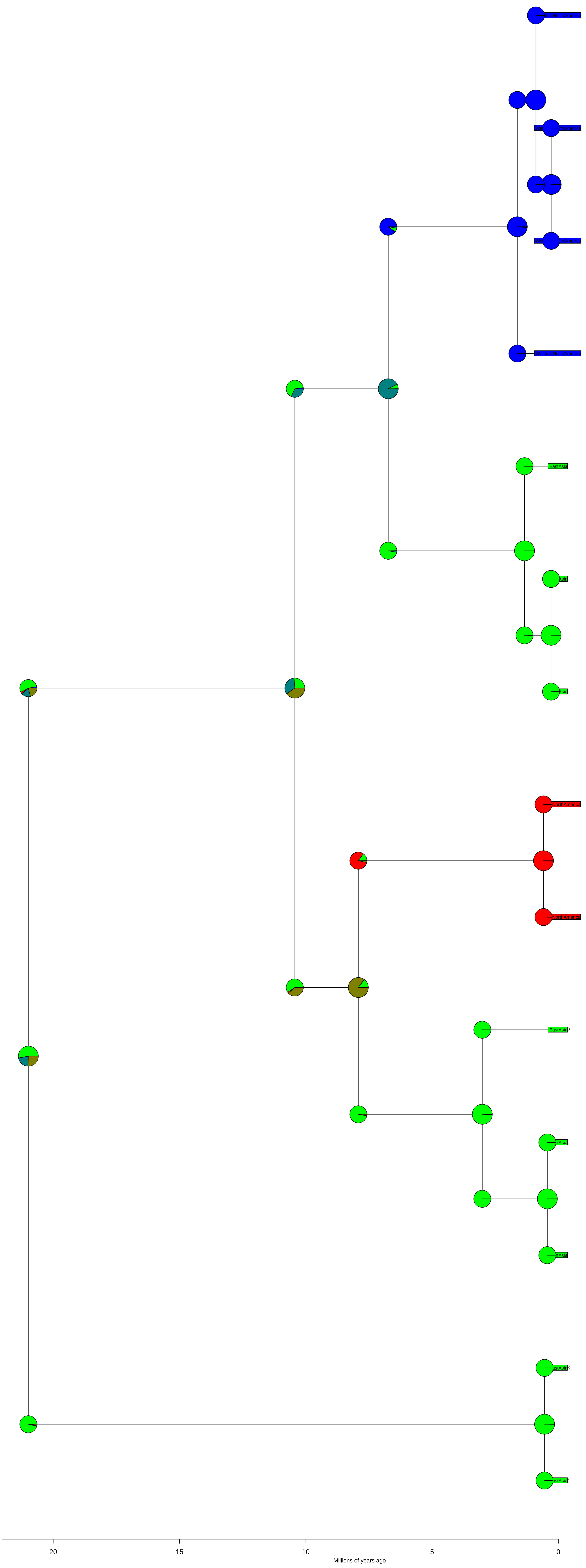

Rhus BioGeoBEARS DEC

ancstates: global optim, 2 areas max. d=0.0015; e=0; j=0; LnL=−21.30

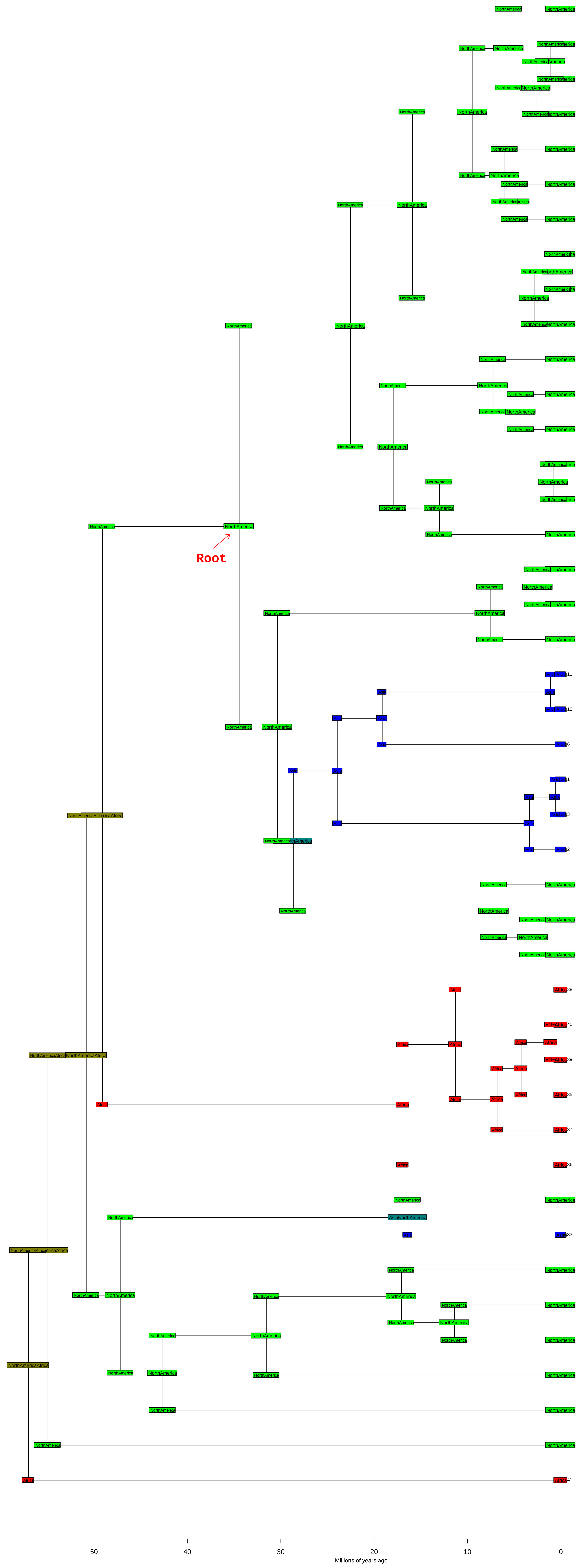

Rhus BioGeoBEARS DEC

ancstates: global optim, 2 areas max. d=0.0015; e=0; j=0; LnL=−21.30

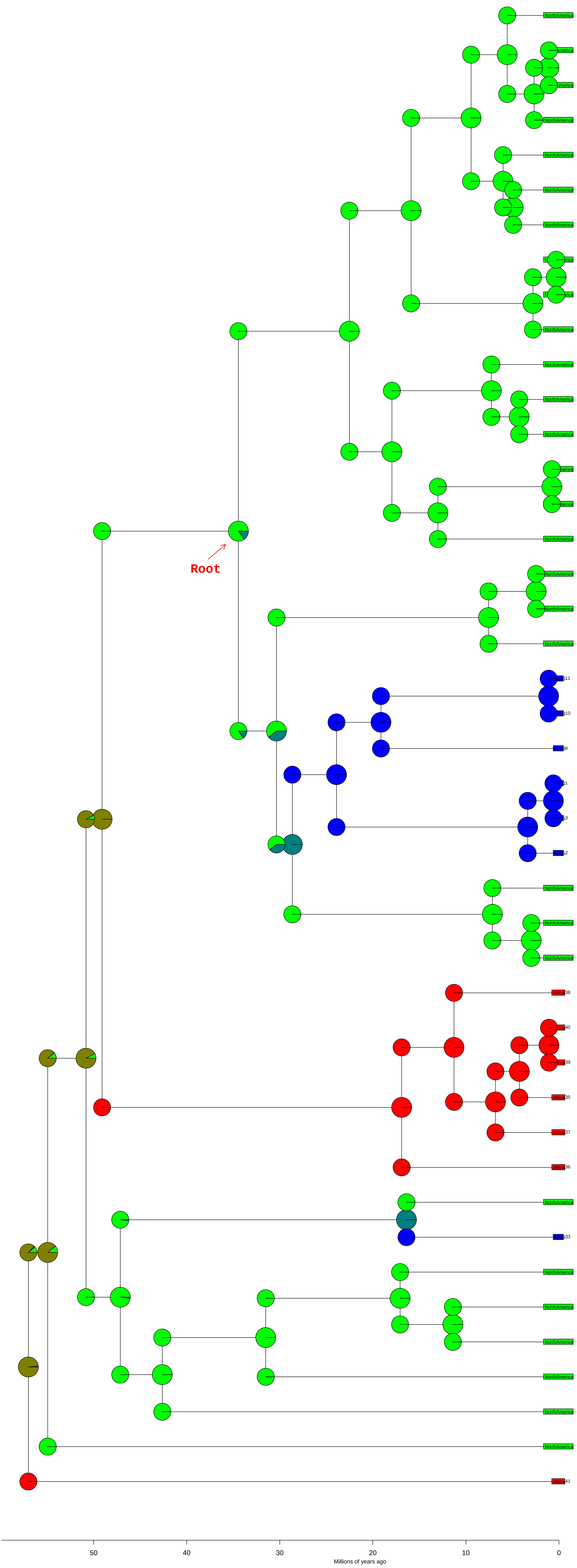

Pseudotsuga BioGeoBEARS DEC

ancstates: global optim, 3 areas max. d=0; e=0; j=0; LnL=−1.79

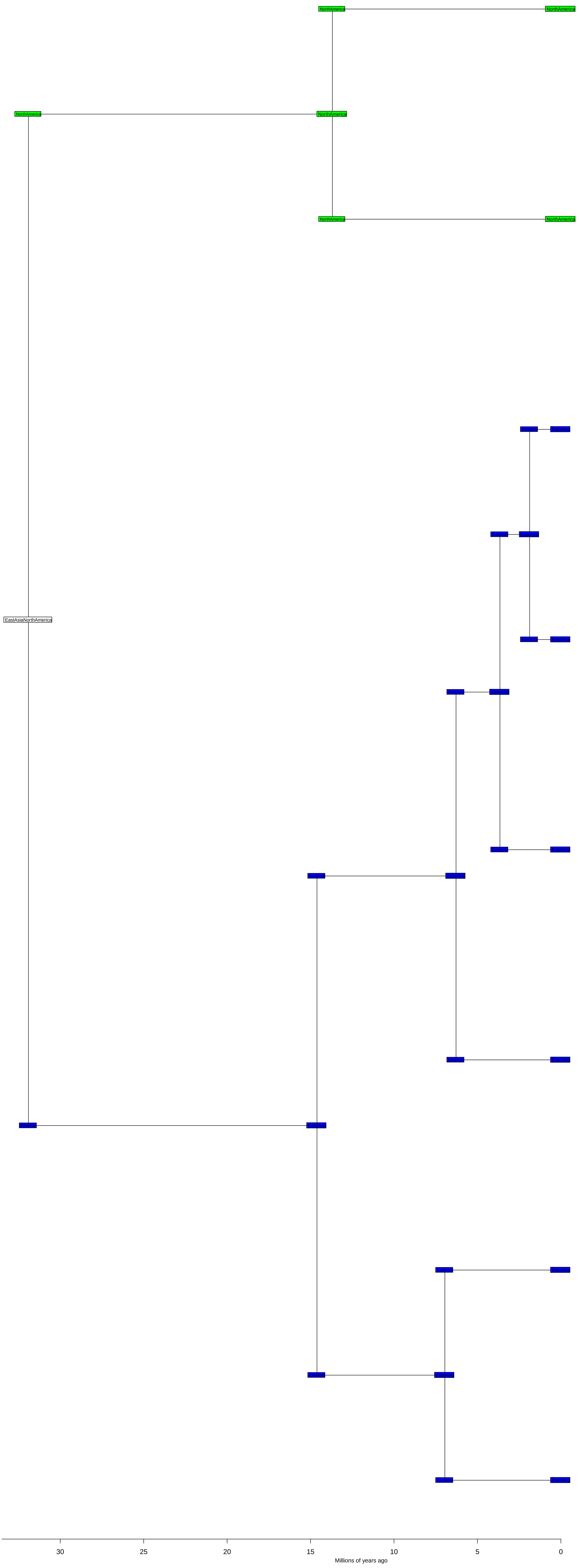

Pseudotsuga BioGeoBEARS DEC

ancstates: global optim, 3 areas max. d=0; e=0; j=0; LnL=−1.79

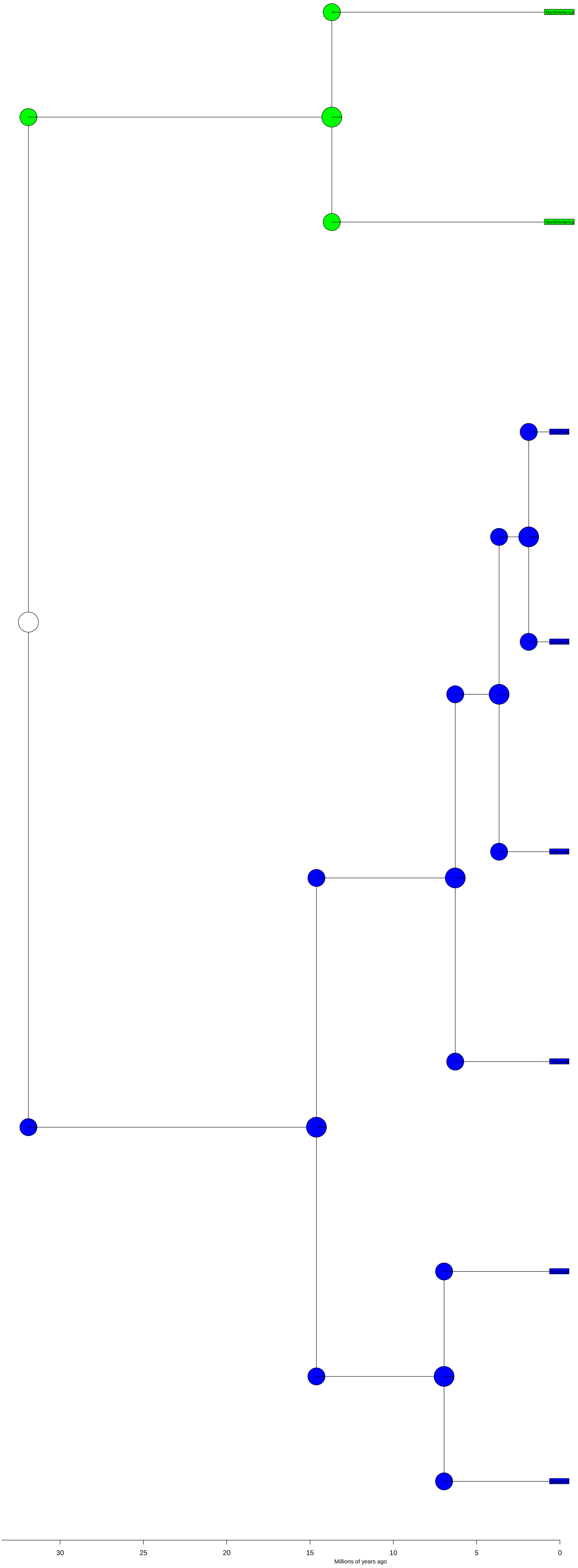

Orontioideae BioGeoBEARS DEC

ancstates: global optim, 3 areas max. d=0.016; e=0; j=0; LnL=−12.46

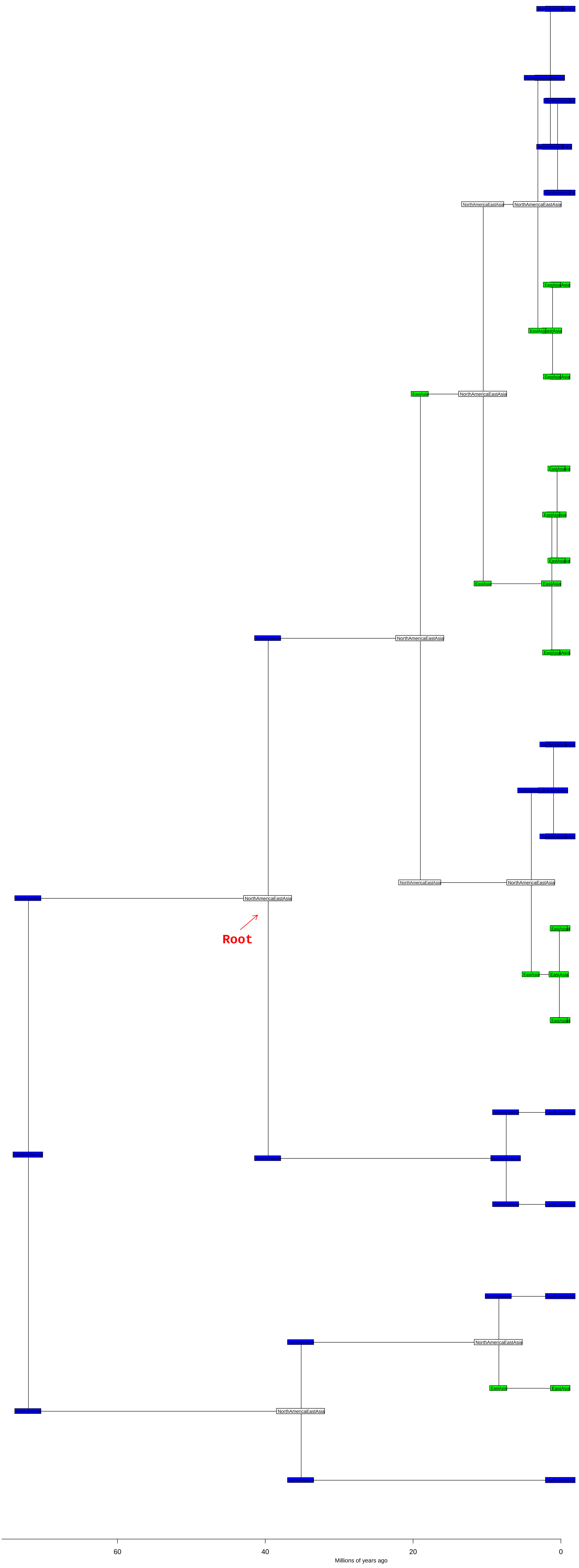

Orontioideae BioGeoBEARS DEC

ancstates: global optim, 3 areas max. d=0.016; e=0; j=0; LnL=−12.46

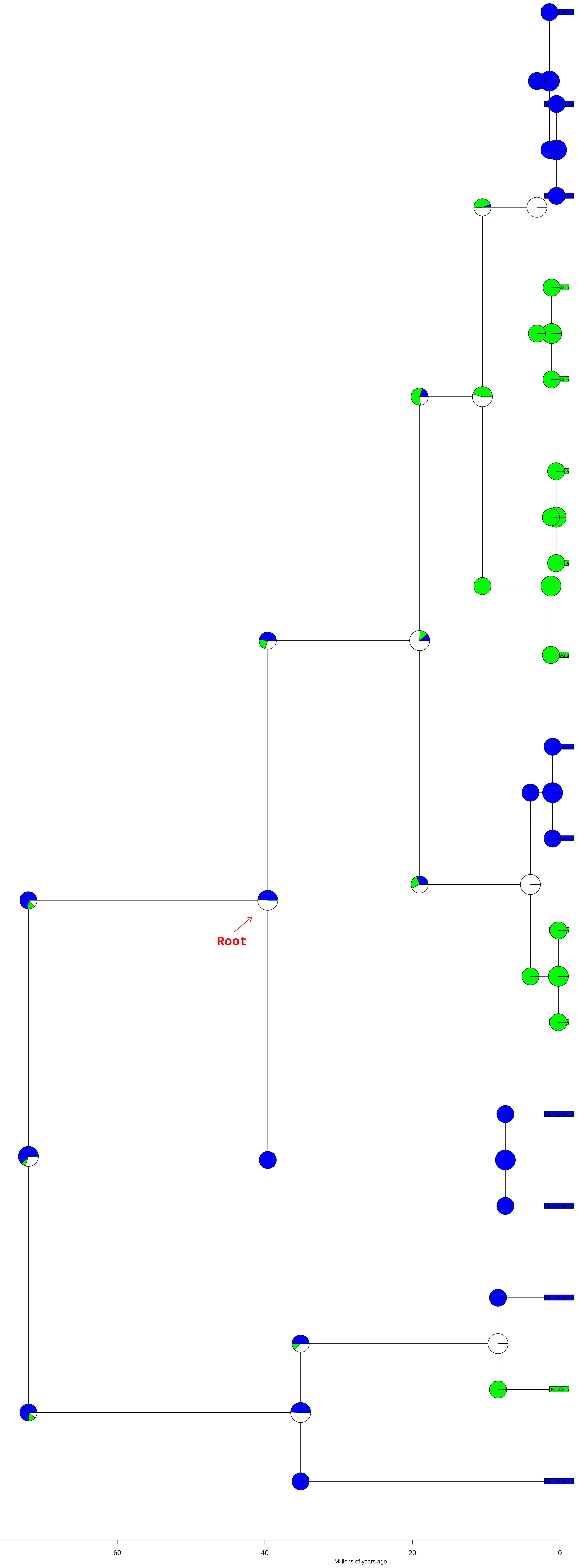

Kelloggia BioGeoBEARS DEC

ancstates: global optim, 3 areas max. d=0; e=0; j=0; LnL=−1.79

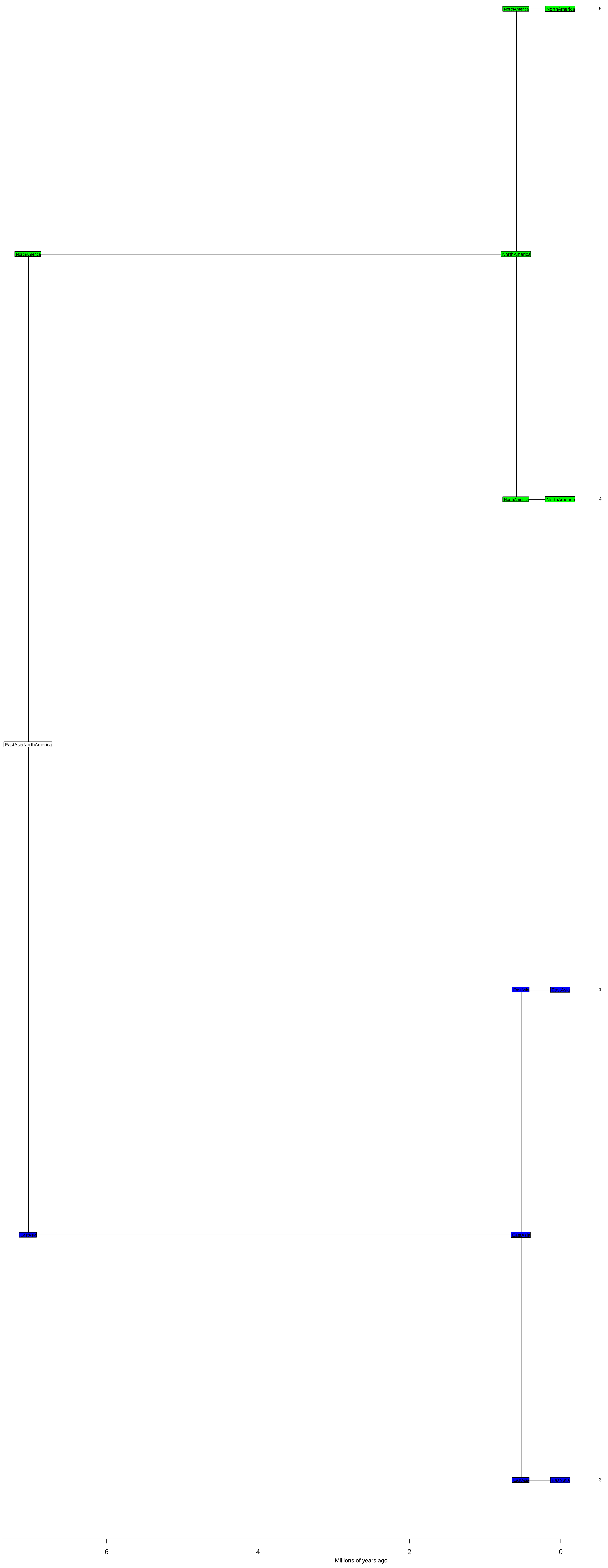

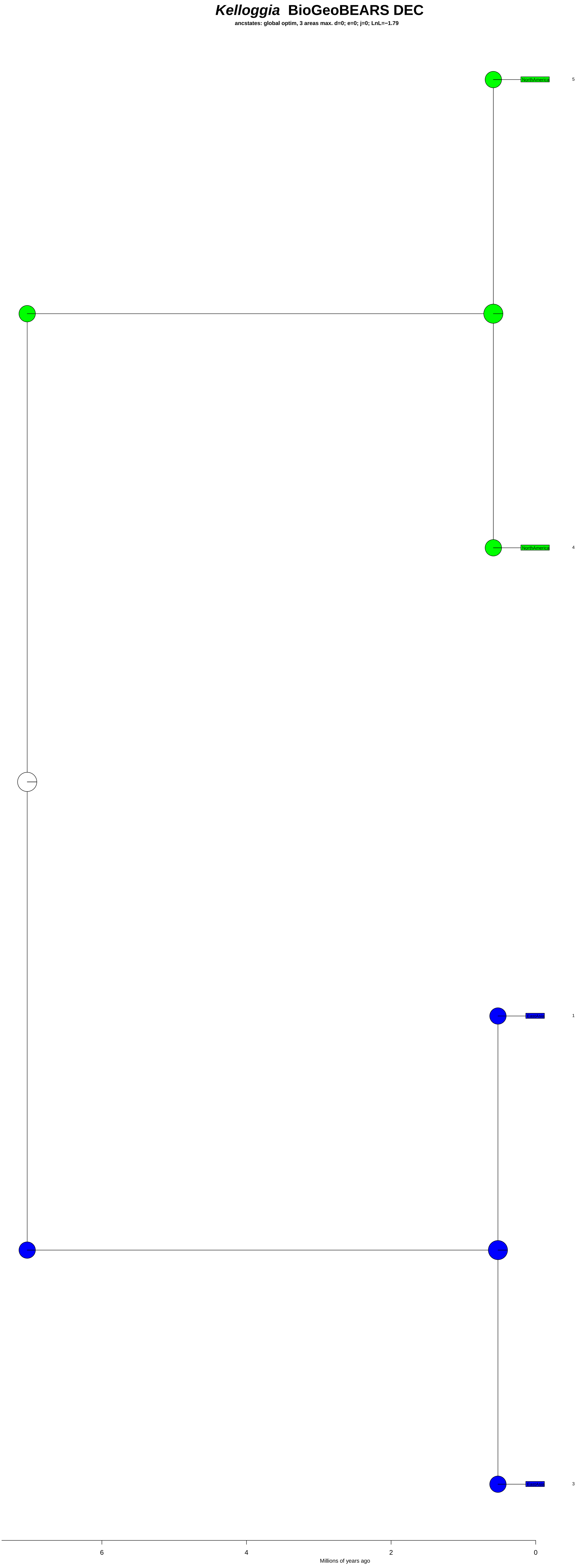
Kelloggia BioGeoBEARS DEC
ancstates: global optim, 3 areas max. d=0; e=0; j=0; LnL=−1.79
NorthAmerica
5
NorthAmerica
4
1
3
6
4
2
0
Millions of years ago

Aesculus BioGeoBEARS DEC

ancstates: global optim, 3 areas max. d=0.0011; e=1e−04; j=0; LnL=−33.01

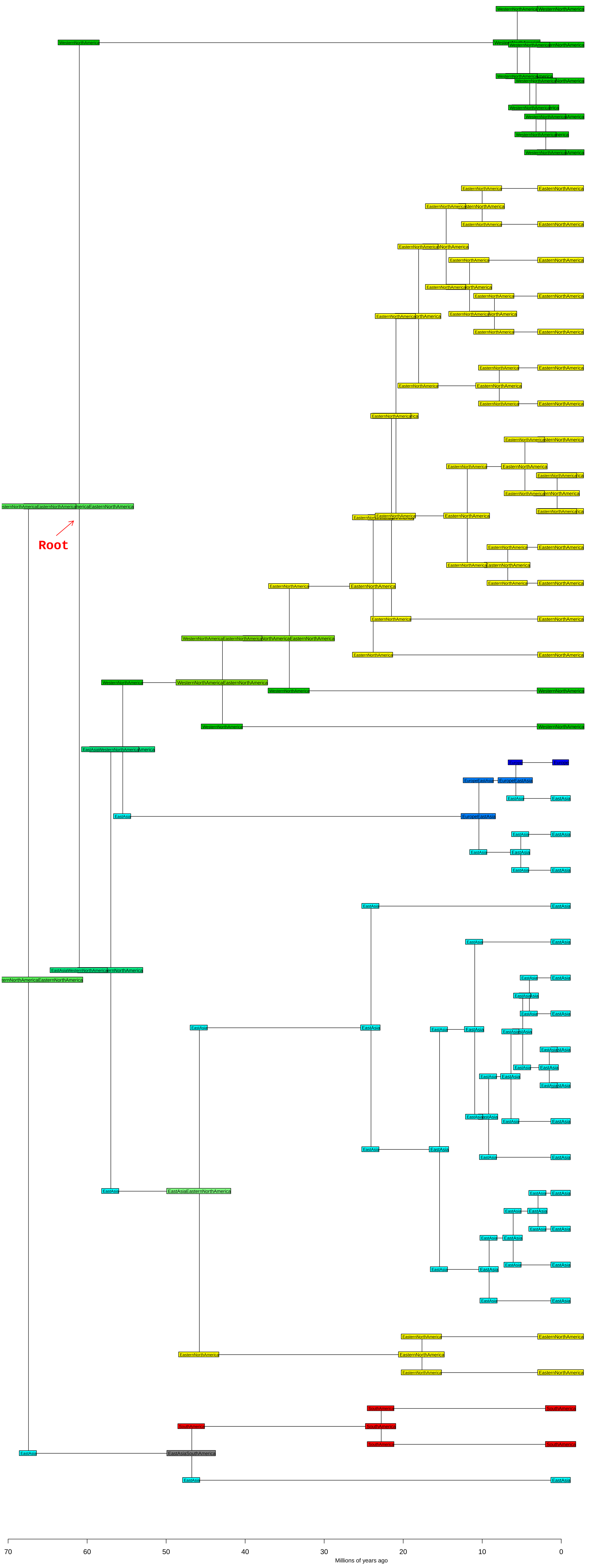

Aesculus BioGeoBEARS DEC

ancstates: global optim, 3 areas max. d=0.0011; e=1e−04; j=0; LnL=−33.01

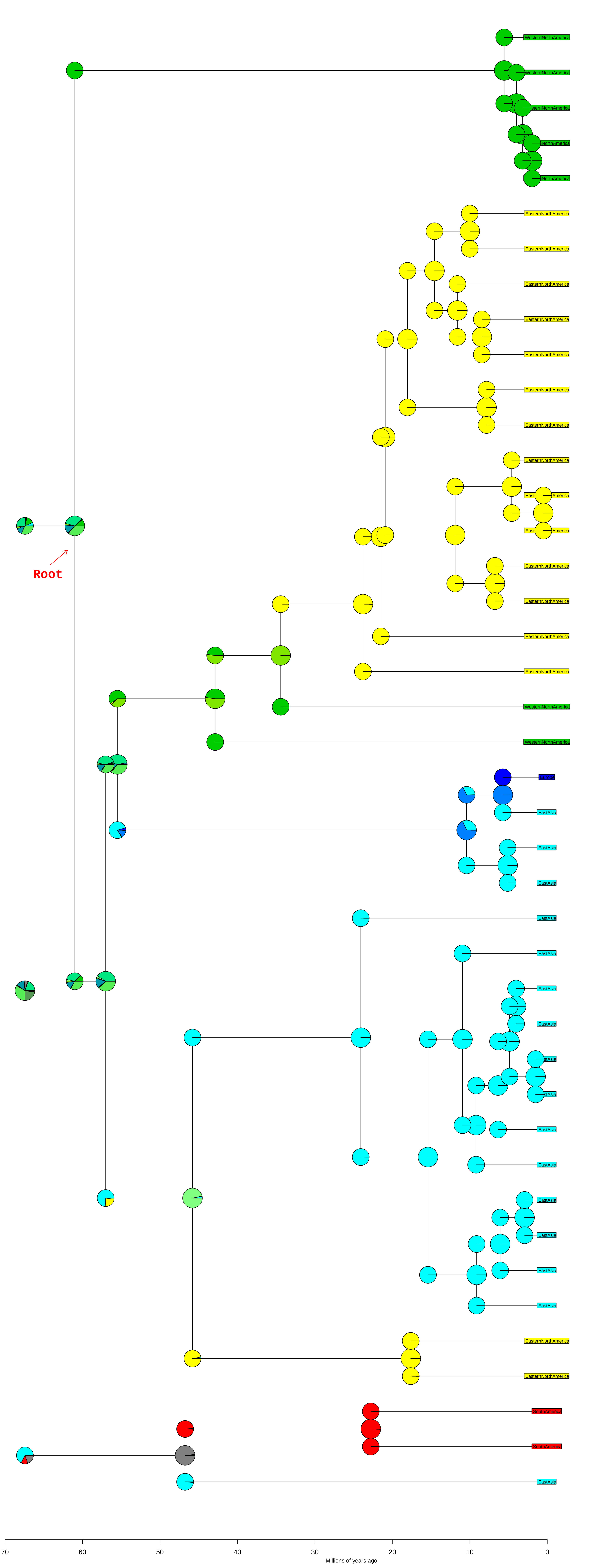

Sect. *Quinquefoliae* BioGeoBEARS DEC

ancstates: global optim, 3 areas max. d=0.0033; e=0; j=0; LnL=−57.50

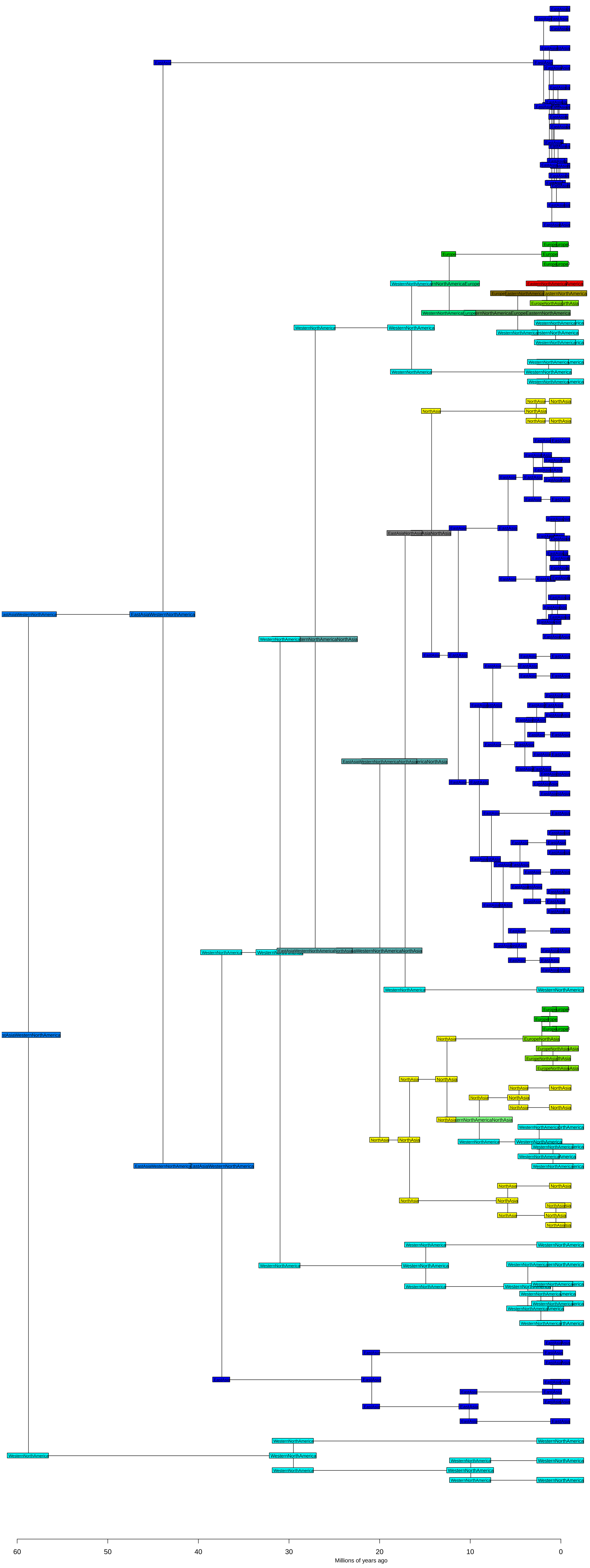

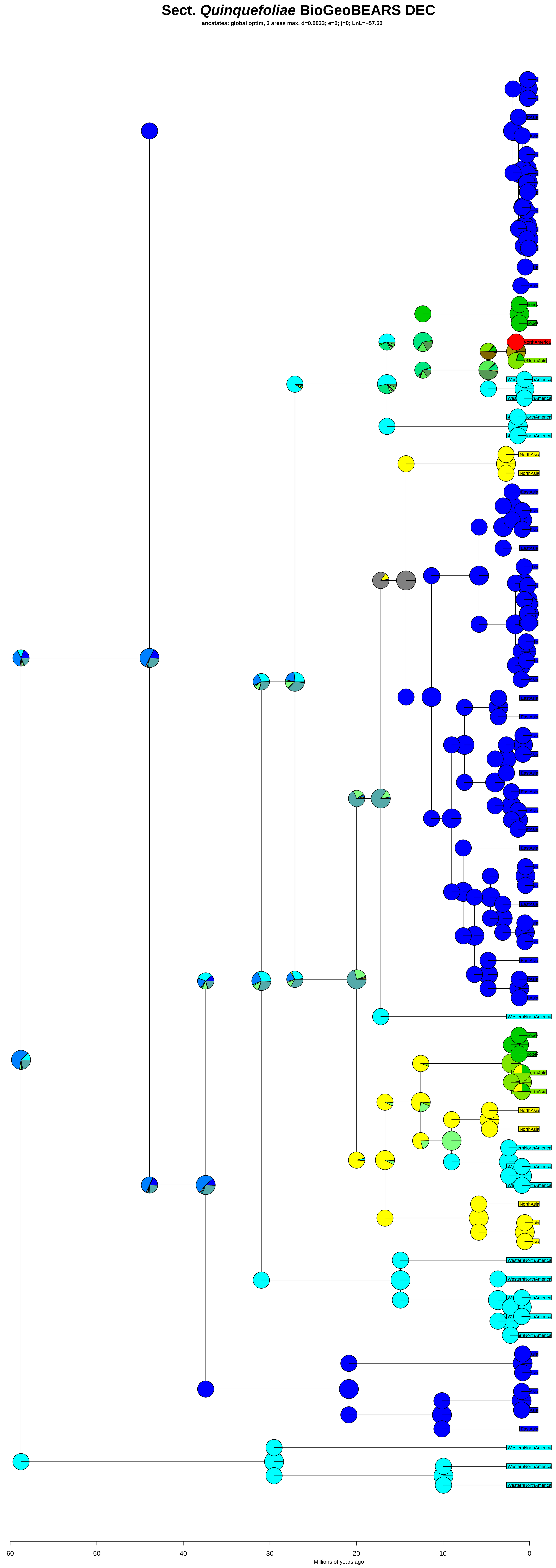
Sect. Quinquefoliae BioGeoBEARS DEC
ancstates: global optim, 3 areas max. d=0.0033; e=0; j=0; LnL=−57.50
WesternNorthAmerica
NorthAsia
60
50
40
30
20
10
0
Millions of years ago

Juniperus BioGeoBEARS DEC

ancstates: global optim, 3 areas max. d=0.003; e=3e−04; j=0; LnL=−133.94

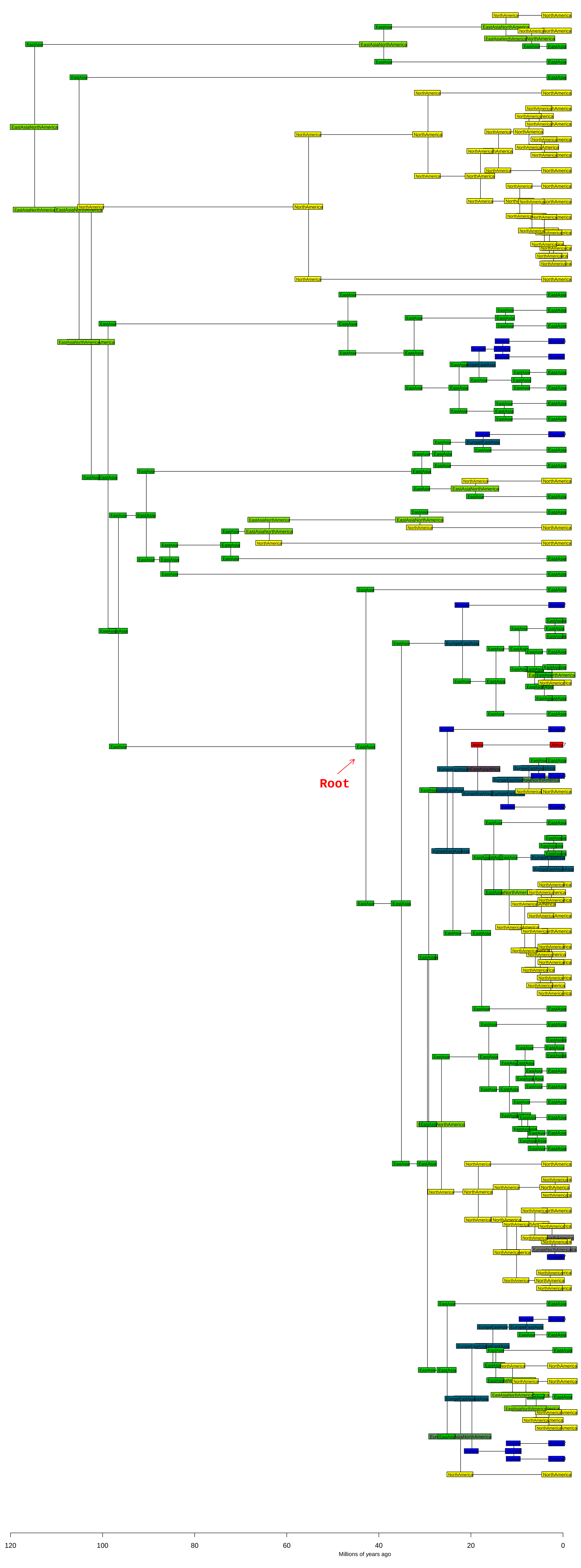

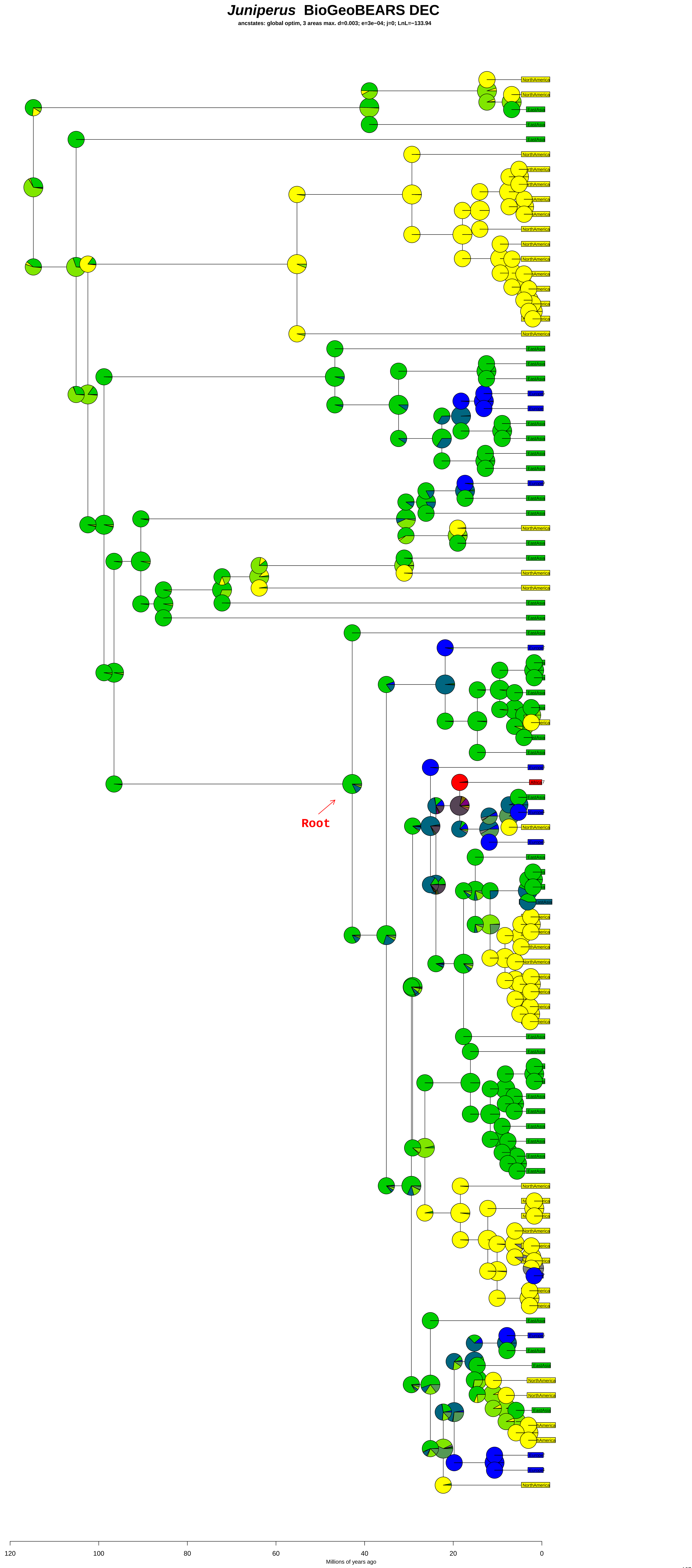
Juniperus BioGeoBEARS DEC
ancstates: global optim, 3 areas max. d=0.003; e=3e−04; j=0; LnL=−133.94
Root
NorthAmerica
EastAsia
120
100
80
60
40
20
0
Millions of years ago

Abies BioGeoBEARS DEC

ancstates: global optim, 3 areas max. d=0.0039; e=0; j=0; LnL=−38.35

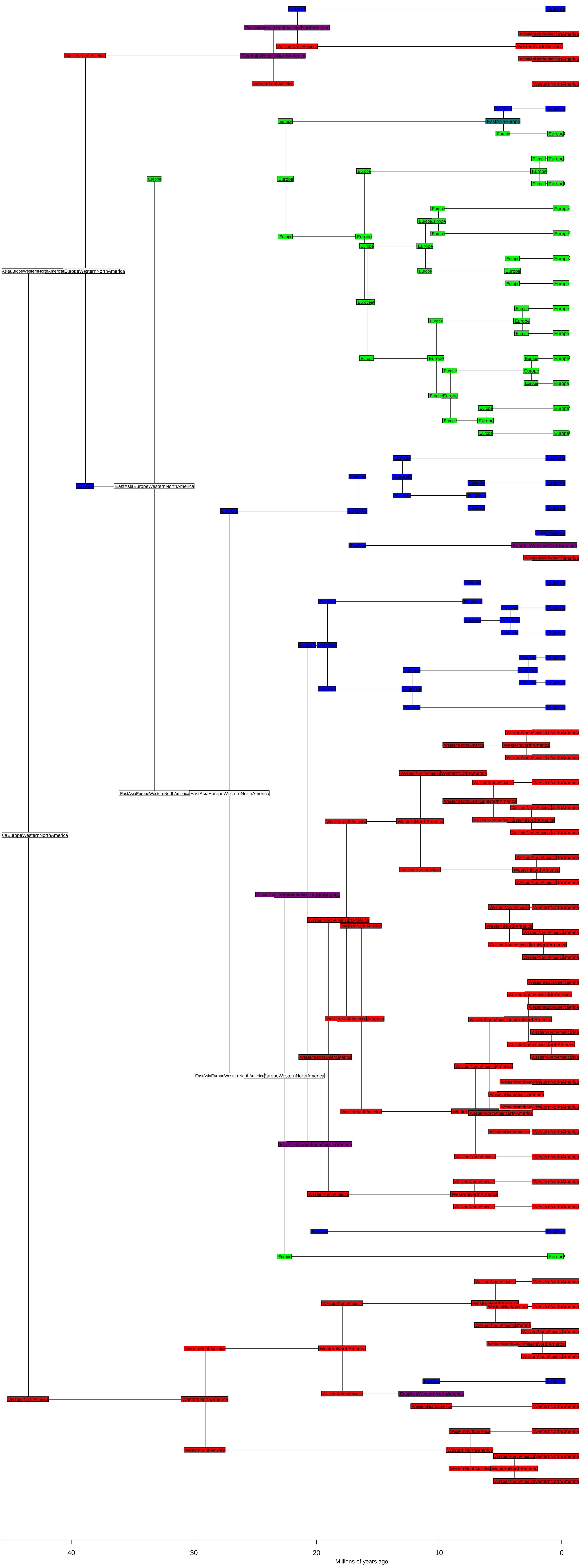

Abies BioGeoBEARS DEC

ancstates: global optim, 3 areas max. d=0.0039; e=0; j=0; LnL=−38.35

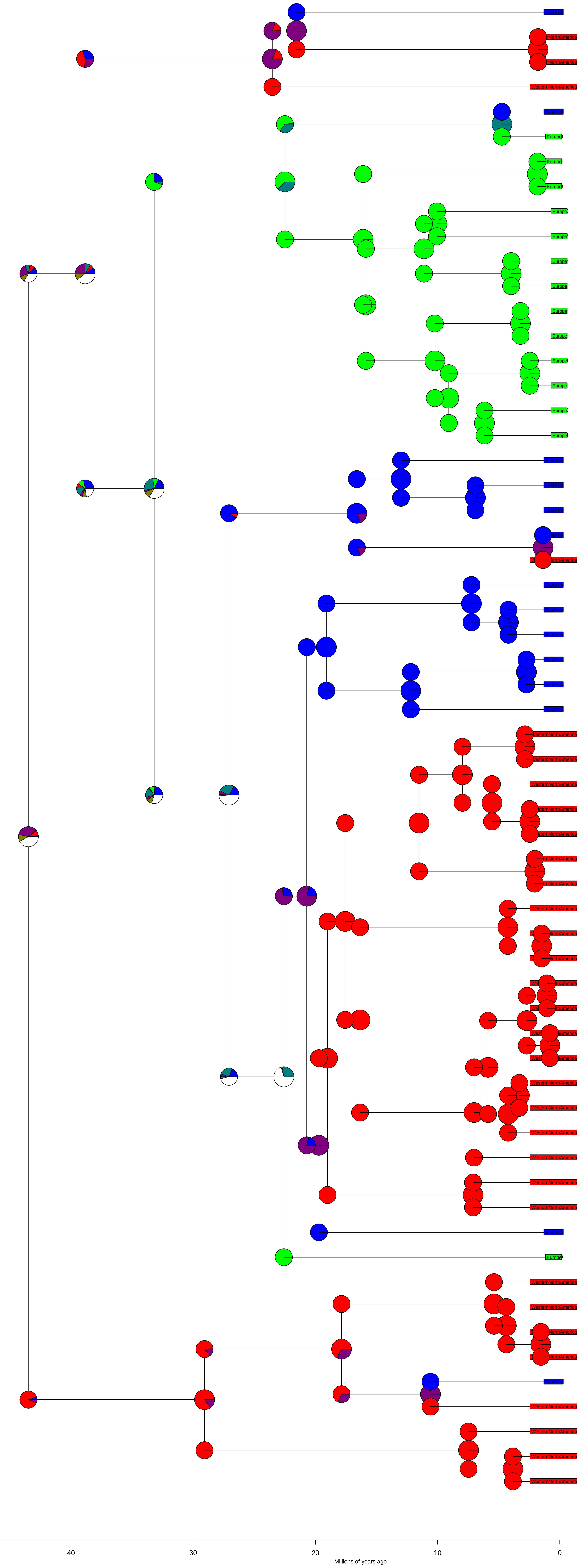

Peracarpeae BioGeoBEARS DEC

ancstates: global optim, 3 areas max. d=0.0054; e=0; j=0; LnL=−37.14

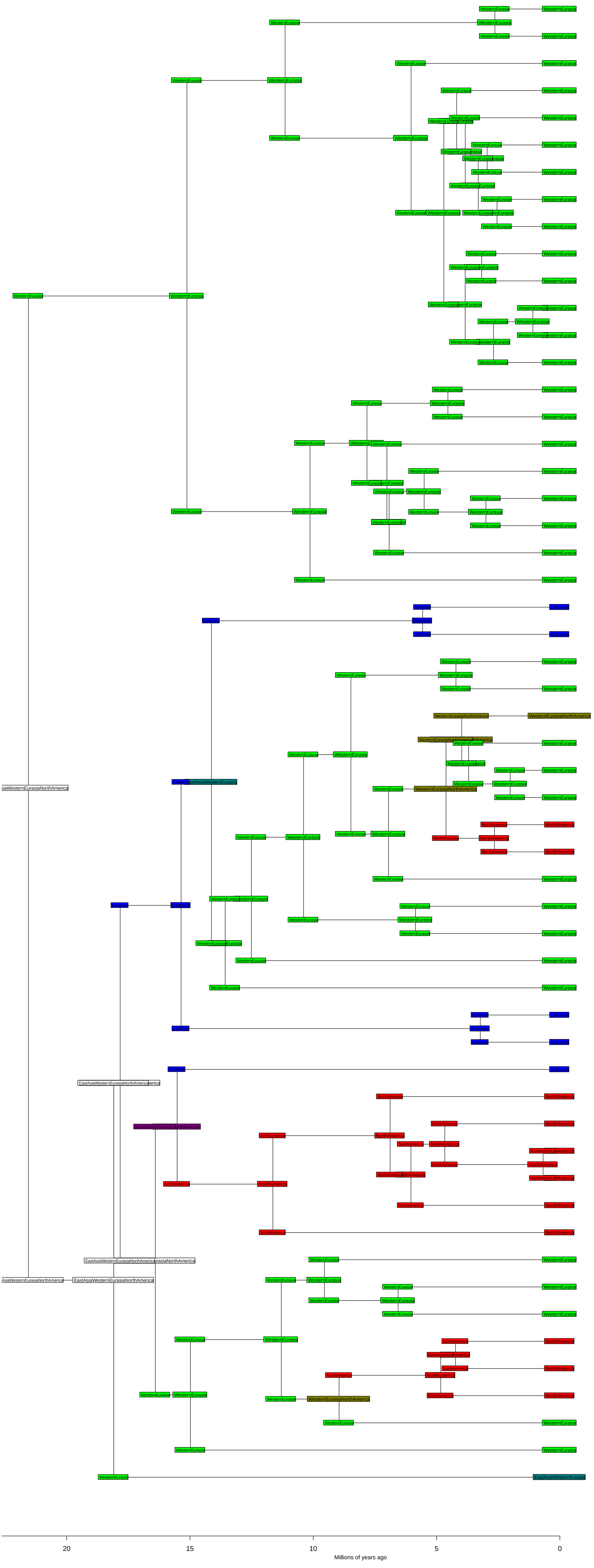

***Peracarpeae* BioGeoBEARS DEC**

ancstates: global optim, 3 areas max. d=0.0054; e=0; j=0; LnL=−37.14

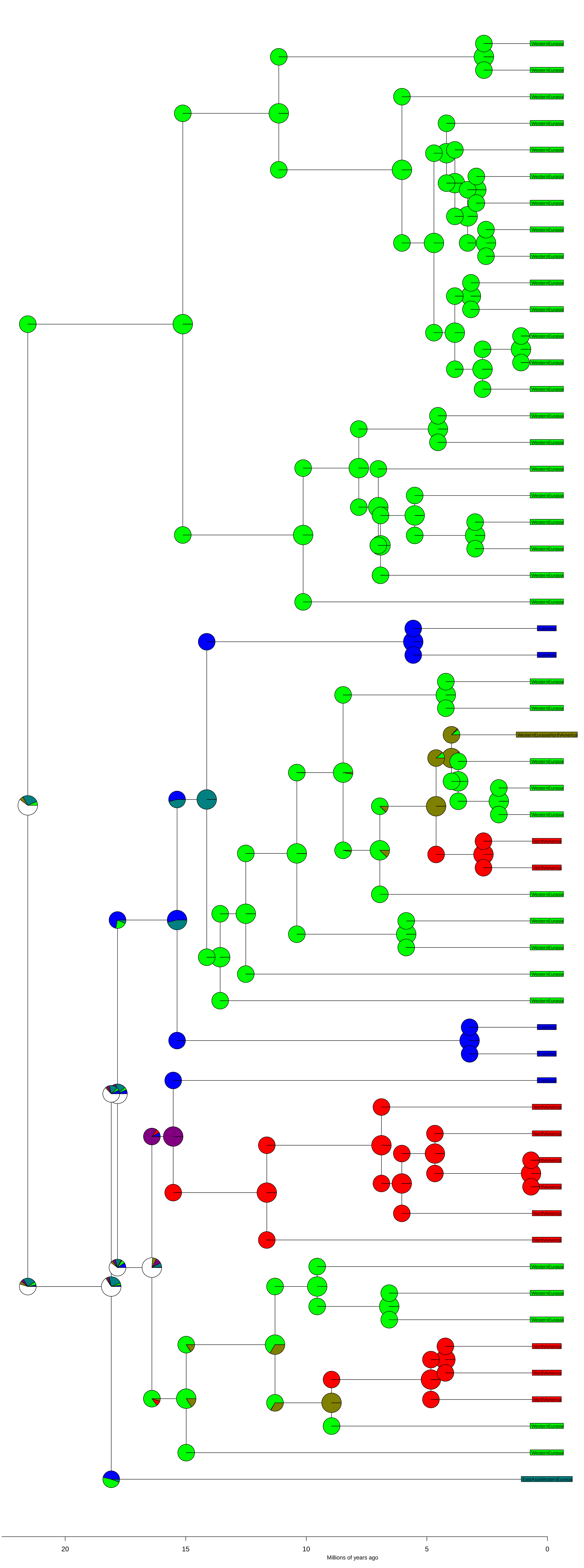

Calocedrus BioGeoBEARS DEC

ancstates: global optim, 3 areas max. d=0; e=0; j=0; LnL=−1.79

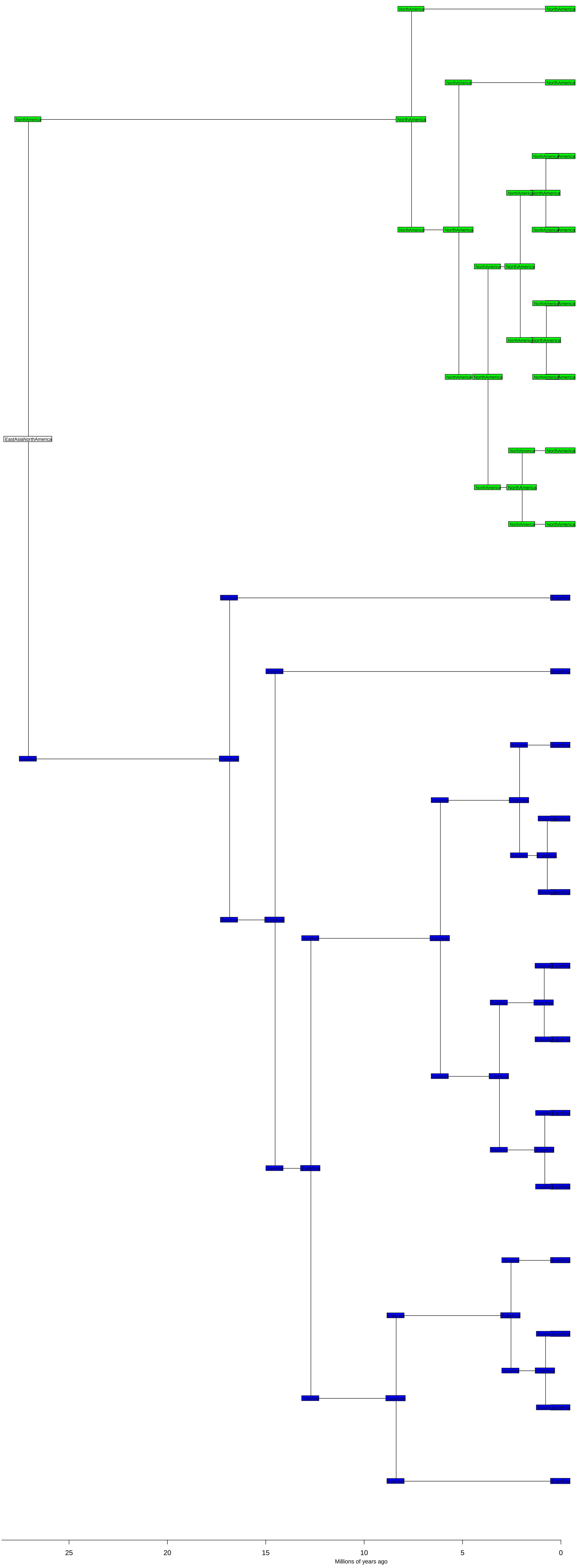

Calocedrus BioGeoBEARS DEC

ancstates: global optim, 3 areas max. d=0; e=0; j=0; LnL=−1.79

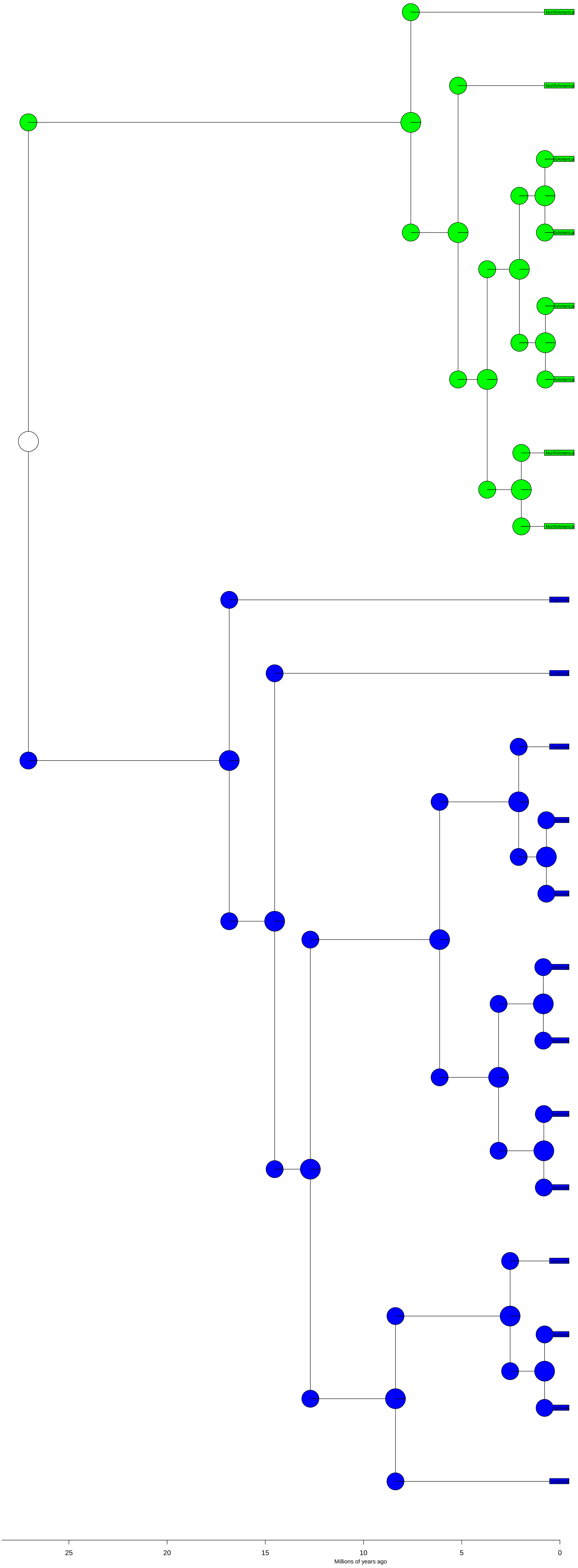

Altingiaceae BioGeoBEARS DEC

ancstates: global optim, 3 areas max. d=0.0018; e=0; j=0; LnL=−24.91

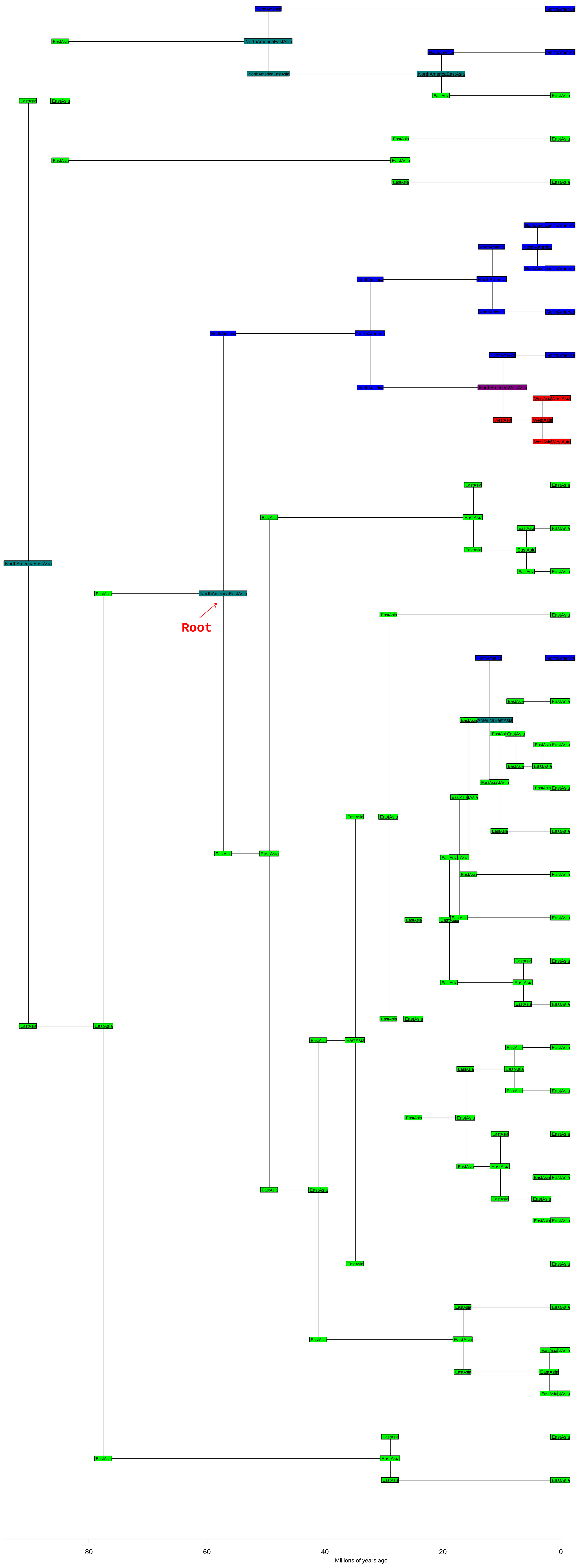

Altingiaceae BioGeoBEARS DEC

ancstates: global optim, 3 areas max. d=0.0018; e=0; j=0; LnL=-24.91

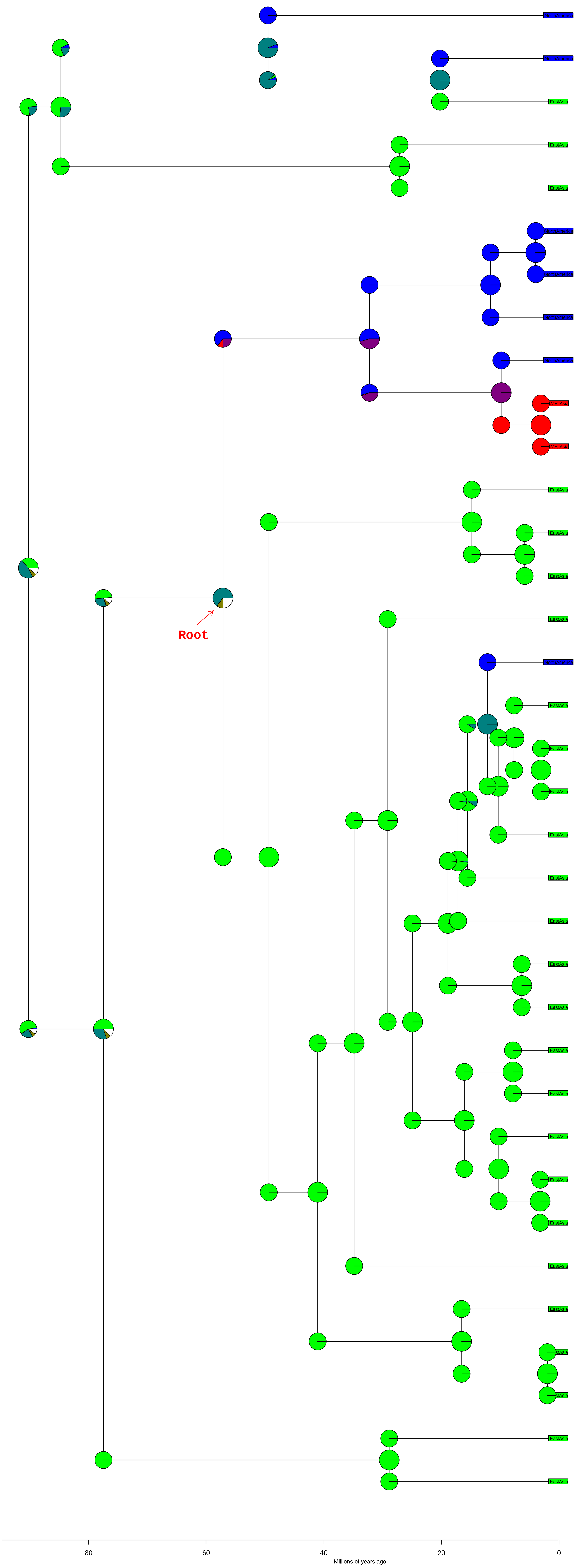

Rhodiola BioGeoBEARS DEC

ancstates: global optim, 3 areas max. d=0.0057; e=0; j=0; LnL=−30.78

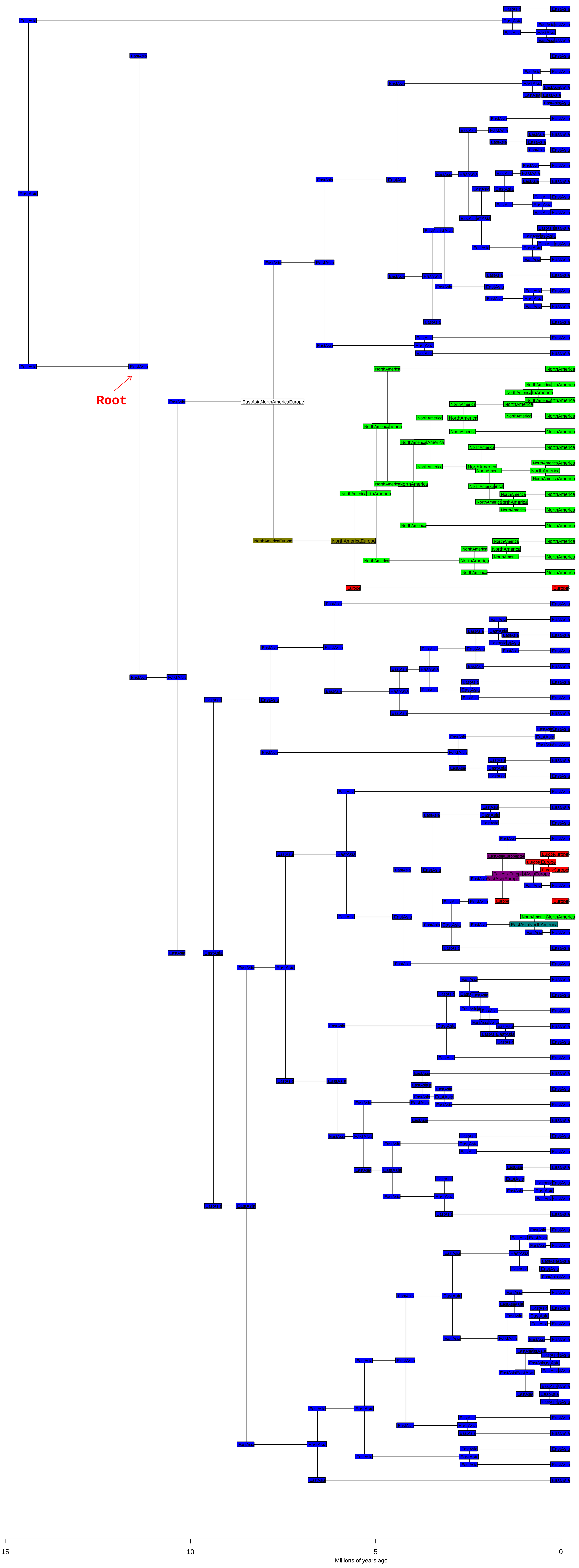

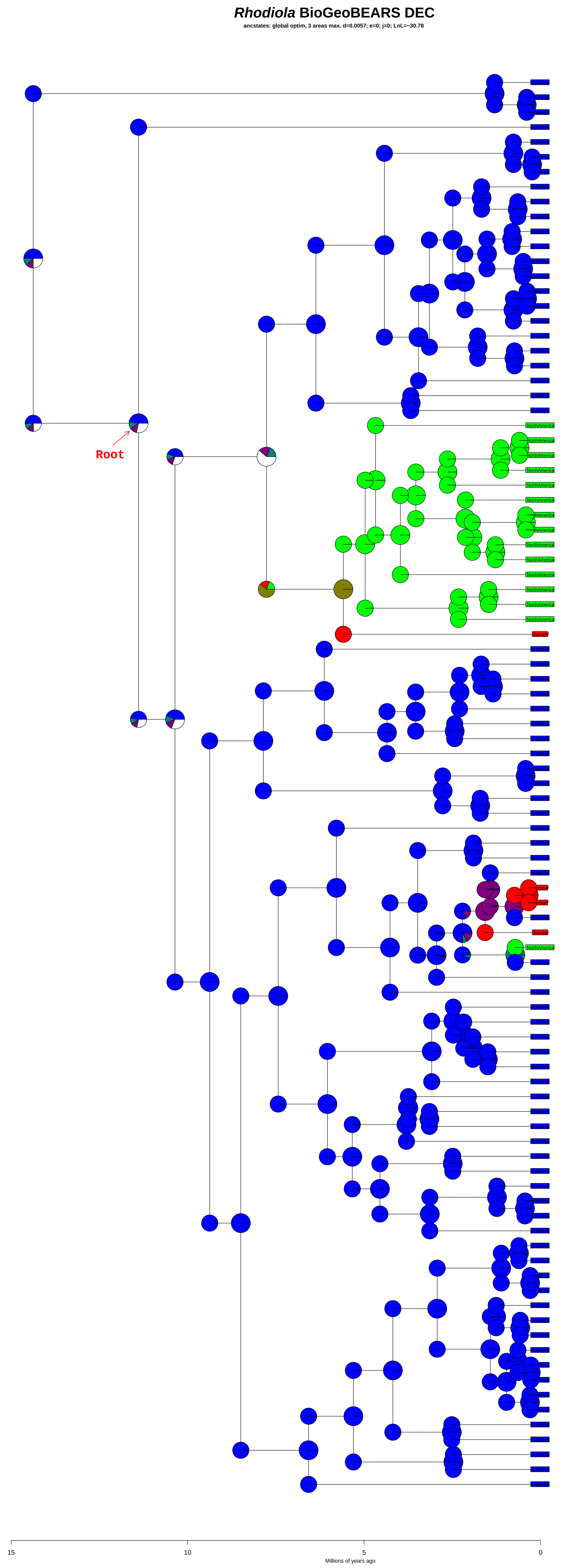
Rhodiola BioGeoBEARS DEC
ancstates: global optim, 3 areas max. d=0.0057; e=0; j=0; LnL=−30.78
Root
NorthAmerica
Europe
15
10
5
0
Millions of years ago

Dryopteridaceae BioGeoBEARS DEC

ancstates: global optim, 3 areas max. d=0.0014; e=0; j=0; LnL=-210.98

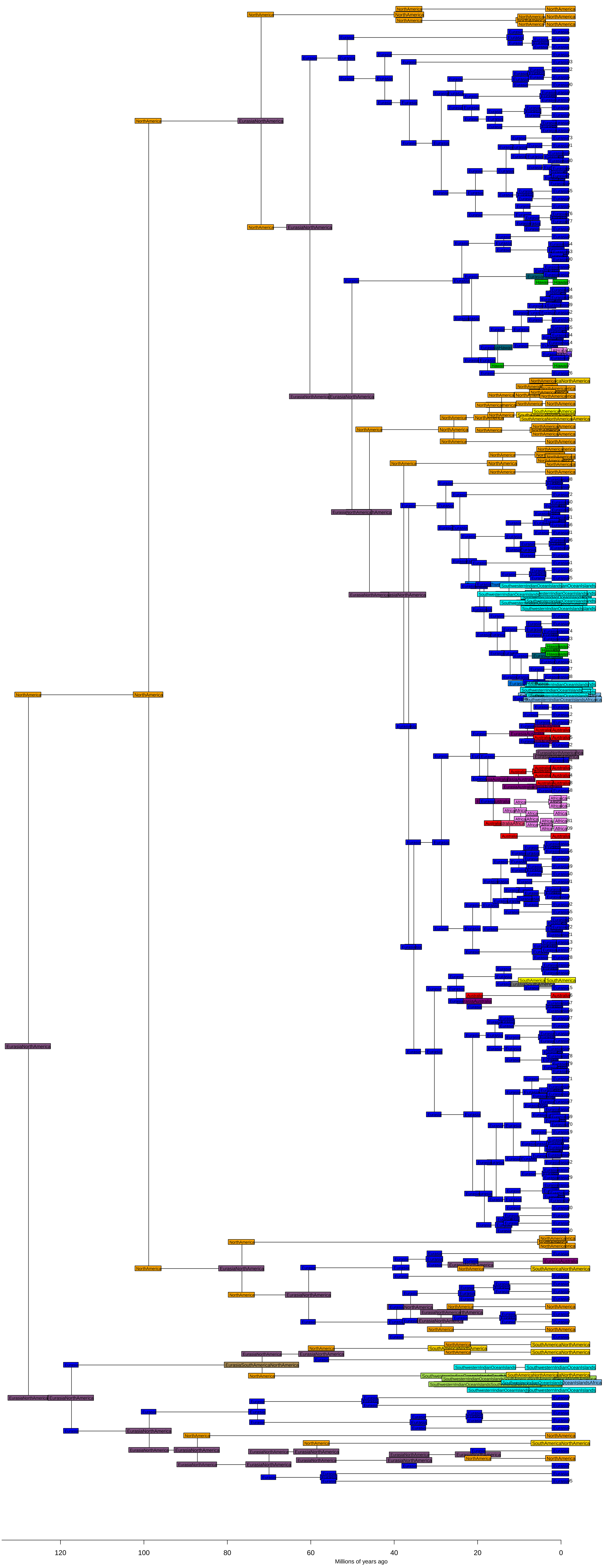

Dryopteridaceae BioGeoBEARS DEC

ancstates: global optim, 3 areas max. d=0.0014; e=0; j=0; LnL=−210.98

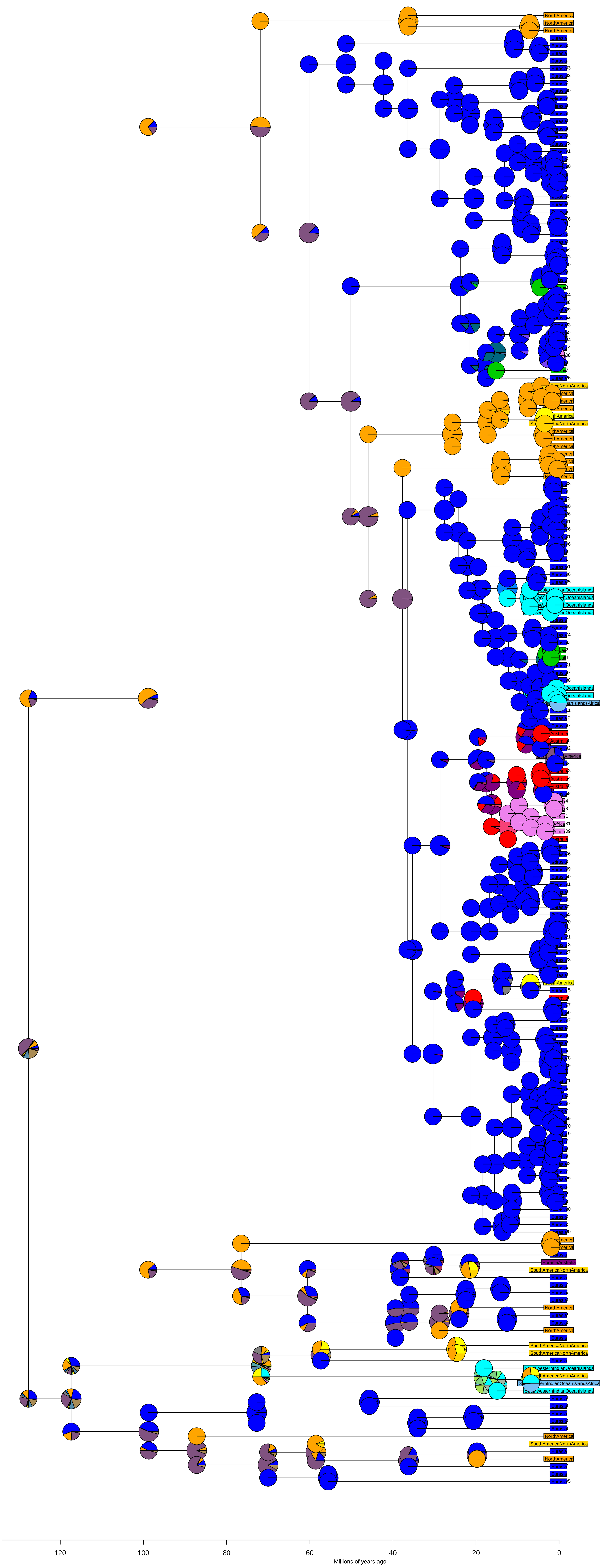

Rana BioGeoBEARS DEC

ancstates: global optim, 3 areas max. d=9e−04; e=0; j=0; LnL=−75.36

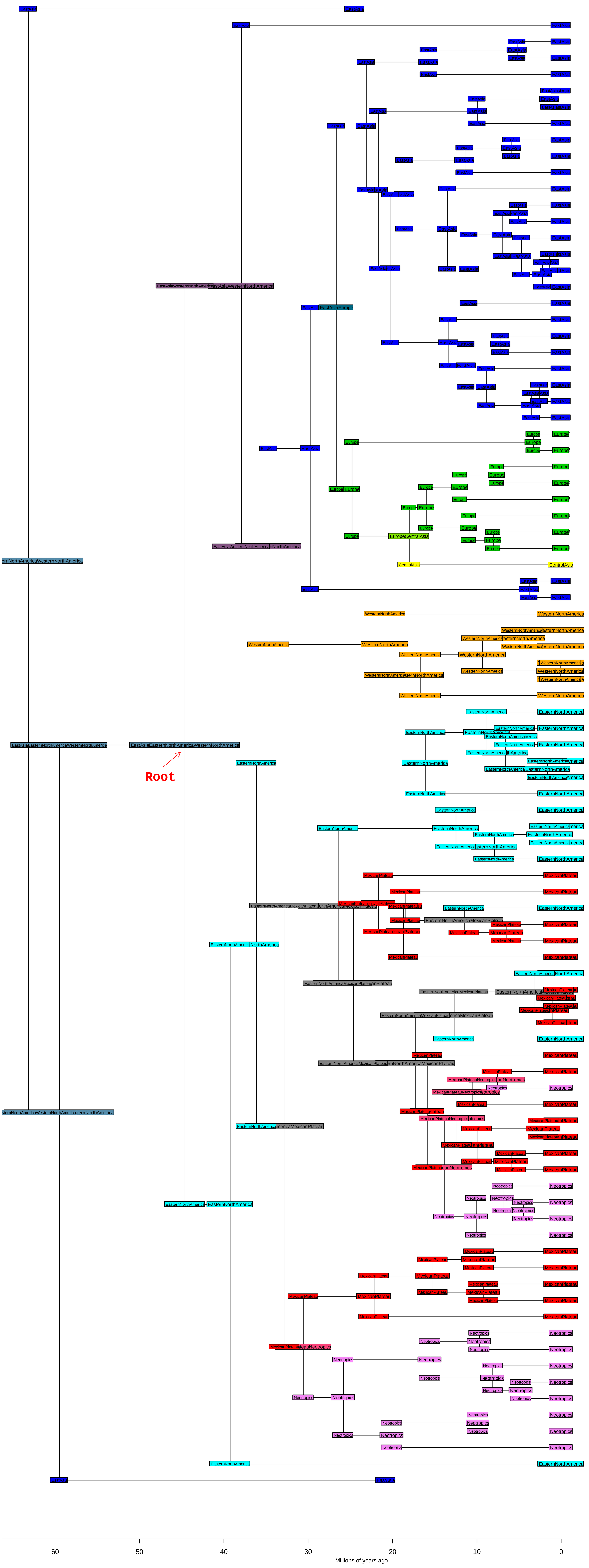

Rana BioGeoBEARS DEC

ancstates: global optim, 3 areas max. d=9e−04; e=0; j=0; LnL=−75.36

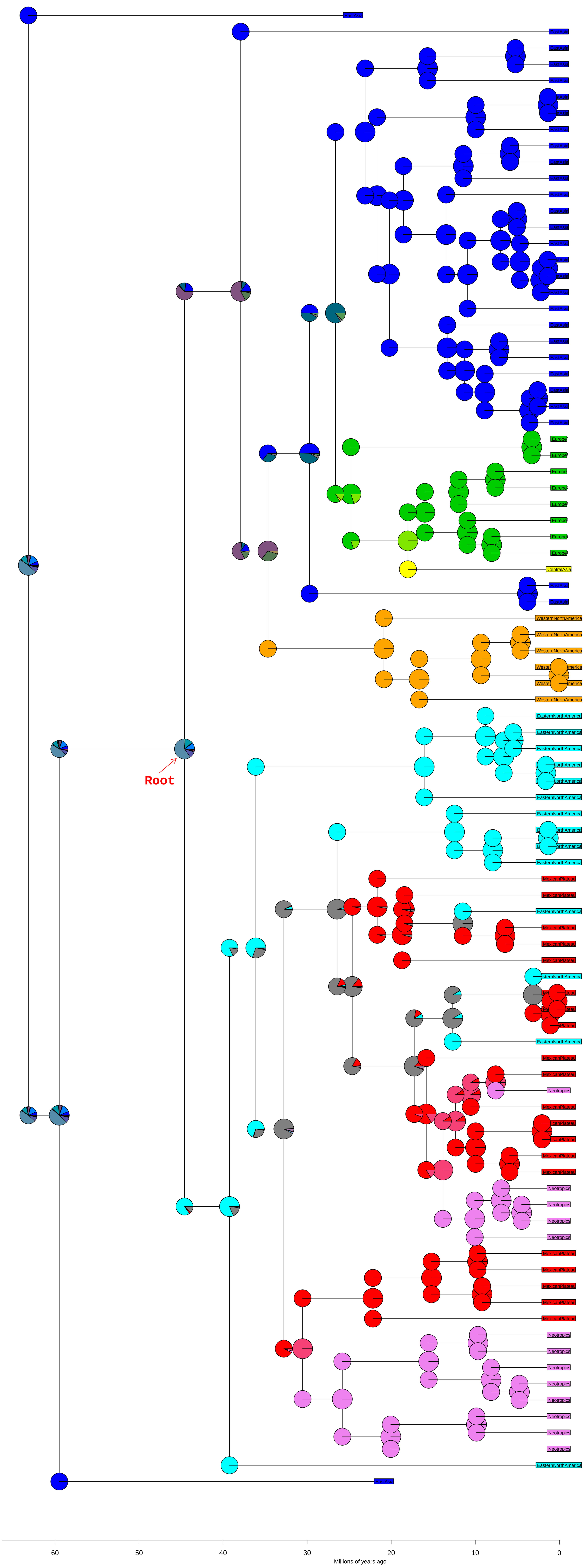

***Hyla* BioGeoBEARS DEC**

ancstates: global optim, 3 areas max. d=6e−04; e=0; j=0; LnL=−25.57

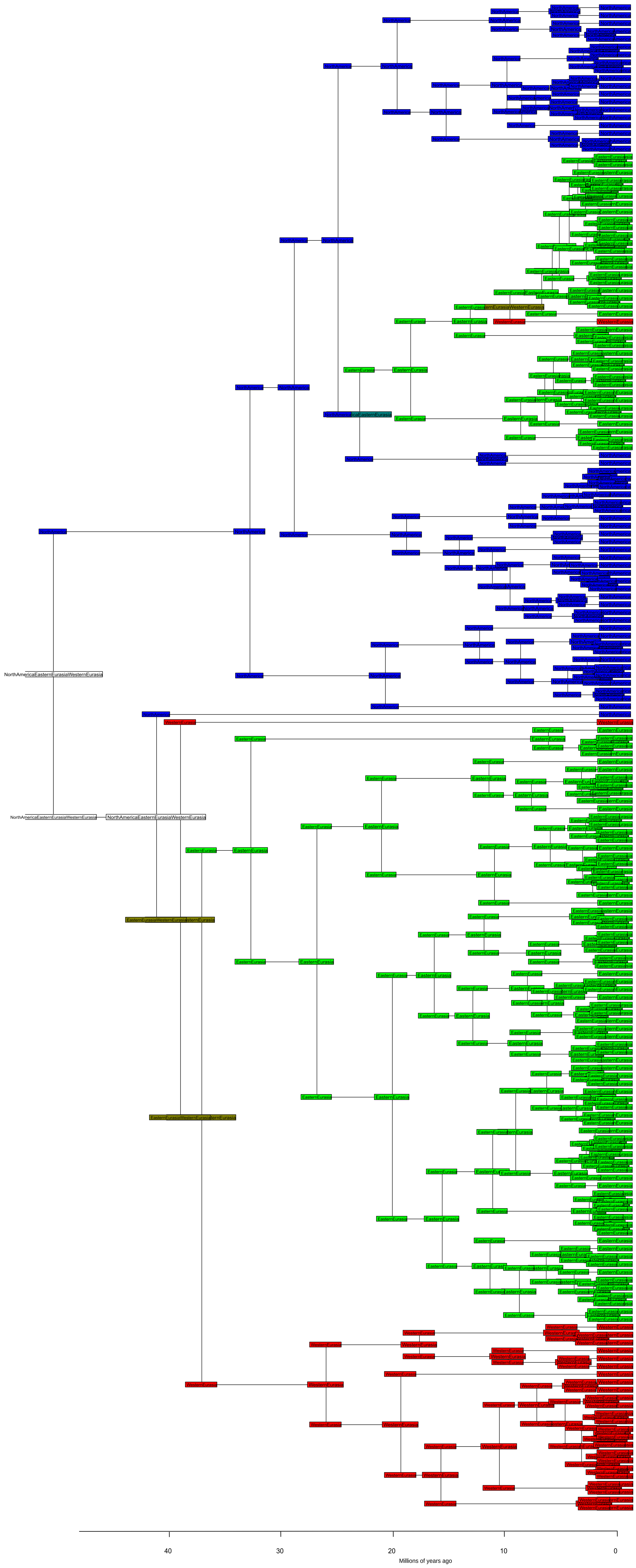

Hyla BioGeoBEARS DEC

ancstates: global optim, 3 areas max. d=6e−04; e=0; j=0; LnL=−25.57

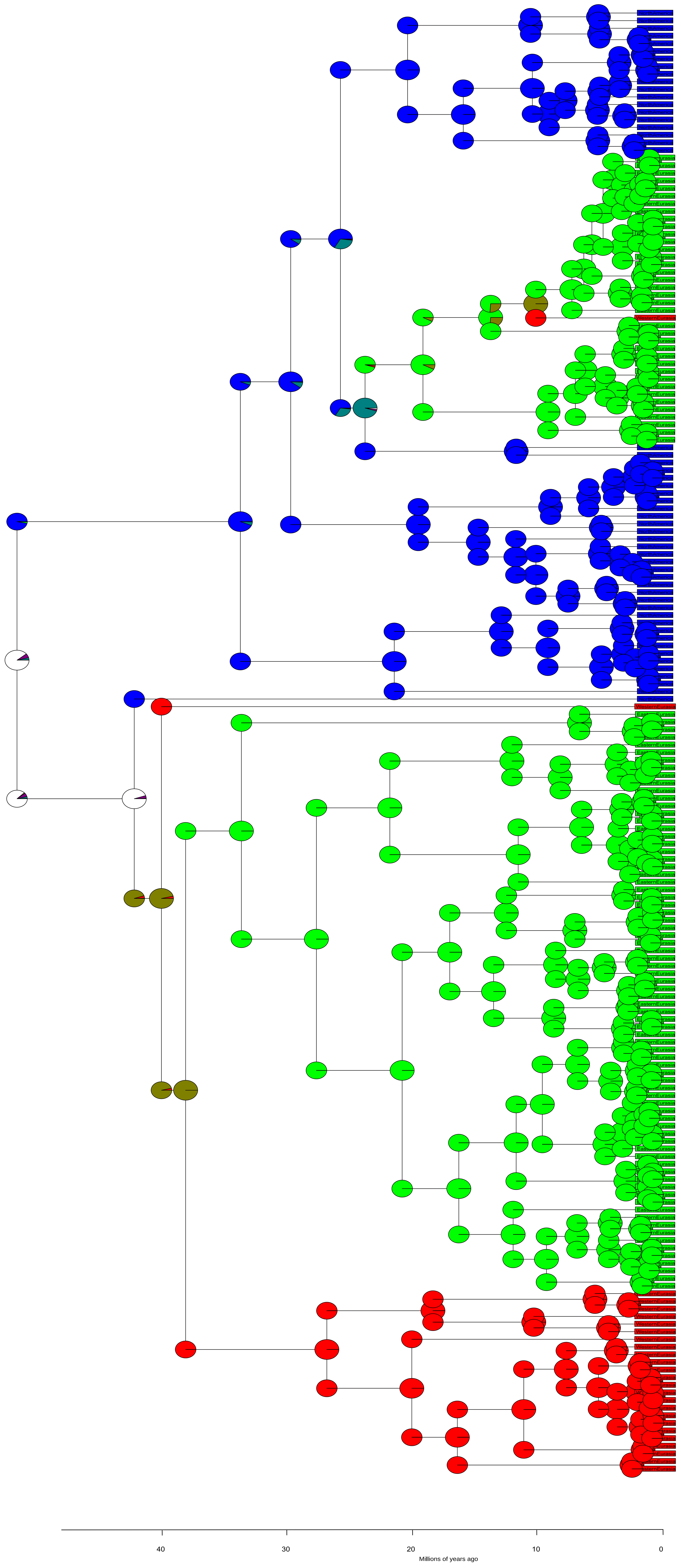

Bufonidae BioGeoBEARS DEC

ancstates: global optim, 2 areas max. d=0.0011; e=4e−04; j=0; LnL=−32.04

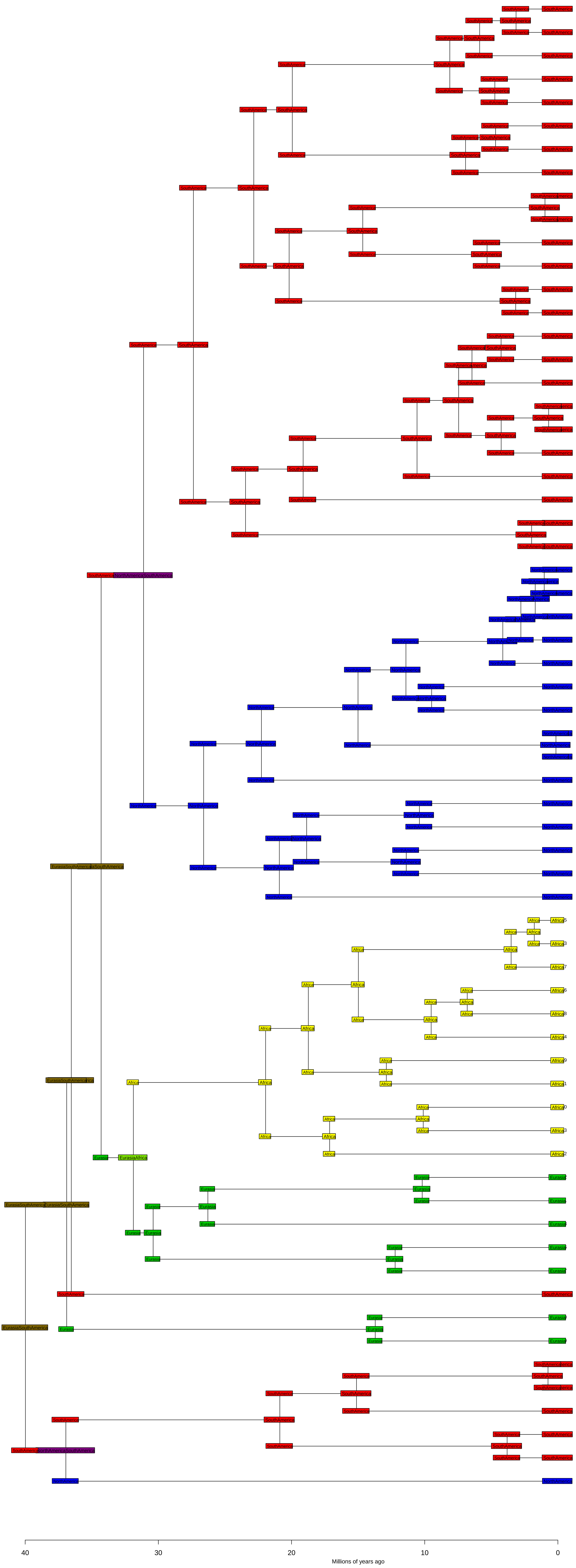

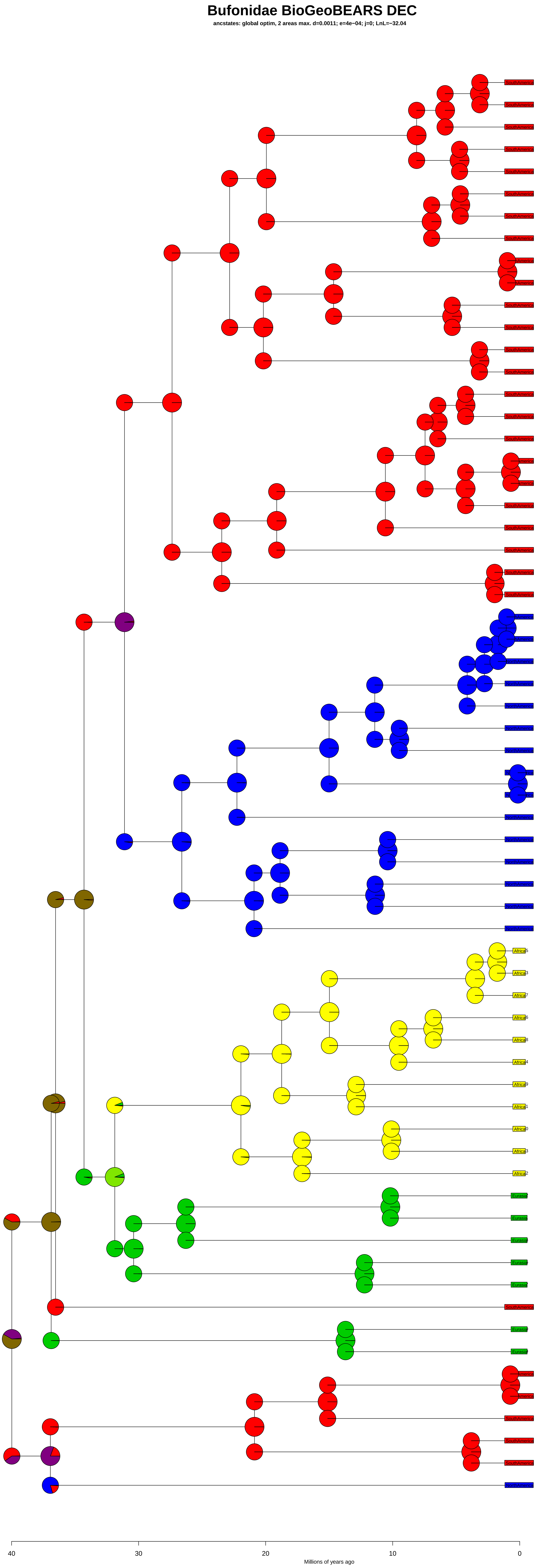
Bufonidae BioGeoBEARS DEC
ancstates: global optim, 2 areas max. d=0.0011; e=4e−04; j=0; LnL=−32.04
40
30
20
10
0
Millions of years ago

Natricine BioGeoBEARS DEC

ancstates: global optim, 3 areas max. d=4e−04; e=0; j=0; LnL=−15.78

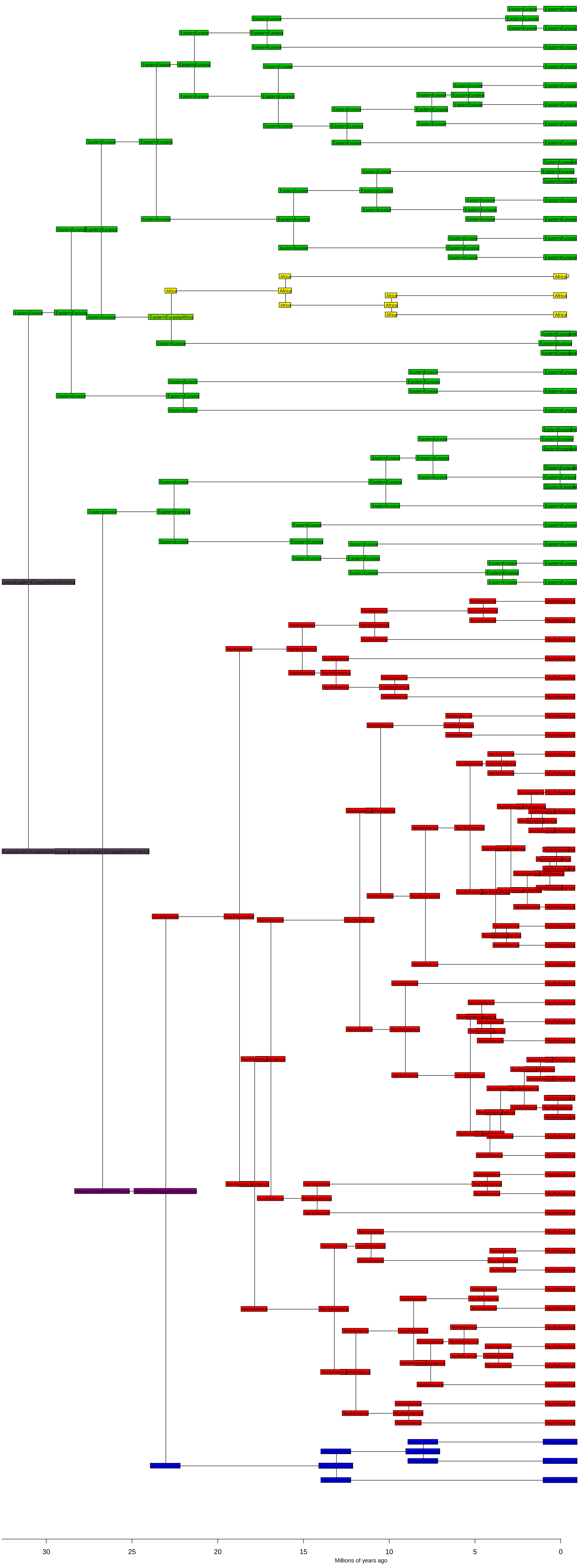

Natricine BioGeoBEARS DEC

ancstates: global optim, 3 areas max. d=4e−04; e=0; j=0; LnL=−15.78

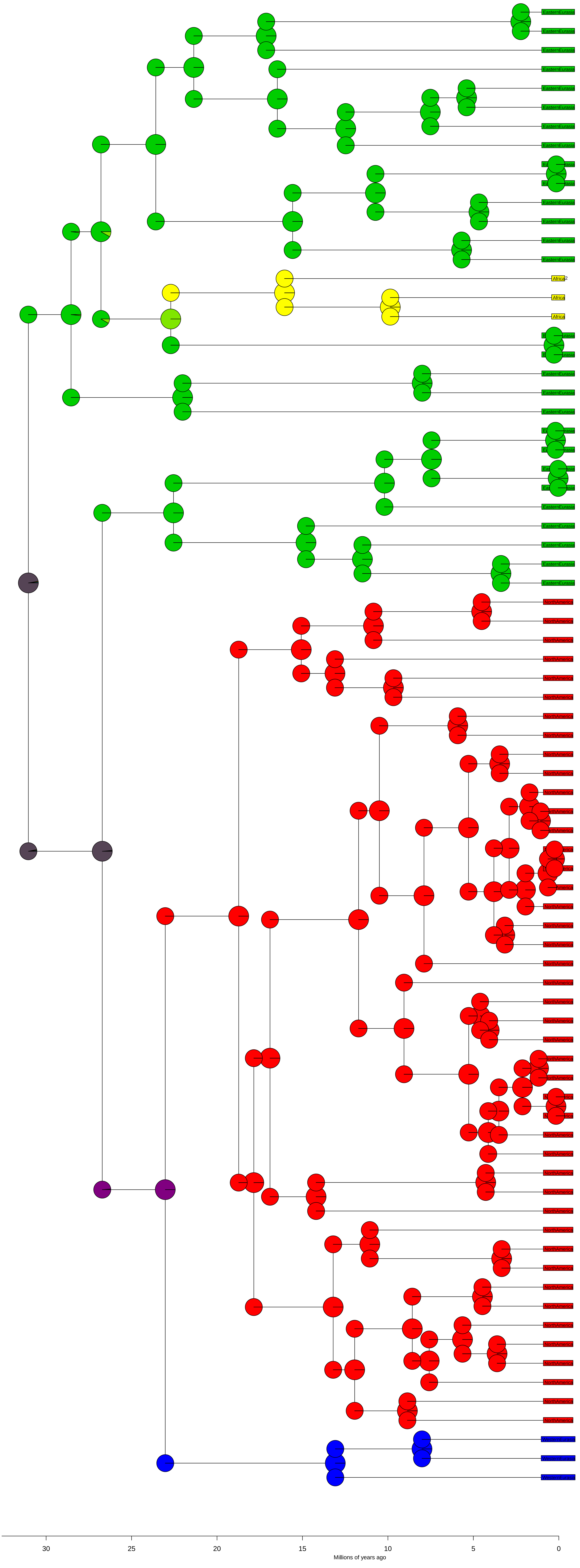

Rat snakes BioGeoBEARS DEC

ancstates: global optim, 3 areas max. d=0.0013; e=0.0016; j=0; LnL=-60.54

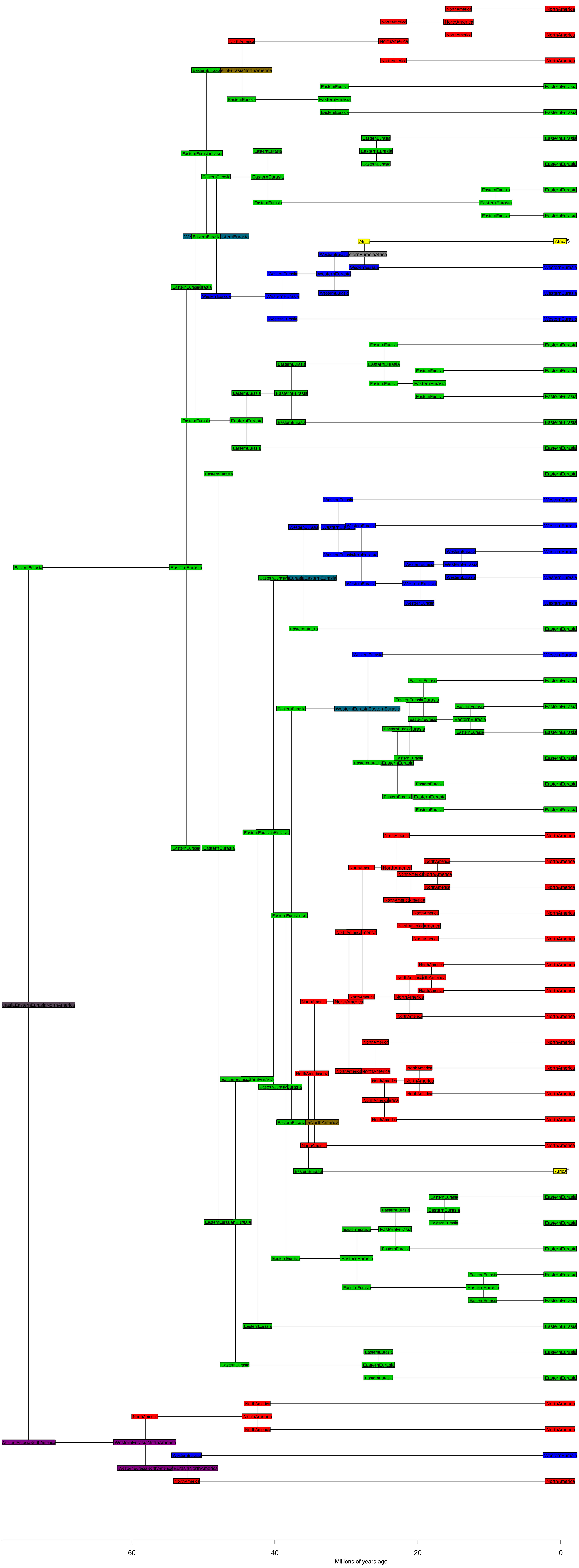

Rat snakes BioGeoBEARS DEC

ancstates: global optim, 3 areas max. d=0.0013; e=0.0016; j=0; LnL=−60.54

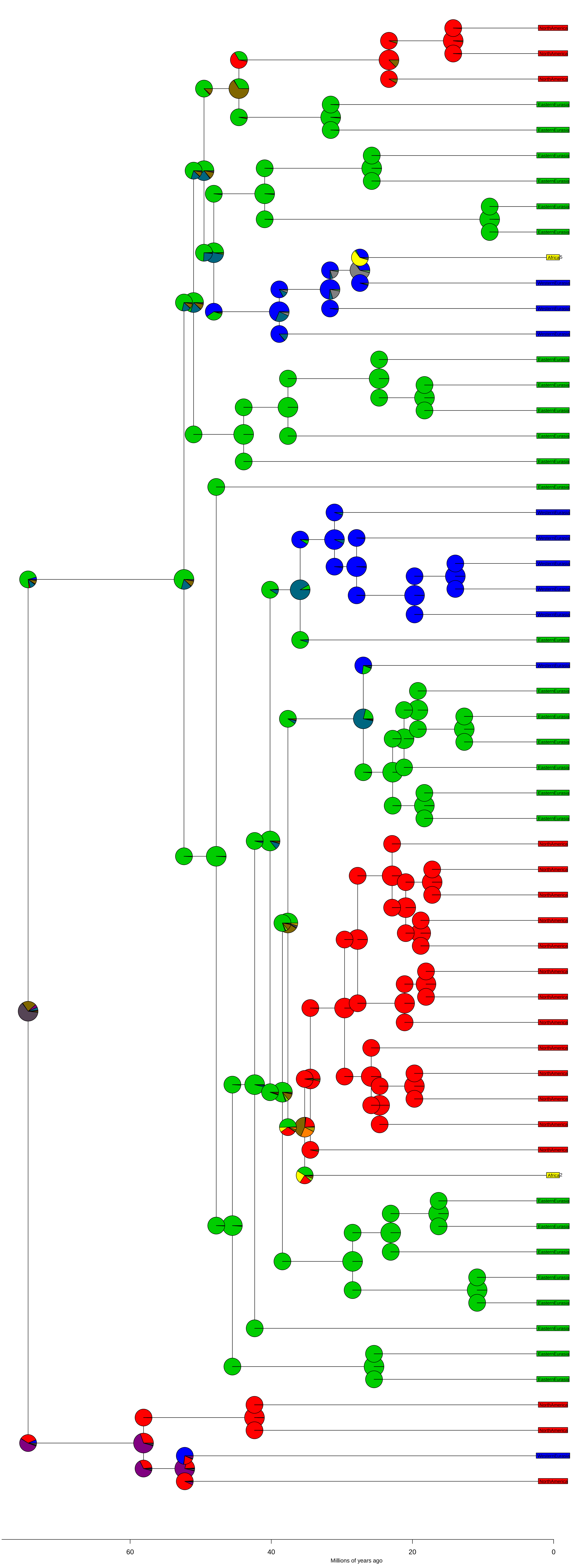

Geoemydidae BioGeoBEARS DEC

ancstates: global optim, 3 areas max. d=0.2837; e=0.3977; j=0; LnL=−42.31

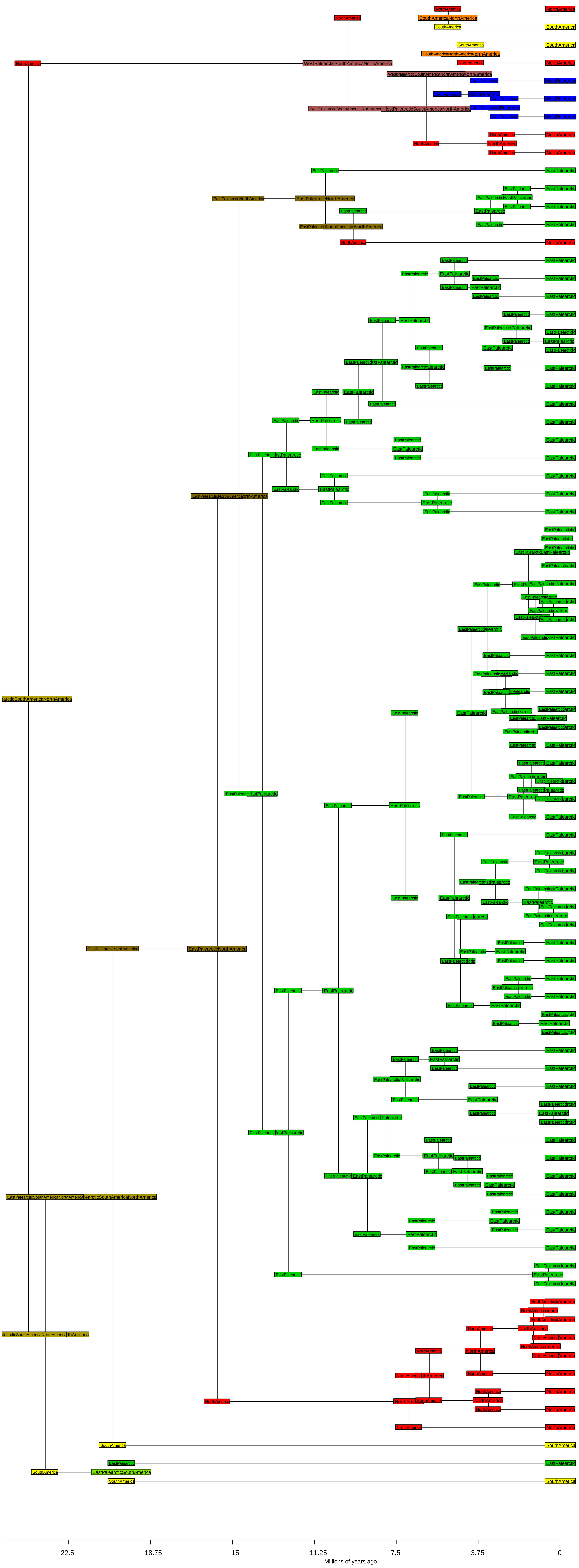

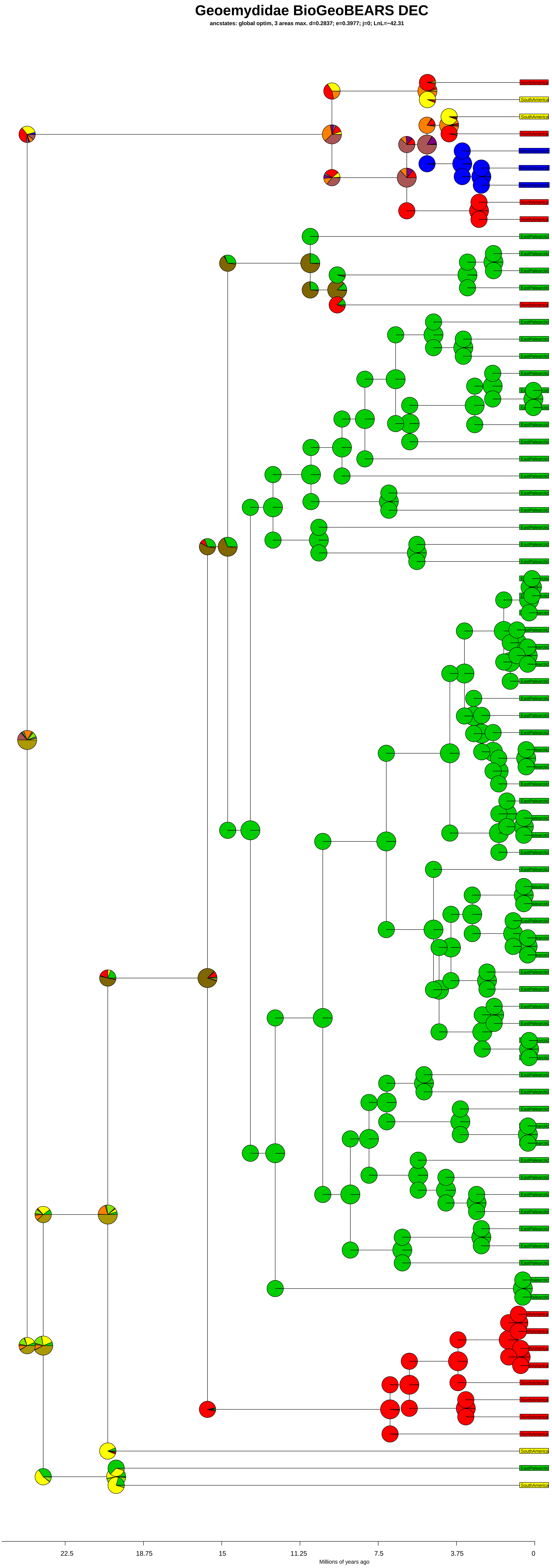
Geoemydidae BioGeoBEARS DEC
ancstates: global optim, 3 areas max. d=0.2837; e=0.3977; j=0; LnL=-42.31
Millions of years ago
22.5
18.75
15
11.25
7.5
3.75
0

Emys BioGeoBEARS DEC

ancstates: global optim, 3 areas max. d=0; e=0; j=0; LnL=−4.28

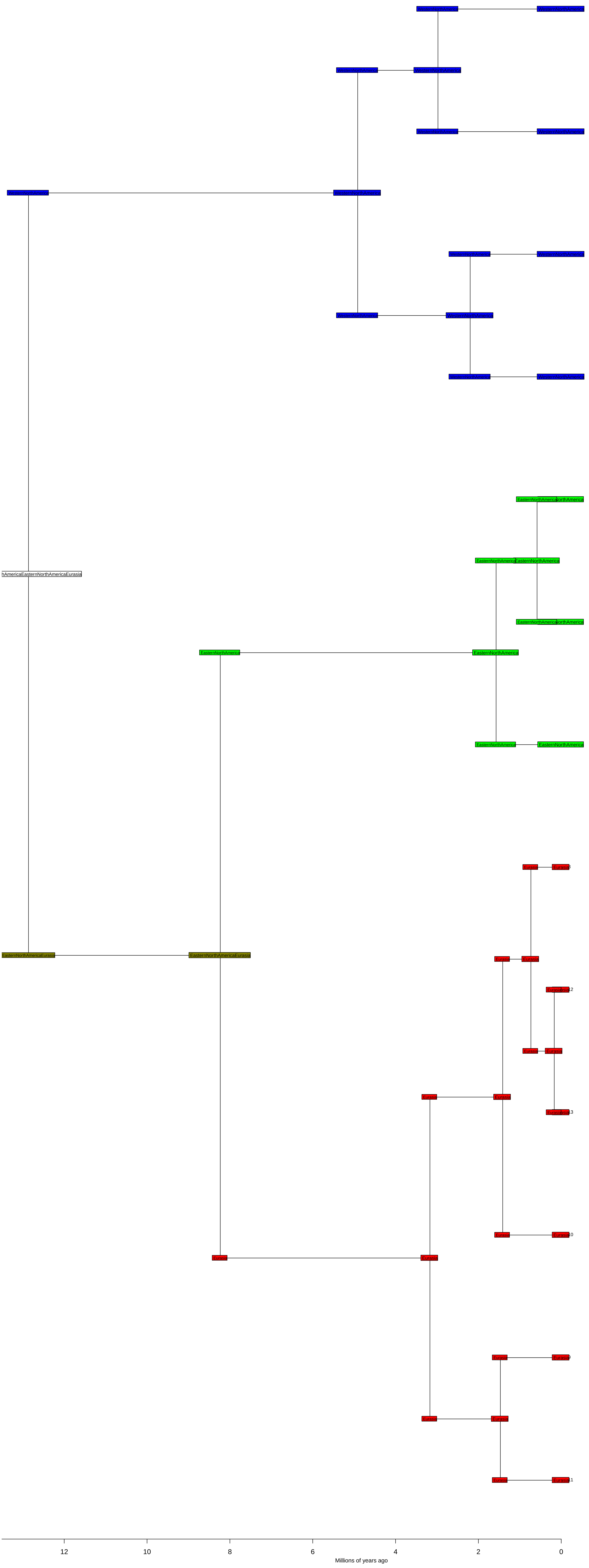

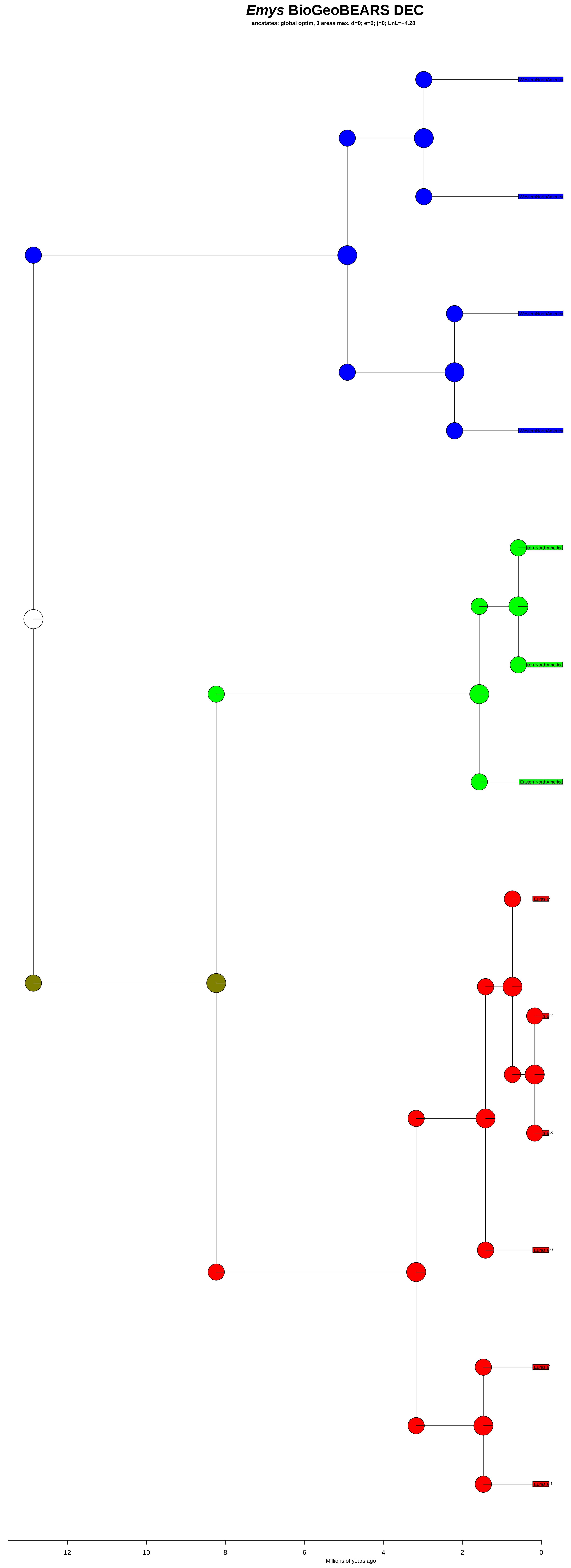

Emys BioGeoBEARS DEC
ancstates: global optim, 3 areas max. d=0; e=0; j=0; LnL=−4.28
EasternNorthAmerica
12
10
8
6
4
2
0
Millions of years ago

Elapoidea BioGeoBEARS DEC

ancstates: global optim, 2 areas max. d=0.0034; e=0.0012; j=0; LnL=−64.15

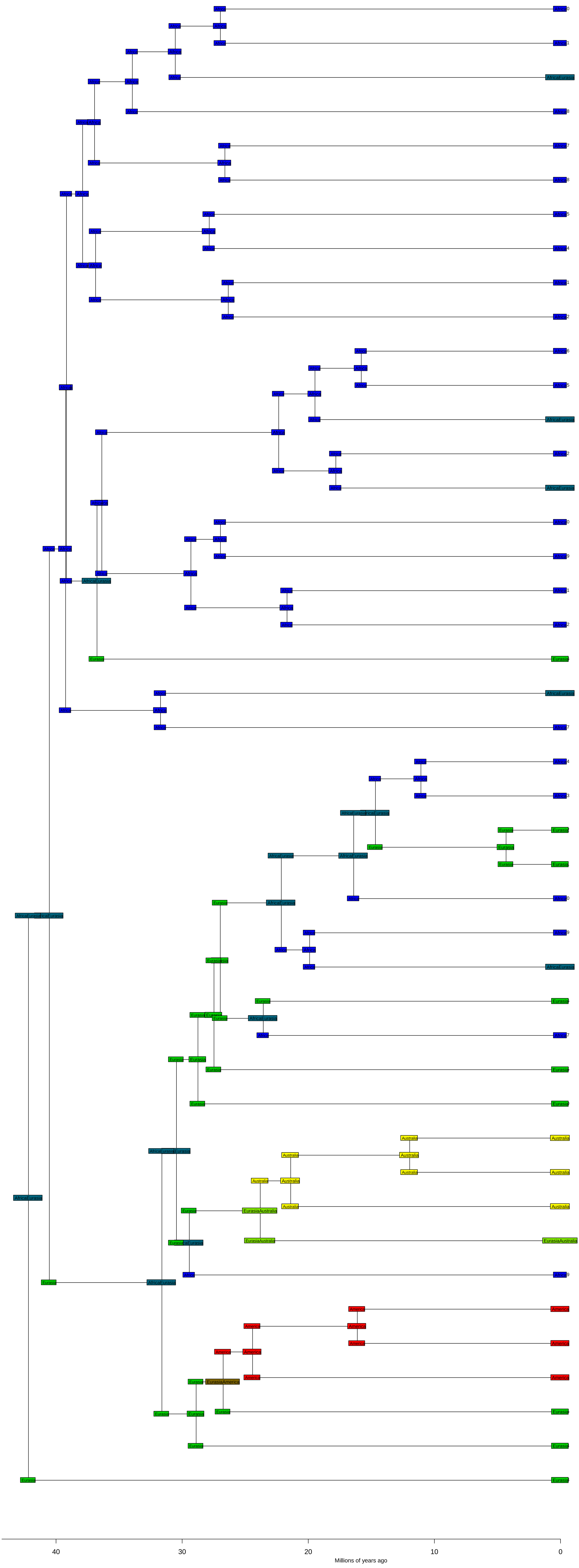

Elapoidea BioGeoBEARS DEC

ancstates: global optim, 2 areas max. d=0.0034; e=0.0012; j=0; LnL=-64.15

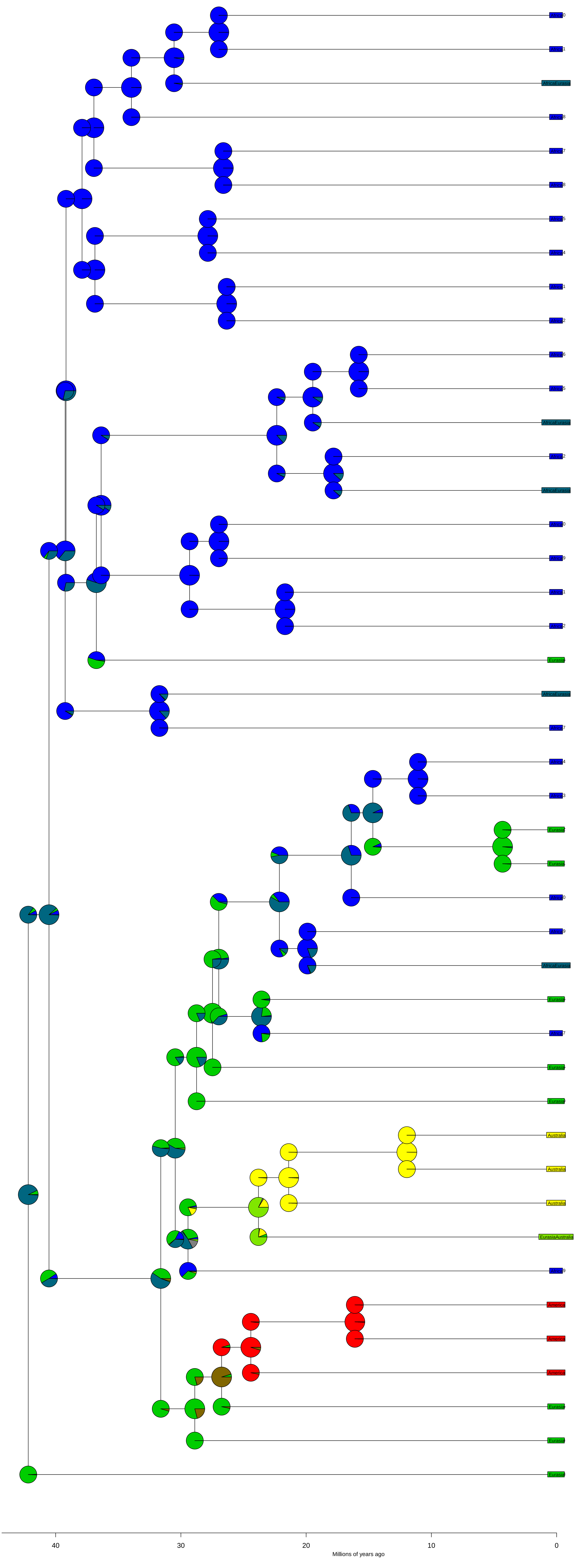

Plestiodon BioGeoBEARS DEC

ancstates: global optim, 3 areas max. d=0; e=0; j=0; LnL=−3.58

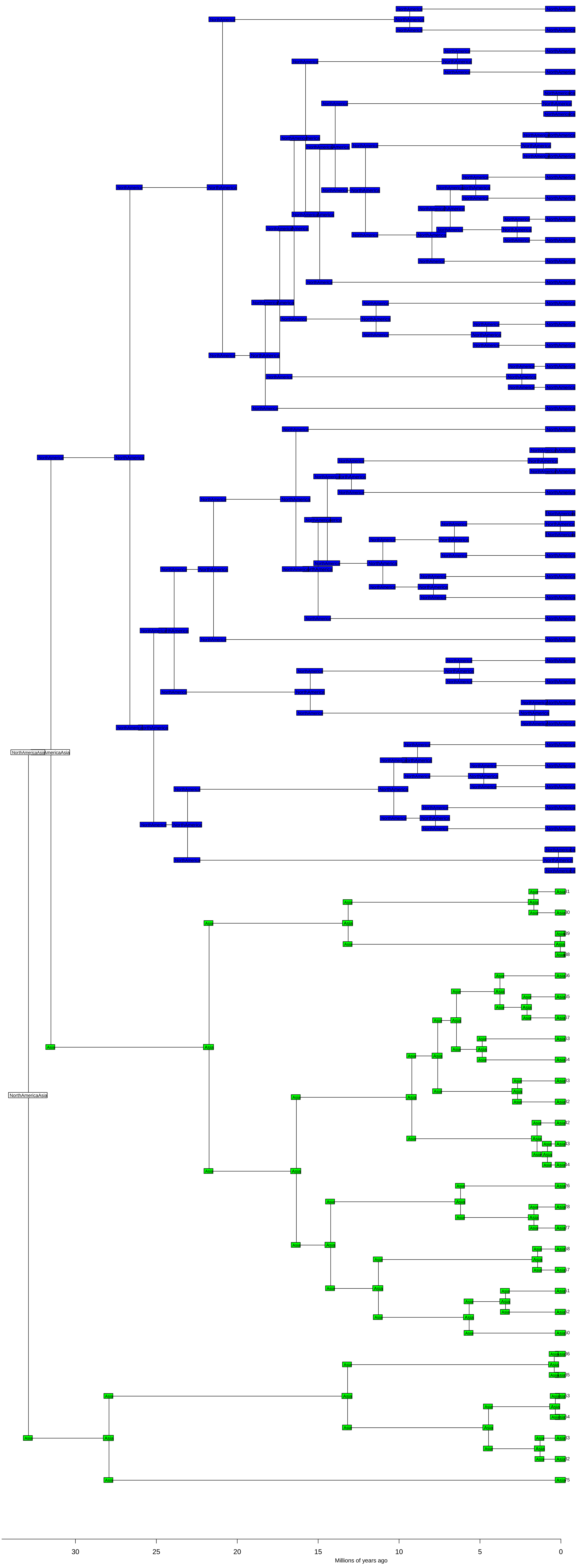

Plestiodon BioGeoBEARS DEC

ancstates: global optim, 3 areas max. d=0; e=0; j=0; LnL=−3.58

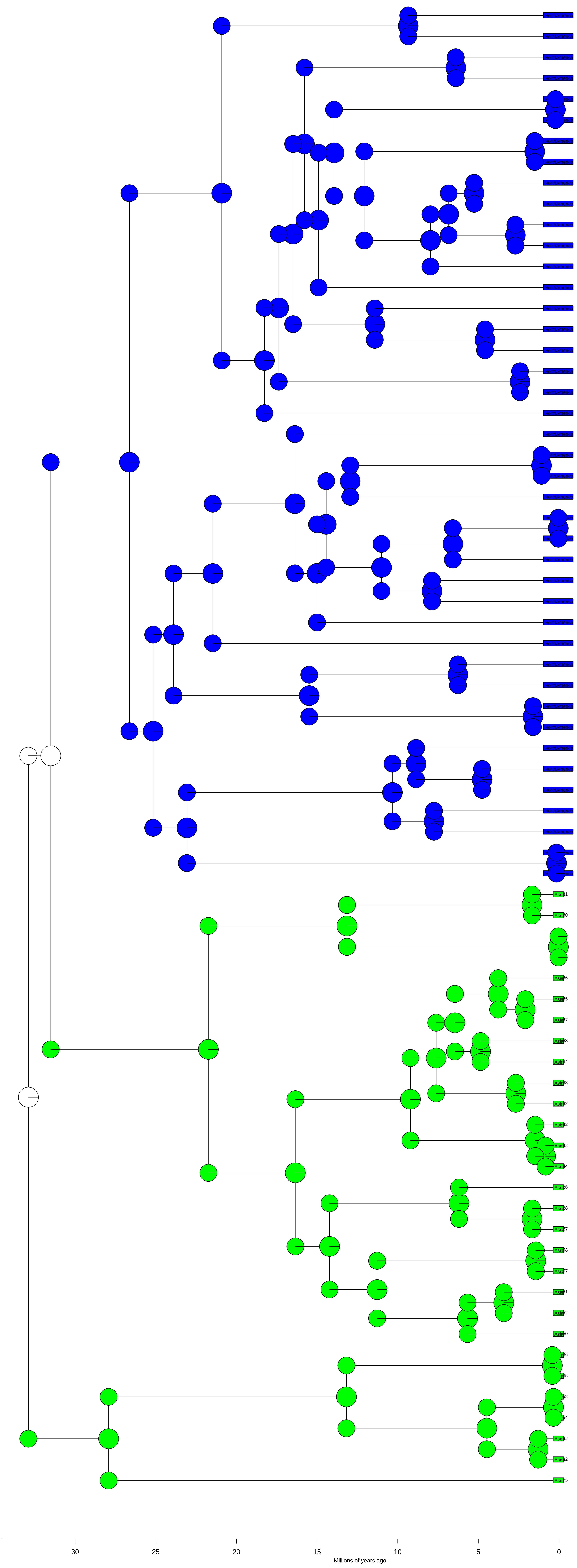

Viperidae BioGeoBEARS DEC

ancstates: global optim, 3 areas max. d=0.0022; e=4e−04; j=0; LnL=−191.29

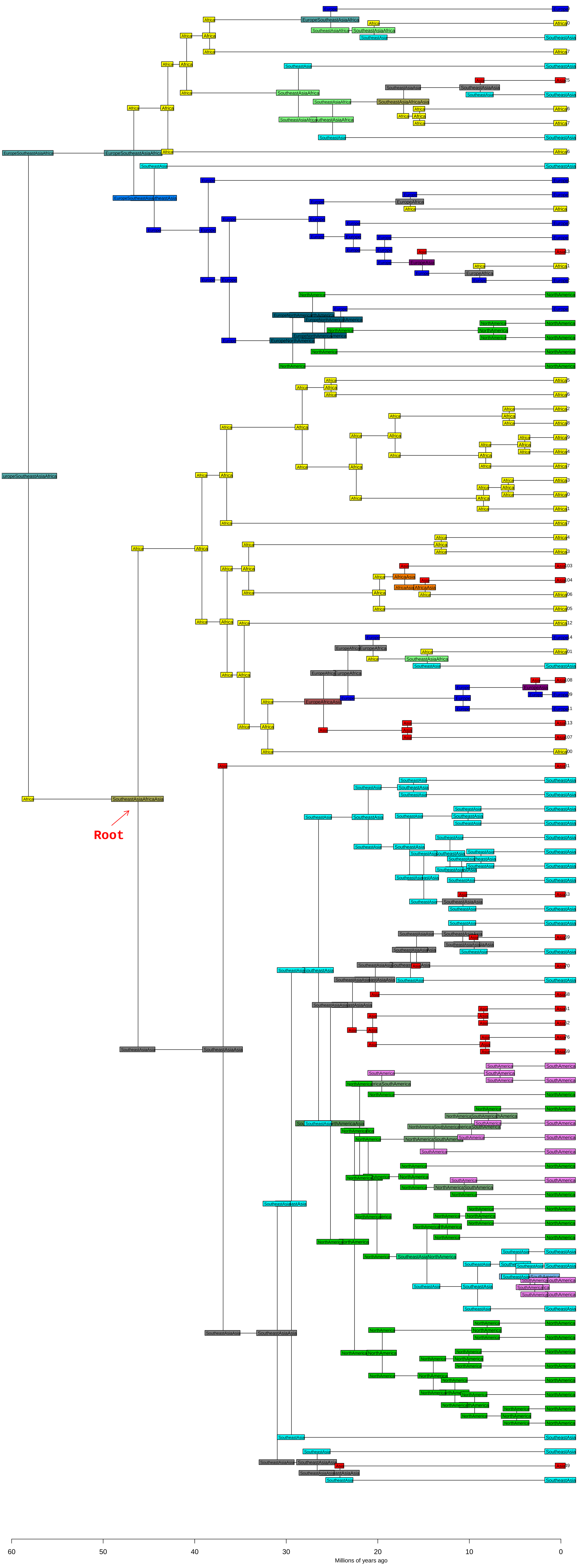

Viperidae BioGeoBEARS DEC

ancstates: global optim, 3 areas max. d=0.0022; e=4e−04; j=0; LnL=−191.29

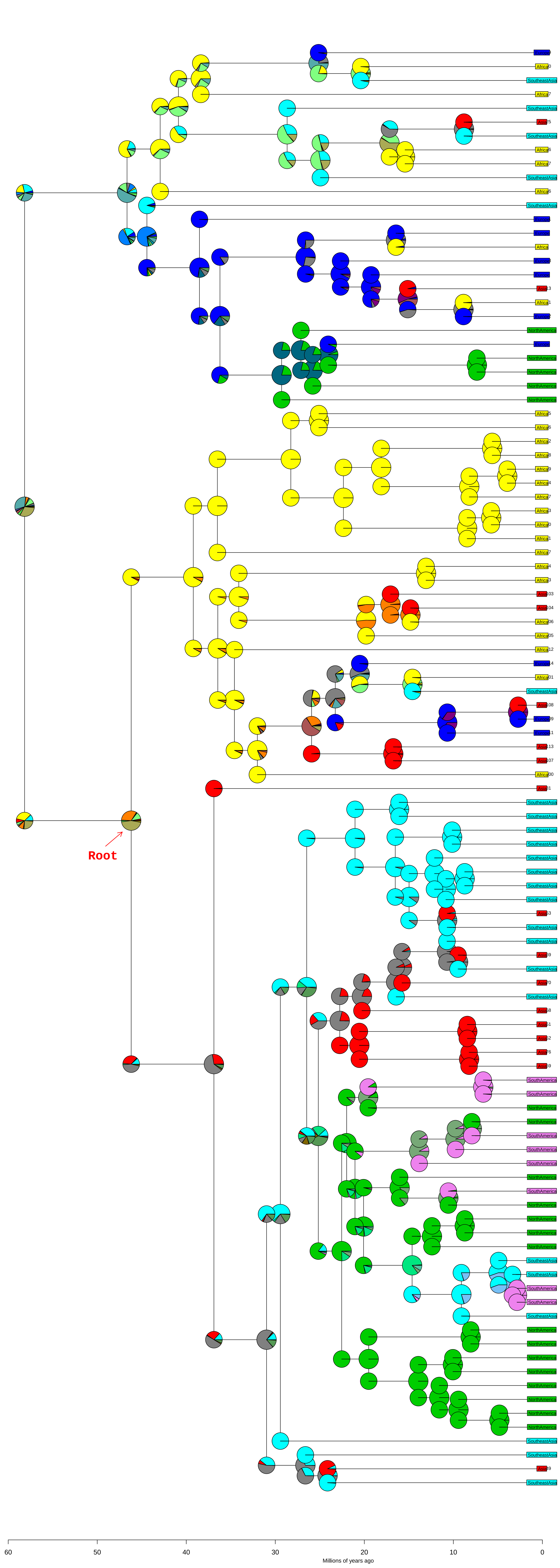

Dipodoidea BioGeoBEARS DEC

ancstates: global optim, 3 areas max. d=0.0028; e=0; j=0; LnL=−37.43

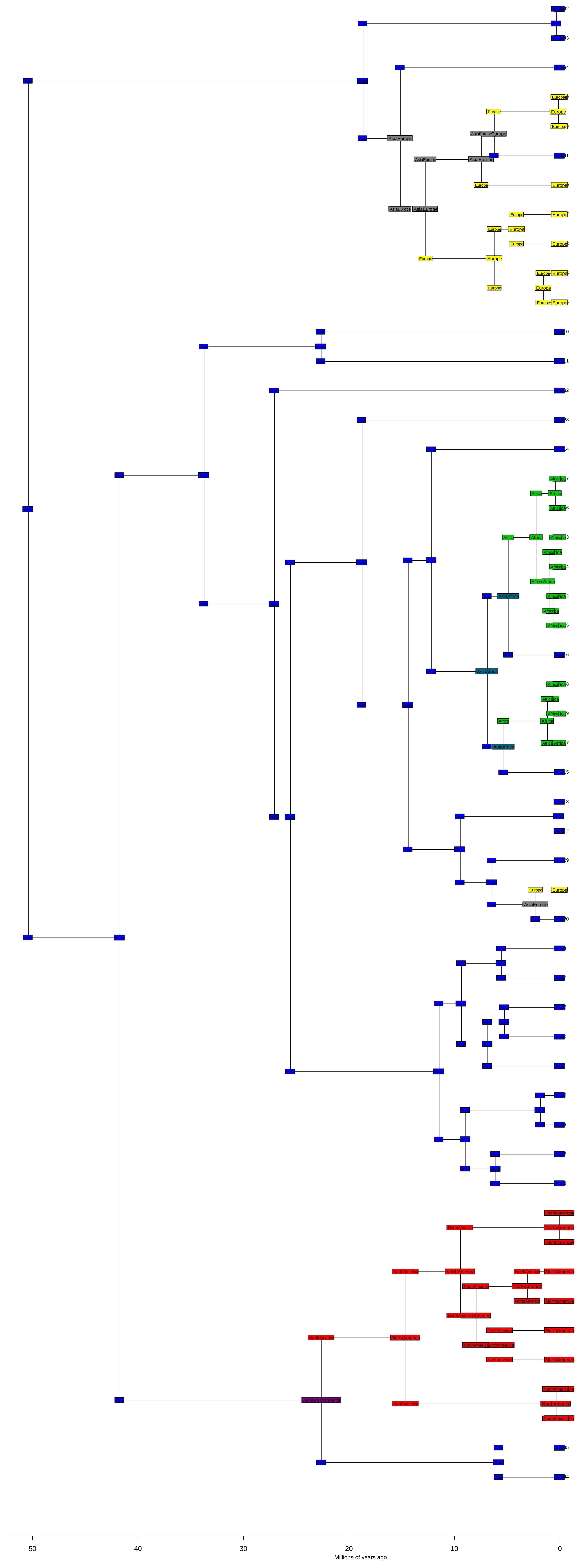

Dipodoidea BioGeoBEARS DEC

ancstates: global optim, 3 areas max. d=0.0028; e=0; j=0; LnL=−37.43

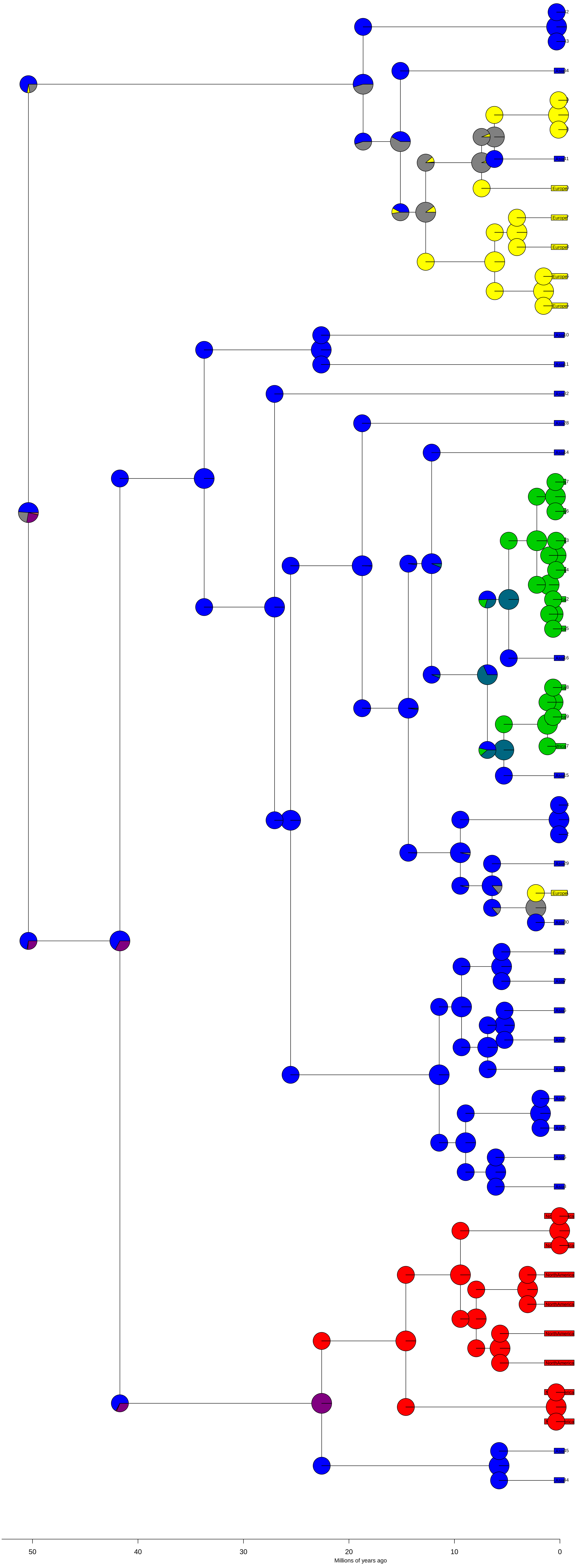

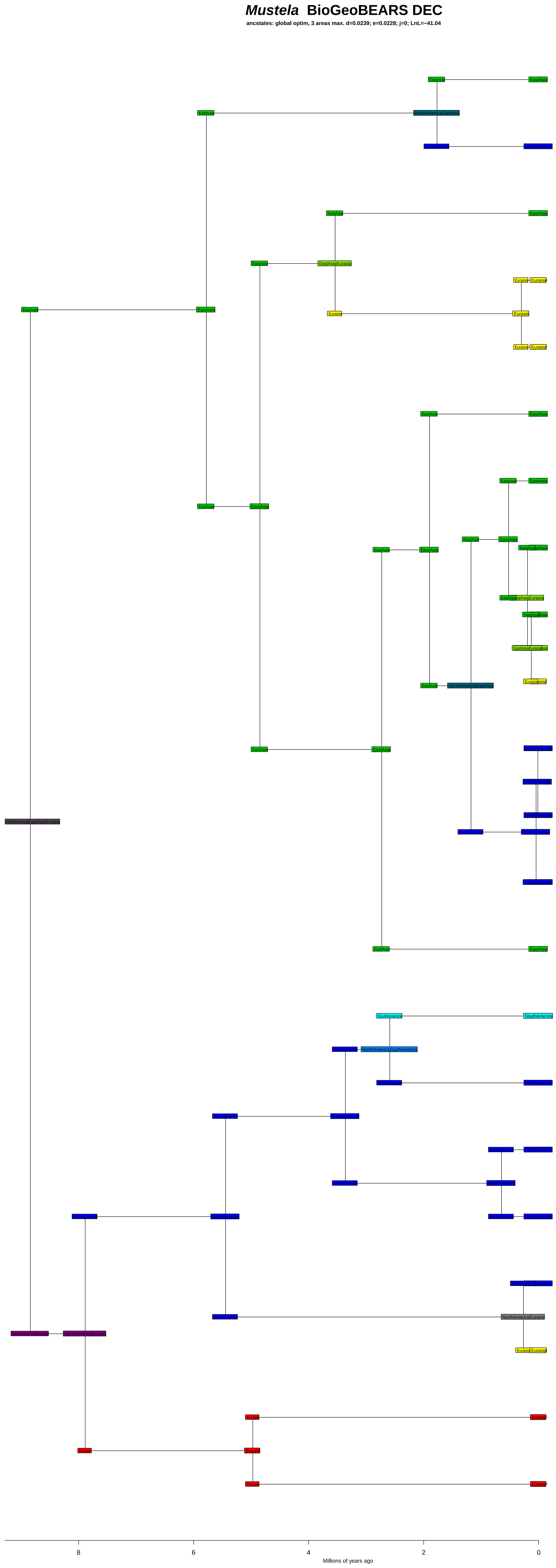
Mustela BioGeoBEARS DEC
ancstates: global optim, 3 areas max. d=0.0239; e=0.0228; j=0; LnL=−41.04
EastAsia
NorthAmericaEastAsia
EastAsiaEurasia
Eurasia
NorthAmericaEastAsiaEurope
SouthAmerica
NorthAmericaSouthAmerica
NorthAmericaEurasia
8
6
4
2
0
Millions of years ago

Mustela **BioGeoBEARS DEC**

ancstates: global optim, 3 areas max. d=0.0239; e=0.0228; j=0; LnL=−41.04

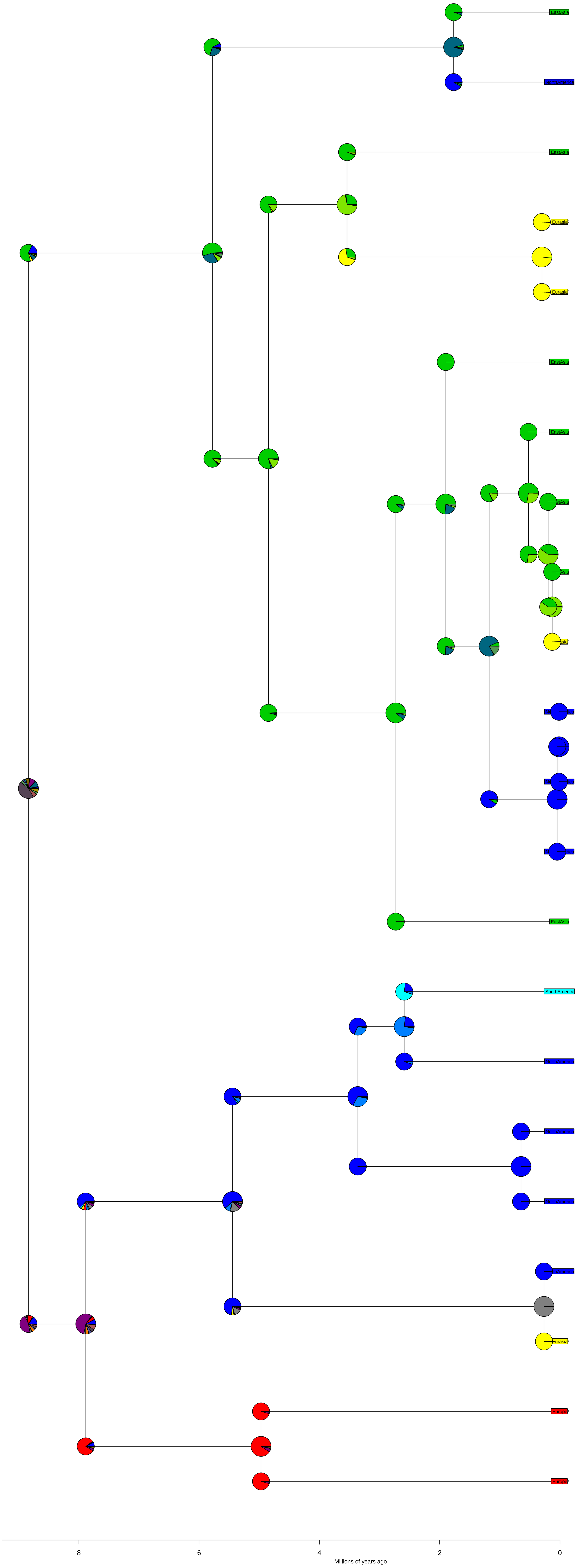

Microtus BioGeoBEARS DEC

ancstates: global optim, 3 areas max. d=0; e=0; j=0; LnL=−4.28

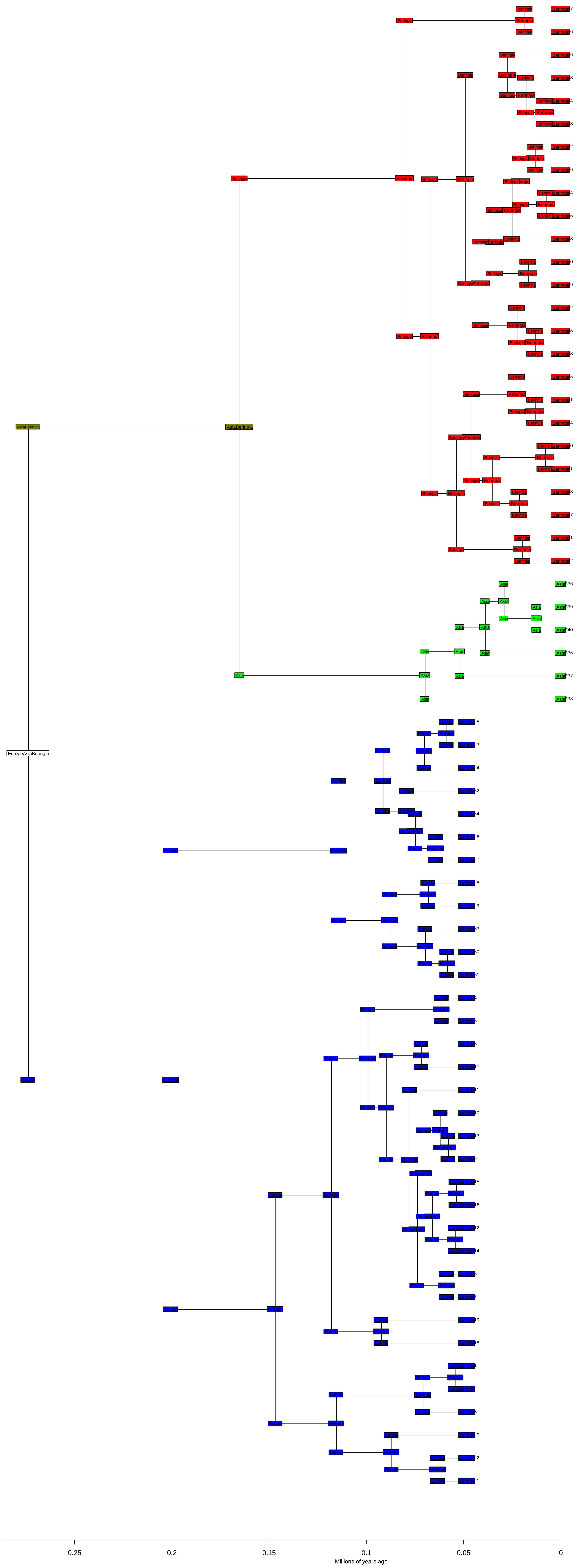

Microtus BioGeoBEARS DEC

ancstates: global optim, 3 areas max. d=0; e=0; j=0; LnL=−4.28

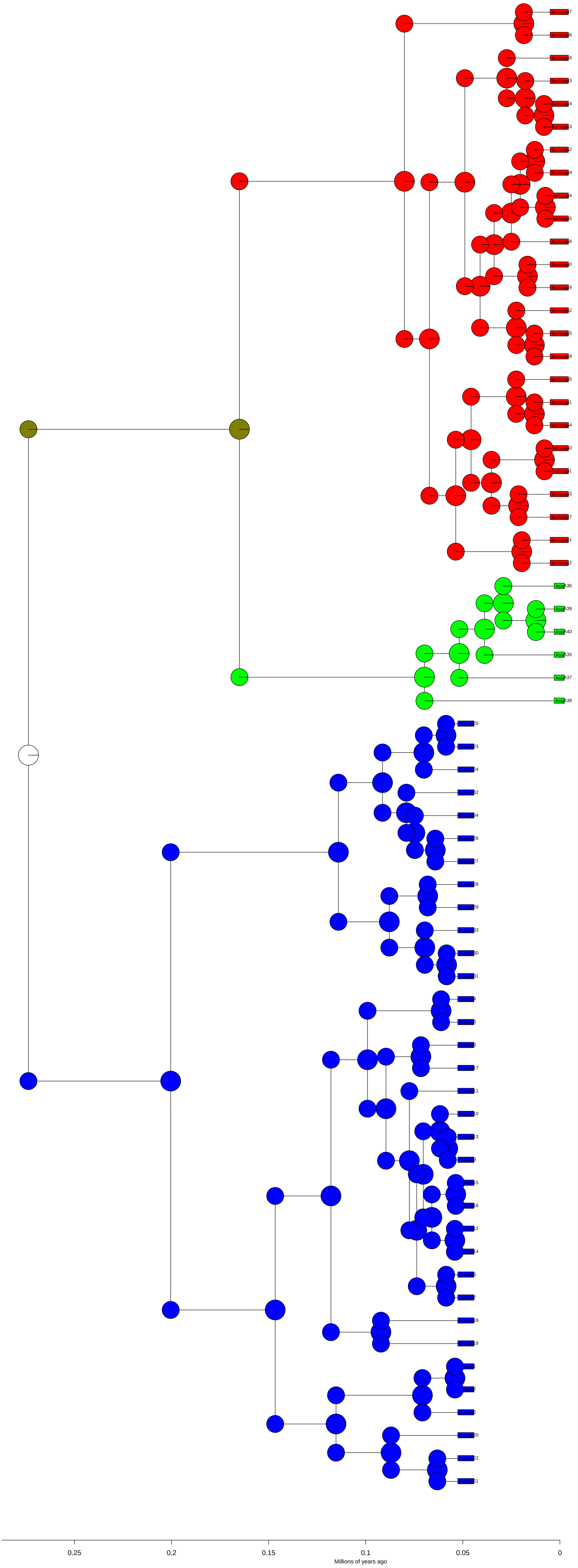

Myotis BioGeoBEARS DEC

ancstates: global optim, 2 areas max. d=0.0042; e=0; j=0; LnL=−46.19

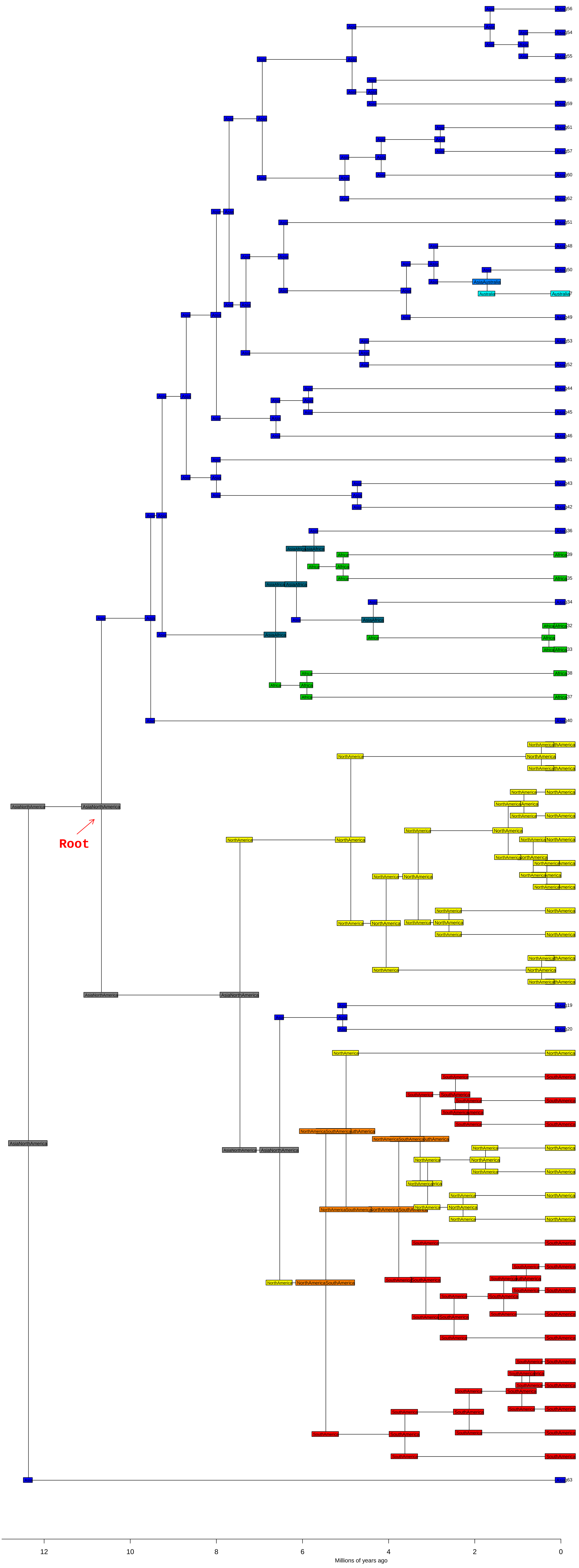

Myotis BioGeoBEARS DEC

ancstates: global optim, 2 areas max. d=0.0042; e=0; j=0; LnL=-46.19

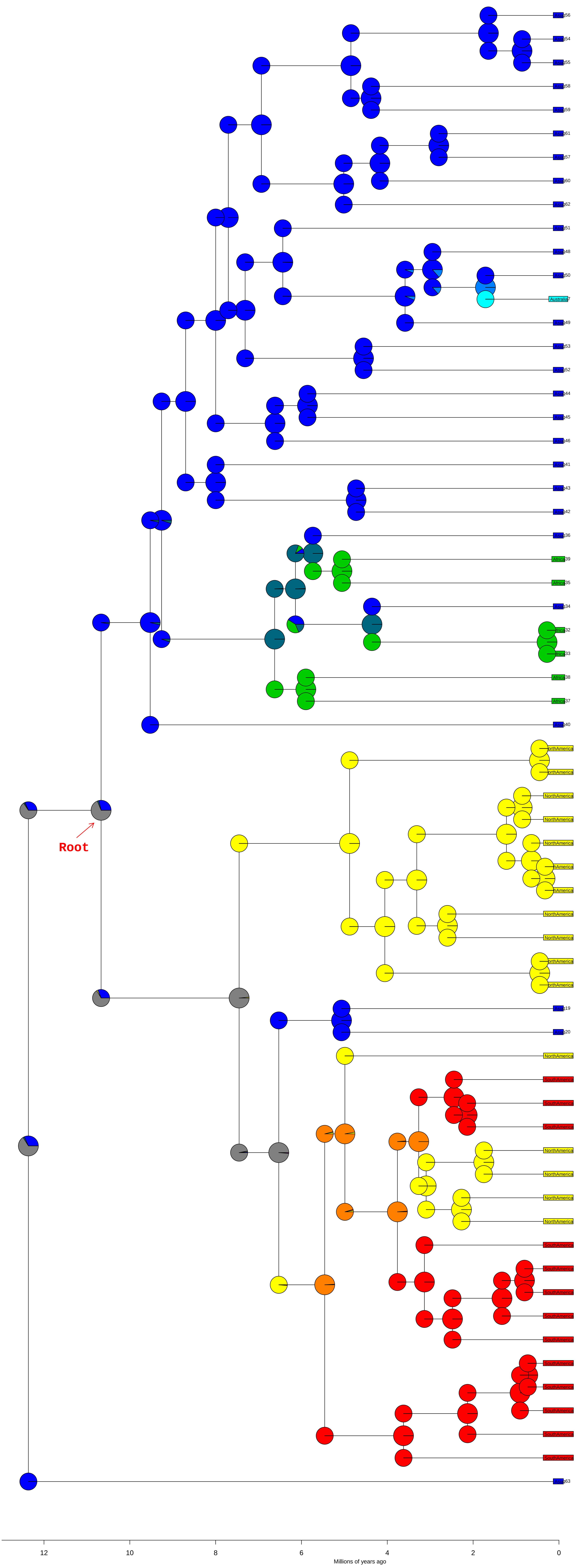

Leporidae BioGeoBEARS DEC

ancstates: global optim, 5 areas max. d=0.0172; e=0.0178; j=0; LnL=−34.80

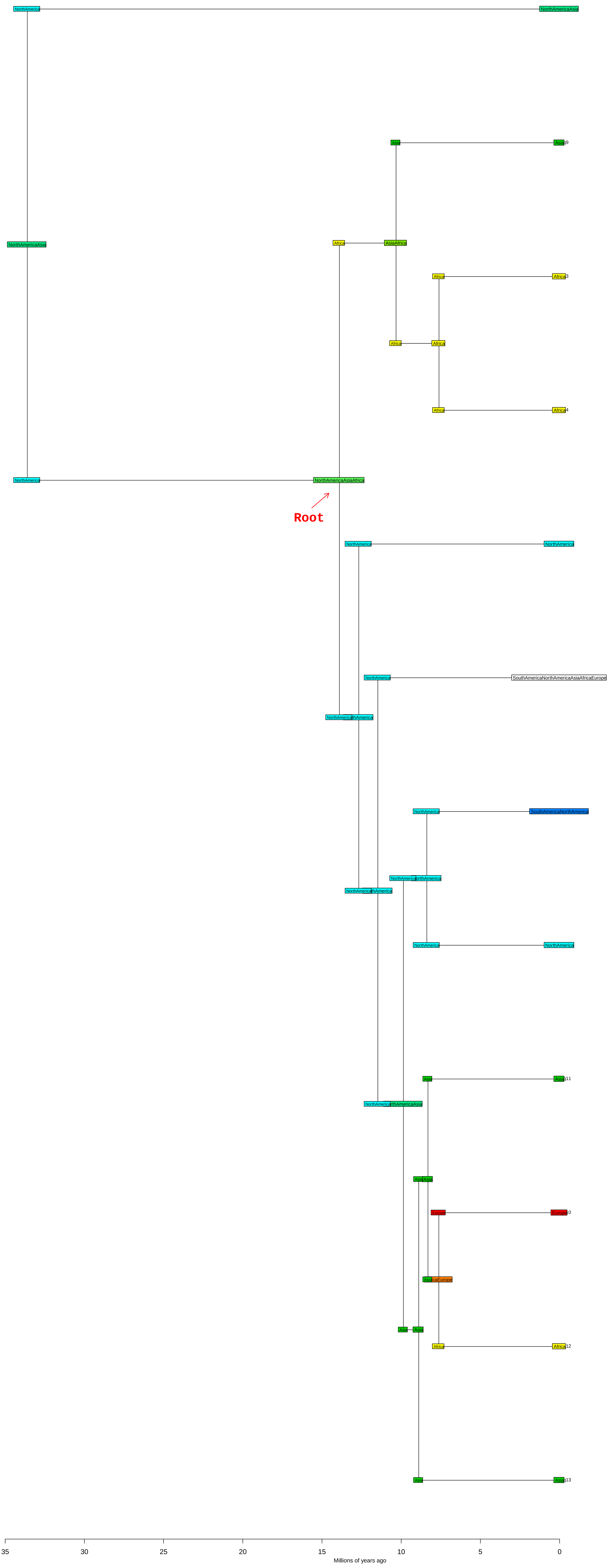

Leporidae BioGeoBEARS DEC

ancstates: global optim, 5 areas max. d=0.0172; e=0.0178; j=0; LnL=-34.80

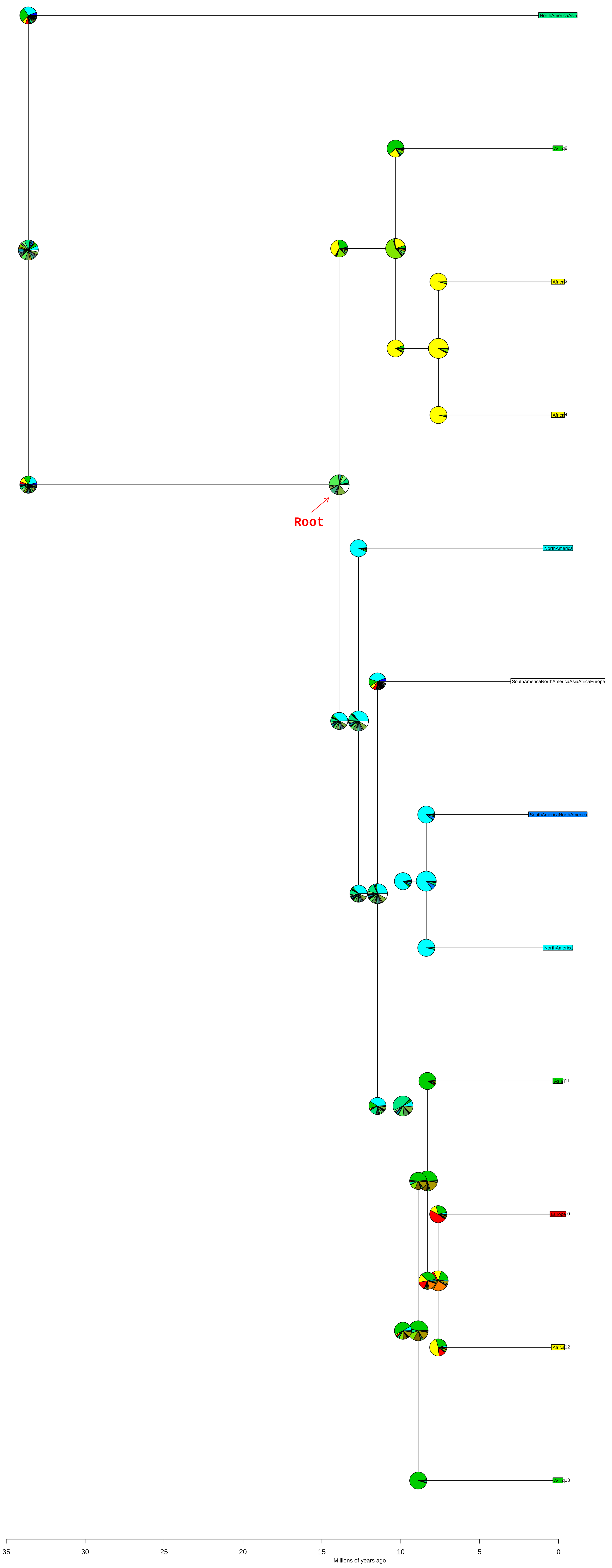

Melaphidina BioGeoBEARS DEC

ancstates: global optim, 3 areas max. d=0.0015; e=1e−04; j=0; LnL=−6.68

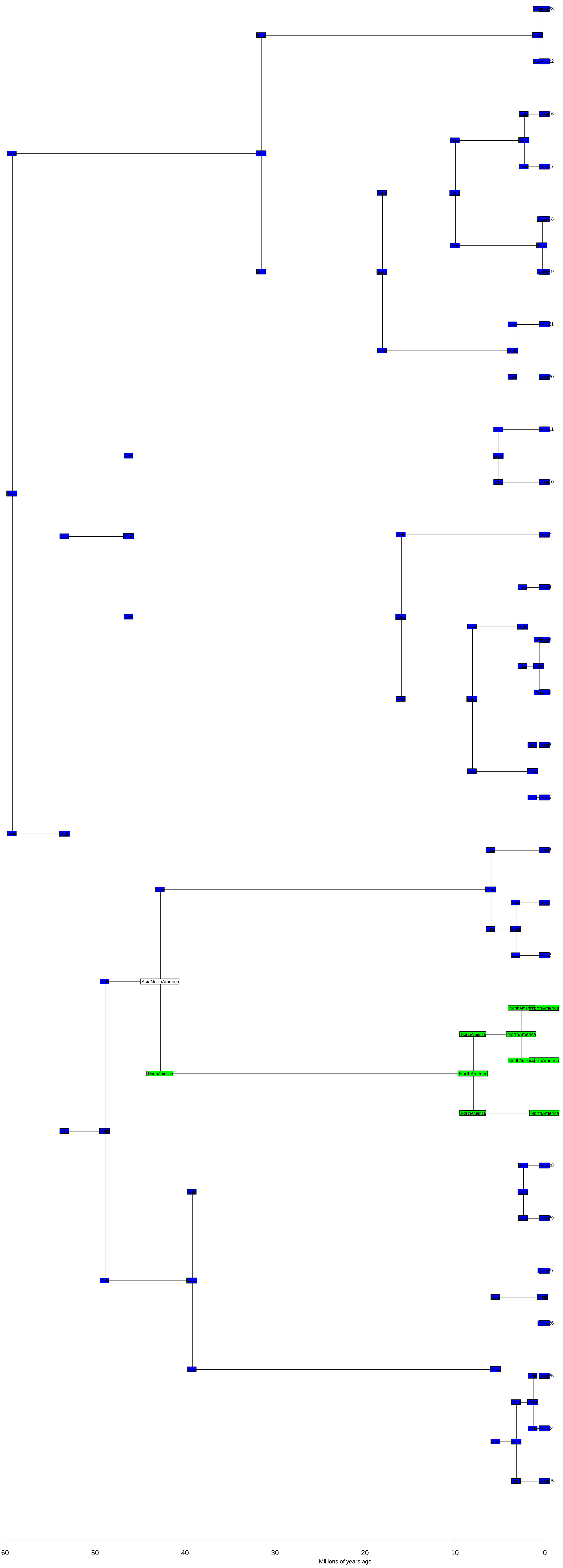

Melaphidina BioGeoBEARS DEC

ancstates: global optim, 3 areas max. d=0.0015; e=1e−04; j=0; LnL=−6.68

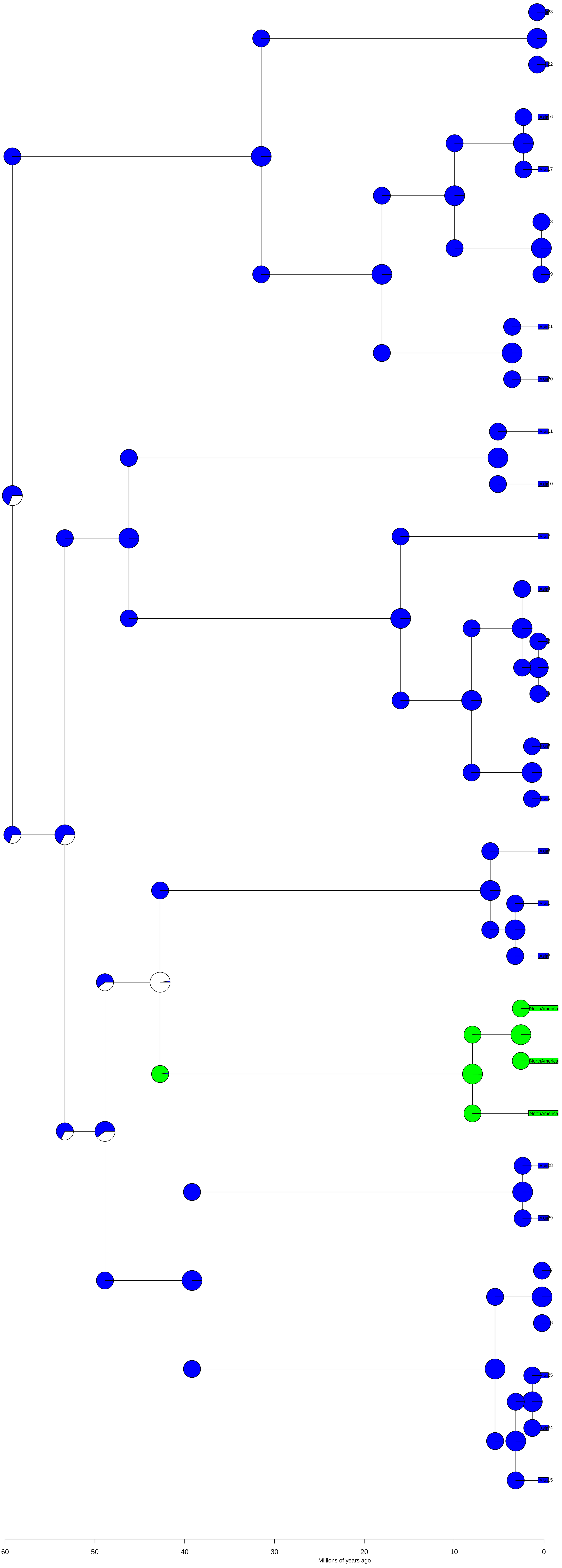

Limnogonus BioGeoBEARS DEC

ancstates: global optim, 4 areas max. d=0.0025; e=0; j=0; LnL=−43.18

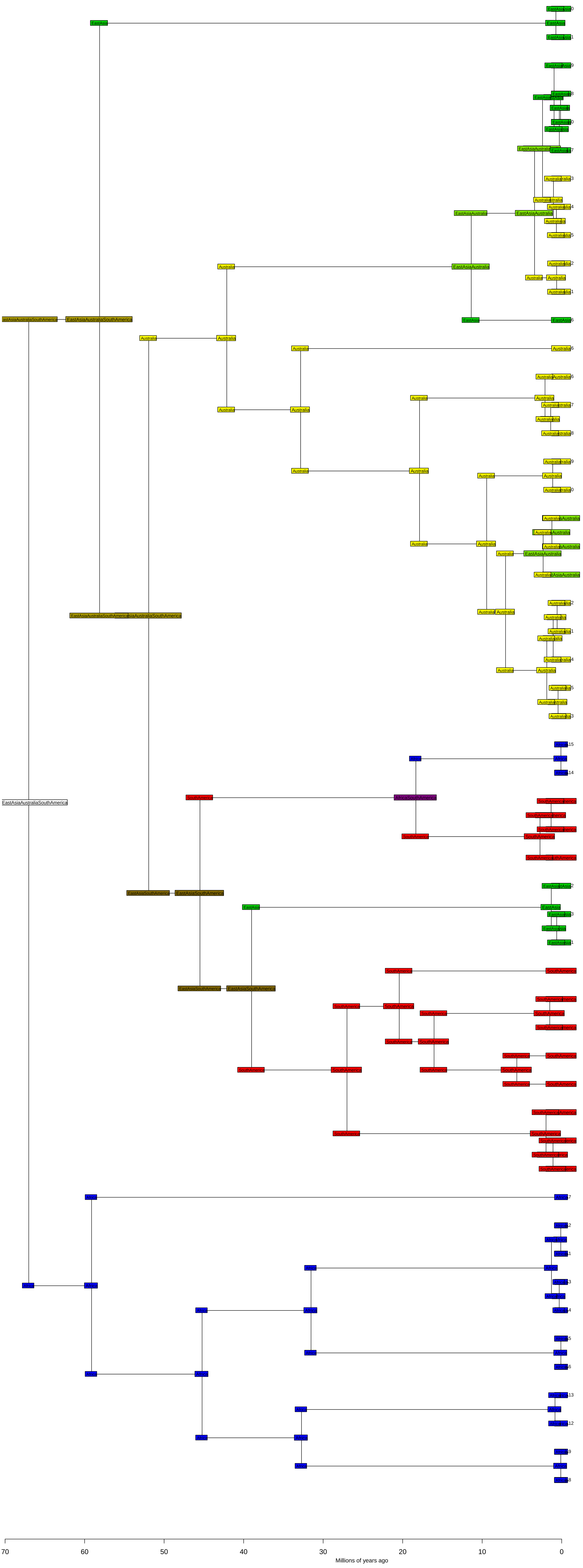

Limnogonus BioGeoBEARS DEC

ancstates: global optim, 4 areas max. d=0.0025; e=0; j=0; LnL=−43.18

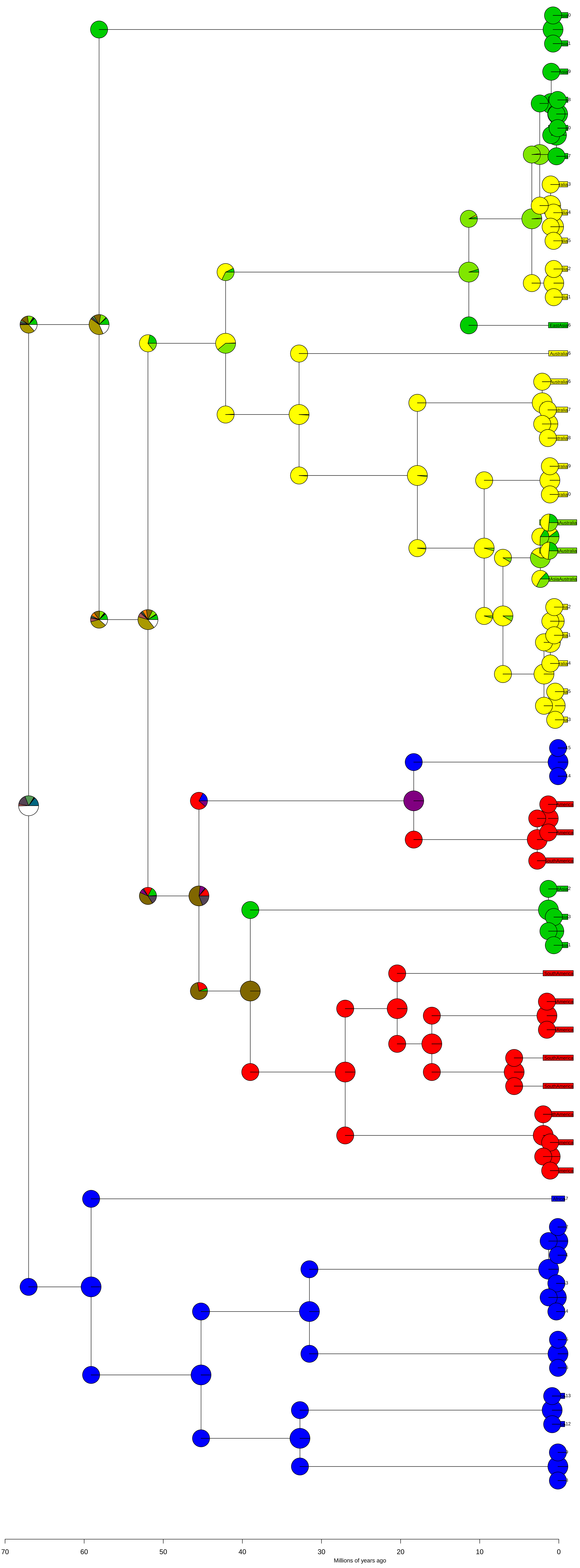

Aporini BioGeoBEARS DEC

ancstates: global optim, 4 areas max. d=0.0038; e=0; j=0; LnL=−39.01

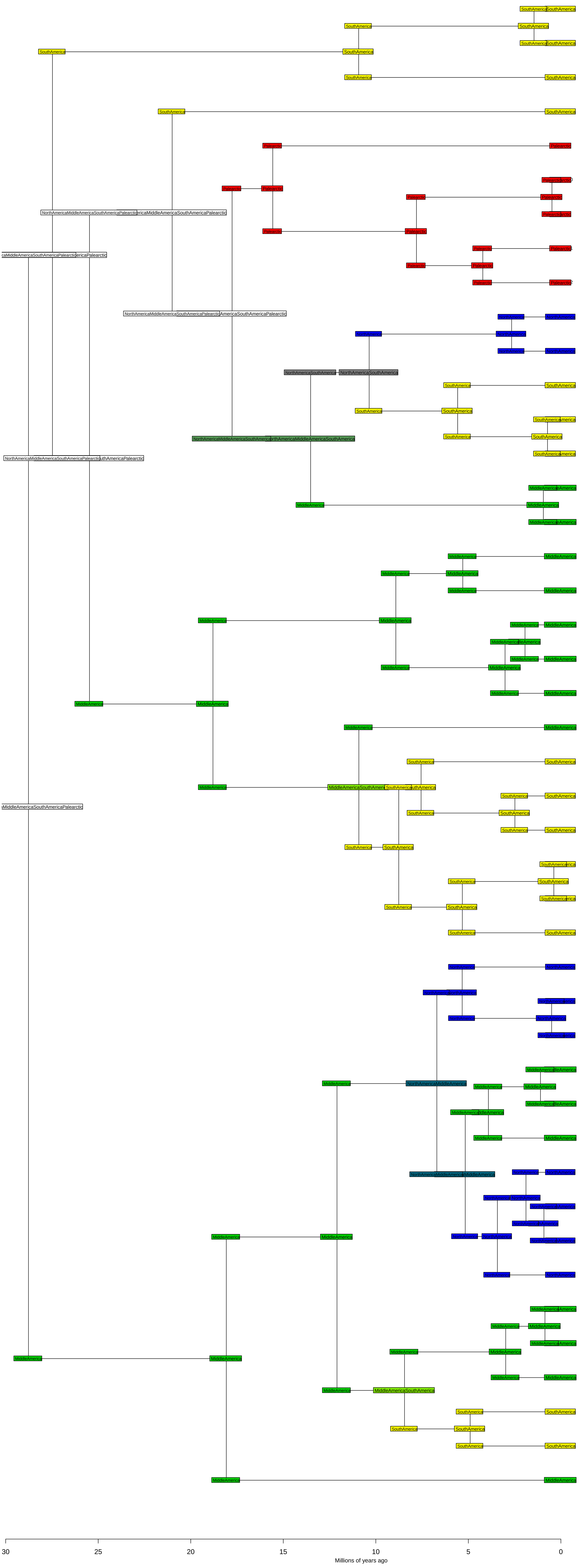

Aporini BioGeoBEARS DEC

ancstates: global optim, 4 areas max. d=0.0038; e=0; j=0; LnL=−39.01

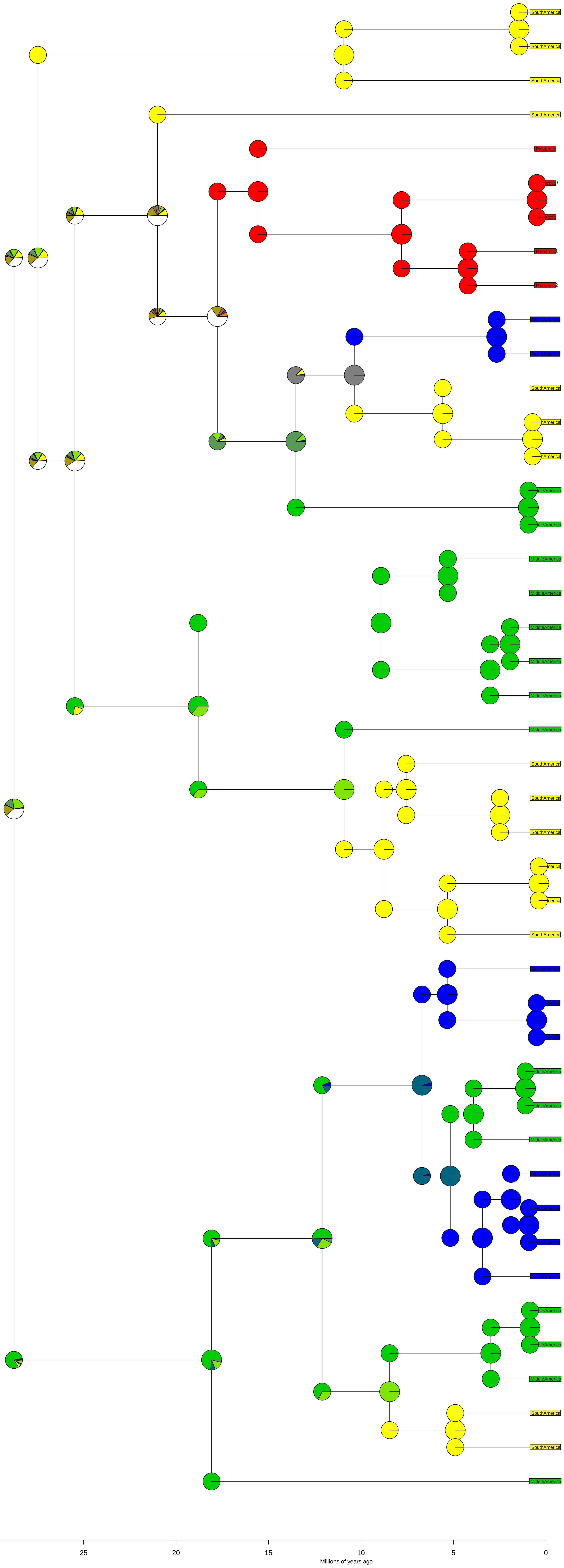

Papilionidae BioGeoBEARS DEC

ancstates: global optim, 4 areas max. d=0.0049; e=0; j=0; LnL=−586.41

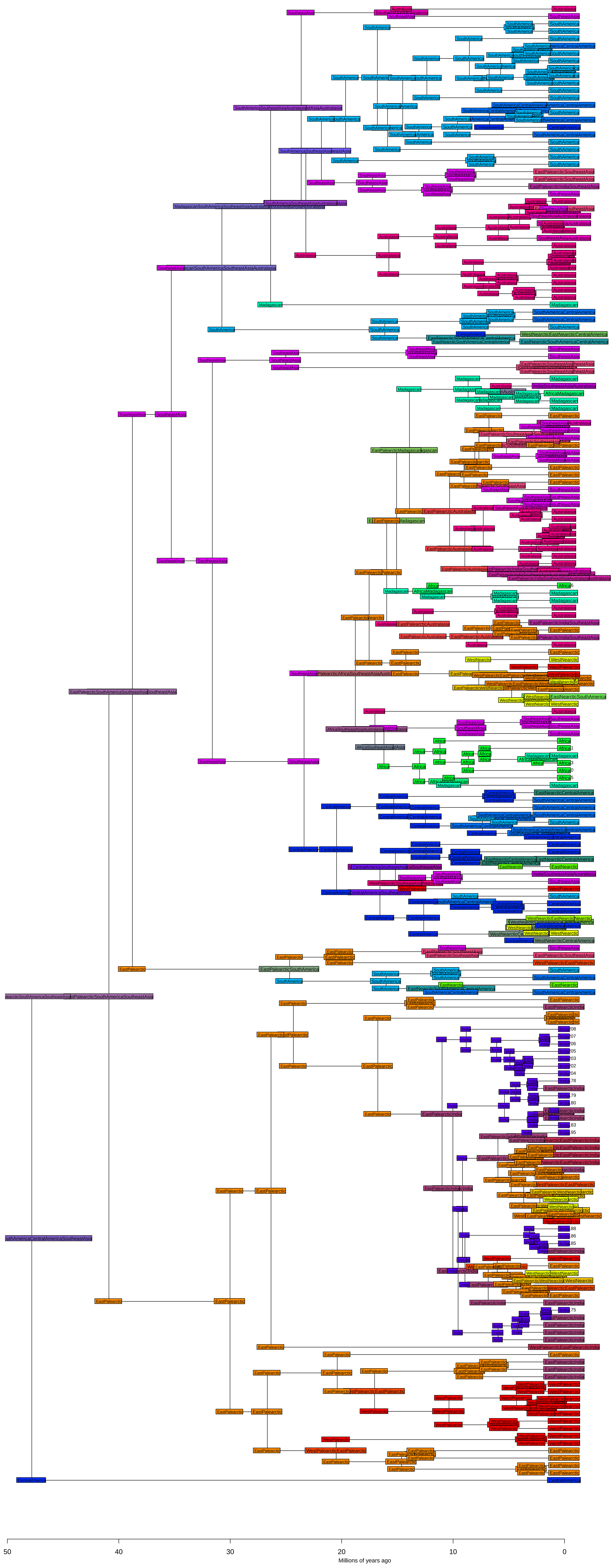

Papilionidae BioGeoBEARS DEC

ancstates: global optim, 4 areas max. d=0.0049; e=0; j=0; LnL=−586.41

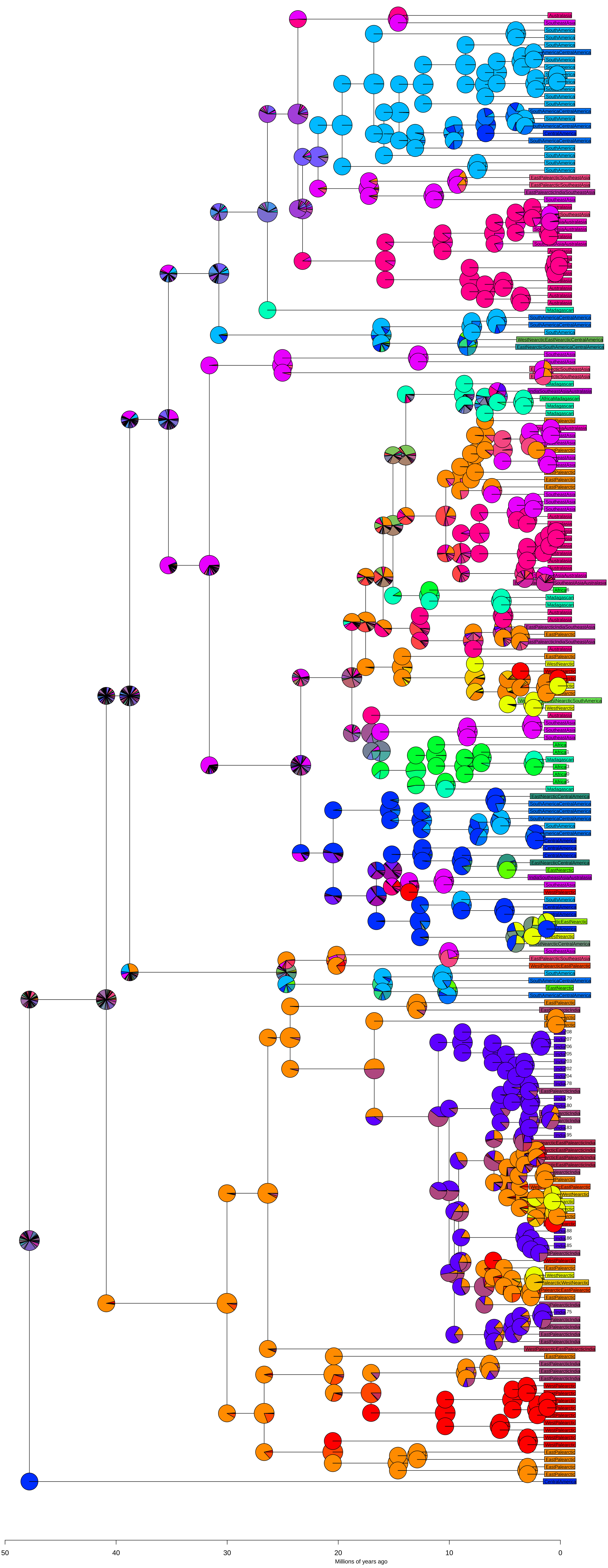

Apaturinae BioGeoBEARS DEC

ancstates: global optim, 3 areas max. d=0.0025; e=0.0073; j=0; LnL=−29.83

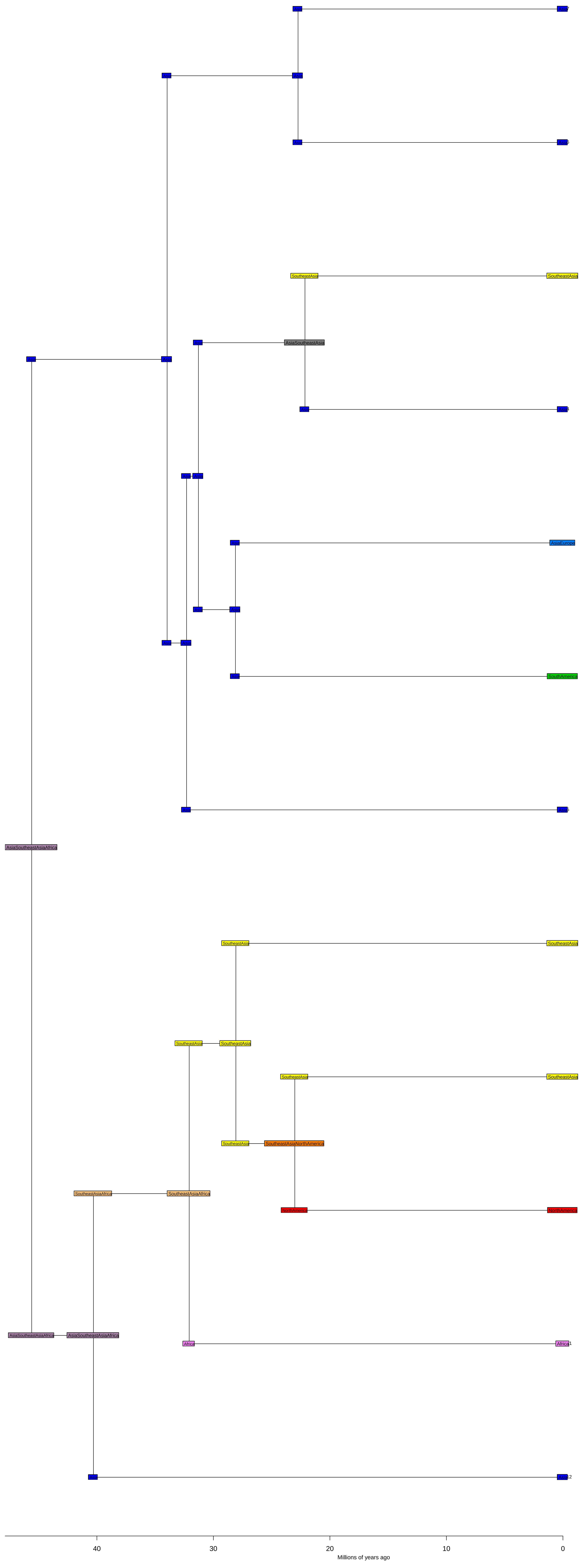

Apaturinae BioGeoBEARS DEC

ancstates: global optim, 3 areas max. d=0.0025; e=0.0073; j=0; LnL=−29.83

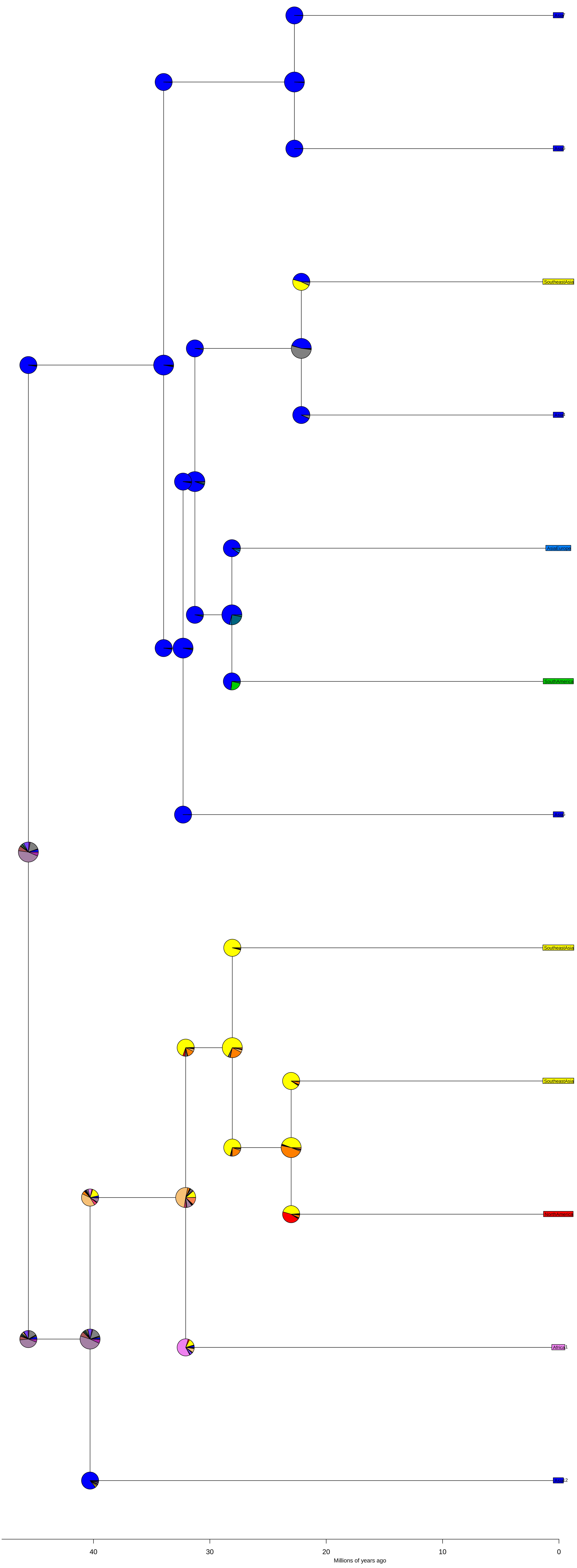

Sect. *Phalloideae* BioGeoBEARS DEC

ancstates: global optim, 3 areas max. d=7e−04; e=0; j=0; LnL=−44.08

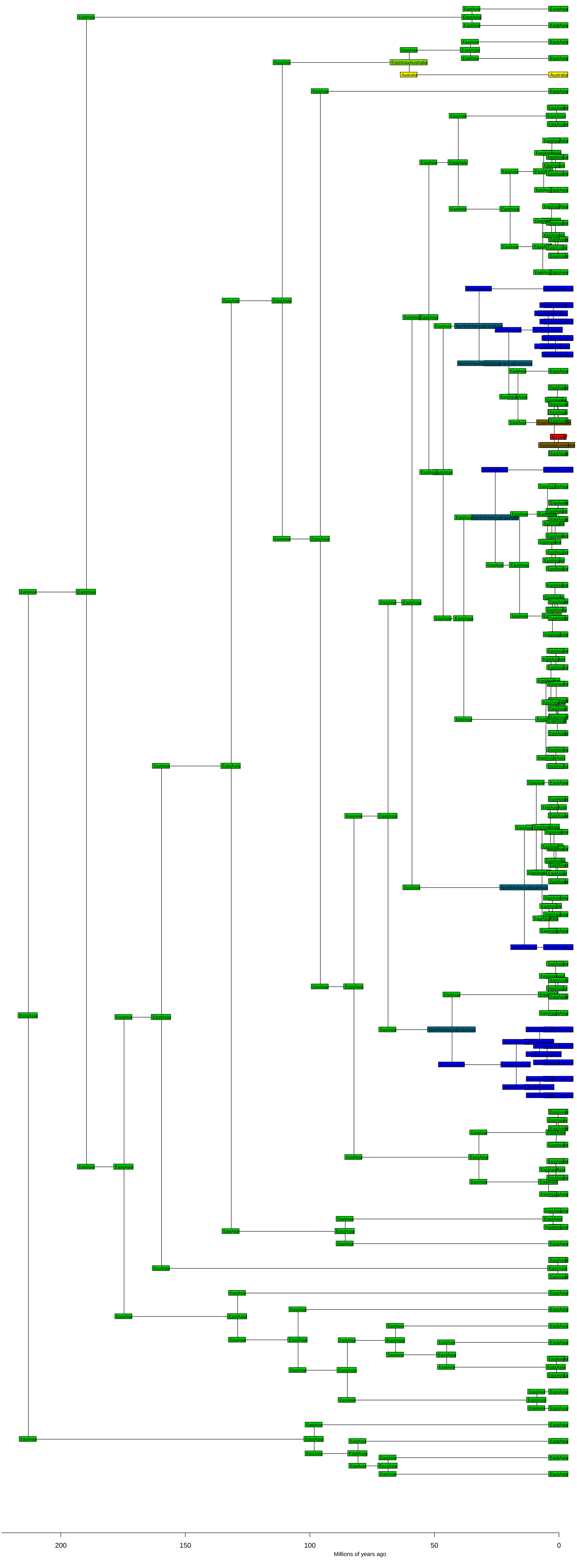

Sect. *Phalloideae* BioGeoBEARS DEC

ancstates: global optim, 3 areas max. d=7e−04; e=0; j=0; LnL=−44.08

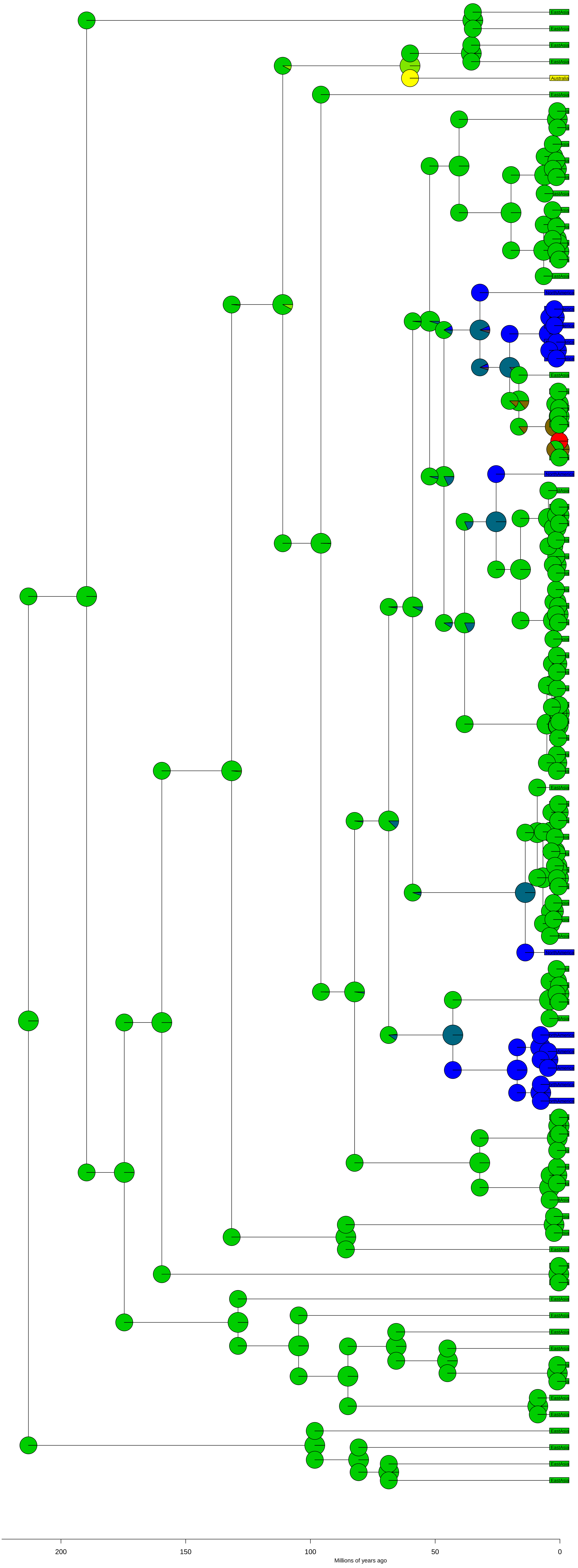

Supplement: nwz035_Supplemental_File [file nwz035_supplemental_file.pdf]
